# Ru‐NHC‐Catalyzed Asymmetric Hydrogenation of 2‐Quinolones to Chiral 3,4‐Dihydro‐2‐Quinolones

**DOI:** 10.1002/anie.202108503

**Published:** 2021-09-22

**Authors:** Tianjiao Hu, Lukas Lückemeier, Constantin Daniliuc, Frank Glorius

**Affiliations:** ^1^ Westfälische Wilhelms-Universität Münster Organisch-Chemisches Institut Corrensstrasse 36 48149 Münster Germany

**Keywords:** asymmetric hydrogenation, dihydroquinolone, N-heterocyclic carbene, quinolone, ruthenium

## Abstract

Direct enantioselective hydrogenation of unsaturated compounds to generate chiral three‐dimensional motifs is one of the most straightforward and important approaches in synthetic chemistry. We realized the Ru(II)‐NHC‐catalyzed asymmetric hydrogenation of 2‐quinolones under mild reaction conditions. Alkyl‐, aryl‐ and halogen‐substituted optically active dihydro‐2‐quinolones were obtained in high yields with moderate to excellent enantioselectivities. The reaction provides an efficient and atom‐economic pathway to construct simple chiral 3,4‐dihydro‐2‐quinolones. The desired products could be further reduced to tetrahydroquinolines and octahydroquinolones.

## Graphical abstract

Ruthenium‐NHC‐catalyzed asymmetric hydrogenation of 2‐quinolones to simple 3,4‐dihydro‐2‐quinolones has been developed with high yields and moderate to excellent enantioselectivities under mild conditions.

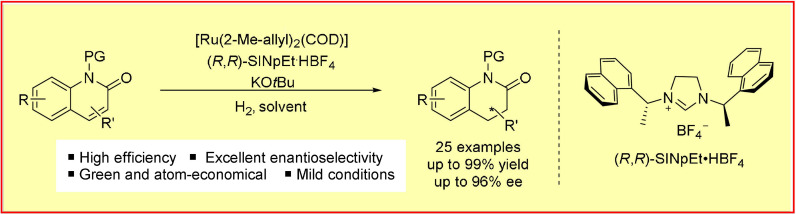

Dihydroquinolones, which widely exist in natural products and marketed pharmaceuticals, are known as a class of important heterocycles and exhibit significant biological activities.^[1]^ For example, aripiprazole (antipsychotic drug), carteolol (non‐selective beta blocker), vesnarinone (cardiotonic agent), cilostazol (phosphodiesterase‐3 inhibitor), as well as melosuavne[^1c^, ^1g^] are drugs or medically useful natural products which all contain the 3,4‐dihyro‐2‐quinolone motif (Scheme 1). In addition, dihydroquinolones could potentially become versatile intermediates which could be further transformed to several other common heterocycles such as tetrahydroquinolines^[2]^ or octahydroquinolones.^[3]^

**Scheme 1:**
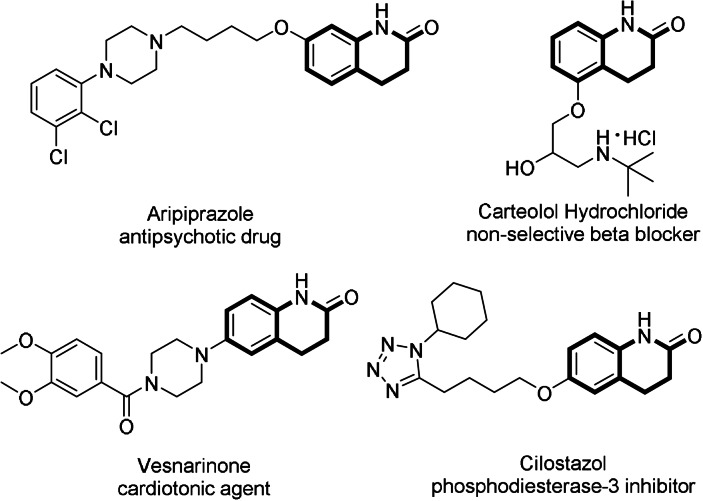
Representative marketed pharmaceuticals containing 2‐dihydroquinolone moieties.

Although several methods have been explored to form achiral and racemic dihydroquinolones,^[4]^ the construction of optically active dihydroquinolones, especially dihydro‐2‐quinolones, is still rare and highly desirable. Currently, there are two main approaches to access chiral dihydro‐2‐quinolones. The first one is transition‐metal‐catalyzed asymmetric conjugate addition,^[5]^ however, most examples are focusing on arylations. In 2019, Harutyunyan^[6]^ explored asymmetric alkylation using Grignard reagents to form alkyl‐substituted dihydro‐2‐quinolones, although harsh conditions and limited scope remain an issue due to low activity of 2‐quinolone (Scheme 2 a). Alternatively, Cao,^[7]^ Gong,^[8]^ and Xiao^[9]^ developed an asymmetric [4+2] cycloaddition to form the above‐mentioned motifs. Again, specific functional groups, such as vinyl or ethynyl, are required (Scheme 2 b). Thus, developing a more general and atom‐economic approach to synthesize simple dihydro‐2‐quinolones is highly demanding.

**Scheme 2:**
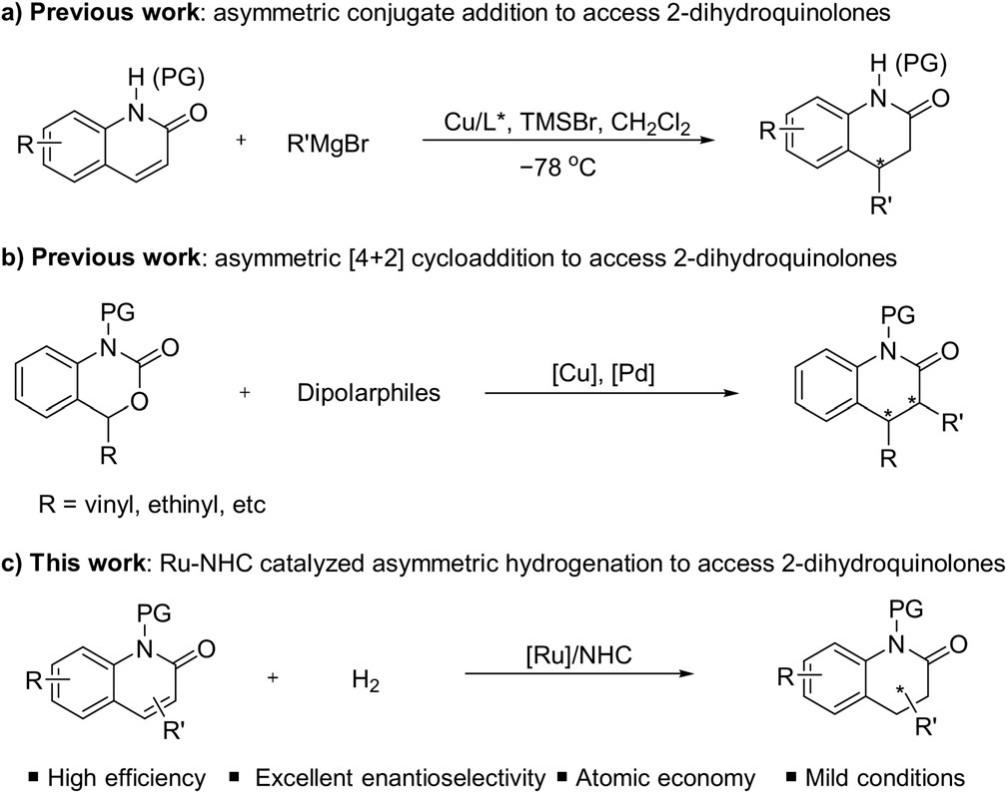
a, b) Previous work to access chiral 2‐dihydroquinolones. c) This work.

Direct hydrogenation of quinolone derivatives to generate dihydroquinolone‐containing bioactive molecules is one of the most straightforward and atom‐economic approaches and thus has the potential to be applied in large‐scale synthesis.[^1b^, ^1e^, ^1f^, ^10^] Surprisingly, direct asymmetric hydrogenation of 2‐quinolones, especially simple 2‐quinolones to dihydroquinolones, has rarely been realized.^[11]^ Hydrogenation of 2‐quinolones is hampered by the low reactivity of cyclic α,β‐conjugated amides^[6]^ and the poisoning effect of the nitrogen atoms.^[12]^ Undoubtedly, achieving this goal would remove a huge obstacle in the discovery of potential drug targets.

Promisingly, several privileged asymmetric hydrogenation catalyst systems have emerged during the past decades.^[13]^ Among these powerful catalysts, the Ru–NHC complex developed by our group exhibited excellent performance for the hydrogenation of many heteroarenes and nonaromatic cyclic olefins.^[14]^ Inspired by our previous work, we achieved the direct asymmetric hydrogenation of 2‐quinolones using our Ru–NHC catalyst (Scheme 2 c).

We started our study with commercial substrate **1 a**. An initial experiment was conducted under 70 bar H_2_ in hexane at room temperature (Table 1). Unfortunately, no desired product was detected (Table 1, entry 1). According to our group's previous work, unprotected quinolone **1 a** would tautomerize to quinolin‐2‐ol, which is more stable.^[14g]^ Another possible reason would be the poisoning effect of the free amide group on the catalyst.^[12]^ Then, methyl‐protected substrate **1 b** was tested (Table 1, entry 2). We were pleased to find that the reaction occurred smoothly and gave the desired product with 93:7 e.r. We subsequently screened solvents and pressure (Table 1, entries 3–8) and found that the enantiomeric ratio increased slightly to 94:6 when the reaction was performed in diethyl ether (Table 1, entry 7). H_2_ pressure had no effect on the yield and enantioselectivity (Table 1, entry 8). Finally, the enantiomeric ratio was improved to 95:5 when the temperature was decreased to 15 °C (Table 1, entry 9).

**Table 1:** Optimization of the reaction conditions.^[a]^ 
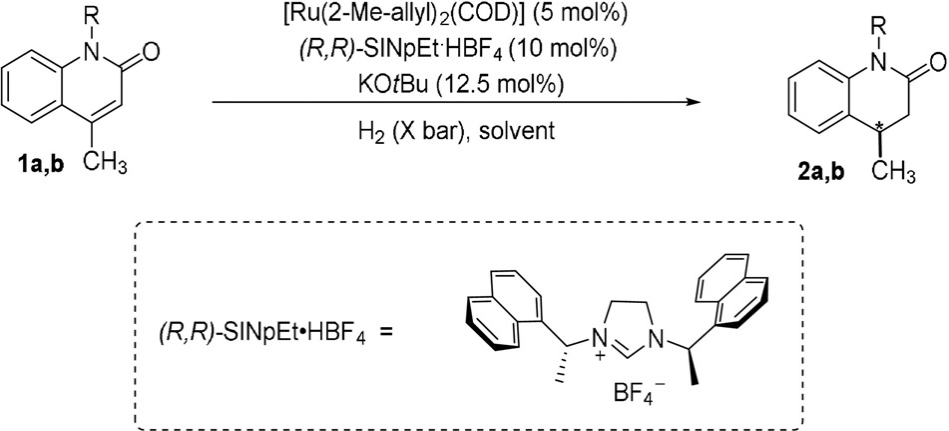

Entry	R	Solvent	Conversion [%]^[b]^	e.r.^[c]^
1	H (**1 a**)	*n*‐hexane	–	–
2	Me (**1 b**)	*n*‐hexane	>99	93:7
3	Me (**1 b**)	toluene	>99	90.5:9.5
4	Me (**1 b**)	THF	>99	90.5:9.5
5	Me (**1 b**)	Et_2_O	>99	94:6
6	Me (**1 b**)	*t*‐amylOH	>99	82:18
7^[d]^	Me (**1 b**)	Et_2_O	>99	94:6
8^[e]^	Me (**1 b**)	Et_2_O	>99	94:6
9^[e,f]^	Me (**1 b**)	Et_2_O	>99	95:5

[a] General conditions: [Ru(COD)(2‐methylally)_2_] (0.01 mmol), KO*t*Bu (0.025 mmol), (*R*,*R*)‐SINpEt⋅HBF_4_ (0.02 mmol) were stirred at 70 °C in *n*‐hexane (0.33 mL) for 16 h, after which it was added to **1 a** or **1 b** (0.2 mmol) in solvent (1 mL), and the hydrogenation was performed at 25 °C under 70 bar H_2_ for 24 h. [b] Determined by GC–MS. [c] Determined by HPLC on a chiral stationary phase. [d] Reaction run under 40 bar H_2_. [e] Reaction run under 10 bar H_2_. [f] The reaction was performed at 15 °C.

With the optimized condition in hand, we investigated the substrate scope of the reaction. When the protecting group was changed to a benzyl group, the desired product was isolated in 98 % yield with 95:5 e.r. (**2 c**). Then, the influence of the substitution in 6‐position was studied. As shown in Scheme 3, when methyl or longer alkyl chains were introduced, the corresponding products were obtained in high yields and excellent enantioselectivities (**2 d**–**f**). Halogen substituents were also tolerated in this catalytic system, giving the products (**2 g**, **2 h**) smoothly in good enantiomeric ratios. Remarkably, dehalogenated byproduct was not detected. It is worth noting that when methoxy as an electron‐donating group was introduced, the e.r. value was increased to 97:3 (**2 i**). We postulate that although the methoxy in 6‐position seems far from the olefin, it still has influence on the electronic property of the reduced double bond. Additionally, aryl‐substituted products **2 j**, **2 k** were also obtained smoothly with e.r. values up to 98:2.

**Scheme 3:**
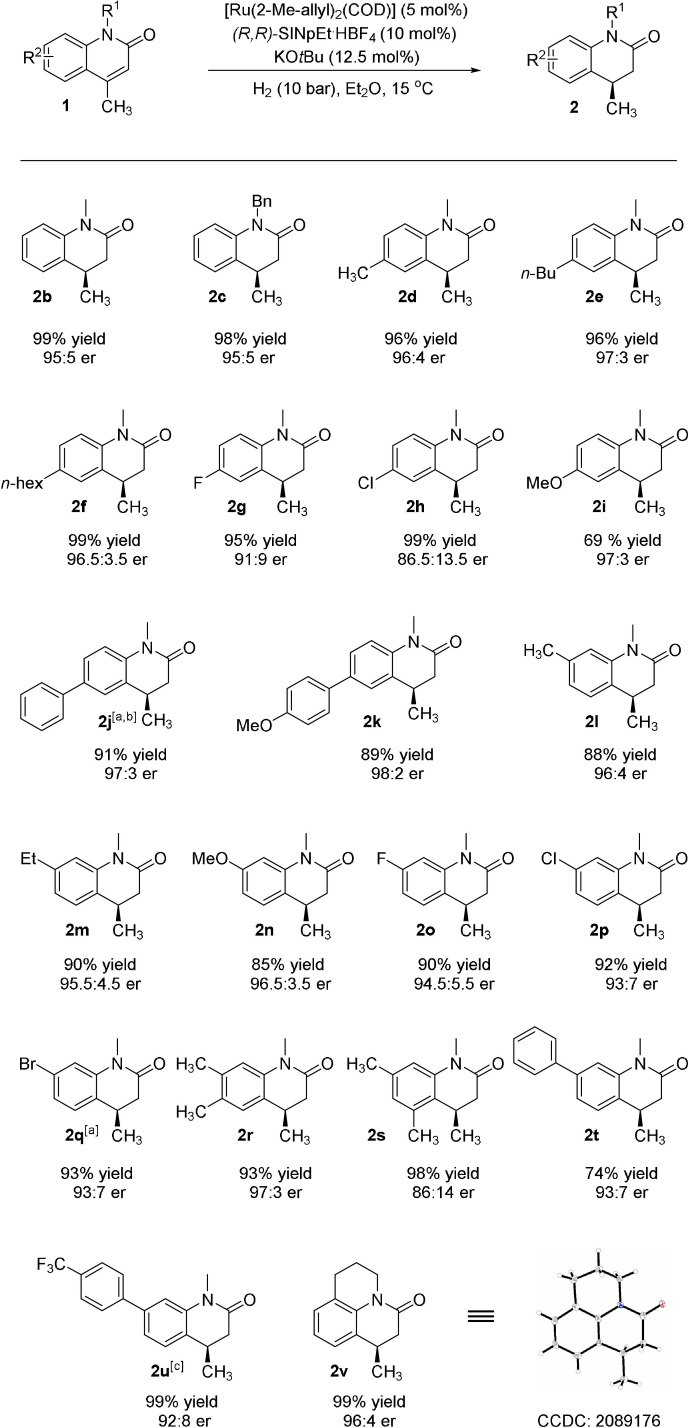
Substrate scope of substituted 2‐quinolones. General conditions: [Ru(COD)(2‐methylallyl)_2_] (0.01 mmol), KO*t*Bu (0.025 mmol), (*R*,*R*)‐SINpEt⋅HBF_4_ (0.02 mmol) were stirred at 70 °C in *n*‐hexane (0.33 mL) for 16 h, after which the mixture was added to **1** (0.2 mmol) in Et_2_O (1 mL). The hydrogenation was performed at 15 °C under 10 bar H_2_ for 24 h. Yields of isolated products are given. Enantiomeric ratio was determined by HPLC on a chiral stationary phase. [a] 0.1 mmol substrates were added. [b] 99:1 e.r. after recrystallization. [c] Enantiomeric ratio was determined by GC–FID.

To our delight, if these substitutions were moved to the 7‐position, the alkyl (**1 l**, **1 m**), phenyl (**1 t**, **1 u**), and halogen substrates (**1 o**–**1 q**) could be reduced successfully with high yield and excellent enantioselectivity. The bromo and chloro compounds (**2 p**, **2 q**) would be useful building blocks for further manipulation. We also investigated substitutions in 5‐ and 8‐positions. With a methyl substituent in the 5‐position, the enantioselectivity of the corresponding product **2 s** decreased. A remarkable motif in bioactive molecules **2 v** was obtained in 99 % yield with high enantioselectivity,^[1f]^ which demonstrates this strategy's potential in pharmaceutical synthesis. In addition, the absolute configuration of **2 v** was determined to be *R* by X‐ray crystallographic analysis.^[15]^

Next, we turned our attention to the impact of substituent groups in the 4‐position (Scheme 4). Ethyl‐substituted substrate **1 w** was hydrogenated to the corresponding product (**2 w**) with full conversion and moderate enantiomeric ratio (77:23). Surprisingly, sterically even more demanding substrates **1 x** and **1 y** gave the desired products with excellent yields and better enantiomeric excess compared to **1 w**.^[16]^ Interestingly, 3‐methyl‐substituted product **2 z** is the key motif of D_2_/5‐HT_2A_ receptor dual antagonist SIPI 6360,^[1e]^ which could be hydrogenated from the corresponding quinolone in moderate yield under our mild standard conditions.

**Scheme 4:**
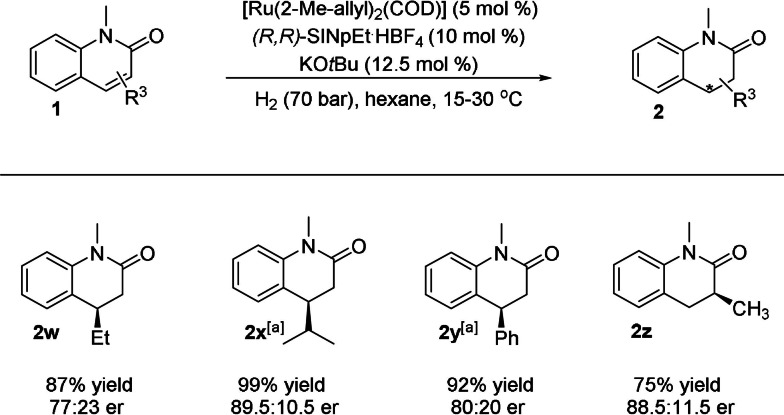
Substrate scope of 4‐substituted 2‐quinolones. General conditions: [Ru(COD)(2‐methylallyl)_2_] (0.01 mmol), KO*t*Bu (0.025 mmol), (*R*,*R*)‐SINpEt⋅HBF_4_ (0.02 mmol) were stirred at 70 °C in *n*‐hexane (0.33 mL) for 16 h, after which it was added to **1** (0.2 mmol) in Et_2_O (1 mL). The hydrogenation was performed at 15 °C under 70 bar H_2_ for 24 h. Yields of isolated products are given. Enantiomeric ratios were determined by HPLC on a chiral stationary phase. [a] Reaction run at 30 °C using 20 % [Ru] catalyst under 70 bar H_2_ for 48 h.

To demonstrate the utility of this reaction, the products were further manipulated. The dihydro‐2‐quinolone **2 b** could be further reduced to tetrahydroquinoline **3 b** using DIBAL‐H as reductant without loss in enantiomeric excess (Scheme 5).^[2a]^ More impressively, the dimethyl‐substituted product **2 r** was smoothly transformed to the saturated octahydroquinolone **4 r** using the Rh–CAAC/H_2_ catalyst system.[^3b^, ^17^]

**Scheme 5:**
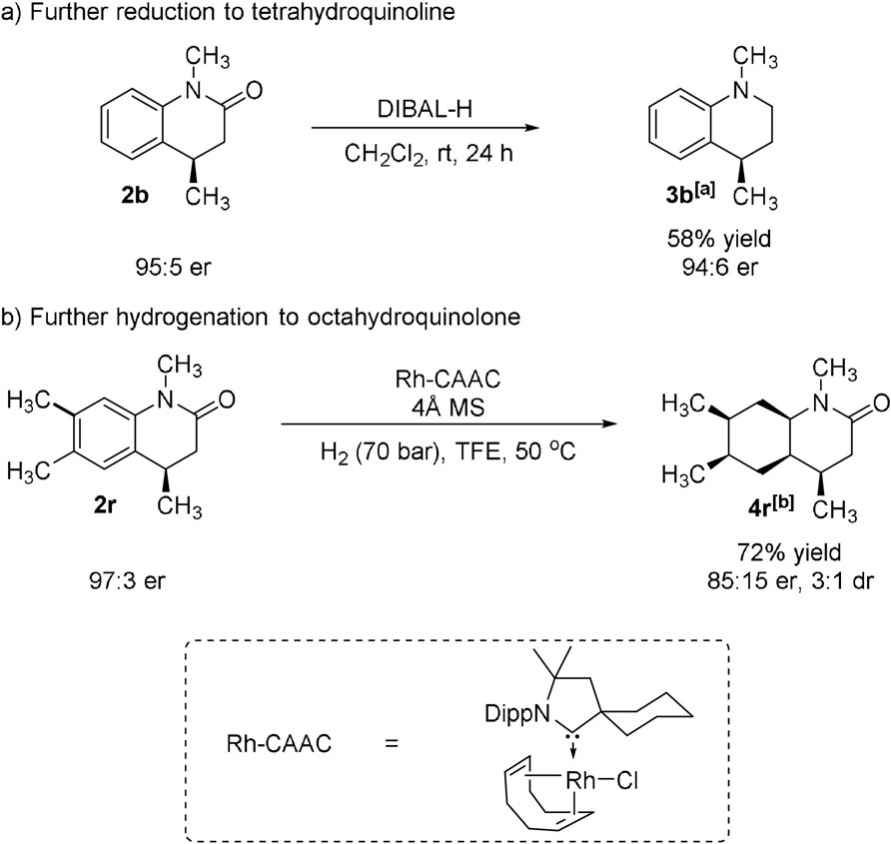
Transformations of dihydro‐2‐quinolones. [a] Enantiomeric ratio was determined by HPLC on a chiral stationary phase. [b] Enantiomeric ratio was determined by GC‐FID; d.r. value was determined by ^1^H NMR analysis.

In summary, the first ruthenium‐NHC‐catalyzed asymmetric hydrogenation of 2‐quinolones to simple 3,4‐dihydro‐2‐quinolones has been developed with high yields (up to 99 %) and moderate to excellent enantioselectivities (up to 98:2 e.r.). This method shows good functional group compatibility. Alkyl, methoxy, aryl, halogens, and trifluoromethyl were tolerated under the mild conditions. Additionally, 3,4‐dihydro‐2‐quinolone derivatives could be further reduced to other interesting chiral three‐dimensional motifs. We anticipate that this newly developed procedure would fulfill its potential in the synthesis of building blocks and pharmaceutical compounds.

## Conflict of interest

The authors declare no conflict of interest.

## Supporting information

As a service to our authors and readers, this journal provides supporting information supplied by the authors. Such materials are peer reviewed and may be re‐organized for online delivery, but are not copy‐edited or typeset. Technical support issues arising from supporting information (other than missing files) should be addressed to the authors.

Supporting Information
