# Supplementary material for: Ru‐NHC‐Catalyzed Asymmetric Hydrogenation of 2‐Quinolones to Chiral 3,4‐Dihydro‐2‐Quinolones
Source: Angew Chem Int Ed Engl. 2021 Sep 22;60(43):23193–6. doi: 10.1002/anie.202108503 (PMC8596914; doi:10.1002/anie.202108503)
Supplement: Supplementary file 1 — Supporting Information [file ANIE-60-23193-s001.pdf]

Angewandte Chemie
Eine Zeitschrift der Gesellschaft Deutscher Chemiker

Supporting Information

Ru-NHC-Catalyzed Asymmetric Hydrogenation of 2-Quinolones to Chiral 3,4-Dihydro-2-Quinolones

*Tianjiao Hu, Lukas Lückemeier, Constantin Daniliuc, and Frank Glorius**

anie_202108503_sm_miscellaneous_information.pdf

Supporting Information

Table of Contents

1. General information 2

2. Preparation of substrates 3

3. General procedure for the asymmetric hydrogenation of 2-quinolones 9

4. Transformations 34

5. X-Ray analysis 37

6. Proposed enantiodiscrimination model 38

7. References 39

8. Copies of NMR spectra 40

1. General information

All reagents were obtained commercially unless otherwise noted. Unless otherwise noted, all reactions were carried out under an atmosphere of air. The employed solvents were either dried by distillation over standard drying agents and stored under argon over molecular sieves or directly used from a solvent purification system (HPLC grade, dried via an alumina/molecular sieves column under positive argon pressure; n-hexane, dichloromethane, toluene, DMF, diethyl ether, MeCN, THF). Catalytic hydrogenation reactions were prepared under argon and carried out in Berghof High Pressure Reactors using hydrogen gas. Reaction temperatures are reported as the temperature of the bath surrounding the vessel unless otherwise stated.

Analytical thin layer chromatography (TLC) was performed on silica gel 60 F254 aluminum plates (Merck). TLC plates were visualized by exposure to short wave ultraviolet light (254nm, 366nm). Flash chromatography was performed on Acros Organics silica gel (35-70 mesh) under a positive pressure of argon, eluting with the specified solvent system.

GC-MS spectra were recorded on an Agilent Technologies 7890A GC-system with an Agilent 5975C VL MSD or an Agilent 5975 inert Mass Selective Detector (EI) and a HP-5MS column (0.25 mm x 30 m, film: 0.25 μm). Enantiomeric ratios of isolated products were determined with an Agilent Technologies 7890B GC-system and a Supelco b-Dex column or on an Astec Chiraldex G-TA column. Alternatively, enantiomeric ratios were determined using an Agilent Technologies 1200 Series HPLC with Daicel Chemical Industries LTD Chiralpak AD-H, AS-H or Chiralcel OD-H, OJ-H columns (0.46 cm x 25 cm). The signals were detected by UV-absorption spectroscopy (at 210, 230 or 254 nm). ESI mass spectra were recorded on a Bruker Daltonics MicroTof spectrometer. APCI mass spectra were recorded on a Thermo Fisher Scientific Orbitrap LTQ XL. ^{1}H, ^{13}C, ^{19}F spectra were recorded on a Bruker Avance II300 or Avance II400, AgilentDD2 500 or AgilentDD2 600 in the indicated solvents. Chemical shifts (δ) are given in ppm relative to TMS. The residual solvent signals were used as references and the chemical shifts converted to the TMS scale ($CDCl_3$: δ_H = 7.26 ppm, δ_C = 77.16 ppm). Multiplicities are reported using the following abbreviations: s = singlet, d = doublet, t = triplet, q = quartet, quintet = quint, heptet = hept, m = multiplet, br = broad resonance.

2. Preparation of substrates

(a) Synthesis of **1a, 1b, 1d-i, 1l-s, 1v-y**[1, 2, 3]

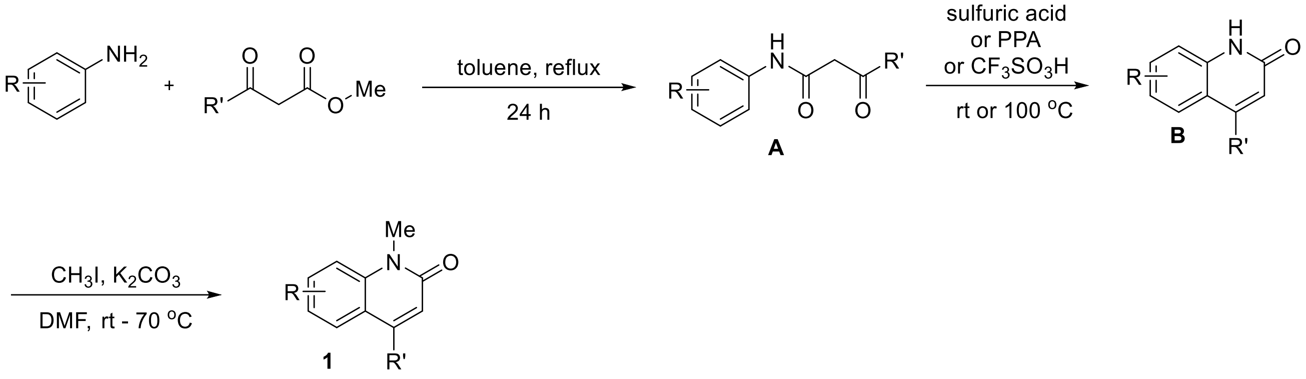

Aniline (0.4 mmol) and methyl acetoacetate (0.6 mmol, 1.5 equiv.) were added in to toluene (4 ml). The mixture was refluxed with stirring for 24 hours. Then solvent was removed under vacuum. The residue was purified by column chromatography on silica gel (PE: EA = 2:1) to afford product **A**. Product **A** was added in 98% sulfuric acid or PPA or CF_3SO_3H. The reaction mixture was stirred 3 hours at indicated temperature. After cooling down to room temperature, ice was added. The precipitate was filtered. The crude product **B** (100 mg), K_2CO_3 (170 mg, 2 equiv.) and CH_3I (0.2 mL) were stirred in DMF. The suspension was kept at room temperature or 70 ℃ until no starting material **B** was observed. To the mixture was added water, then the mixture was extracted with EtOAc, the combined EtOAc solution was washed with brine, dried over anhydrous $MgSO_4$. After removing the solvent, the residue was purified by flash chromatography on silica gel to afford **1**.

(b) Synthesis of **1c**

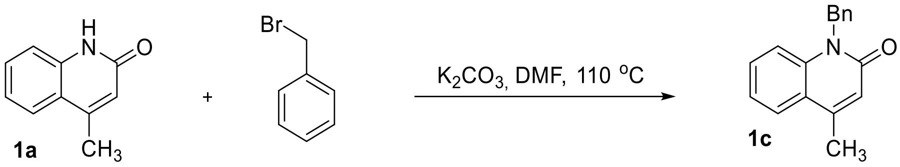

1a (318 mg, 2 mmol), K_2CO_3 (414 mg, 3 mmol, 1.5 equiv.), benzyl bromide (0.4 mL,1.5 equiv.) were added in DMF (2 mL) in the Schlenk tube under argon atmosphere. The mixture was heated at 110 ℃ for 24 hours. To the mixture was added water, then the mixture was extracted with EtOAc, the combined EtOAc solution was washed with brine, dried over anhydrous $MgSO_4$. After removing the solvent, the residue was purified by flash chromatography on silica gel to afford **1c** (195 mg).

(c) Synthesis of **1j, 1k, 1t, 1u**

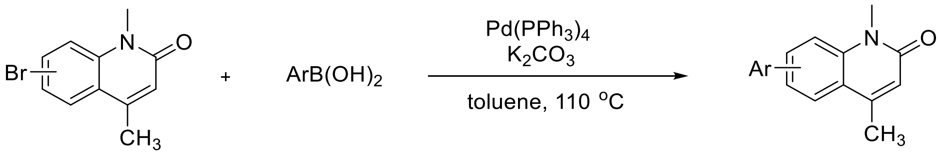

Bromo-substituted 2 –quinolones (0.2 mmol), arylboronic acid (48.8 mg, 0.4 mmol, 2 equiv.), $Pd(PPh_3)_4$ (11.6 mg, 5 %), K_2CO_3 (55.2 mg, 0.4 mmol, 2 equiv.) were dissolved in toluene (1 mL) under argon

atmosphere in Schlenk tube. The reaction mixture was stirred at 110 ℃ for 24 hours. To the mixture was added water, then the mixture was extracted with EtOAc, the combined EtOAc solution was washed with brine, dried over anhydrous $MgSO_4$. After removing the solvent, the residue was purified by flash chromatography on silica gel to afford **1j, 1k, 1t, 1u**.

(d) Synthesis of **1z**[4]

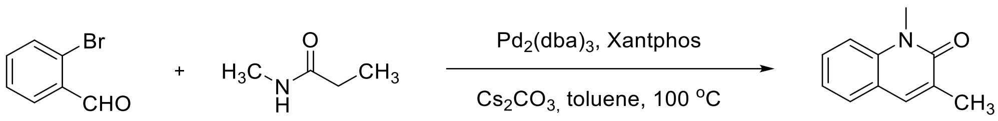

1z was synthesized according to a literature procedure. A pressure tube was charged with 2-Bromobenzaldehyde (640 µL, 5.50 mmol, 1.0 equiv.), *N*-methylpropionamide (618 µL, 6.60 mmol, 1.2 equiv.), $Pd_2(dba)_3$ (50 mg, 55 µmol, 1 mol%), Xantphos (95 mg, 0.17 mmol, 3 mol%) and Cs_2CO_3 (2.51 g, 7.7 mmol, 1.4 equiv.) and dry toluene (20 mL) was added. The mixture was degassed (3×) and stirred at 100 °C for 16 h. The mixture was filtered over Celite® with CH_2Cl_2 and purified by column chromatography (pentane: EtOAc = 2:1, later 3:2) to afford the product as yellow solid (329 mg, 1.90 mmol, 35%).

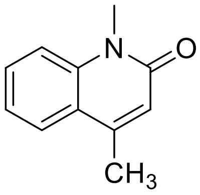

1,4-Dimethylquinolin-2(1*H*)-one (**1b**)

^{1}H NMR (400 MHz, $CDCl_3$) δ 7.65 (dd, *J* = 8.0, 1.5 Hz, 1H), 7.52 (ddd, *J* = 8.6, 7.1, 1.5 Hz, 1H), 7.31 (dd, *J* = 8.6, 1.1 Hz, 1H), 7.21 (ddd, *J* = 8.1, 7.1, 1.1 Hz, 1H), 6.54 (s, 1H), 3.65 (s, 3H), 2.41 (d, *J* = 1.3 Hz, 3H); **^{13}C NMR** (101 MHz, $CDCl_3$) δ 162.1, 146.4, 139.7, 130.5, 125.2, 121.1, 121.4, 121.0, 114.4, 29.2, 19.0 ; **ESI-MS**: calculated $[C_{11}H_{11}NONa]^+$:196.0733, found:196.0731.

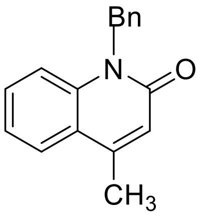

1-Benzyl-4-methylquinolin-2(1*H*)-one (**1c**)

^{1}H NMR (400 MHz, $CDCl_3$) δ 7.60 (dd, *J* = 7.9, 1.6 Hz, 1H), 7.31 (ddd, *J* = 8.6, 7.1, 1.5 Hz, 1H), 7.21 – 7.15 (m, 3H), 7.14 – 7.06 (m, 4H), 6.61 (d, *J* = 1.3 Hz, 1H), 5.46 (s, 2H), 2.40 (d, *J* = 1.3 Hz, 3H); **^{13}C NMR** (101 MHz, $CDCl_3$) δ 162.3, 147.1, 139.2, 136.6, 130.5, 128.8, 127.2, 126.6, 125.3, 122.1, 121.7, 121.0, 115.4, 45.7, 19.2; **ESI-MS**: calculated $[C_{17}H_{17}NONa]^+$:274.1202, found:274.1199.

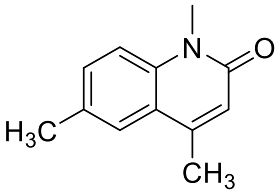

1,4,6-Trimethylquinolin-2(1*H*)-one (**1d**)

^{1}H NMR (400 MHz, $CDCl_3$) δ 7.37 (d, *J* = 2.0 Hz, 1H), 7.29 (dd, *J* = 8.7, 2.0 Hz, 1H), 7.15 (d, *J* = 8.6 Hz, 1H), 6.48 (d, *J* = 1.5 Hz, 1H), 3.58 (s, 3H), 2.37 (s, 3H), 2.35 (d, *J* = 1.3 Hz, 3H); **^{13}C NMR** (101 MHz, $CDCl_3$) δ 161.8, 146.0, 137.6, 131.5, 131.2, 125.0, 121.2, 120.9, 114.2, 29.1, 20.8, 18.9; **ESI-MS**: calculated $[C_{12}H_{13}NONa]^+$:210.0889, found:210.0887.

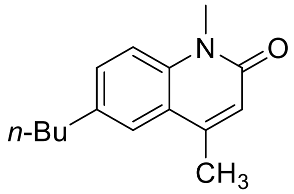

6-Butyl-1,4-dimethylquinolin-2(1*H*)-one (**1e**)

^{1}H NMR (400 MHz, $CDCl_3$) δ 7.53 (d, *J* = 2.0 Hz, 1H), 7.46 (dd, *J* = 8.6, 2.0 Hz, 1H), 7.36 (d, *J* = 8.6 Hz, 1H), 6.83 (s, 1H), 3.75 (s, 3H), 2.78 – 2.68 (m, 2H), 2.51 (d, *J* = 1.1 Hz, 3H), 1.71 – 1.59 (m, 2H), 1.39 (dq, *J* = 14.7, 7.4 Hz, 2H), 0.95 (t, *J* = 7.4 Hz, 3H); **^{13}C NMR** (101 MHz, $CDCl_3$) δ 162.2, 147.7, 137.7, 137.5, 131.6, 124.8, 121.9, 120.1, 114.8, 35.2, 33.9, 29.9, 22.5, 19.3, 14.1; **ESI-MS**: calculated $[C_{15}H_{19}NONa]^+$:252.1359, found:252.1359.

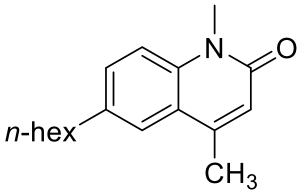

6-Hexyl-1,4-dimethylquinolin-2(1*H*)-one (**1f**)

^{1}H NMR (400 MHz, $CDCl_3$) δ 7.46 (d, *J* = 2.0 Hz, 1H), 7.39 (dd, *J* = 8.6, 2.0 Hz, 1H), 7.31 – 7.24 (m, 1H), 6.57 (s, 1H), 3.68 (s, 3H), 2.72 – 2.64 (m, 2H), 2.44 (s, 3H), 1.68 – 1.63 (m, 2H), 1.39 – 1.24 (m, 6H), 0.88 (td, *J* = 5.7, 2.4 Hz, 3H); **^{13}C NMR** (101 MHz, $CDCl_3$) δ 162.1, 146.3, 138.0, 136.6, 131.0, 124.6, 121.4, 121.1, 114.4, 35.4, 31.8, 31.8, 29.3, 29.0, 22.7, 19.1, 14.2; **ESI-MS**: calculated $[C_{17}H_{23}NONa]^+$:280.1672, found: 280.1671.

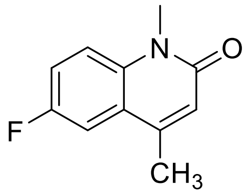

6-Fluoro-1,4-dimethylquinolin-2(1*H*)-one (**1g**)

^{1}H NMR (400 MHz, $CDCl_3$) δ 7.36 – 7.08 (m, 3H), 6.62 (s, 1H), 3.64 (s, 3H), 2.36 (d, *J* = 1.2 Hz, 3H); **^{13}C NMR** (101 MHz, $CDCl_3$) δ 161.9, 158.1 (d, *J* = 242.2 Hz), 145.9 (d, *J* = 3.1 Hz), 136.4, 122.7 (d, *J* = 7.8 Hz), 122.2, 118.3 (d, *J* = 23.7 Hz), 116.1 (d, *J* = 8.2 Hz), 110.9 (d, *J* = 23.0 Hz), 29.8, 19.1; **^{19}F NMR** (376 MHz, CDCl3) δ -120.6; **ESI-MS**: calculated $[C_{11}H_{10}NOFNa]^+$:214.0639, found:214.0637.

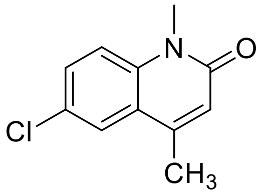

6-Chloro-1,4-dimethylquinolin-2(1*H*)-one (**1h**)

^{1}H NMR (400 MHz, $CDCl_3$) δ 7.60 (d, *J* = 2.4 Hz, 1H), 7.46 (dd, *J* = 9.0, 2.4 Hz, 1H), 7.27 – 7.18 (m, 1H), 6.59 (d, *J* = 1.5 Hz, 1H), 3.63 (s, 3H), 2.38 (d, *J* = 1.2 Hz, 3H); **^{13}C NMR** (101 MHz, $CDCl_3$) δ 161.8, 145.6, 138.5, 130.5, 127.8, 124.9, 122.8, 122.2, 116.0, 29.6, 19.0; **ESI-MS**: calculated $[C_{11}H_{10}NOClNa]^+$:230.0343, found:230.0341.

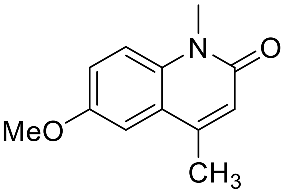

6-Methoxy-1,4-dimethylquinolin-2(1*H*)-one (1i)

^{1}H NMR (400 MHz, $CDCl_3$) δ 7.30 (d, *J* = 9.2 Hz, 1H), 7.18 (dd, *J* = 9.2, 2.8 Hz, 1H), 7.12 (d, *J* = 2.9 Hz, 1H), 6.60 (s, 1H), 3.88 (s, 3H), 3.69 (s, 3H), 2.43 (s, 3H); **^{13}C NMR** (101 MHz, $CDCl_3$) δ 161.8, 154.7, 145.7, 134.5, 122.4, 121.9, 118.3, 115.7, 108.1, 55.9, 29.5, 19.2; **ESI-MS**: calc. for $C_{12}H_{13}NO_2Na$ $[M+Na]^+$ 226.0839, found 226.0834.

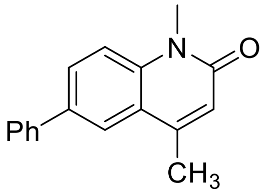

1,4-Dimethyl-6-phenylquinolin-2(1*H*)-one (**1j**)

^{1}H NMR (400 MHz, $CDCl_3$) δ 7.89 (d, *J* = 2.1 Hz, 1H), 7.81 (dd, *J* = 8.7, 2.1 Hz, 1H), 7.67 – 7.59 (m, 2H), 7.53 – 7.43 (m, 3H), 7.43 – 7.34 (m, 1H), 6.67 (d, *J* = 1.4 Hz, 1H), 3.75 (s, 3H), 2.53 (d, *J* = 1.2 Hz, 3H); **^{13}C NMR** (101 MHz, $CDCl_3$) δ 162.2, 146.7, 140.3, 139.2, 135.2, 129.6, 129.1, 127.6, 127.2, 123.6, 121.9, 121.6, 115.1, 29.5, 19.2; **ESI-MS**: calculated $[C_{17}H_{15}NONa]^+$:272.1046, found:272.1045

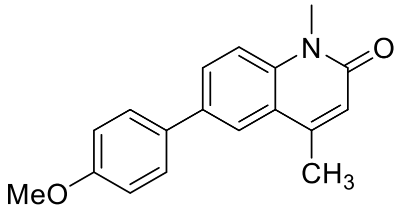

6-(4-Methoxyphenyl)-1,4-dimethylquinolin-2(1*H*)-one (**1k**)

^{1}H NMR (400 MHz, $CDCl_3$) δ 7.30 (d, *J* = 9.2 Hz, 1H), 7.18 (dd, *J* = 9.2, 2.8 Hz, 1H), 7.12 (d, *J* = 2.9 Hz, 1H), 6.60 (s, 1H), 3.88 (s, 3H), 3.69 (s, 3H), 2.43 (s, 3H); **^{13}C NMR** (101 MHz, $CDCl_3$) δ 161.8, 154.7, 145.7, 134.5, 122.4, 121.9, 118.3, 115.7, 108.1, 55.9, 29.5, 19.2; **ESI-MS**: calculated $[C_{18}H_{19}NO_2Na]^+$: 304.1308, found: 304.1306.

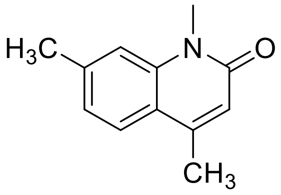

1,4,7-Trimethylquinolin-2(1*H*)-one (**1l**)

^{1}H NMR (400 MHz, $CDCl_3$) δ 7.56 (d, *J* = 8.1 Hz, 1H), 7.15 (s, 1H), 7.06 (dd, *J* = 8.2, 1.6 Hz, 1H), 6.52 (d, *J* = 1.5 Hz, 1H), 3.67 (s, 3H), 2.49 (s, 3H), 2.42 (d, *J* = 1.2 Hz, 3H); **^{13}C NMR** (101 MHz, $CDCl_3$) δ 162.4, 146.4, 141.1, 140.0, 125.1, 123.3, 120.1, 119.3, 114.8, 29.3, 22.2, 19.0; **ESI-MS**: calculated $[C_{12}H_{13}NONa]^+$:210.0889, found:210.0886.

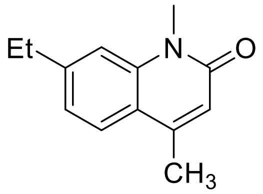

7-Ethyl-1,4-dimethylquinolin-2(1*H*)-one (**1m**)

^{1}H NMR (400 MHz, $CDCl_3$) δ 7.61 (d, *J* = 8.1 Hz, 1H), 7.18 (d, *J* = 1.5 Hz, 1H), 7.12 (dd, *J* = 8.1, 1.6 Hz, 1H), 6.57 (q, *J* = 1.2 Hz, 1H), 3.71 (s, 3H), 2.79 (q, *J* = 7.6 Hz, 2H), 2.44 (d, *J* = 1.2 Hz, 3H), 1.31 (t, *J* = 7.6 Hz, 3H); **^{13}C NMR** (101 MHz, $CDCl_3$) δ 162.4, 147.6, 146.7, 140.0, 125.3, 122.3, 120.0, 119.6, 113.7, 29.5, 29.4, 19.1, 15.7; **ESI-MS**: calculated $[C_{13}H_{15}NONa]^+$:224.1046, found:224.1041.

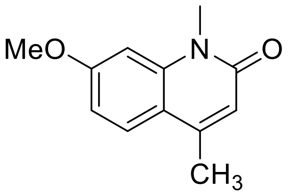

7-Methoxy-1,4-dimethylquinolin-2(1*H*)-one (1n)

^{1}H-NMR (400 MHz, DMSO-d_6) δ 7.69 (d, *J* = 8.7 Hz, 1H), 6.94 – 6.87 (m, 2H), 6.34 (d, *J* = 1.3 Hz, 1H), 3.90 (s, 3H), 3.57 (s, 3H), 2.38 (s, 3H); **^{13}C-NMR** (101 MHz, DMSO-d_6) δ 161.3, 161.2, 146.4, 141.2, 126.8, 117.3, 114.6, 109.4, 98.9, 55.6, 28.8, 18.4; **ESI-MS**: calc. for $C_{12}H_{13}NO_2Na$ $[M+Na]^+$ 226.0839, found 226.0834.

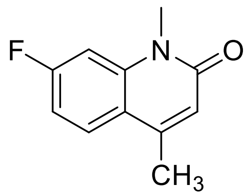

7-Fluoro-1,4-dimethylquinolin-2(1*H*)-one (**1o**)

^{1}H NMR (400 MHz, $CDCl_3$) δ 7.68 (dd, *J* = 8.9, 6.2 Hz, 1H), 7.05 (dd, J = 11.0, 2.4 Hz, 1H), 6.98 (ddd, *J* = 8.8, 7.9, 2.4 Hz, 1H), 6.56 (d, *J* = 1.3 Hz, 1H), 3.66 (s, 3H), 2.45 (d, *J* = 1.1 Hz, 3H); **^{13}C NMR** (101 MHz, $CDCl_3$) δ 164.0 (d, *J* = 249.6 Hz), 162.3, 146.2, 141.5 (d, *J* = 11.0 Hz), 127.2 (d, *J* = 10.3 Hz), 120.0 (d, *J* = 2.5 Hz), 118.1 (d, *J* = 2.2 Hz), 109.8 (d, *J* = 22.7 Hz), 101.4 (d, *J* = 26.7 Hz), 29.5, 19.1; **^{19}F NMR** (377 MHz, $CDCl_3$) δ -107.67; **ESI-MS**: calculated $[C_{11}H_{10}NOFNa]^+$:214.0639, found:214.0635.

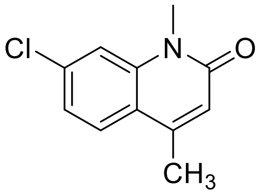

7-Chloro-1,4-dimethylquinolin-2(1*H*)-one (**1p**)

^{1}H NMR (400 MHz, $CDCl_3$) δ 7.54 (d, *J* = 8.5 Hz, 1H), 7.29 (d, *J* = 2.0 Hz, 1H), 7.15 (dd, *J* = 8.5, 2.0 Hz, 1H), 6.52 (q, *J* = 1.2 Hz, 1H), 3.60 (s, 3H), 2.37 (d, *J* = 1.2 Hz, 3H); **^{13}C NMR** (101 MHz, $CDCl_3$) δ 162.1, 146.1, 140.8, 136.7, 126.5, 122.4, 121.3, 120.1, 114.6, 29.5, 19.1; **ESI-MS**: calculated $[C_{11}H_{10}NOClNa]^+$:230.0343, found:230.0340.

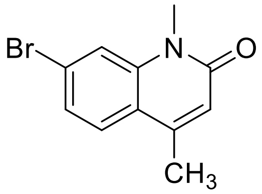

7-Bromo-1,4-dimethylquinolin-2(1*H*)-one (**1q**)

^{1}H NMR (400 MHz, $CDCl_3$) δ 7.54 – 7.46 (m, 2H), 7.33 (dd, *J* = 8.5, 1.8 Hz, 1H), 6.57 (q, *J* = 1.3 Hz, 1H), 3.64 (s, 3H), 2.41 (d, *J* = 1.2 Hz, 3H); **^{13}C NMR** (101 MHz, $CDCl_3$) δ 161.9, 146.1, 140.9, 126.6, 125.1, 124.9, 121.4, 120.3, 117.5, 29.4, 19.0; **ESI-MS**: calculated $[C_{11}H_{10}NOBrNa]^+$:273.9838, found:273.9837.

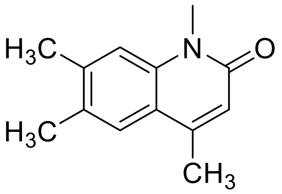

1,4,6,7-Tetramethylquinolin-2(1*H*)-one (**1r**)

^{1}H NMR (400 MHz, $CDCl_3$) δ 7.42 (s, 1H), 7.14 (s, 1H), 6.53 (d, *J* = 1.4 Hz, 1H), 3.68 (s, 3H), 2.42 (d, *J* = 1.2 Hz, 3H), 2.40 (s, 3H), 2.34 (s, 3H); **^{13}C NMR** (101 MHz, $CDCl_3$) δ 162.3, 146.3, 140.2, 138.2, 130.7, 125.7, 120.1, 119.6, 115.4, 29.3, 20.7, 19.4, 19.1; **ESI-MS**: calculated $[C_{13}H_{15}NONa]^+$:224.1046, found:224.1042.

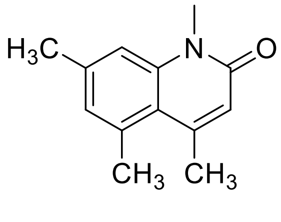

1,4,5,7-Tetramethylquinolin-2(1*H*)-one (**1s**)

^{1}H NMR (400 MHz, $CDCl_3$) δ 7.06 (d, *J* = 1.7 Hz, 1H), 6.86 (d, *J* = 1.7 Hz, 1H), 6.48 (s, 1H), 3.68 (s, 3H), 2.72 (s, 3H), 2.62 (d, *J* = 1.2 Hz, 3H), 2.42 (s, 3H); **^{13}C NMR** (101 MHz, $CDCl_3$) δ 161.8, 148.1, 141.6, 140.1, 137.1, 128.1, 121.9, 119.0, 113.8, 30.1, 25.9, 25.4, 21.8; **ESI-MS**: calculated $[C_{13}H_{15}NONa]^+$:224.1046, found:224.1041.

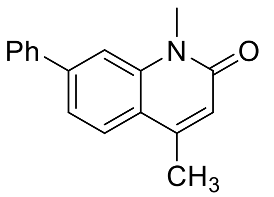

1,4-Dimethyl-7-phenylquinolin-2(1*H*)-one (**1t**)

^{1}H NMR (400 MHz, $CDCl_3$) δ 7.76 (d, *J* = 8.3 Hz, 1H), 7.70 – 7.62 (m, 2H), 7.57 – 7.46 (m, 4H), 7.46 – 7.36 (m, 1H), 6.61 (d, *J* = 1.4 Hz, 1H), 3.76 (s, 3H), 2.49 (d, *J* = 1.2 Hz, 3H); **^{13}C NMR** (101 MHz, $CDCl_3$) δ 162.4, 146.4, 143.7, 140.5, 140.3, 129.1, 128.3, 127.6, 125.8, 121.3, 121.0, 120.6, 113.0, 29.4, 19.1; **ESI-MS**: calculated $[C_{17}H_{15}NONa]^+$:272.1046, found:272.1042.

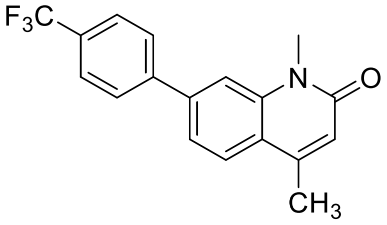

1,4-Dimethyl-7-(4-(trifluoromethyl)phenyl)quinolin-2(1*H*)-one (**1u**)

^{1}H NMR (400 MHz, $CDCl_3$) δ 7.82 – 7.70 (m, 5H), 7.52 (d, *J* = 1.7 Hz, 1H), 7.47 (dd, *J* = 8.2, 1.7 Hz, 1H), 6.63 (d, *J* = 1.3 Hz, 1H), 3.76 (s, 3H), 2.49 (d, *J* = 1.3 Hz, 3H); **^{13}C NMR** (101 MHz, $CDCl_3$) δ 162.3, 146.2, 144.0 (d, *J* = 1.5 Hz), 142.1, 140.4, 130.3 (q, *J* = 32.6 Hz), 127.9,126.1 (q, *J* = 11.3 Hz),126.1, 124.3 (q, *J* = 272.1 Hz), 121.6, 121.3, 121.2, 113.2, 29.4, 19.1; **^{19}F NMR** (376 MHz, $CDCl_3$) δ -62.52; **ESI-MS**: calculated $[C_{18}H_{14}NONa]^+$:340.0920, found:340.0917.

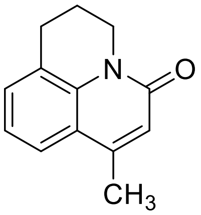

7-Methyl-2,3-dihydro-1H,5H-pyrido[3,2,1-ij]quinolin-5-one (**1v**)

^{1}H NMR (400 MHz, $CDCl_3$) δ 7.53 (dd, *J* = 8.0, 1.5 Hz, 1H), 7.34 – 7.24 (m, 1H), 7.18 – 7.10 (m, 1H), 6.59 (d, *J* = 1.3 Hz, 1H), 4.22 – 4.14 (m, 2H), 2.98 (t, *J* = 6.2 Hz, 2H), 2.45 (d, *J* = 1.2 Hz, 3H), 2.14 – 2.04 (m, 2H); **^{13}C NMR** (101 MHz, $CDCl_3$) δ 161.8, 146.5, 136.5, 130.0, 125.2, 123.2, 121.6, 121.4, 120.8, 42.3, 28.1, 20.8, 19.3; **ESI-MS**: calculated $[C_{13}H_{13}NONa]^+$:222.0890, found:222.0884.

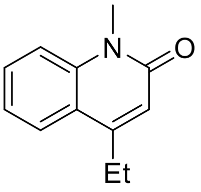

4-Ethyl-1-methylquinolin-2(1*H*)-one (**1w**)

^{1}H NMR (400 MHz, $CDCl_3$) δ 7.77 (dd, *J* = 8.0, 1.5 Hz, 1H), 7.57 (ddd, *J* = 8.6, 7.1, 1.5 Hz, 1H), 7.39 (dd, *J* = 8.5, 1.1 Hz, 1H), 7.30 – 7.22 (m, 1H), 6.64 (s, 1H), 3.72 (s, 3H), 2.87 (qd, *J* = 7.5, 1.1 Hz, 2H), 1.34 (t, *J* = 7.4 Hz, 3H); **^{13}C NMR** (101 MHz, $CDCl_3$) δ 162.5, 151.8, 140.1, 130.5, 124.9, 122.1, 120.9, 119.2, 114.8, 29.4, 25.0, 13.0; **ESI-MS**: calculated $[C_{12}H_{13}NONa]^+$:210.0889, found:210.0886.

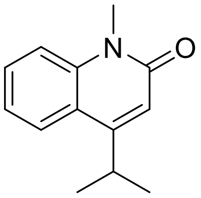

4-Isopropyl-1-methylquinolin-2(1*H*)-one (**1x**)

^{1}H NMR (400 MHz, $CDCl_3$) δ 7.81 (dd, *J* = 8.1, 1.5 Hz, 1H), 7.55 (ddd, *J* = 8.6, 7.1, 1.5 Hz, 1H), 7.39 (dd, *J* = 8.6, 1.1 Hz, 1H), 7.30 – 7.21 (m, 1H), 6.66 (d, *J* = 0.8 Hz, 1H), 3.71 (s, 3H), 3.47 – 3.32 (m, 1H), 1.32 (d, *J* = 6.8 Hz, 6H); **^{13}C NMR** (101 MHz, $CDCl_3$) δ 162.7, 156.2, 140.2, 130.3, 124.6, 122.0, 120.3, 117.1, 114.9, 29.4, 28.4, 22.4; **ESI-MS**: calculated $[C_{13}H_{15}NONa]^+$:224.1046, found:224.1041.

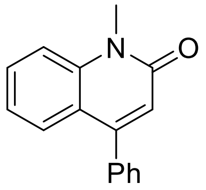

1-Methyl-4-phenylquinolin-2(1*H*)-one (**1y**)

^{1}H NMR (400 MHz, $CDCl_3$) δ 7.63 – 7.53 (m, 2H), 7.51 – 7.38 (m, 6H), 7.17 (ddd, *J* = 8.1, 7.1, 1.1 Hz, 1H), 6.70 (s, 1H), 3.79 (s, 3H); **^{13}C NMR** (101 MHz, $CDCl_3$) δ 162.1, 151.1, 140.4, 137.2, 130.8, 129.1, 128.8, 128.7, 127.9, 122.1, 121.3, 120.7, 114.6, 29.6; **ESI-MS**: calculated $[C_{16}H_{13}NONa]^+$:258.0890, found:258.0886.

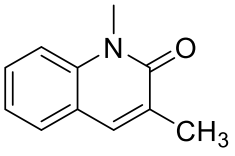

1,3-Dimethylquinolin-2(1*H*)-one (**1z**)

^{1}H NMR (400 MHz, $CDCl_3$) δ 7.48 – 7.37 (m, 3H), 7.24 (dd, *J* = 8.5, 1.0 Hz, 1H), 7.15 (ddd, *J* = 8.0, 7.2, 1.0 Hz, 1H), 3.66 (s, 3H), 2.20 (d, *J* = 1.5 Hz, 3H); **^{13}C NMR** (101 MHz, $CDCl_3$) δ 162.8, 139.0, 135.6, 129.9, 129.2, 127.7, 121.9, 120.7, 113.8, 29.6, 17.7; **ESI-MS**: calculated $[C_{11}H_{11}NONa]^+$:196.0733, found:196.0730.

3. General procedure for the asymmetric hydrogenation of 2-quinolones

In a glove box, to a flame-dried screw-capped tube equipped with a magnetic stir bar was added [Ru(COD)(2-methylallyl)$_2$] (0.3 mmol; cod=cyclooctadiene), NHC·HX (0.6 mmol), and dry KO*t*Bu (0.75 mmol). The mixture was suspended in n-hexane (10 mL) and stirred at 70 °C for 16 h. To a 4 mL glass vial (screw-cap with septum) equipped with a stir bar the substrate **1** (0.2 mmol, 1.00 equiv.), Under argon atmosphere, n-hexane (1 mL) and the preformed Ru((*R,R*)-SINpEt)$_2$ catalyst as stock suspension (0.33 mL, 5 mol%) in n-hexane were added. The glass vial was placed in a 150 mL stainless steel autoclave. The autoclave was pressurized and depressurized with hydrogen gas three times before the indicated pressure was set. The reaction mixture was stirred at 15-30 °C for 24-48 h. After the autoclave was carefully depressurized, the mixture was purified by flash column chromatography on silica gel eluting *n*-pentane: ethyl acetate = (5:1) to afford the desired product **2**.

Preparation of racemic products:

For substrates **1a-p**, **1r-z**: to a dry glass vial equipped with a magnetic stir bar was added Pd/C (30 mg), 2-quinolones **1** (10 mg) and CH_3OH (1 mL). The glass vial was placed in a 150 mL stainless steel autoclave. The autoclave was pressurized and depressurized with hydrogen gas three times before a pressure of 20 bar H_2 was set. The reaction mixture was stirred at 40 ^{o}C for 24 h. After the autoclave was carefully depressurized, the mixture was purified by flash column chromatography on silica gel (*n*-pentane/ethyl acetate = 5:1) to afford the desired racemic product **2**.

For substrates **1q**: same as asymmetric hydrogenation using the mixture of Ru((*R,R*)-SINpEt)$_2$ catalyst and Ru((*S*,*S*)-SINpEt)$_2$ catalyst.

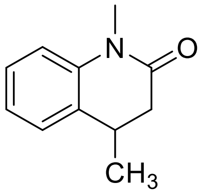

1,4-Dimethyl-3,4-dihydroquinolin-2(1*H*)-one (**2b**)

^{1}H NMR (400 MHz, $CDCl_3$) δ 7.24 (td, *J* = 7.7, 1.6 Hz, 1H), 7.18 (dd, *J* = 7.0, 1.4 Hz, 1H), 7.03 (td, *J* = 7.5, 1.2 Hz, 1H), 6.97 (dd, *J* = 8.1, 1.1 Hz, 1H), 3.35 (s, 3H), 3.03 (td, *J* = 7.3, 5.5 Hz, 1H), 2.71 (dd, *J* = 15.8, 5.5 Hz, 1H), 2.43 (dd, *J* = 15.8, 7.6 Hz, 1H),

1.26 (d, *J* = 7.0 Hz, 3H); ^{13}**C NMR** (101 MHz, $CDCl_3$) δ 170.0, 139.9, 131.2, 127.5, 126.3, 123.1, 114.9, 39.2, 30.4, 29.5, 19.4; **ESI-MS**: calculated $[C_{11}H_{14}NO]^+$:176.1070, found:176.1066; **HPLC** DAICEL CHIRALCEL OD-H, *n*-hexane/2-propanol = 95/5, flow rate = 1.0 mL/min, λ = 254 nm, retention time: 12.7 min (major), 14.3 min (minor).

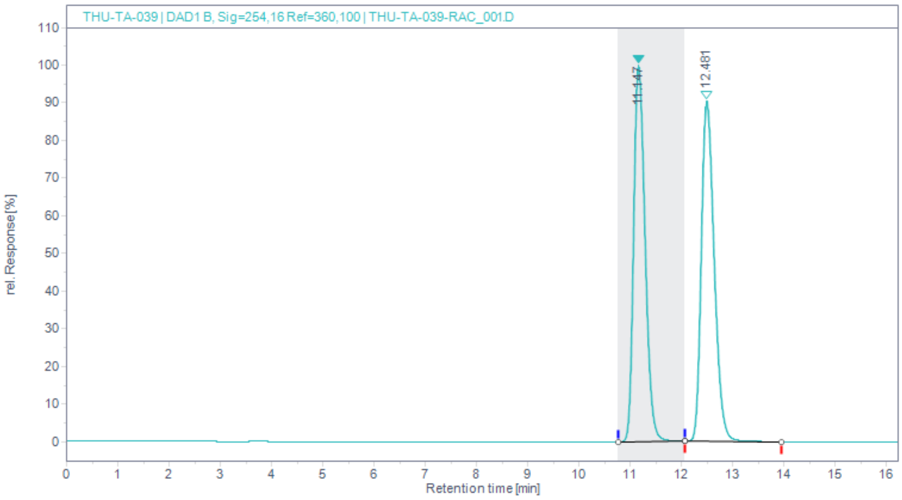

#	Name	Signal description	RT (min)	Area (mAU·s)	Area%	Height (mAU)	Height%	Amount	Concentration	Start time (min)	End time (min)
1		DAD1 B, Sig=254,16 Ref=360,100	11.147	23159.269	49.632	1491.170	52.54			10.753	12.076
2		DAD1 B, Sig=254,16 Ref=360,100	12.481	23502.245	50.368	1346.850	47.46			12.076	13.956

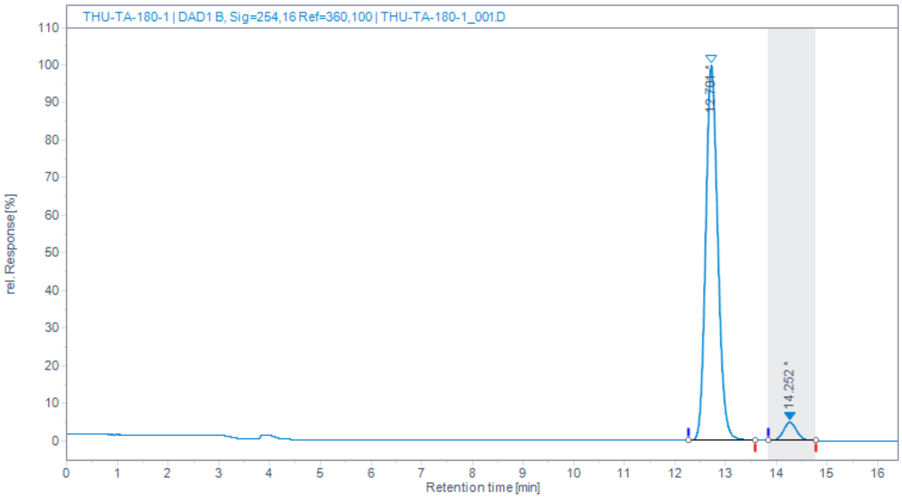

#	Name	Signal description	RT (min)	Area (mAU·s)	Area%	Height (mAU)	Height%	Amount	Concentration	Start time (min)	End time (min)
1		DAD1 B, Sig=254,16 Ref=360,100	12.701	6379.933	94.998	387.681	95.39			12.264	13.567
2		DAD1 B, Sig=254,16 Ref=360,100	14.252	335.930	5.002	18.740	4.61			13.825	14.765

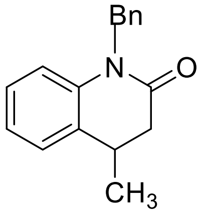

1-Benzyl-4-methyl-3,4-dihydroquinolin-2(1*H*)-one (2c)

^{1}**H NMR** (400 MHz, $CDCl_3$) δ 7.35 – 7.26 (m, 2H), 7.26 – 7.17 (m, 4H), 7.11 (td, *J* = 7.8, 1.7 Hz, 1H), 7.01 (td, *J* = 7.4, 1.2 Hz, 1H), 6.90 (dd, *J* = 8.1, 1.2 Hz, 1H), 5.27 – 5.13 (m, 2H), 3.13 (td, *J* = 7.2, 5.5 Hz, 1H), 2.88 (dd, *J* = 15.6, 5.4 Hz, 1H), 2.61 (dd, *J* = 15.7, 7.2 Hz, 1H), 1.33 (d, *J* = 7.0 Hz, 3H; ^{13}**C NMR** (101 MHz, $CDCl_3$) δ 170.2,

139.1, 137.2, 131.3, 128.9, 127.5, 127.2, 126.6, 126.6, 123.4, 115.9, 46.2, 39.3, 30.7, 19.6; **ESI-MS**: calculated $[C_{17}H_{17}NONa]^+$:274.1202, found:274.1199; **HPLC** DAICEL CHIRALCEL AD-H, *n*-hexane/2-propanol = 95/5, flow rate = 1.0 mL/min, λ = 254 nm, retention time: 14.2 min (major), 15.9 min (minor).

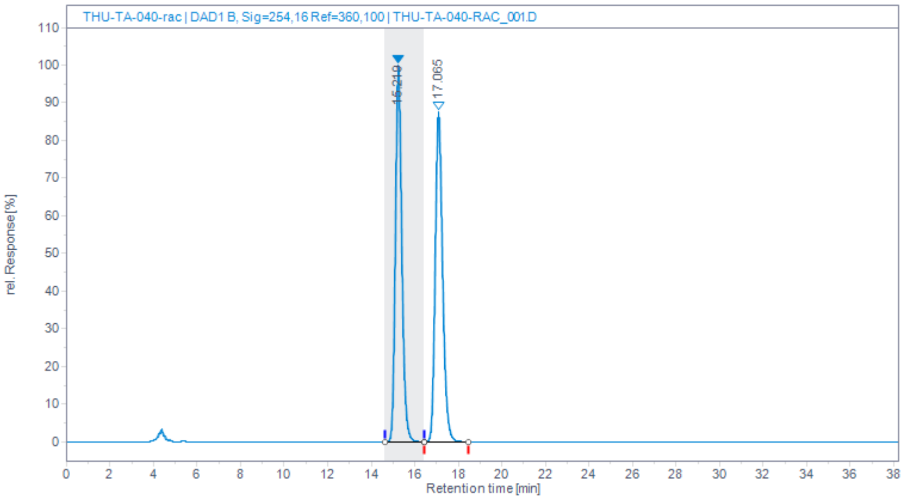

#	Name	Signal description	RT (min)	Area (mAU·s)	Area%	Height (mAU)	Height%	Amount	Concentration	Start time (min)	End time (min)
1		DAD1 B, Sig=254.16 Ref=360.100	15.219	25693.116	49.825	1265.460	53.32			14.650	16.420
2		DAD1 B, Sig=254.16 Ref=360.100	17.065	25873.169	50.175	1107.729	46.68			16.420	18.464

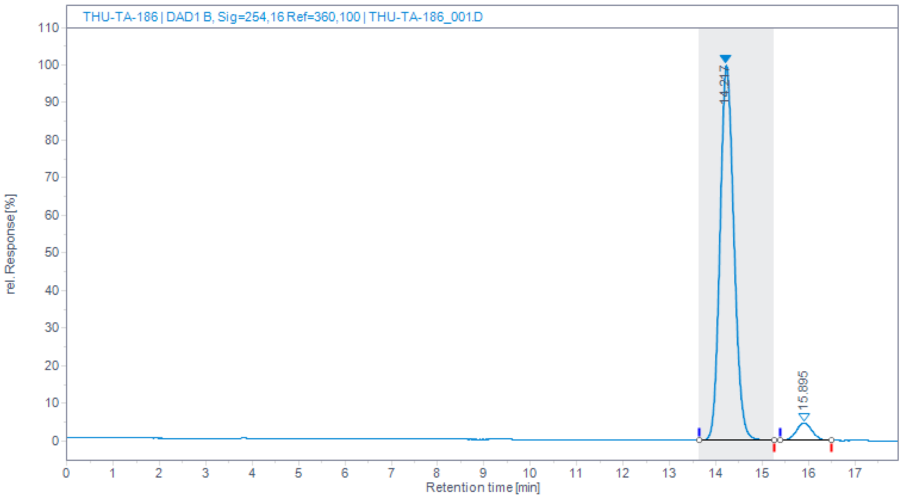

#	Name	Signal description	RT (min)	Area (mAU·s)	Area%	Height (mAU)	Height%	Amount	Concentration	Start time (min)	End time (min)
1		DAD1 B, Sig=254.16 Ref=360.100	14.217	3897.007	95.003	186.987	95.56			13.633	15.270
2		DAD1 B, Sig=254.16 Ref=360.100	15.895	204.980	4.997	8.693	4.44			15.400	16.492

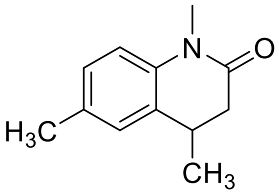

1,4,6-Trimethyl-3,4-dihydroquinolin-2(1*H*)-one (2d)

^{1}H NMR (400 MHz, $CDCl_3$) δ 7.05 (dd, *J* = 8.1, 2.0 Hz, 1H), 7.00 (d, *J* = 2.0 Hz, 1H), 6.88 (d, *J* = 8.2 Hz, 1H), 3.34 (d, *J* = 1.3 Hz, 3H), 3.07 – 2.94 (m, 1H), 2.70 (dd, *J* = 15.8, 5.5 Hz, 1H), 2.43 (dd, *J* = 15.8, 7.4 Hz, 1H), 2.32 (s, 3H), 1.26 (dd, *J* = 7.0, 1.2 Hz, 3H); **^{13}C NMR** (101 MHz, $CDCl_3$) δ 169.9, 137.5, 132.6, 131.0,

127.8, 127.1, 114.8, 39.3, 30.4, 29.5, 20.8, 19.4; **ESI-MS**: calculated $[C_{12}H_{15}NONa]^+$:212.1046, found:212.1043; **HPLC** DAICEL CHIRALCEL OD-H, *n*-hexane/2-propanol = 95/5, flow rate = 1.0 mL/min, λ = 254 nm, retention time: 10.5 min (major), 12.6 min (minor).

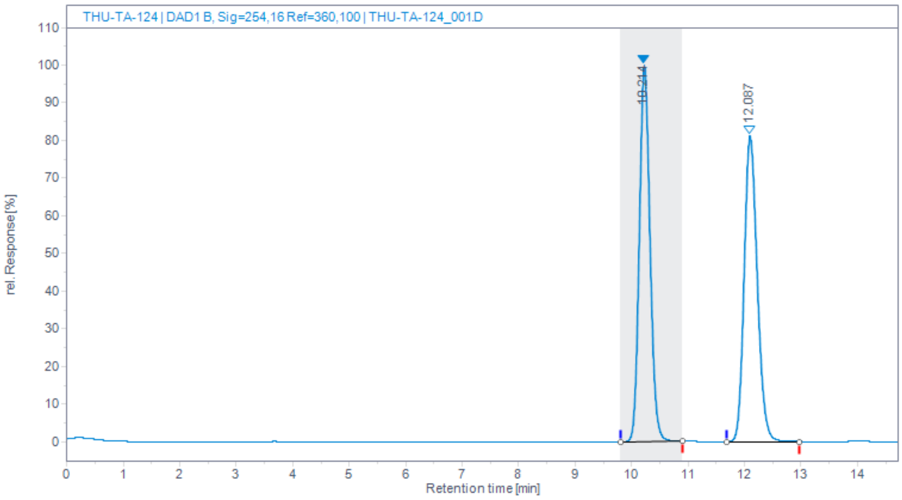

#	Name	Signal description	RT (min)	Area (mAU·s)	Area%	Height (mAU)	Height%	Amount	Concentration	Start time (min)	End time (min)
1		DAD1 B, Sig=254,16 Ref=360,100	10.214	5655.066	49.961	433.095	55.15			9.810	10.901
2		DAD1 B, Sig=254,16 Ref=360,100	12.087	5663.793	50.039	352.228	44.85			11.674	12.975

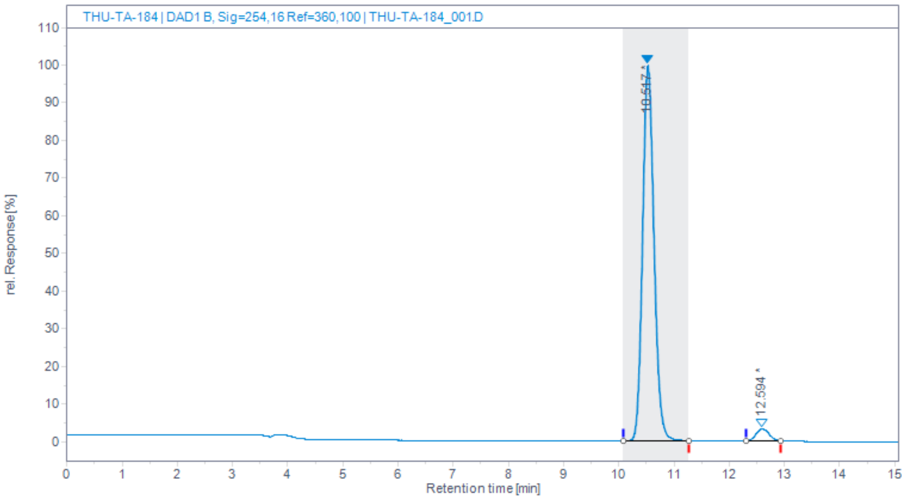

#	Name	Signal description	RT (min)	Area (mAU·s)	Area%	Height (mAU)	Height%	Amount	Concentration	Start time (min)	End time (min)
1		DAD1 B, Sig=254,16 Ref=360,100	10.517	4524.371	96.413	316.809	96.88			10.085	11.259
2		DAD1 B, Sig=254,16 Ref=360,100	12.594	168.350	3.587	10.201	3.12			12.296	12.944

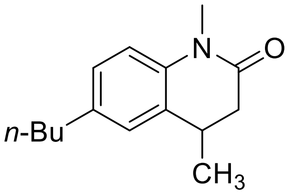

6-Butyl-1,4-dimethyl-3,4-dihydroquinolin-2(1*H*)-one (2e)

^{1}H NMR (400 MHz, $CDCl_3$) δ 7.06 (dd, *J* = 8.2, 2.1 Hz, 1H), 7.00 (d, *J* = 2.0 Hz, 1H), 6.90 (d, *J* = 8.2 Hz, 1H), 3.35 (s, 3H), 3.02 (td, *J* = 7.2, 5.5 Hz, 1H), 2.72 (dd, *J* = 15.8, 5.5 Hz, 1H), 2.62 – 2.53 (m, 2H), 2.45 (dd, *J* = 15.8, 7.4 Hz, 1H), 1.65 – 1.52 (m, 2H), 1.36 (dq, *J* = 14.6, 7.3 Hz, 2H), 1.27 (d, *J* = 7.0 Hz, 3H), 0.93 (t, *J* = 7.4 Hz, 3H); **^{13}C** NMR (101 MHz, $CDCl_3$) δ 169.9, 137.9, 137.6, 131.0, 127.2, 126.4, 114.8, 39.3, 35.1, 33.9, 30.5, 29.5, 22.5, 19.5, 14.1; **ESI-MS**: calculated

$[C_{15}H_{21}NONa]^+$:254.1515, found:254.1513; **HPLC** DAICEL CHIRALCEL OD-H, *n*-hexane/2-propanol = 95/5, flow rate = 1.0 mL/min, λ = 254 nm, retention time: 8.2 min (major), 9.4 min (minor).

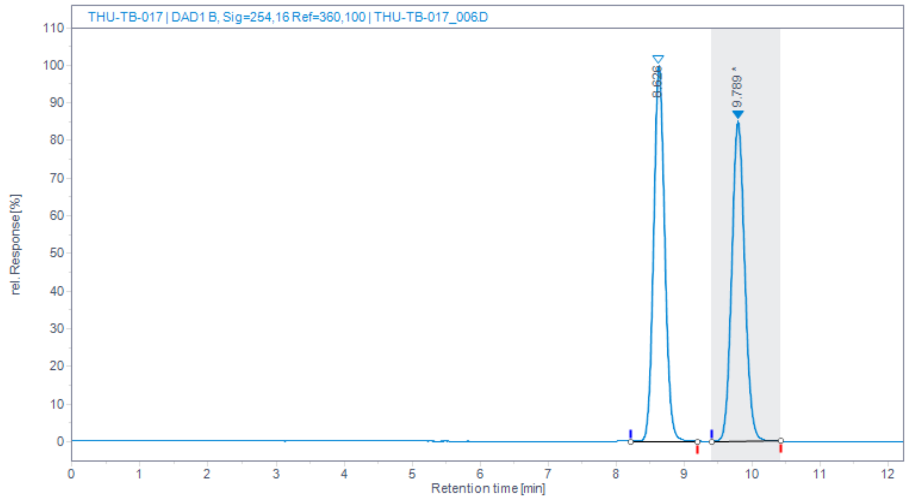

#	Name	Signal description	RT (min)	Area (mAU·s)	Area%	Height (mAU)	Height%	Amount	Concentration	Start time (min)	End time (min)
1		DAD1 B, Sig=254,16 Ref=360,100	8.626	1821.121	50.077	161.068	54.00			8.214	9.203
2		DAD1 B, Sig=254,16 Ref=360,100	9.789	1815.509	49.923	137.209	46.00			9.416	10.420

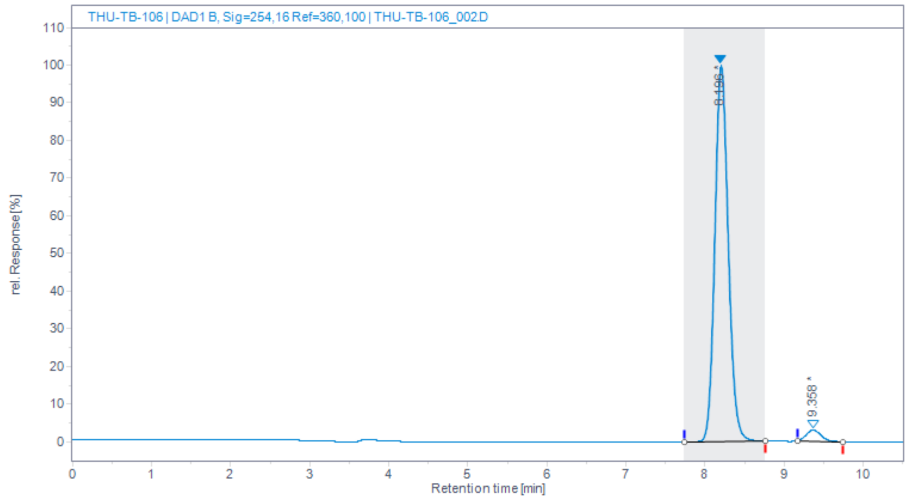

#	Name	Signal description	RT (min)	Area (mAU·s)	Area%	Height (mAU)	Height%	Amount	Concentration	Start time (min)	End time (min)
1		DAD1 B, Sig=254,16 Ref=360,100	8.196	6599.567	96.925	560.983	97.14			7.743	8.755
2		DAD1 B, Sig=254,16 Ref=360,100	9.358	209.348	3.075	16.494	2.86			9.162	9.734

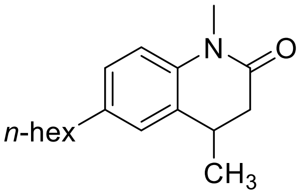

6-Hexyl-1,4-dimethyl-3,4-dihydroquinolin-2(1*H*)-one (**2f**)

^{1}H NMR (400 MHz, $CDCl_3$) δ 7.06 (dd, *J* = 8.2, 2.1 Hz, 1H), 7.00 (d, *J* = 2.0 Hz, 1H), 6.90 (d, *J* = 8.2 Hz, 1H), 3.35 (s, 3H), 3.02 (td, *J* = 7.2, 5.5 Hz, 1H), 2.72 (dd, *J* = 15.8, 5.5 Hz, 1H), 2.57 (dd, *J* = 8.8, 6.8 Hz, 2H), 2.44 (dd, *J* = 15.8, 7.4 Hz, 1H), 1.59 (td, *J* = 8.4, 4.9 Hz, 2H), 1.41 – 1.24 (m, 9H), 0.93 – 0.85 (m, 3H); **^{13}C NMR** (101 MHz, $CDCl_3$) δ 169.9, 137.9, 137.7, 131.0, 127.2, 126.4, 114.8, 39.4, 35.4, 31.8, 31.7, 30.5, 29.5, 29.1, 22.7, 19.5, 14.2; **ESI-MS**: calculated $[C_{17}H_{25}NONa]^+$:282.1828, found:282.1826; **HPLC** DAICEL CHIRALCEL OD-H, *n*-hexane/2-propanol = 95/5, flow rate = 1.0 mL/min, λ = 254 nm, retention time: 7.8 min (major), 8.8 min (minor).

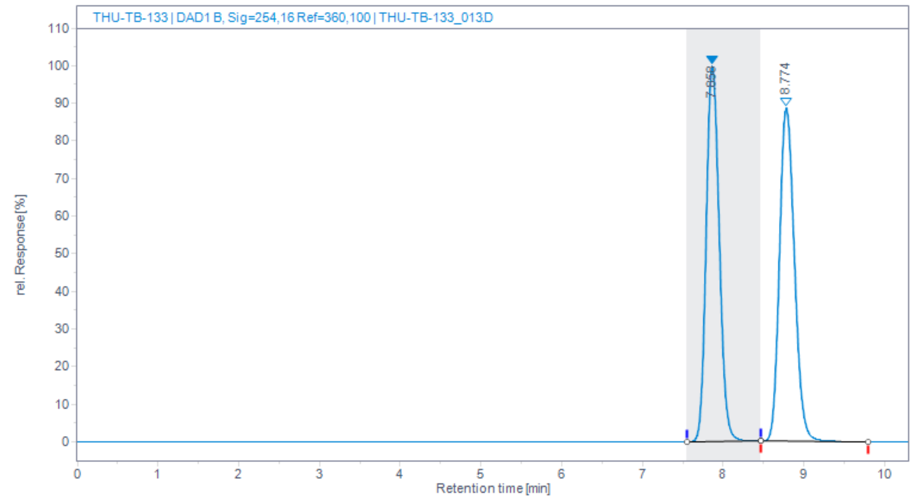

#	Name	Signal description	RT (min)	Area (mAU·s)	Area%	Height (mAU)	Height%	Amount	Concentration	Start time (min)	End time (min)
1		DAD1 B, Sig=254,16 Ref=360,100	7.858	16222.338	49.524	1444.565	52.95			7.557	8.460
2		DAD1 B, Sig=254,16 Ref=360,100	8.774	16533.919	50.476	1283.403	47.05			8.460	9.800

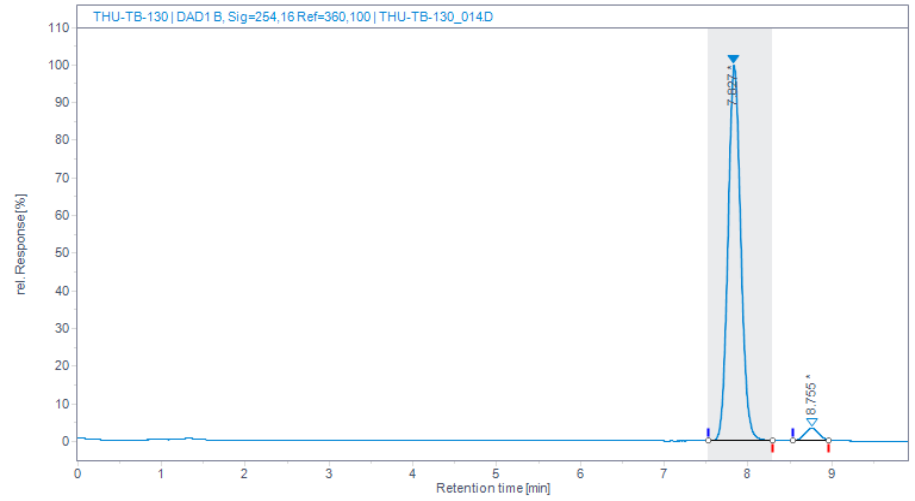

#	Name	Signal description	RT (min)	Area (mAU·s)	Area%	Height (mAU)	Height%	Amount	Concentration	Start time (min)	End time (min)
1		DAD1 B, Sig=254,16 Ref=360,100	7.827	1678.328	96.612	158.859	96.85			7.529	8.285
2		DAD1 B, Sig=254,16 Ref=360,100	8.755	58.855	3.388	5.172	3.15			8.527	8.953

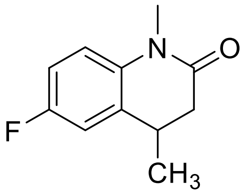

6-Fluoro-1,4-dimethyl-3,4-dihydroquinolin-2(1*H*)-one (2g)

^{1}H NMR (400 MHz, $CDCl_3$) δ 6.99 – 6.87 (m, 3H), 3.35 (d, *J* = 1.0 Hz, 3H), 3.09 – 2.96 (m, 1H), 2.71 (dd, *J* = 15.8, 5.4 Hz, 1H), 2.42 (dd, *J* = 15.9, 8.2 Hz, 1H), 1.28 (d, *J* = 7.0 Hz, 3H); **^{13}C NMR** (101 MHz, $CDCl_3$) δ 169.6, 158.9 (d, *J* = 242.6 Hz), 136.2 (d, *J* = 2.5 Hz), 133.3 (d, *J* = 7.1 Hz), 116.0(d, *J* = 8.2 Hz), 113.6 (d, *J* = 22.2 Hz), 113.4 (d, *J* = 23.0 Hz), 39.0, 30.3 (d, *J* = 1.4 Hz), 29.8, 19.0; **^{19}F NMR** (377 MHz, $CDCl_3$) δ -120.47; **ESI-MS**: calculated $[C_{11}H_{12}NOFNa]^+$:216.0795, found:216.0793; **HPLC** DAICEL CHIRALCEL OD-H, *n*-hexane/2-propanol = 95/5, flow rate = 1.0 mL/min, λ = 254 nm, retention time: 14.2 min (major), 15.2 min (minor).

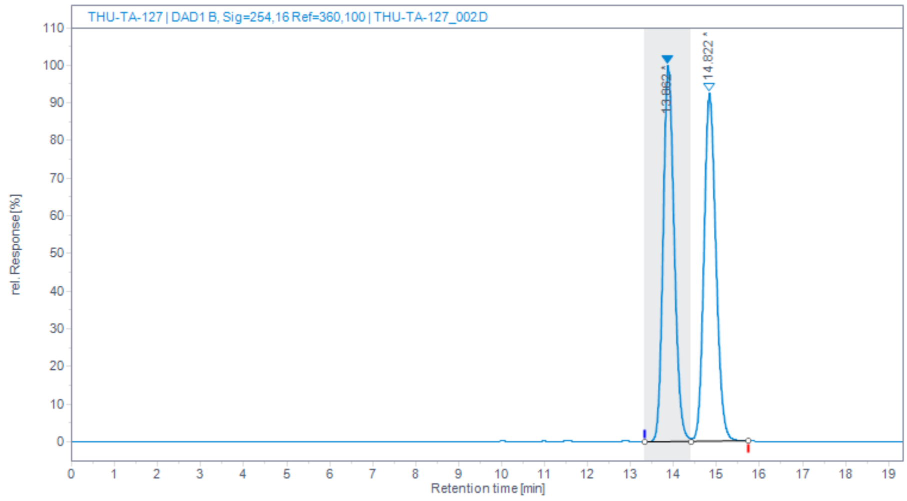

#	Name	Signal description	RT (min)	Area (mAU·s)	Area%	Height (mAU)	Height%	Amount	Concentration	Start time (min)	End time (min)
1		DAD1 B, Sig=254,16 Ref=360,100	13.862	6548.482	49.902	367.899	51.90			13.317	14.406
2		DAD1 B, Sig=254,16 Ref=360,100	14.822	6574.307	50.098	340.901	48.10			14.406	15.725

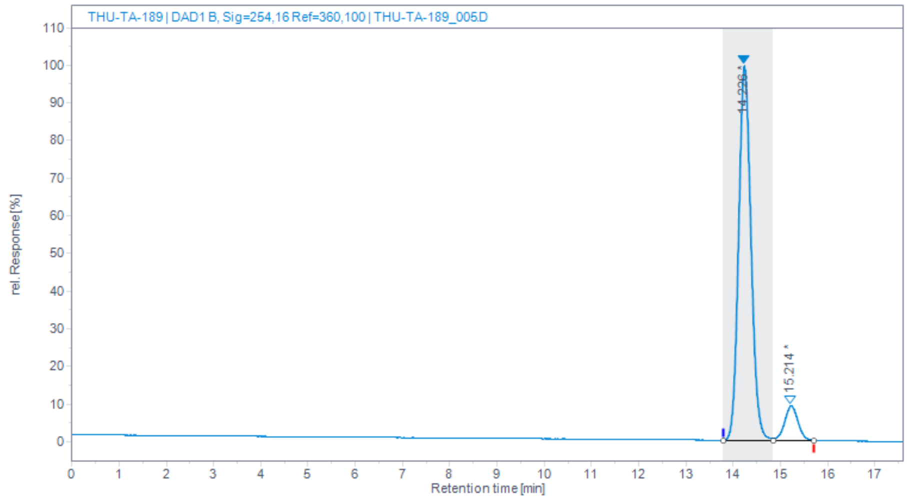

#	Name	Signal description	RT (min)	Area (mAU·s)	Area%	Height (mAU)	Height%	Amount	Concentration	Start time (min)	End time (min)
1		DAD1 B, Sig=254,16 Ref=360,100	14.226	1409.838	91.087	77.799	91.65			13.782	14.842
2		DAD1 B, Sig=254,16 Ref=360,100	15.214	137.960	8.913	7.085	8.35			14.842	15.694

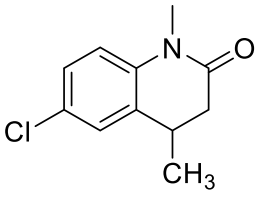

6-Chloro-1,4-dimethyl-3,4-dihydroquinolin-2(1*H*)-one (**2h**)

^{1}H NMR (400 MHz, $CDCl_3$) δ 7.28 – 7.14 (m, 2H), 6.90 (d, *J* = 8.6 Hz, 1H), 3.34 (s, 3H), 3.09 – 2.96 (m, 1H), 2.71 (dd, *J* = 15.9, 5.4 Hz, 1H), 2.44 (dd, *J* = 15.9, 7.9 Hz, 1H), 1.28 (dd, J = 7.0, 1.3 Hz, 3H); **^{13}C NMR** (101 MHz, $CDCl_3$) δ 169.6, 138.6, 132.9, 128.4, 127.3, 126.4, 116.1, 39.0, 30.3, 29.7, 19.2; **ESI-MS**: calculated $[C_{11}H_{12}NOClNa]^+$:232.0500, found:232.0497; **HPLC** DAICEL CHIRALCEL OD-H, *n*-hexane/2-propanol = 95/5, flow rate = 1.0 mL/min, λ = 254 nm, retention time: 15.0 min (major), 17.5 min (minor).

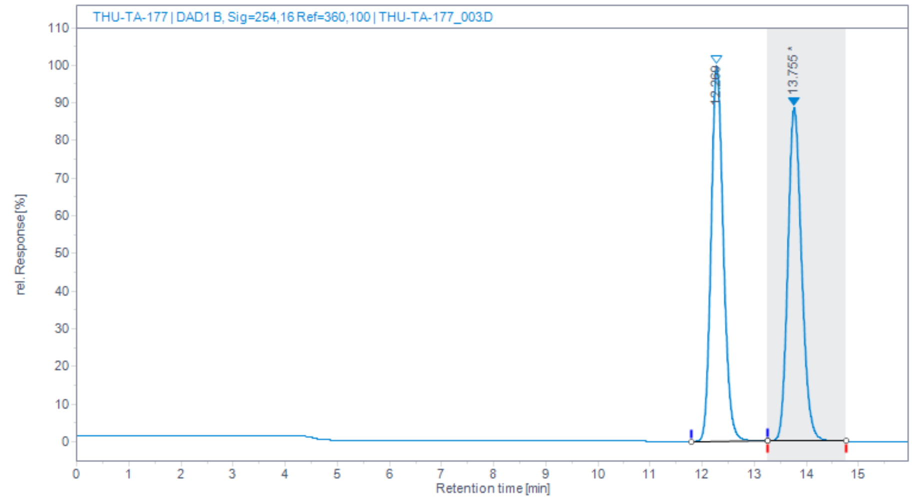

#	Name	Signal description	RT (min)	Area (mAU·s)	Area%	Height (mAU)	Height%	Amount	Concentration	Start time (min)	End time (min)
1		DAD1 B, Sig=254,16 Ref=360,100	12.269	6531.472	50.083	398.592	52.97			11.796	13.259
2		DAD1 B, Sig=254,16 Ref=360,100	13.755	6509.775	49.917	353.887	47.03			13.259	14.777

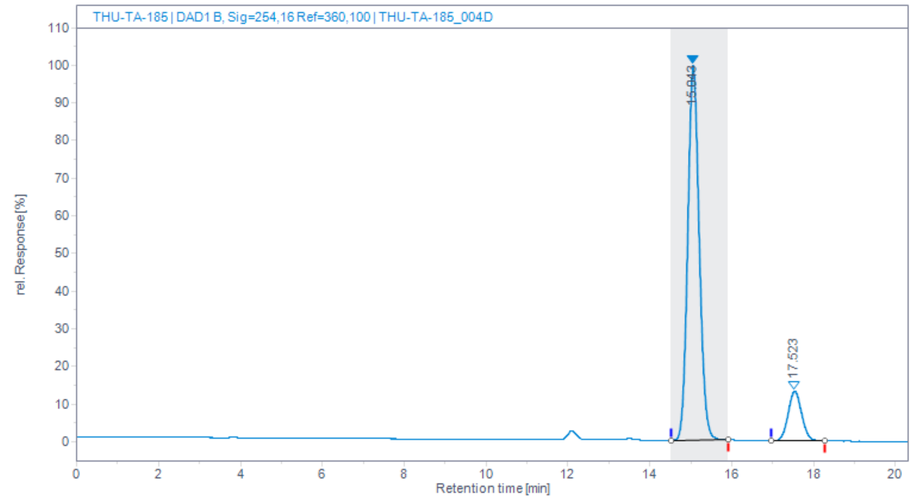

#	Name	Signal description	RT (min)	Area (mAU·s)	Area%	Height (mAU)	Height%	Amount	Concentration	Start time (min)	End time (min)
1		DAD1 B, Sig=254,16 Ref=360,100	15.043	3240.679	86.463	166.707	88.28			14.504	15.921
2		DAD1 B, Sig=254,16 Ref=360,100	17.523	507.365	13.537	22.126	11.72			16.968	18.268

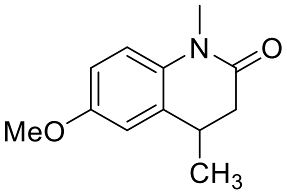

6-Methoxy-1,4-dimethyl-3,4-dihydroquinolin-2(1*H*)-one (2i)

^{1}H-NMR (400 MHz, $CDCl_3$) δ 6.94 – 6.87 (m, 1H), 6.81 – 6.73 (m, 2H), 3.80 (s, 3H), 3.34 (s, 3H), 3.01 (td, *J* = 7.3, 5.5 Hz, 1H), 2.70 (dd, *J* = 15.8, 5.4 Hz, 1H), 2.42 (dd, *J* = 15.8, 7.7 Hz, 1H), 1.26 (d, *J* = 7.0 Hz, 3H); **^{13}C-NMR** (101 MHz, $CDCl_3$) δ 169.5, 155.6, 133.6, 132.8, 115.7, 112.8, 111.5, 55.7, 39.2, 30.6, 29.6, 19.3; **ESI-MS**: calc. for $C_{12}H_{15}NO_2Na$ $[M+Na]^+$ 228.0995, found 228.0991; **HPLC** DAICEL CHIRALCEL OD-H, *n*-hexane/2-propanol = 95/5, flow rate = 1.0 mL/min, λ = 254 nm, retention time: 17.9 min (major), 20.8 min (minor).

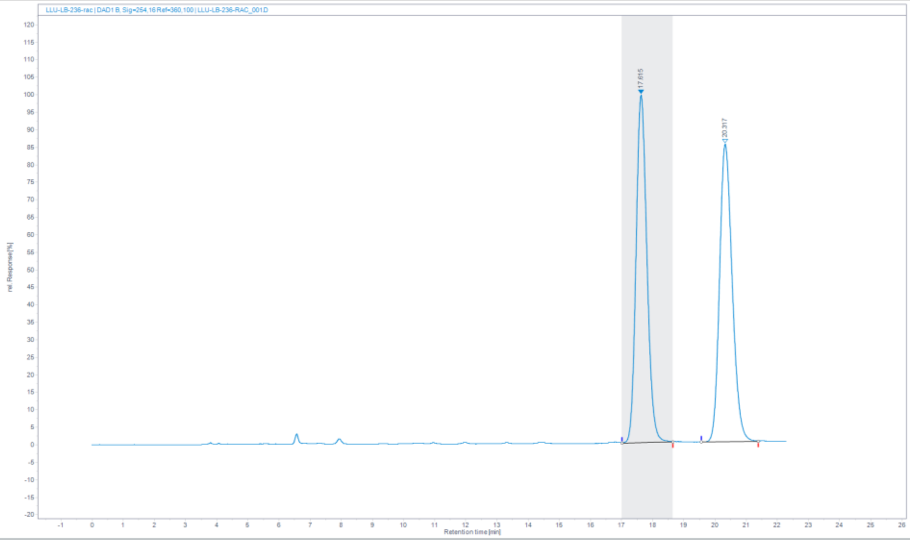

#	Name	Signal description	RT (min)	Area (mAU·s)	Area%	Height (mAU)	Height%	Amount	Concentration	Start time (min)	End time (min)
1		DAD1 B, Sig=254,16 Ref=360,100	17.615	3309.010	50.002	136.678	53.87			17.018	18.655
2		DAD1 B, Sig=254,16 Ref=360,100	20.317	3308.754	49.998	117.045	46.13			19.561	21.379

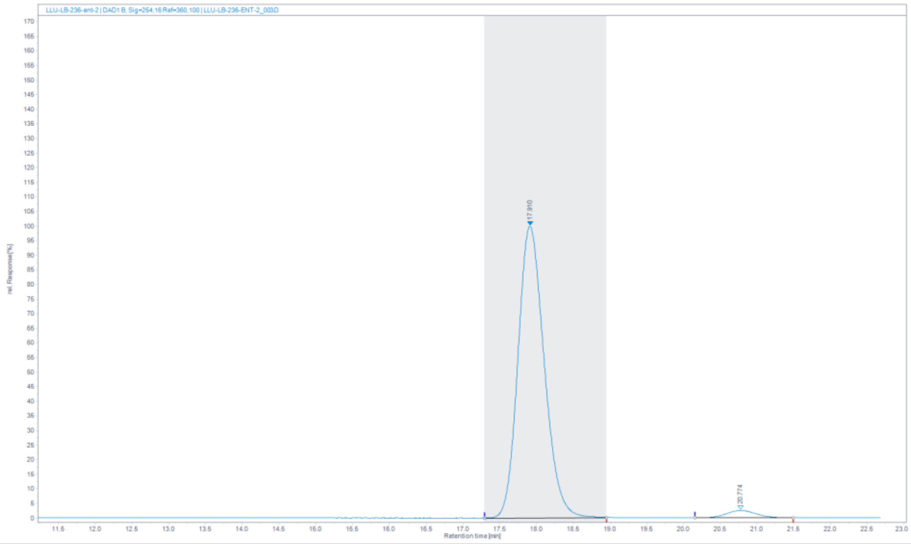

#	Name	Signal description	RT (min)	Area (mAU·s)	Area%	Height (mAU)	Height%	Amount	Concentration	Start time (min)	End time (min)
1		DAD1 B, Sig=254,16 Ref=360,100	17.910	4179.301	97.134	168.934	97.50			17.294	18.957
2		DAD1 B, Sig=254,16 Ref=360,100	20.774	123.330	2.866	4.324	2.50			20.157	21.493

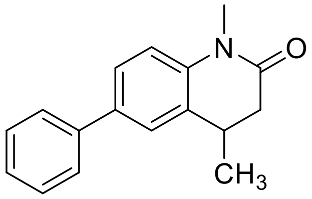

1,4-Dimethyl-6-phenyl-3,4-dihydroquinolin-2(1*H*)-one (**2j**)

^{1}H NMR (400 MHz, $CDCl_3$) δ 7.61 – 7.55 (m, 2H), 7.53 – 7.40 (m, 4H), 7.39 – 7.30 (m, 1H), 7.07 (d, *J* = 8.4 Hz, 1H), 3.41 (s, 3H), 3.13 (td, *J* = 7.2, 5.5 Hz, 1H), 2.78 (dd, *J* = 15.8, 5.5 Hz, 1H), 2.51 (dd, *J* = 15.9, 7.4 Hz, 1H), 1.34 (d, *J* = 7.0 Hz, 3H); **^{13}C NMR** (101 MHz, $CDCl_3$) δ 169.9, 140.5, 139.2, 136.2, 131.5, 129.0, 127.3, 126.9, 126.1, 125.2, 115.3, 39.3, 30.6, 29.6, 19.5; **ESI-MS**: calculated $[C_{17}H_{17}NONa]^+$:274.1202, found:274.1200; **HPLC** DAICEL CHIRALCEL OD-H, *n*-hexane/2-propanol = 90/10, flow rate = 1.0 mL/min, λ = 254 nm, retention time: 9.3 min (major), 10.5 min (minor).

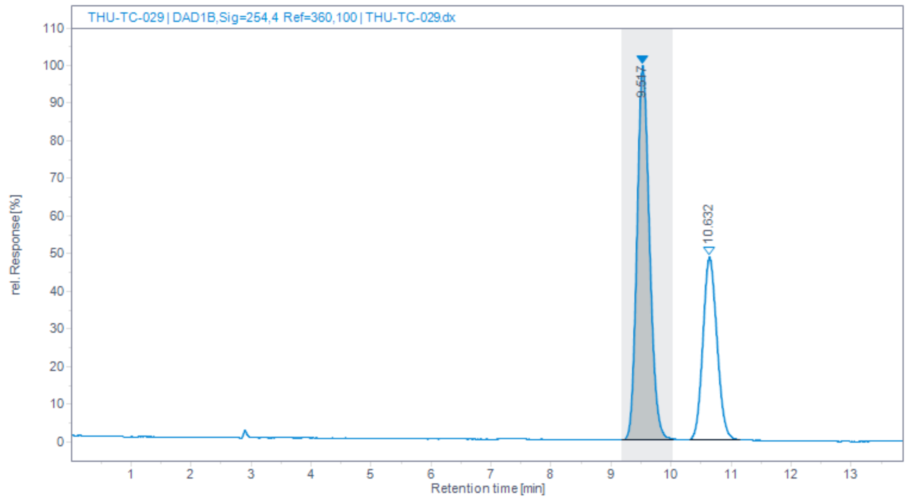

#	Name	Signal description	RT (min)	Area (mAU·s)	Area%	Height (mAU)	Height%	Amount	Concentration	Start time (min)	End time (min)
1		DAD1B,Sig=254,4 Ref=360,100	9.517	191.307	64.821	13.129	67.21			9.181	10.034
2		DAD1B,Sig=254,4 Ref=360,100	10.632	103.822	35.179	6.405	32.79			10.304	11.150

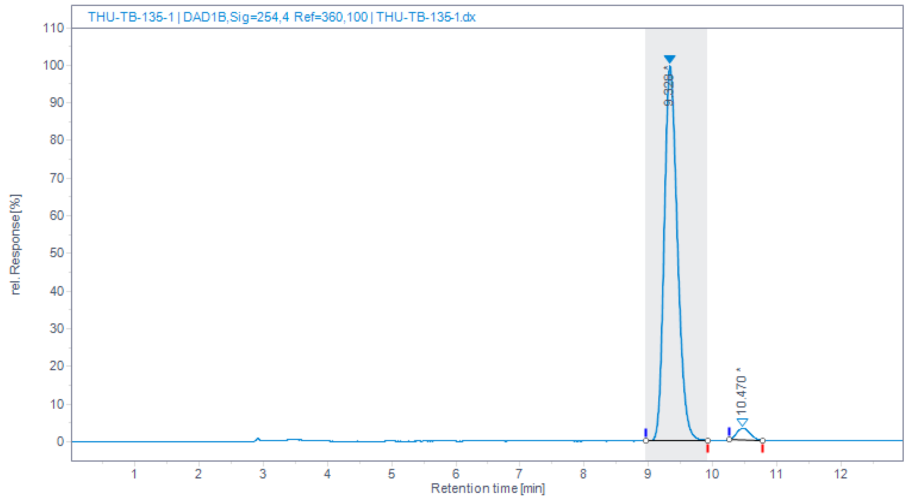

#	Name	Signal description	RT (min)	Area (mAU·s)	Area%	Height (mAU)	Height%	Amount	Concentration	Start time (min)	End time (min)
1		DAD1B,Sig=254,4 Ref=360,100	9.328	512.966	96.941	35.929	97.01			8.951	9.922
2		DAD1B,Sig=254,4 Ref=360,100	10.470	16.186	3.059	1.108	2.99			10.251	10.771

After recrystallization:

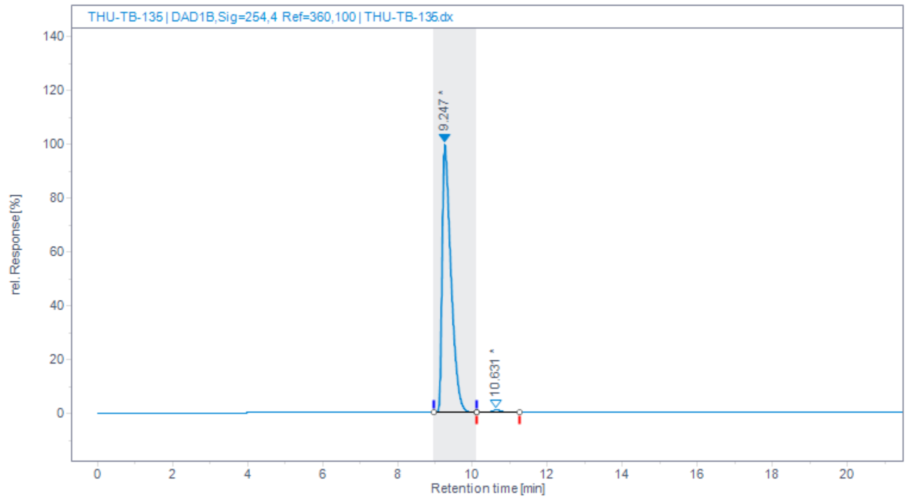

#	Name	Signal description	RT (min)	Area (mAU·s)	Area%	Height (mAU)	Height%	Amount	Concentration	Start time (min)	End time (min)
1		DAD1B,Sig=254,4 Ref=360,100	9.247	10213.404	99.078	628.694	98.96			8.978	10.096
2		DAD1B,Sig=254,4 Ref=360,100	10.631	95.027	0.922	6.580	1.04			10.096	11.261

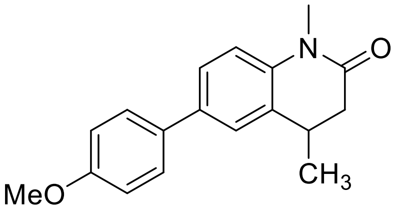

6-(4-Methoxyphenyl)-1,4-dimethyl-3,4-dihydroquinolin-2(1*H*)-one (2k) **^{1}H-NMR** (400 MHz, $CDCl_3$) δ 7.54 – 7.48 (m, 2H), 7.44 (dd, *J* = 8.4, 2.2 Hz, 1H), 7.38 (dd, *J* = 2.1, 0.9 Hz, 1H), 7.04 (d, *J* = 8.4 Hz, 1H), 7.02 – 6.94 (m, 2H), 3.86 (s, 3H), 3.40 (s, 3H), 3.18 – 3.05 (m, 1H), 2.77 (dd, *J* = 15.8, 5.5 Hz, 1H), 2.50 (dd, *J* = 15.8, 7.3 Hz, 1H), 1.33 (d, *J* = 7.0 Hz, 3H);**^{13}C-NMR** (101 MHz, $CDCl_3$) δ 169.9, 159.2, 138.7, 135.9, 133.1, 131.5, 128.0, 125.6, 124.8, 115.3, 114.4, 55.5, 39.3, 30.7, 29.6, 19.6; **ESI-MS**: calc. for $C_{18}H_{19}NO_2Na$ $[M+Na]^+$ 304.1308, found 304.1306; **HPLC** DAICEL CHIRALCEL OD-H, *n*-hexane/2-propanol = 95/5, flow rate = 1.0 mL/min, λ = 254 nm, retention time: 33.3 min (major), 41.0 min (minor).

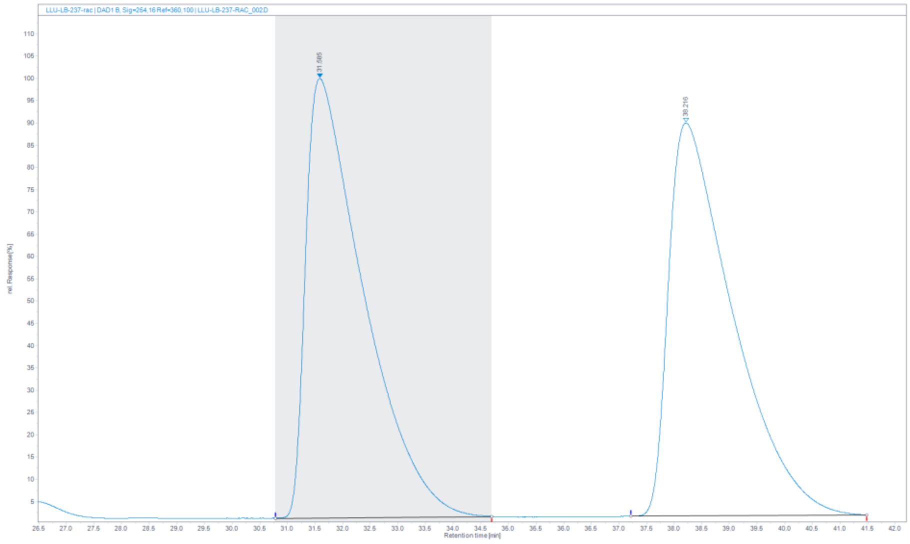

#	Name	Signal description	RT (min)	Area (mAU·s)	Area%	Height (mAU)	Height%	Amount	Concentration	Start time (min)	End time (min)
1		DAD1 B, Sig=254,16 Ref=360,100	31.585	13265.933	50.148	182.314	52.79			30.788	34.699
2		DAD1 B, Sig=254,16 Ref=360,100	38.216	13187.807	49.852	163.051	47.21			37.211	41.485

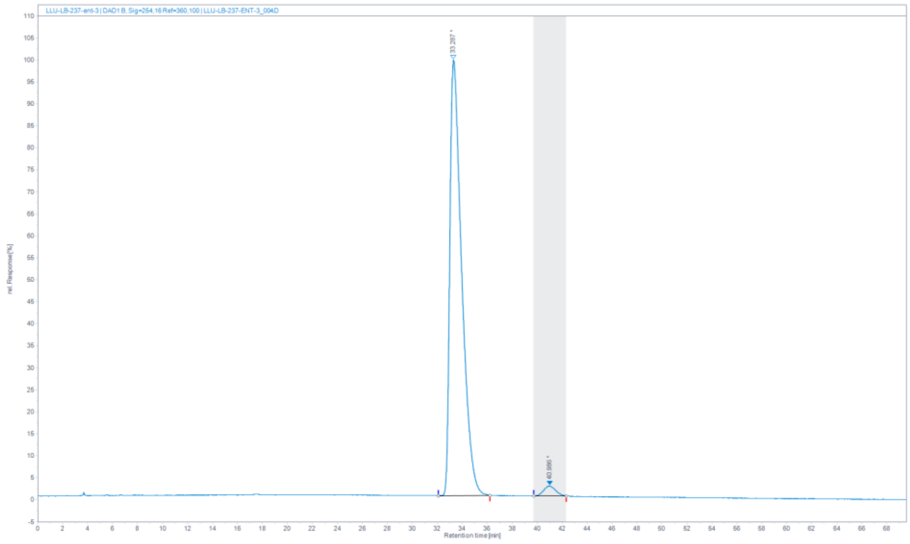

#	Name	Signal description	RT (min)	Area (mAU·s)	Area%	Height (mAU)	Height%	Amount	Concentration	Start time (min)	End time (min)
1		DAD1 B, Sig=254,16 Ref=360,100	33.287	3587.341	97.784	54.673	97.79			32.063	36.191
2		DAD1 B, Sig=254,16 Ref=360,100	40.986	81.303	2.216	1.236	2.21			39.703	42.313

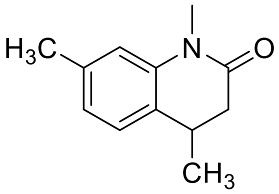

1,4,7-Trimethyl-3,4-dihydroquinolin-2(1*H*)-one (**2l**)

^{1}H NMR (400 MHz, $CDCl_3$) δ 7.08 (dd, *J* = 7.5, 2.1 Hz, 1H), 6.89 – 6.84 (m, 1H), 6.81 (s, 1H), 3.36 (d, *J* = 2.3 Hz, 3H), 3.00 (p, *J* = 6.7 Hz, 1H), 2.75 – 2.65 (m, 1H), 2.50 – 2.29 (m, 4H), 1.30 – 1.22 (m, 3H); **^{13}C NMR** (101 MHz, $CDCl_3$) δ 170.2, 139.8, 137.3, 128.2, 126.2, 123.7, 115.8, 39.5, 30.0, 29.5, 21.6, 19.5; **ESI-MS**: calculated $[C_{12}H_{16}NO]^+$:190.1226, found:190.1224; **HPLC** DAICEL CHIRALCEL OD-H, *n*-hexane/2-propanol = 95/5, flow rate = 1.0 mL/min, λ = 254 nm, retention time: 10.1 min (major), 11.1 min (minor).

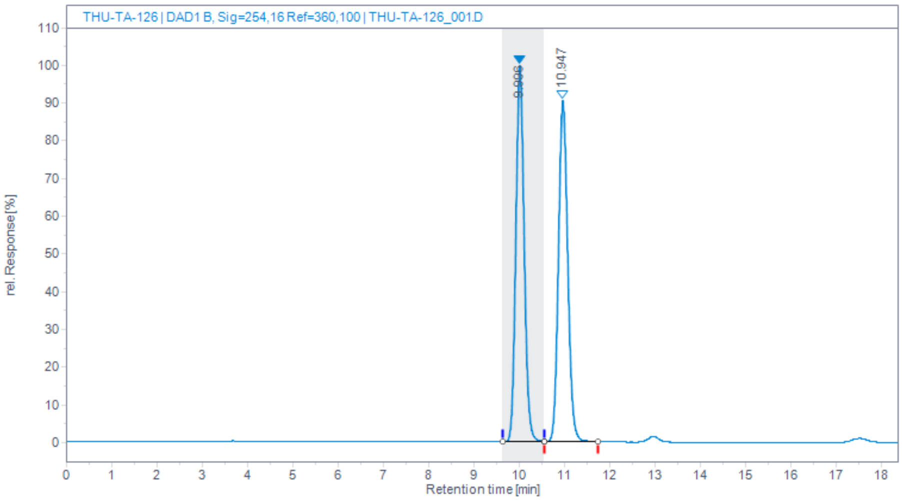

#	Name	Signal description	RT (min)	Area (mAU·s)	Area%	Height (mAU)	Height%	Amount	Concentration	Start time (min)	End time (min)
1		DAD1 B, Sig=254,16 Ref=360,100	9.996	1167.827	49.695	91.924	52.45			9.629	10.554
2		DAD1 B, Sig=254,16 Ref=360,100	10.947	1182.173	50.305	83.331	47.55			10.554	11.745

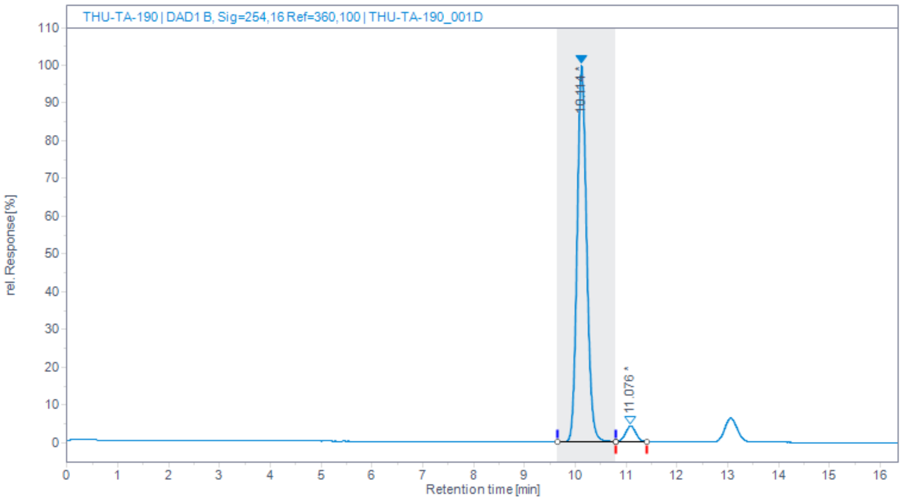

#	Name	Signal description	RT (min)	Area (mAU·s)	Area%	Height (mAU)	Height%	Amount	Concentration	Start time (min)	End time (min)
1		DAD1 B, Sig=254,16 Ref=360,100	10.114	2553.579	95.786	197.633	96.06			9.638	10.786
2		DAD1 B, Sig=254,16 Ref=360,100	11.076	112.341	4.214	8.100	3.94			10.786	11.396

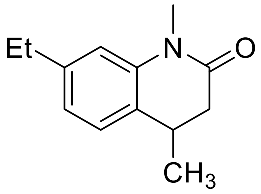

7-Ethyl-1,4-dimethyl-3,4-dihydroquinolin-2(1*H*)-one (**2m**)

^{1}H NMR (400 MHz, $CDCl_3$) δ 7.11 (dd, *J* = 7.7, 0.9 Hz, 1H), 6.90 (dd, *J* = 7.7, 1.6 Hz, 1H), 6.83 (d, *J* = 1.6 Hz, 1H), 3.38 (s, 3H), 3.02 (td, *J* = 7.3, 5.5 Hz, 1H), 2.78 – 2.58 (m, 3H), 2.44 (dd, *J* = 15.8, 7.8 Hz, 1H), 1.30 – 1.20 (m, 6H); **^{13}C NMR** (101 MHz, $CDCl_3$) δ 170.2, 143.8, 139.9, 128.5, 126.2, 122.5, 114.7, 39.4, 30.1, 29.6, 29.0, 19.5, 15.8; **ESI-MS**: calculated $[C_{13}H_{18}NO]^{+}$:204.1383, found:204.1380; **HPLC** DAICEL CHIRALCEL OD-H, *n*-hexane/2-propanol = 95/5, flow rate = 1.0 mL/min, λ = 230 nm, retention time: 9.2 min (major), 9.8 min (minor).

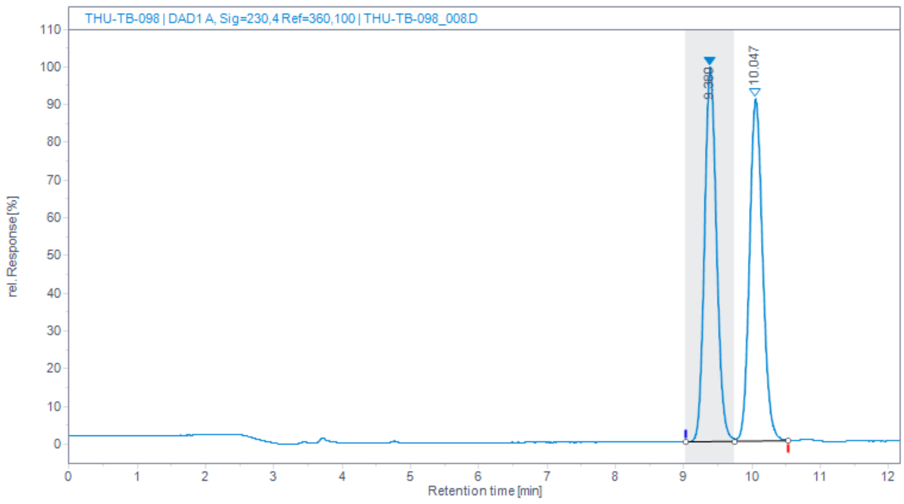

#	Name	Signal description	RT (min)	Area (mAU·s)	Area%	Height (mAU)	Height%	Amount	Concentration	Start time (min)	End time (min)
1		DAD1 A, Sig=230,4 Ref=360,100	9.380	1981.392	49.947	162.503	52.21			9.038	9.753
2		DAD1 A, Sig=230,4 Ref=360,100	10.047	1985.630	50.053	148.773	47.79			9.753	10.533

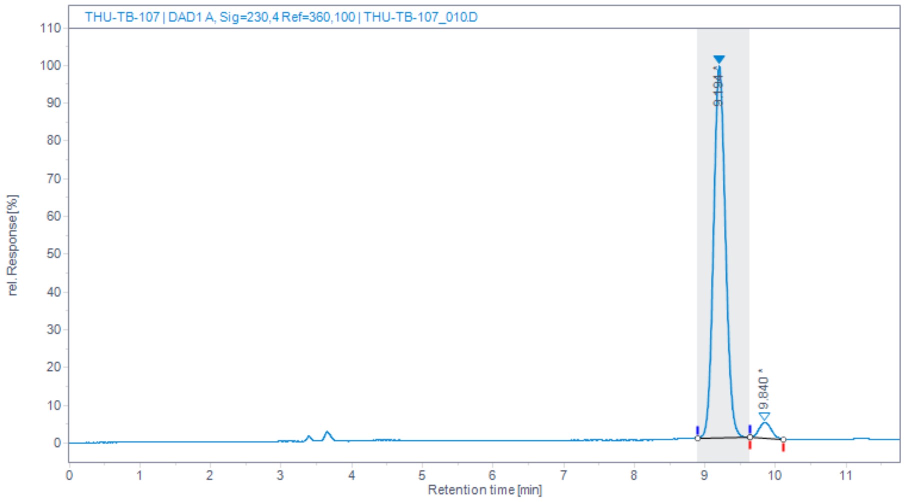

#	Name	Signal description	RT (min)	Area (mAU·s)	Area%	Height (mAU)	Height%	Amount	Concentration	Start time (min)	End time (min)
1		DAD1 A, Sig=230,4 Ref=360,100	9.194	602.047	95.697	51.669	95.86			8.901	9.629
2		DAD1 A, Sig=230,4 Ref=360,100	9.840	27.068	4.303	2.230	4.14			9.629	10.113

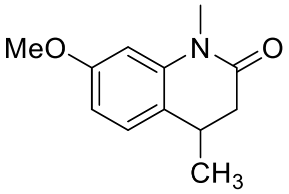

7-Methoxy-1,4-dimethyl-3,4-dihydroquinolin-2(1*H*)-one (**2n**)

^{1}H NMR (400 MHz, $CDCl_3$) δ 7.09 (dt, *J* = 7.7, 1.0 Hz, 1H), 6.61 – 6.53 (m, 2H), 3.81 (s, 3H), 3.34 (s, 3H), 3.06 – 2.93 (m, 1H), 2.70 (dd, *J* = 15.8, 5.4 Hz, 1H), 2.42 (dd, *J* = 15.8, 7.9 Hz, 1H), 1.24 (d, *J* = 7.0 Hz, 3H); **^{13}C NMR** (101 MHz, $CDCl_3$) δ 170.2, 159.2, 141.0, 126.9, 123.5, 106.7, 102.6, 55.5, 39.6, 29.6, 29.6, 19.6; **ESI-MS**: calculated $[C_{12}H_{15}NO_2Na]^+$:228.0995, found:228.0992; **HPLC** DAICEL CHIRALCEL OD-H, *n*-hexane/2-propanol = 95/5, flow rate = 1.0 mL/min, λ = 254 nm, retention time: 14.1 min (major), 17.6 min (minor).

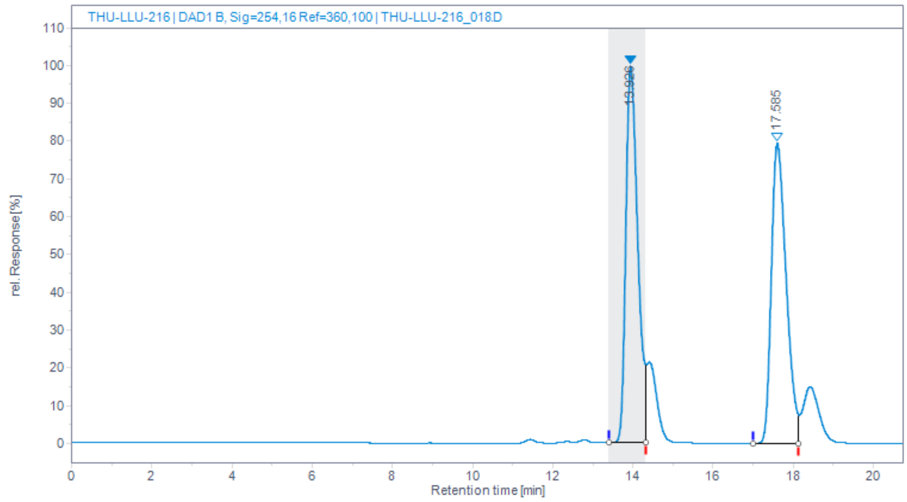

#	Name	Signal description	RT (min)	Area (mAU·s)	Area%	Height (mAU)	Height%	Amount	Concentration	Start time (min)	End time (min)
1		DAD1 B, Sig=254,16 Ref=360,100	13.926	22467.975	50.119	1089.781	55.71			13.399	14.310
2		DAD1 B, Sig=254,16 Ref=360,100	17.585	22361.352	49.881	866.506	44.29			17.001	18.139

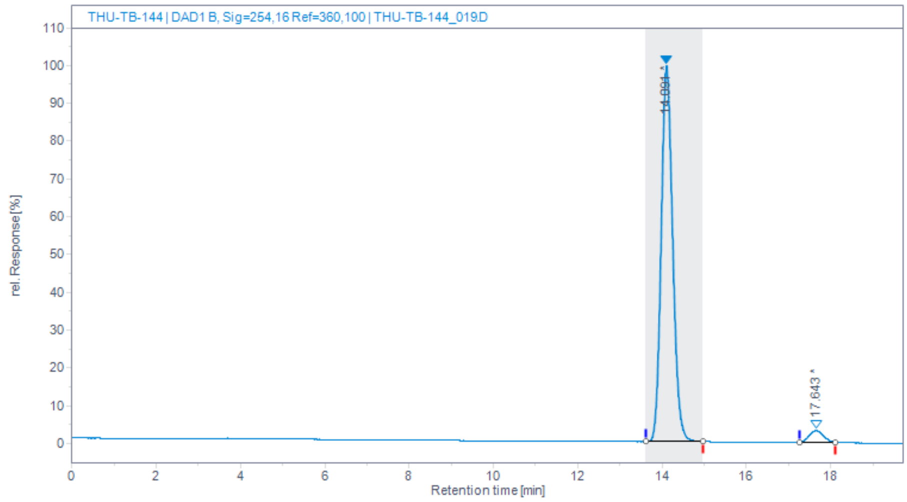

#	Name	Signal description	RT (min)	Area (mAU·s)	Area%	Height (mAU)	Height%	Amount	Concentration	Start time (min)	End time (min)
1		DAD1 B, Sig=254,16 Ref=360,100	14.091	2383.753	96.545	126.523	97.11			13.616	14.956
2		DAD1 B, Sig=254,16 Ref=360,100	17.643	85.317	3.455	3.765	2.89			17.244	18.092

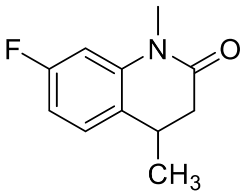

7-Fluoro-1,4-dimethyl-3,4-dihydroquinolin-2(1*H*)-one (**2o**)

^{1}H NMR (400 MHz, $CDCl_3$) δ 7.17 – 7.09 (m, 1H), 6.78 – 6.67 (m, 2H), 3.34 (s, 3H), 3.10 – 2.97 (m, 1H), 2.72 (dd, *J* = 15.8, 5.4 Hz, 1H), 2.45 (dd, *J* = 15.8, 7.7

Hz, 1H), 1.26 (d, J = 7.0 Hz, 3H); **^{13}C NMR** (101 MHz, $CDCl_3$) δ 169.9, 162.3 (d, J = 243.5 Hz), 141.4 (d, J = 9.9 Hz), 127.4 (d, J = 9.3 Hz), 126.7, 109.2 (d, J = 21.0 Hz), 102.9 (d, J = 26.5 Hz), 39.3, 29.9, 29.6, 19.5; **^{19}F NMR** (377 MHz, $CDCl_3$) δ -114.30; **ESI-MS**: calculated $[C_{11}H_{12}NOFNa]^+$:216.0795, found:216.0793; **HPLC** DAICEL CHIRALCEL OD-H, *n*-hexane/2-propanol = 95/5, flow rate = 1.0 mL/min, λ = 254 nm, retention time: 8.4 min (major), 9.6 min (minor).

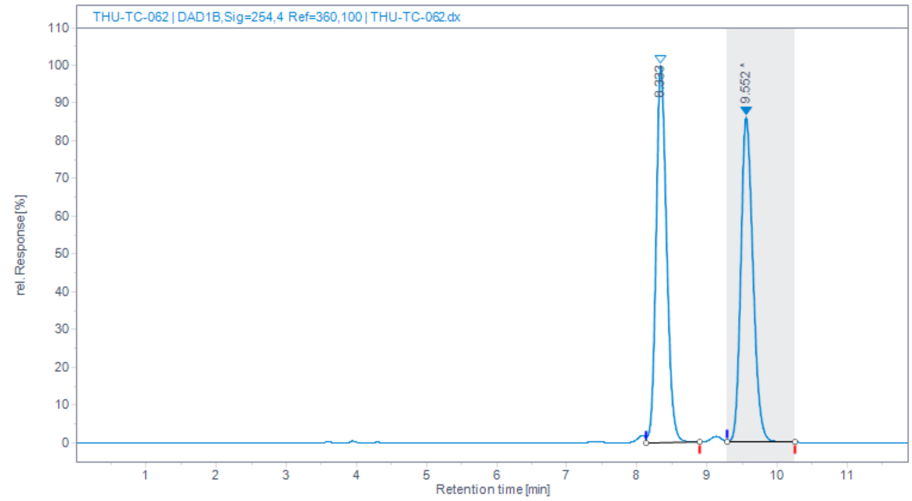

#	Name	Signal description	RT (min)	Area (mAU·s)	Area%	Height (mAU)	Height%	Amount	Concentration	Start time (min)	End time (min)
1		DAD1B,Sig=254,4 Ref=360,100	8.333	10927.730	50.034	1052.657	53.68			8.131	8.901
2		DAD1B,Sig=254,4 Ref=360,100	9.552	10912.724	49.966	908.253	46.32			9.294	10.264

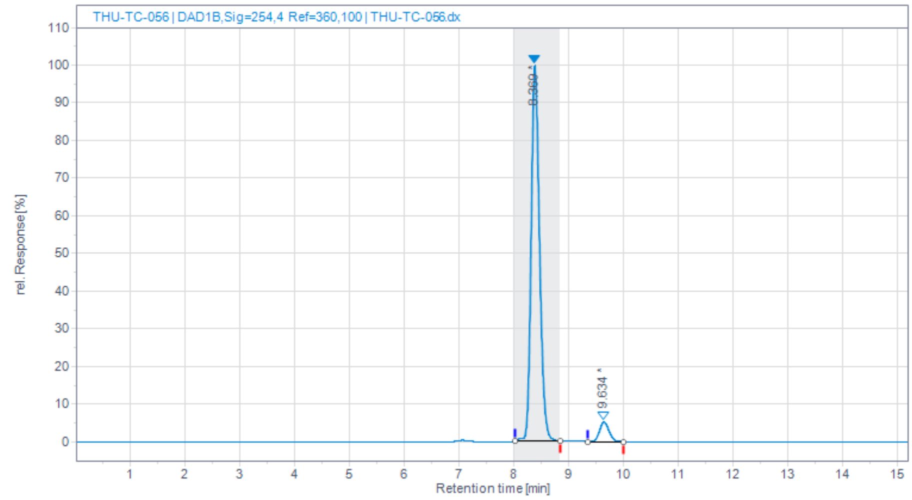

#	Name	Signal description	RT (min)	Area (mAU·s)	Area%	Height (mAU)	Height%	Amount	Concentration	Start time (min)	End time (min)
1		DAD1B,Sig=254,4 Ref=360,100	8.369	13329.046	94.436	1268.350	95.06			8.007	8.855
2		DAD1B,Sig=254,4 Ref=360,100	9.634	785.269	5.564	65.982	4.94			9.355	9.987

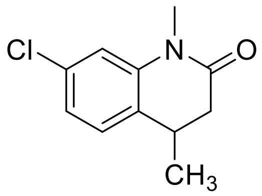

7-Chloro-1,4-dimethyl-3,4-dihydroquinolin-2(1*H*)-one (**2p**)

^{1}H NMR (400 MHz, $CDCl_3$) δ 7.10 (dd, J = 8.0, 0.9 Hz, 1H), 7.00 (dd, J = 8.0, 2.0 Hz, 1H), 6.96 (d, J = 2.0 Hz, 1H), 3.33 (s, 3H), 3.09 – 2.96 (m, 1H), 2.71 (dd, J = 15.9, 5.5 Hz, 1H), 2.43 (dd, J = 15.9, 7.7 Hz, 1H), 1.25 (d, J = 7.0 Hz, 3H); **^{13}C NMR** (101 MHz, $CDCl_3$) δ 169.7, 141.1, 133.1, 129.5, 127.3, 122.8, 115.2, 39.0, 30.0, 29.6, 19.3; **ESI-**

MS: calculated $[C_{11}H_{12}NOClNa]^+$:232.0500, found:232.0497; **HPLC** DAICEL CHIRALCEL OD-H, *n*-hexane/2-propanol = 95/5, flow rate = 1.0 mL/min, λ = 254 nm, retention time: 12.9 min (major), 14.5 min (minor).

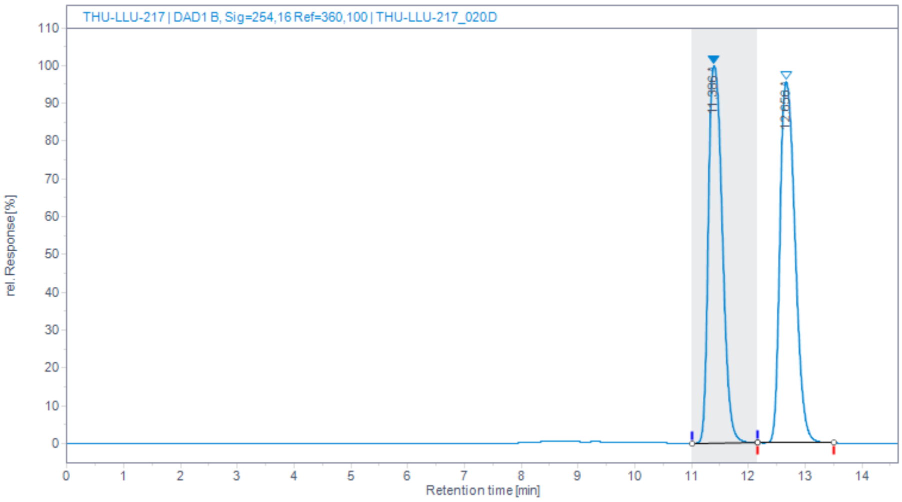

#	Name	Signal description	RT (min)	Area (mAU·s)	Area%	Height (mAU)	Height%	Amount	Concentration	Start time (min)	End time (min)
1		DAD1 B, Sig=254,16 Ref=360,100	11.386	31687.012	49.053	1856.978	51.12			11.018	12.152
2		DAD1 B, Sig=254,16 Ref=360,100	12.656	32909.966	50.947	1775.258	48.88			12.152	13.495

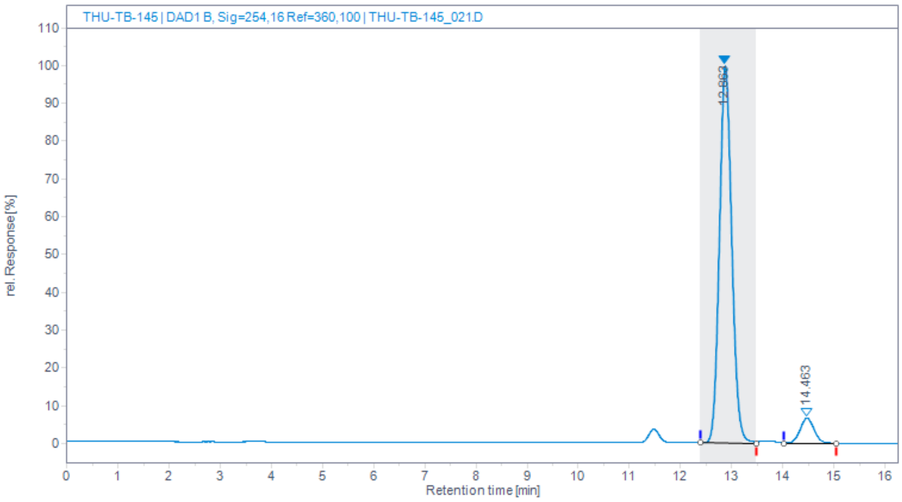

#	Name	Signal description	RT (min)	Area (mAU·s)	Area%	Height (mAU)	Height%	Amount	Concentration	Start time (min)	End time (min)
1		DAD1 B, Sig=254,16 Ref=360,100	12.863	3971.649	92.960	239.232	93.81			12.382	13.474
2		DAD1 B, Sig=254,16 Ref=360,100	14.463	300.771	7.040	15.792	6.19			14.031	15.041

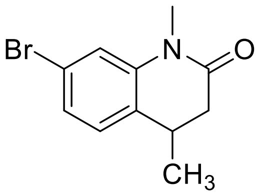

7-Bromo-1,4-dimethyl-3,4-dihydroquinolin-2(1*H*)-one (2q)

^{1}H NMR (400 MHz, $CDCl_3$) δ 7.16 (dd, *J* = 8.0, 1.9 Hz, 1H), 7.11 (d, *J* = 1.9 Hz, 1H), 7.05 (dd, *J* = 8.0, 0.9 Hz, 1H), 3.33 (s, 3H), 3.07 – 2.94 (m, 1H), 2.70 (dd, *J* = 15.9, 5.5 Hz, 1H), 2.43 (dd, *J* = 15.9, 7.6 Hz, 1H), 1.25 (d, *J* = 7.0 Hz, 3H); **^{13}C NMR** (101 MHz, $CDCl_3$) δ 169.7, 141.3, 130.0, 127.7, 125.8, 121.0, 118.0, 39.0, 30.1, 29.6, 19.3; **ESI-MS**: calculated $[C_{11}H_{12}NOBrOH]^-$:254.0175, found:254.0173; **HPLC** DAICEL CHIRALCEL OD-H, *n*-

hexane/2-propanol = 95/5, flow rate = 1.0 mL/min, λ = 254 nm, retention time: 9.3 min (major), 11.0 min (minor).

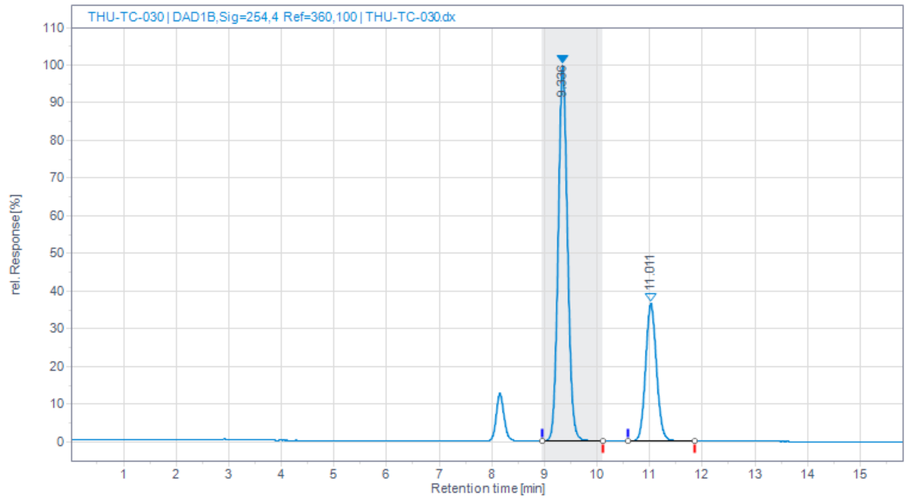

#	Name	Signal description	RT (min)	Area (mAU·s)	Area%	Height (mAU)	Height%	Amount	Concentration	Start time (min)	End time (min)
1		DAD1B,Sig=254,4 Ref=360,100	9.336	905.736	69.999	73.492	73.16			8.950	10.117
2		DAD1B,Sig=254,4 Ref=360,100	11.011	388.184	30.001	26.959	26.84			10.583	11.860

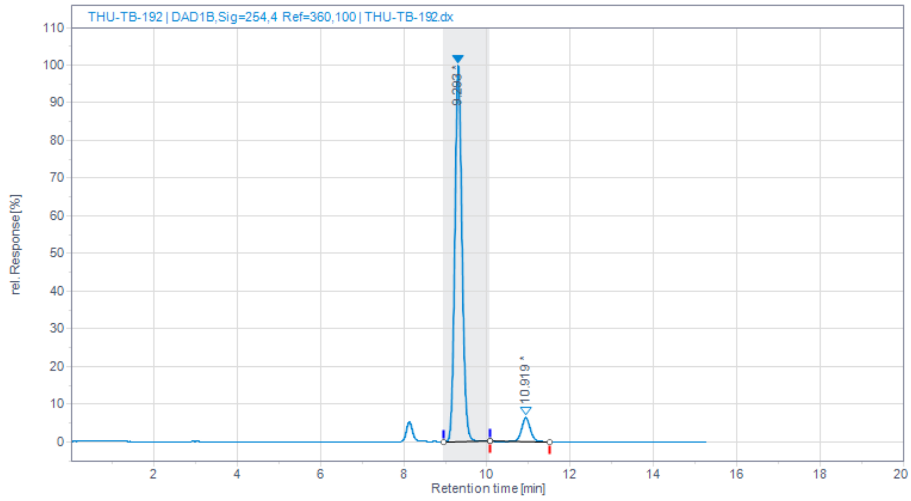

#	Name	Signal description	RT (min)	Area (mAU·s)	Area%	Height (mAU)	Height%	Amount	Concentration	Start time (min)	End time (min)
1		DAD1B,Sig=254,4 Ref=360,100	9.293	2250.138	92.994	188.560	93.97			8.954	10.059
2		DAD1B,Sig=254,4 Ref=360,100	10.919	169.516	7.006	12.097	6.03			10.059	11.491

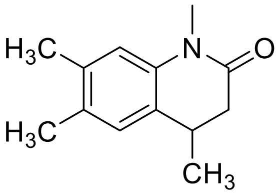

1,4,6,7-Tetramethyl-3,4-dihydroquinolin-2(1*H*)-one (**2r**)

^{1}H NMR (400 MHz, $CDCl_3$) δ 6.95 (s, 1H), 6.78 (s, 1H), 3.35 (s, 3H), 2.99 (q, *J* = 6.7 Hz, 1H), 2.70 (dd, *J* = 15.7, 5.4 Hz, 1H), 2.43 (dd, *J* = 15.8, 7.4 Hz, 1H), 2.25 (d, *J* = 14.5 Hz, 6H), 1.25 (d, *J* = 7.0 Hz, 3H); **^{13}C NMR** (101 MHz, $CDCl_3$) δ 170.0, 137.6, 135.5, 131.2, 128.4, 127.6, 116.4, 39.5, 30.0, 29.5, 19.9, 19.6, 19.1; **ESI-MS**: calculated $[C_{13}H_{17}NONa]^+$:226.1202, found:226.1190; **HPLC** DAICEL CHIRALCEL OD-H, *n*-hexane/2-propanol = 95/5, flow rate = 1.0 mL/min, λ = 254 nm, retention time: 10.2 min (major), 11.8 min (minor).

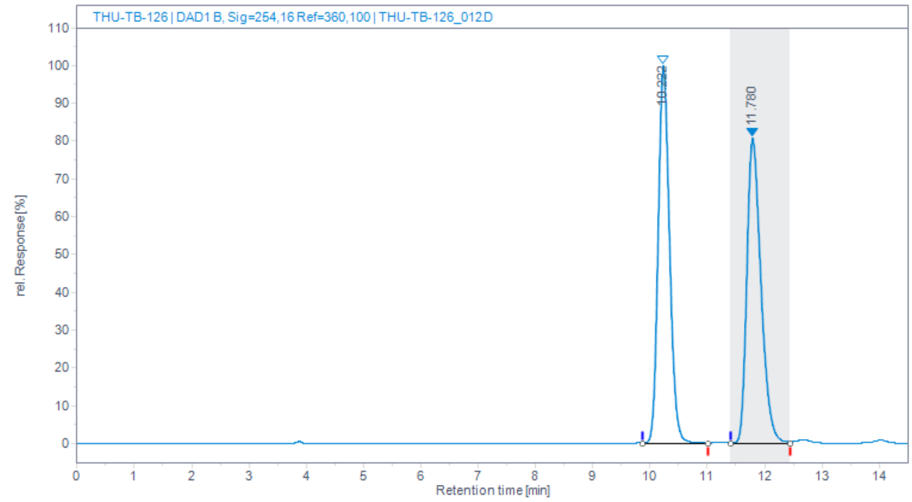

#	Name	Signal description	RT (min)	Area (mAU·s)	Area%	Height (mAU)	Height%	Amount	Concentration	Start time (min)	End time (min)
1		DAD1 B, Sig=254,16 Ref=360,100	10.222	9204.199	49.925	669.787	55.33			9.867	11.014
2		DAD1 B, Sig=254,16 Ref=360,100	11.780	9231.715	50.075	540.689	44.67			11.415	12.439

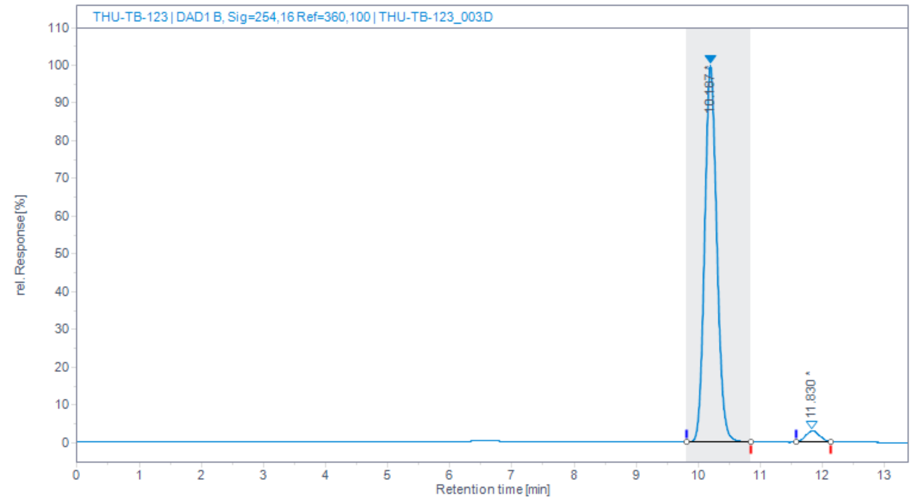

#	Name	Signal description	RT (min)	Area (mAU·s)	Area%	Height (mAU)	Height%	Amount	Concentration	Start time (min)	End time (min)
1		DAD1 B, Sig=254,16 Ref=360,100	10.187	2048.401	96.792	150.902	97.18			9.817	10.838
2		DAD1 B, Sig=254,16 Ref=360,100	11.830	67.898	3.208	4.376	2.82			11.567	12.123

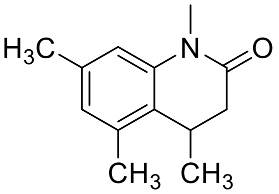

1,4,5,7-Tetramethyl-3,4-dihydroquinolin-2(1*H*)-one (**2s**)

^{1}H NMR (400 MHz, $CDCl_3$) δ 6.74 (dt, *J* = 1.6, 0.8 Hz, 1H), 6.71 – 6.68 (m, 1H), 3.36 (s, 3H), 3.24 – 3.12 (m, 1H), 2.69 (dd, *J* = 15.8, 6.0 Hz, 1H), 2.58 (dd, *J* = 15.8, 2.0 Hz, 1H), 2.31 (d, *J* = 9.4 Hz, 6H), 1.13 (d, *J* = 7.1 Hz, 3H); **^{13}C NMR** (101 MHz, $CDCl_3$) δ 169.6, 139.5, 136.7, 134.9, 126.4, 126.0, 114.1, 38.6, 29.8, 26.9, 21.4, 18.8, 18.3; **ESI-MS**: calculated $[C_{13}H_{17}NONa]^{+}$:226.1202, found:226.1194; **HPLC** DAICEL CHIRALCEL OD-H, *n*-hexane/2-propanol = 95/5, flow rate = 1.0 mL/min, λ = 254 nm, retention time: 11.0 min (major), 12.0 min (minor).

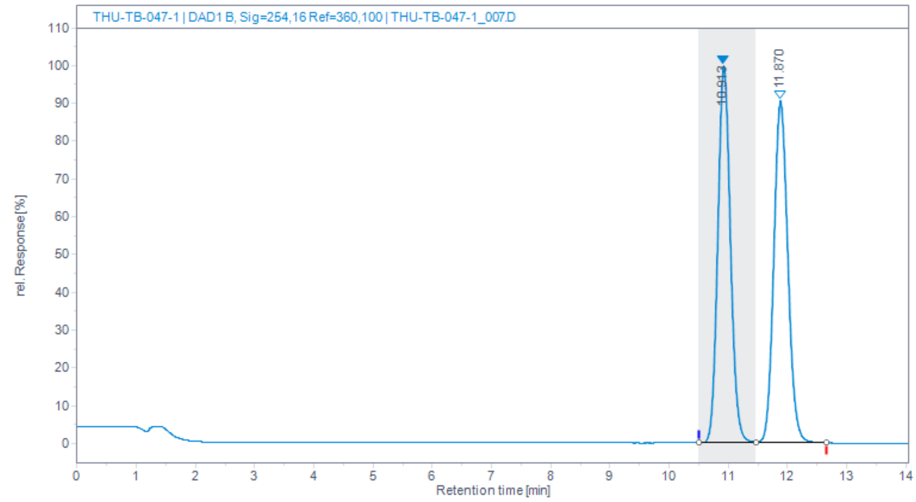

#	Name	Signal description	RT (min)	Area (mAU·s)	Area%	Height (mAU)	Height%	Amount	Concentration	Start time (min)	End time (min)
1		DAD1 B, Sig=254,16 Ref=360,100	10.913	1435.440	49.894	96.639	52.45			10.505	11.458
2		DAD1 B, Sig=254,16 Ref=360,100	11.870	1441.562	50.106	87.602	47.55			11.458	12.661

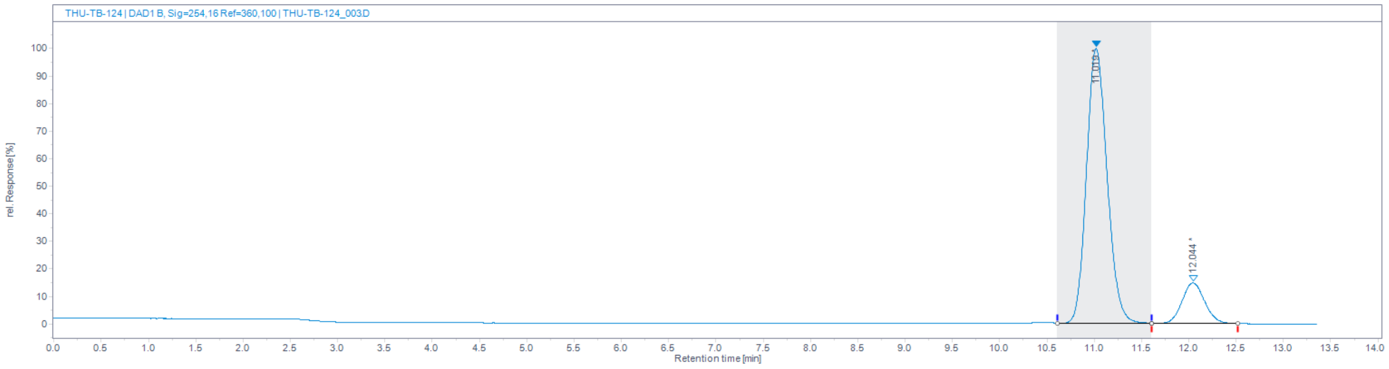

#	Name	Signal description	RT (min)	Area (mAU·s)	Area%	Height (mAU)	Height%	Amount	Concentration	Start time (min)	End time (min)
1		DAD1 B, Sig=254,16 Ref=360,100	11.019	4201.920	86.140	277.309	87.14			10.605	11.612
2		DAD1 B, Sig=254,16 Ref=360,100	12.044	676.075	13.860	40.930	12.86			11.612	12.522

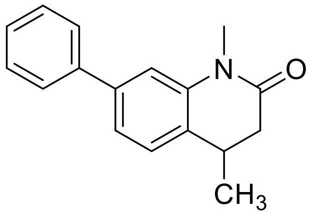

1,4-Dimethyl-7-phenyl-3,4-dihydroquinolin-2(1*H*)-one (2t)

^{1}H NMR (400 MHz, $CDCl_3$) δ 7.61 – 7.53 (m, 2H), 7.45 (t, *J* = 7.6 Hz, 2H), 7.41 – 7.32 (m, 1H), 7.26 (s, 2H), 7.18 (s, 1H), 3.43 (s, 3H), 3.10 (td, *J* = 7.4, 5.6 Hz, 1H), 2.76 (dd, *J* = 15.8, 5.4 Hz, 1H), 2.48 (dd, *J* = 15.8, 7.7 Hz, 1H), 1.32 (d, *J* = 7.0 Hz, 3H); **^{13}C NMR** (101 MHz, $CDCl_3$) δ 170.1, 140.9, 140.9, 140.4, 130.2, 129.0, 127.6, 127.2, 126.7, 121.9, 113.9, 39.3, 30.1, 29.6, 19.4; **ESI-MS**: calculated $[C_{17}H_{17}NONa]^+$:274.1202, found:274.1201; **HPLC** DAICEL CHIRALCEL OD-H, *n*-hexane/2-propanol = 95/5, flow rate = 1.0 mL/min, λ = 254 nm, retention time: 16.7 min (major), 18.5 min (minor).

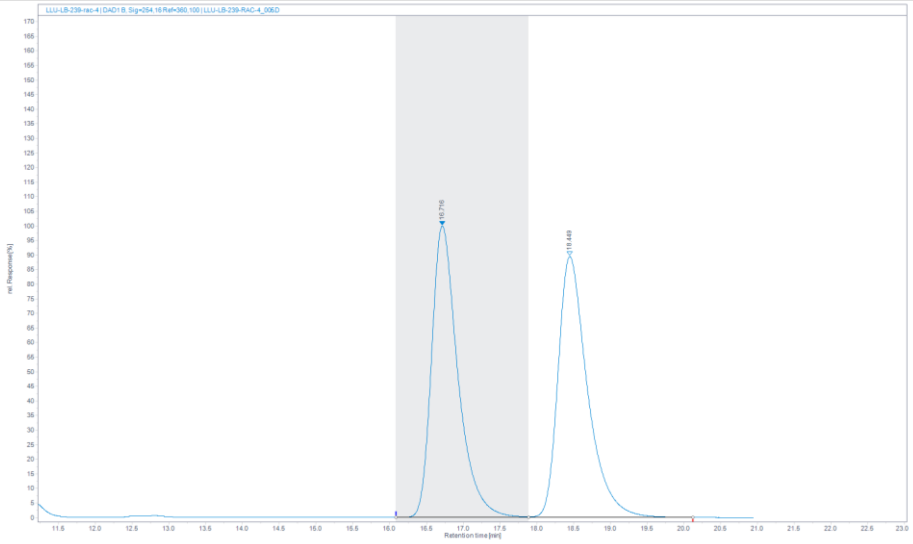

#	Name	Signal description	RT (min)	Area (mAU·s)	Area%	Height (mAU)	Height%	Amount	Concentration	Start time (min)	End time (min)
1		DAD1 B, Sig=254,16 Ref=360,100	16.716	13102.862	49.948	527.534	52.77			16.088	17.893
2		DAD1 B, Sig=254,16 Ref=360,100	18.449	13130.406	50.052	472.072	47.23			17.893	20.119

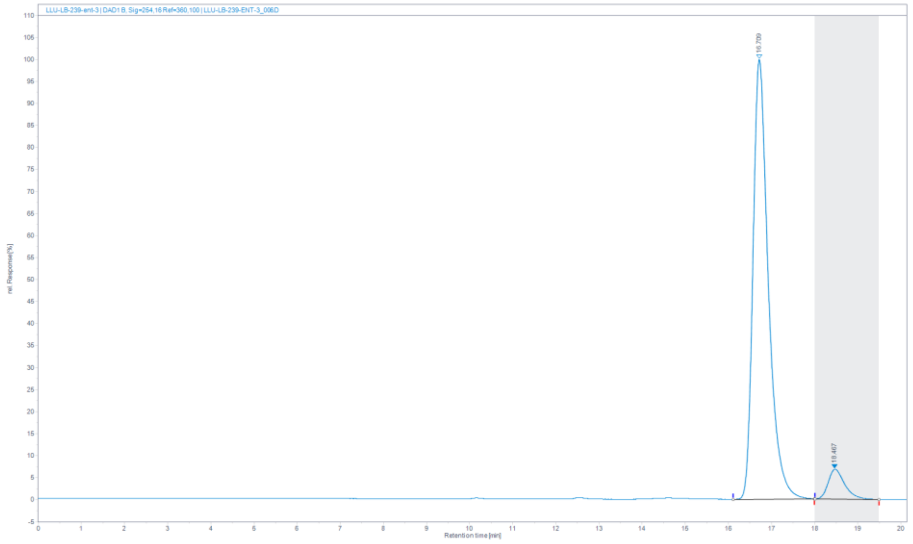

#	Name	Signal description	RT (min)	Area (mAU·s)	Area%	Height (mAU)	Height%	Amount	Concentration	Start time (min)	End time (min)
1		DAD1 B, Sig=254,16 Ref=360,100	16.709	6952.506	93.175	282.031	93.67			16.113	17.998
2		DAD1 B, Sig=254,16 Ref=360,100	18.467	509.230	6.825	19.072	6.33			18.007	19.490

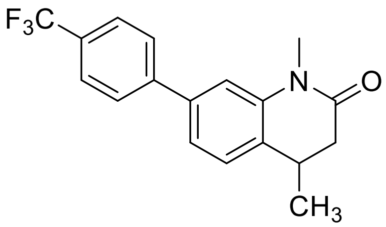

1,4-Dimethyl-7-(4-(trifluoromethyl)phenyl)-3,4-dihydroquinolin-2(1*H*)-one (2u)

^{1}H NMR (400 MHz, $CDCl_3$) δ 7.78 – 7.64 (m, 4H), 7.33 – 7.24 (m, 3H), 7.17 (d, *J* = 1.6 Hz, 1H), 3.44 (s, 3H), 3.12 (td, *J* = 7.3, 5.5 Hz, 1H), 2.77 (dd, *J* = 15.9, 5.5 Hz, 1H), 2.50 (dd, *J* = 15.9, 7.6 Hz, 1H), 1.33 (d, *J* = 7.0 Hz, 3H); **^{13}C NMR** (101 MHz, $CDCl_3$) δ 170.0, 144.5 (d, *J* = 1.8 Hz), 140.7, 139.5, 131.2, 129.8 (q, *J* = 32.6 Hz), 127.6, 127.0, 124.4 (q, *J* = 272.7 Hz), 126.0 (q, *J* = 3.9 Hz), 122.1, 113.9, 39.2, 30.3, 29.7, 19.4; **^{19}F NMR** (377 MHz, $CDCl_3$) δ -62.43; **ESI-MS**: calculated $[C_{18}H_{16}NOF_3Na]^+$:342.1076,

found:342.1076; **Chiral GC-FID**(50_5_80_0.5_220, β-Dex): retention time: 283.6 min (major), 286.0 min (minor)

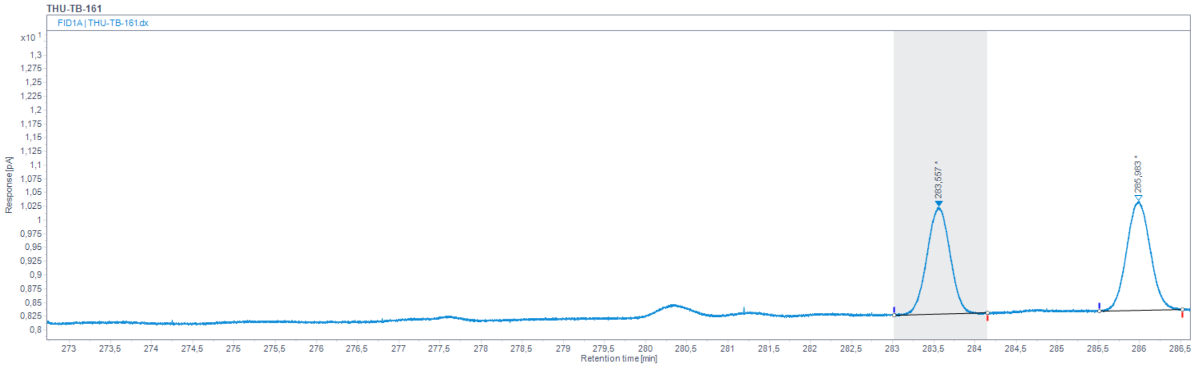

#	Name	Signal description	RT (min)	Area	Area%	Height	Height%	Amount	Concentration	Start time (min)	End time (min)
1		FID1A	283,557	37,308	48,846	1,930	49,55			283,022	284,148
2		FID1A	285,983	39,071	51,154	1,965	50,45			285,506	286,515

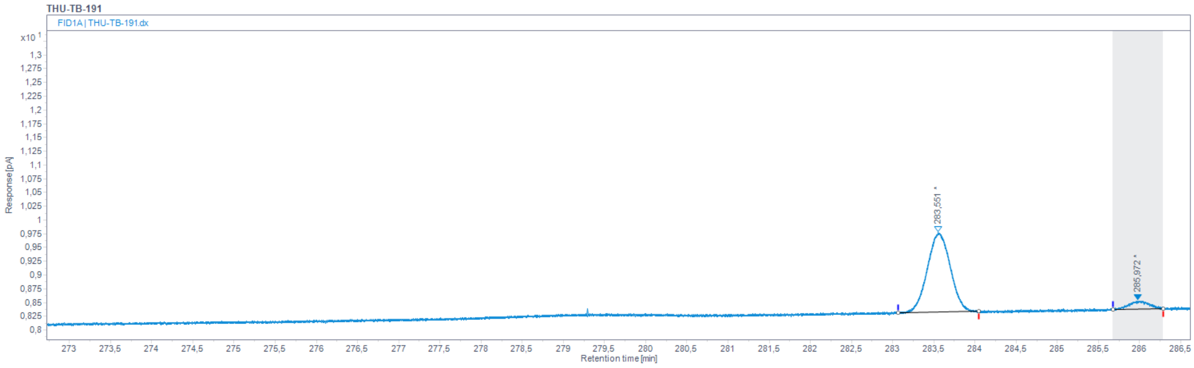

#	Name	Signal description	RT (min)	Area	Area%	Height	Height%	Amount	Concentration	Start time (min)	End time (min)
1		FID1A	283,551	27,676	92,084	1,430	91,16			283,071	284,041
2		FID1A	285,972	2,379	7,916	0,139	8,84			285,680	286,292

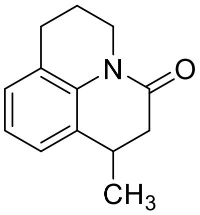

7-Methyl-2,3,6,7-tetrahydro-1H,5H-pyrido[3,2,1-ij]quinolin-5-one (**2v**)

^{1}H NMR (400 MHz, $CDCl_3$) δ 7.06 – 6.98 (m, 2H), 6.97 – 6.89 (m, 1H), 4.05 – 3.76 (m, 2H), 3.13 – 2.95 (m, 1H), 2.80 (t, J = 6.3 Hz, 2H), 2.75 – 2.66 (m, 1H), 2.45 (dd, J = 15.8, 7.5 Hz, 1H), 2.01 – 1.88 (m, 2H), 1.27 (d, J = 7.0 Hz, 3H); **^{13}C NMR** (101 MHz, $CDCl_3$) δ 169.2, 135.4, 130.6, 127.9, 125.5, 124.4, 122.8, 40.9, 39.1, 30.4, 27.6, 21.6, 19.7; **ESI-MS**: calculated $[C_{13}H_{15}NONa]^+$:224.1046, found:224.1044; **HPLC** DAICEL CHIRALCEL OD-H, *n*-hexane/2-propanol = 95/5, flow rate = 1.0 mL/min, λ = 254 nm, retention time: 8.4 min (major), 9.2 min (minor).

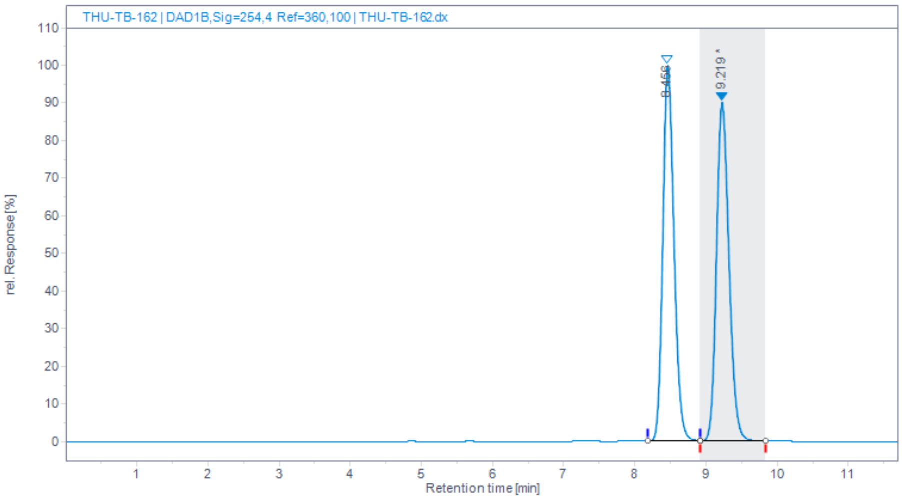

#	Name	Signal description	RT (min)	Area (mAU·s)	Area%	Height (mAU)	Height%	Amount	Concentration	Start time (min)	End time (min)
1		DAD1B,Sig=254,4 Ref=360,100	8.456	7247.040	49.957	660.487	52.59			8.189	8.923
2		DAD1B,Sig=254,4 Ref=360,100	9.219	7259.432	50.043	595.423	47.41			8.923	9.830

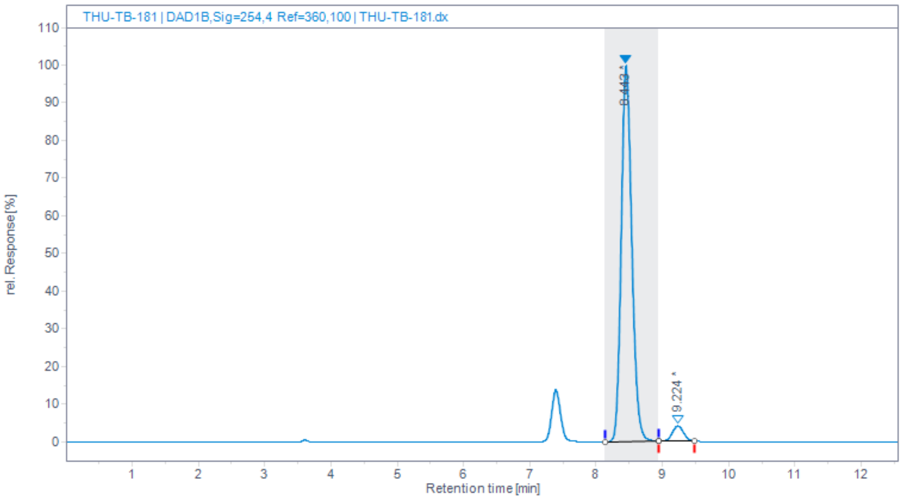

#	Name	Signal description	RT (min)	Area (mAU·s)	Area%	Height (mAU)	Height%	Amount	Concentration	Start time (min)	End time (min)
1		DAD1B,Sig=254,4 Ref=360,100	8.443	6986.183	95.906	636.003	96.11			8.132	8.936
2		DAD1B,Sig=254,4 Ref=360,100	9.224	298.227	4.094	25.733	3.89			8.936	9.475

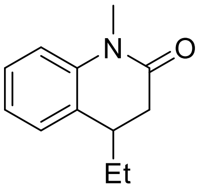

4-Ethyl-1-methyl-3,4-dihydroquinolin-2(1*H*)-one (**2w**)

^{1}H NMR (400 MHz, $CDCl_3$) δ 7.25 (td, *J* = 7.8, 1.6 Hz, 1H), 7.14 (dd, *J* = 7.4, 1.6 Hz, 1H), 7.06 – 6.95 (m, 2H), 3.35 (s, 3H), 2.79 – 2.70 (m, 2H), 2.66 – 2.56 (m, 1H), 1.58 (qt, *J* = 13.9, 7.0 Hz, 2H), 0.92 (t, *J* = 7.4 Hz, 3H); **^{13}C NMR** (101 MHz, $CDCl_3$) δ 169.9, 139.8, 129.8, 127.8, 127.5, 122.8, 115.0, 37.7, 36.7, 29.4, 26.6, 11.6; **ESI-MS**: calculated $[C_{12}H_{15}NONa]^+$:212.1046, found:212.1043; **HPLC** DAICEL CHIRALCEL OD-H, *n*-hexane/2-propanol = 95/5, flow rate = 1.0 mL/min, λ = 254 nm, retention time: 10.3 min (major), 11.6 min (minor).

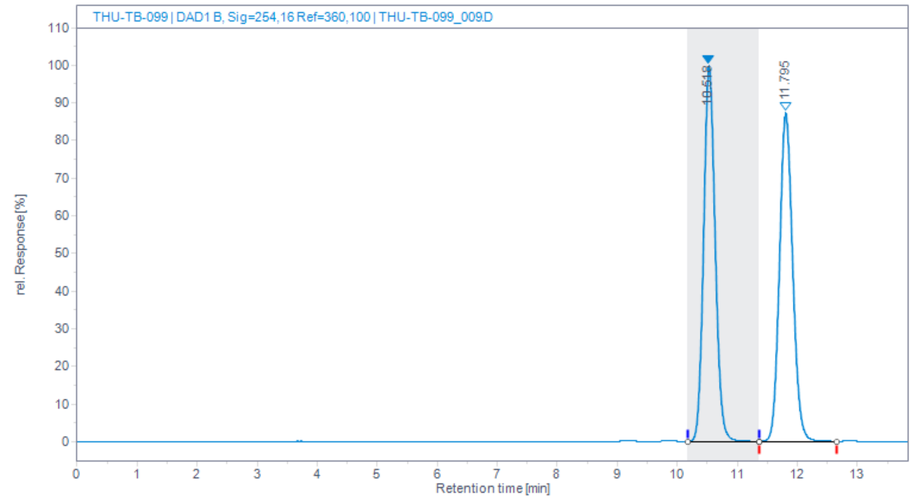

#	Name	Signal description	RT (min)	Area (mAU·s)	Area%	Height (mAU)	Height%	Amount	Concentration	Start time (min)	End time (min)
1		DAD1 B, Sig=254,16 Ref=360,100	10.518	5094.660	50.061	380.708	53.36			10.172	11.369
2		DAD1 B, Sig=254,16 Ref=360,100	11.795	5082.207	49.939	332.729	46.64			11.370	12.648

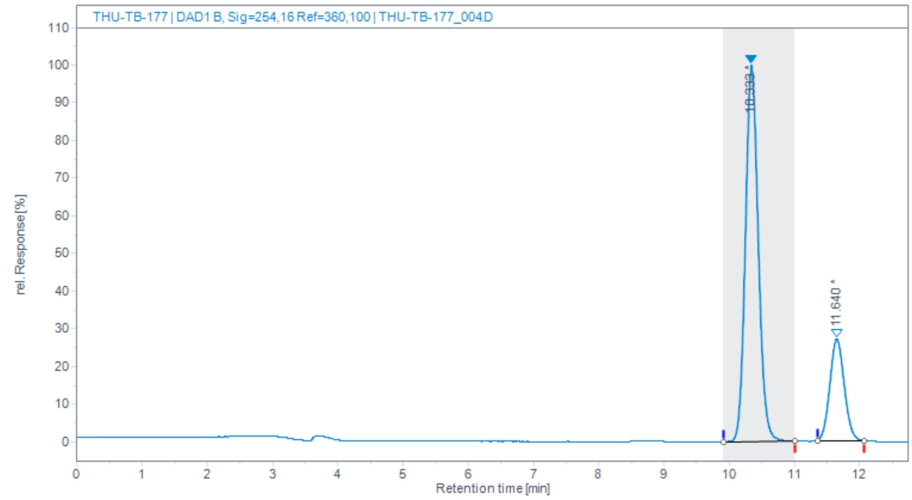

#	Name	Signal description	RT (min)	Area (mAU·s)	Area%	Height (mAU)	Height%	Amount	Concentration	Start time (min)	End time (min)
1		DAD1 B, Sig=254,16 Ref=360,100	10.333	2614.702	76.881	190.571	78.75			9.905	10.999
2		DAD1 B, Sig=254,16 Ref=360,100	11.640	786.281	23.119	51.434	21.25			11.347	12.059

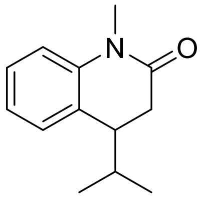

4-Isopropyl-1-methyl-3,4-dihydroquinolin-2(1*H*)-one (**2x**)

^{1}H NMR (400 MHz, $CDCl_3$) δ 7.24 (td, *J* = 7.7, 1.6 Hz, 1H), 7.11 (dd, *J* = 7.5, 1.6 Hz, 1H), 7.00 (d, *J* = 1.1 Hz, 2H), 3.32 (s, 3H), 2.77 (dd, *J* = 16.0, 3.1 Hz, 1H), 2.66 (dd, *J* = 16.0, 6.1 Hz, 1H), 2.54 (ddd, *J* = 7.4, 6.0, 3.1 Hz, 1H), 1.79 (dq, *J* = 13.7, 6.9 Hz, 1H), 0.93 (d, *J* = 6.7 Hz, 3H), 0.84 (d, *J* = 6.7 Hz, 3H); **^{13}C NMR** (101 MHz, $CDCl_3$) δ 170.2, 140.2, 129.1, 129.0, 127.5, 122.6, 115.0, 43.0, 34.7, 30.8, 29.4, 20.9, 19.6; **ESI-MS**: calculated $[C_{13}H_{17}NONa]^+$:226.1202, found:226.1196; **HPLC** DAICEL CHIRALCEL OD-H, *n*-hexane/2-propanol = 95/5, flow rate = 1.0 mL/min, λ = 254 nm, retention time: 6.5 min (major), 7.4 min (minor).

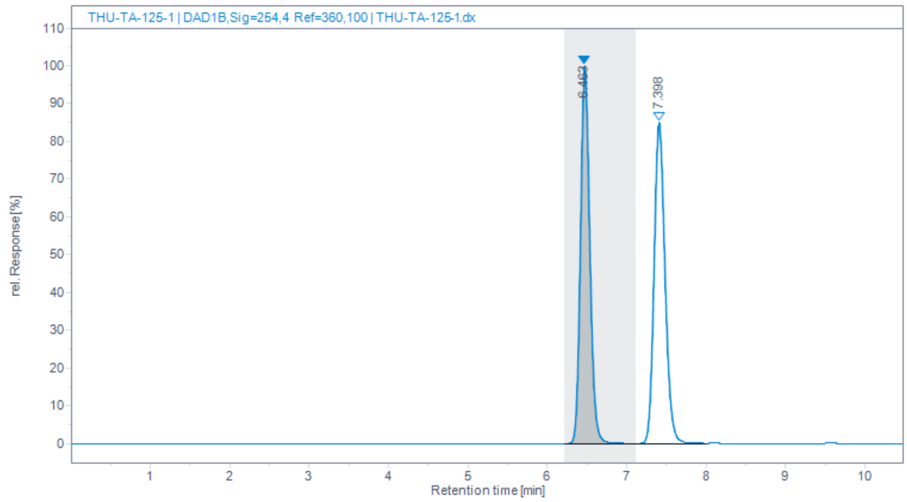

#	Name	Signal description	RT (min)	Area (mAU·s)	Area%	Height (mAU)	Height%	Amount	Concentration	Start time (min)	End time (min)
1		DAD1B,Sig=254,4 Ref=360,100	6.463	3966.135	49.943	490.243	54.05			6.214	7.121
2		DAD1B,Sig=254,4 Ref=360,100	7.398	3975.117	50.057	416.759	45.95			7.121	7.997

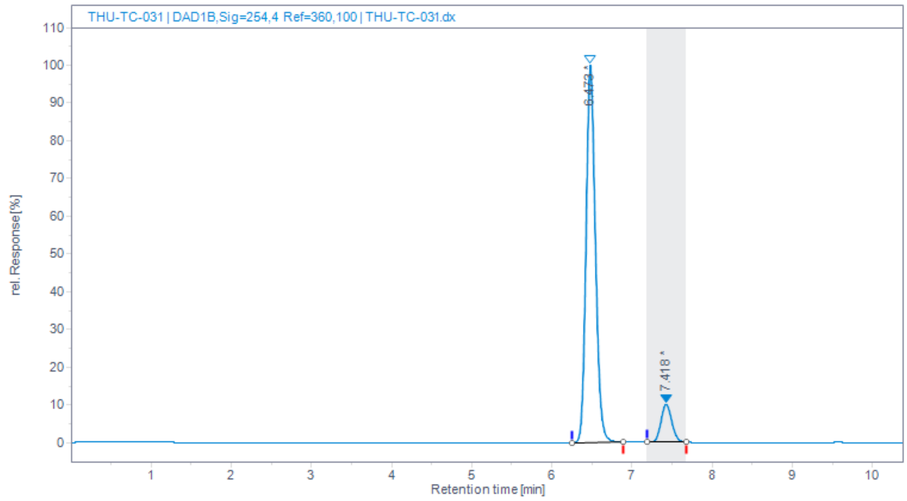

#	Name	Signal description	RT (min)	Area (mAU·s)	Area%	Height (mAU)	Height%	Amount	Concentration	Start time (min)	End time (min)
1		DAD1B,Sig=254,4 Ref=360,100	6.473	3866.486	89.550	478.774	90.86			6.245	6.883
2		DAD1B,Sig=254,4 Ref=360,100	7.418	451.195	10.450	48.169	9.14			7.188	7.670

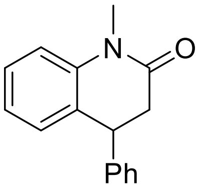

1-Methyl-4-phenyl-3,4-dihydroquinolin-2(1*H*)-one (**2y**)

^{1}H NMR (400 MHz, $CDCl_3$) δ 7.34 – 7.18 (m, 4H), 7.16 – 7.08 (m, 2H), 7.02 (dd, *J* = 8.1, 1.1 Hz, 1H), 6.96 (td, *J* = 7.4, 1.1 Hz, 1H), 6.88 (dt, *J* = 7.5, 1.4 Hz, 1H), 4.19 (t, *J* = 7.4 Hz, 1H), 3.36 (s, 3H), 2.93 (dd, *J* = 7.4, 2.3 Hz, 2H); **^{13}C NMR** (101 MHz, $CDCl_3$) δ 169.5, 141.2, 140.5, 129.3, 129.0, 128.2, 128.0, 127.9, 127.3, 123.2, 115.0, 41.6, 39.0, 29.7; **ESI-MS**: calculated $[C_{16}H_{15}NONa]^+$:260.1046, found:260.1043; **HPLC** DAICEL CHIRALCEL OD-H, *n*-hexane/2-propanol = 95/5, flow rate = 1.0 mL/min, λ = 254 nm, retention time: 13.1 min (major), 14.4 min (minor).

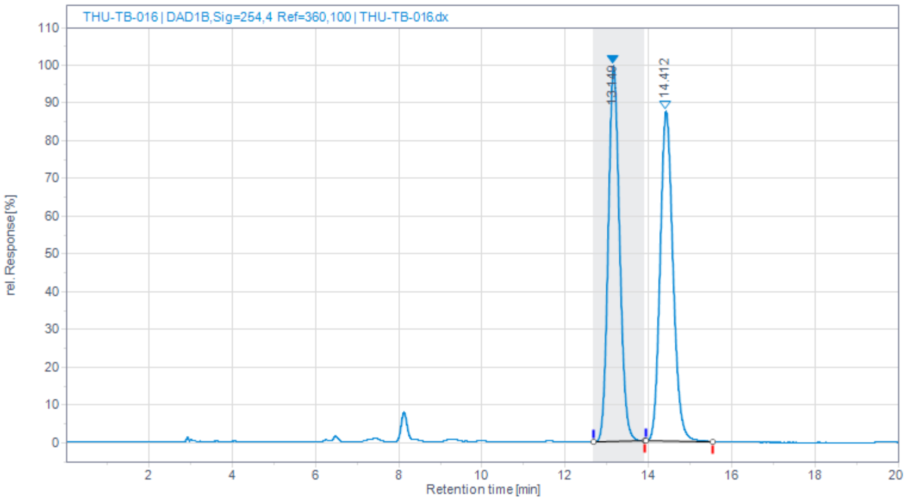

#	Name	Signal description	RT (min)	Area (mAU·s)	Area%	Height (mAU)	Height%	Amount	Concentration	Start time (min)	End time (min)
1		DAD1B,Sig=254,4 Ref=360,100	13.149	381.284	50.109	20.144	53.23			12.681	13.920
2		DAD1B,Sig=254,4 Ref=360,100	14.412	379.624	49.891	17.702	46.77			13.944	15.544

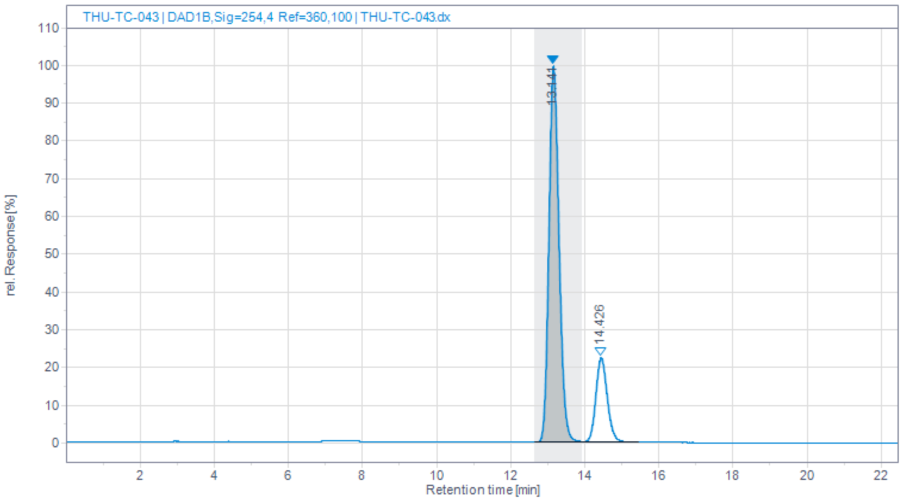

#	Name	Signal description	RT (min)	Area (mAU·s)	Area%	Height (mAU)	Height%	Amount	Concentration	Start time (min)	End time (min)
1		DAD1B,Sig=254,4 Ref=360,100	13.141	1815.428	79.962	95.996	81.74			12.657	13.939
2		DAD1B,Sig=254,4 Ref=360,100	14.426	454.929	20.038	21.449	18.26			13.940	15.460

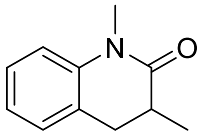

1,3-Dimethyl-3,4-dihydroquinolin-2(1*H*)-one (**2z**)

^{1}H NMR (400 MHz, $CDCl_3$) δ 7.29 – 7.21 (m, 1H), 7.16 (dd, *J* = 7.5, 1.6 Hz, 1H), 7.04 – 6.94 (m, 2H), 3.36 (s, 3H), 2.93 (dd, *J* = 14.5, 4.9 Hz, 1H), 2.78 – 2.55 (m, 2H), 1.26 (d, *J* = 6.6 Hz, 3H); **^{13}C NMR** (101 MHz, $CDCl_3$) δ 173.4, 140.6, 128.0, 127.5, 125.9, 122.8, 114.6, 35.6, 33.5, 29.9, 15.8; **ESI-MS**: calculated $[C_{11}H_{13}NONa]^+$:198.0889, found:198.0888; **HPLC** DAICEL CHIRALCEL AD-H, *n*-hexane/2-propanol = 95/5, flow rate = 1.0 mL/min, λ = 254 nm, retention time: 8.3 min (major), 8.8 min (minor); n-hexane/2-propanol = 97/3, flow rate = 1.0 mL/min, λ = 254 nm, retention time: 10.1 min (minor), 10.7 min (major).

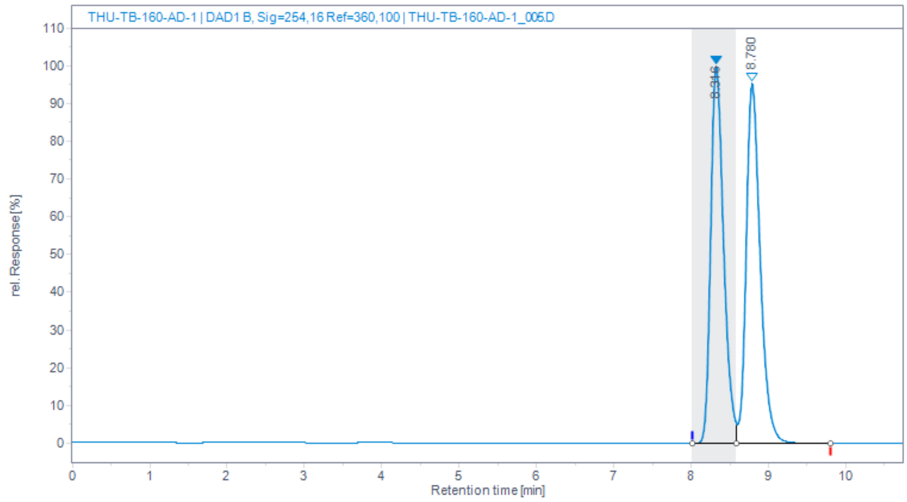

#	Name	Signal description	RT (min)	Area (mAU·s)	Area%	Height (mAU)	Height%	Amount	Concentration	Start time (min)	End time (min)
1		DAD1 B, Sig=254,16 Ref=360,100	8.316	14990.330	49.289	1291.619	51.21			8.019	8.592
2		DAD1 B, Sig=254,16 Ref=360,100	8.780	15422.500	50.711	1230.377	48.79			8.592	9.806

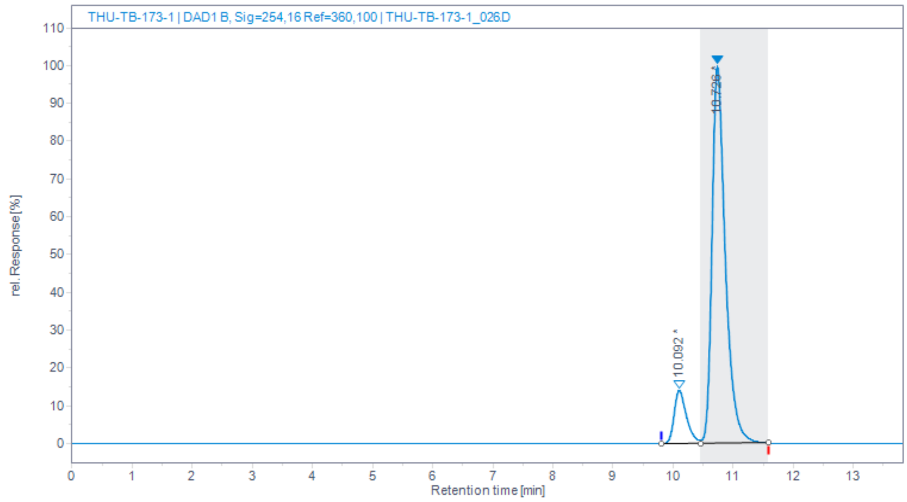

#	Name	Signal description	RT (min)	Area (mAU·s)	Area%	Height (mAU)	Height%	Amount	Concentration	Start time (min)	End time (min)
1		DAD1 B, Sig=254,16 Ref=360,100	10.092	657.130	11.467	46.577	12.38			9.802	10.451
2		DAD1 B, Sig=254,16 Ref=360,100	10.726	5073.698	88.533	329.796	87.62			10.451	11.578

4. Transformations

a)

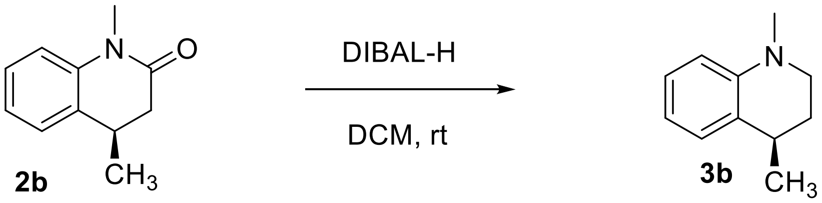

Amide **2b** (17.5 mg, 0.1 mmol, 1 equiv.) was dissolved in dry DCM (1 mL) in the Schlenk tube. Then DIBAL-H (0.4 mmol, 4 equiv.) was dropped in the solution.[5] The mixture was stirred 24 hours under room temperature. Et_2O (20 mL) was added into the mixture. After 30 minutes, the mixture was quenched with 2 M NaOH aqueous solution. The organic layer was collected and dried with $MgSO_4$.

After removing the solvent, the residue was purified by flash chromatography on silica gel to afford **3b** (9 mg, 58 % yield, 94:6 er).

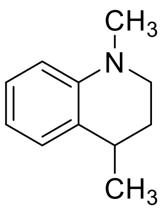

1,4-Dimethyl-1,2,3,4-tetrahydroquinoline (**3b**)
^{1}H NMR (300 MHz, $CDCl_3$) δ 7.15 – 7.03 (m, 2H), 6.70 – 6.59 (m, 2H), 3.31 – 3.15 (m, 2H), 2.95 – 2.85 (m, 4H), 2.05 (ddt, *J* = 13.2, 8.4, 4.9 Hz, 1H), 1.70 (dtd, *J* = 13.0, 6.3, 4.0 Hz, 1H), 1.29 (d, *J* = 7.0 Hz, 3H); **^{13}C NMR** (76 MHz, $CDCl_3$) δ 146.0, 128.0, 127.2, 116.5, 111.2, 48.4, 39.4, 30.9, 30.0, 22.8; **HPLC** DAICEL CHIRALCEL OD-H, *n*-hexane/2-propanol = 95/5, flow rate = 1.0 mL/min, λ = 254 nm, retention time: 4.0 min (major), 4.3 min (minor).

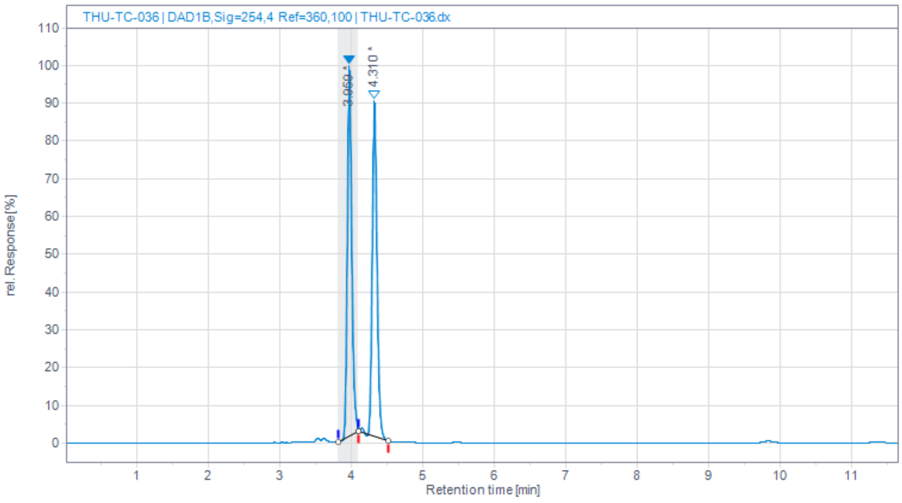

#	Name	Signal description	RT (min)	Area (mAU·s)	Area%	Height (mAU)	Height%	Amount	Concentration	Start time (min)	End time (min)
1		DAD1B,Sig=254,4 Ref=360,100	3.959	1538.449	50.381	365.651	52.48			3.816	4.088
2		DAD1B,Sig=254,4 Ref=360,100	4.310	1515.151	49.619	331.111	47.52			4.088	4.504

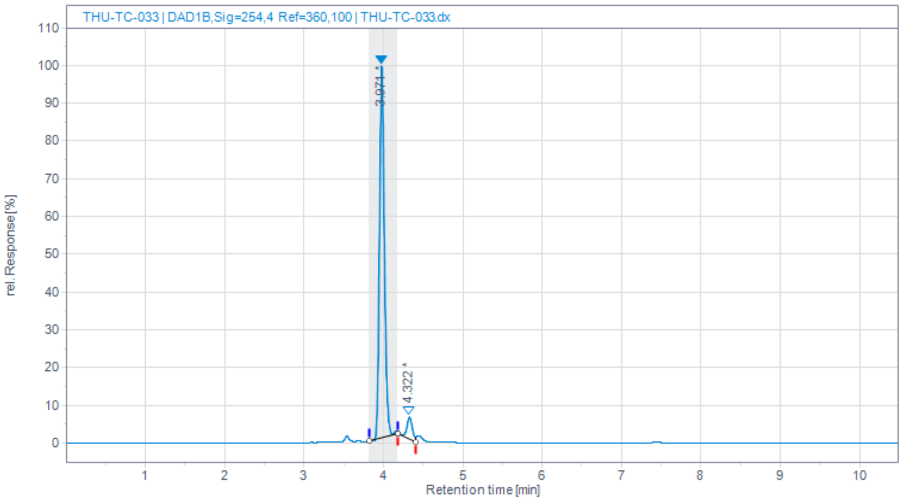

#	Name	Signal description	RT (min)	Area (mAU·s)	Area%	Height (mAU)	Height%	Amount	Concentration	Start time (min)	End time (min)
1		DAD1B,Sig=254,4 Ref=360,100	3.971	4595.574	93.796	1089.098	94.34			3.825	4.174
2		DAD1B,Sig=254,4 Ref=360,100	4.322	303.973	6.204	65.399	5.66			4.174	4.400

b)

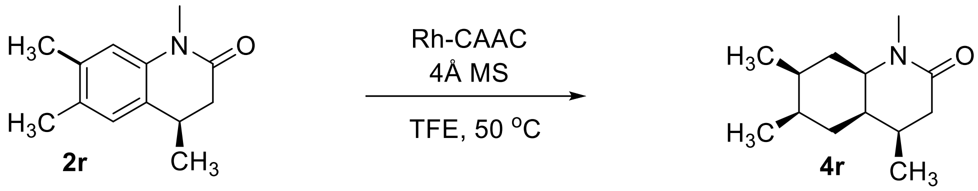

2r (33 mg, 0.16 mmol, 1.0 equiv.), Rh-CAAC (4.6 mg, 8 µmol, 5 mol%) and 4Å molecular sieves (50 mg) were added to a screw-cap tube and TFE was added (1.5 mL).[6] The glass vial was placed in a 150 mL stainless steel autoclave. The autoclave was pressurized and depressurized with hydrogen gas before the indicated pressure was set. The reaction mixture was stirred for 18 h at 50 °C. After the autoclave was depressurized, the mixture was purified by column chromatography (pentane:EtOAC = 1:1) to afford the desire product **4r** as a colourless liquid (24 mg, 0.11 mmol, 72%).

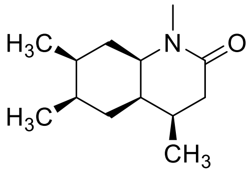

(*4R*,*4aR*,*6R*,*7S*,*8aR*)-1,4,6,7-tetramethyloctahydroquinolin-2(1*H*)-one (**4r**)
^{1}H NMR (300 MHz, $CDCl_3$) δ 3.47 – 3.37 (m, 1H), 2.92 (s, 3H), 2.35 – 2.09 (m, 4H), 1.86 – 1.73 (m, 2H), 1.69 – 1.52 (m, 2H), 1.30 – 1.10 (m, 2H), 1.00 – 0.77 (m, 9H); **^{13}C-NMR** (101 MHz, $CDCl_3$): δ 171.0, 57.9, 40.7, 36.5, 35.1, 34.6, 32.0, 30.6, 25.7, 22.4, 20.0, 18.2, 12.2; **ESI-MS**: calc. for $C_{13}H_{23}NONa$ $[M+Na]^+$ 232.1672, found 232.1668; **Chiral GC-FID** (50_5_90_0.05_110_20_220, β-Dex), retention time: 174.4 min (major), 183.5 min (minor).

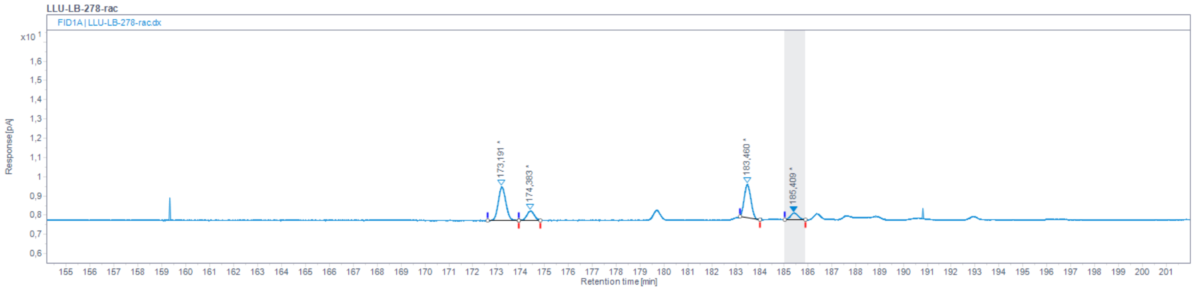

#	Name	Signal description	RT (min)	Area	Area%	Height	Height%	Amount	Concentration	Start time (min)	End time (min)
1		FID1A	173,191	37,127	42,067	1,743	40,22			172,617	173,919
2		FID1A	174,383	9,882	11,197	0,485	11,19			173,919	174,805
3		FID1A	183,460	33,670	38,151	1,747	40,32			183,153	183,988
4		FID1A	185,409	7,576	8,585	0,358	8,27			185,056	185,891

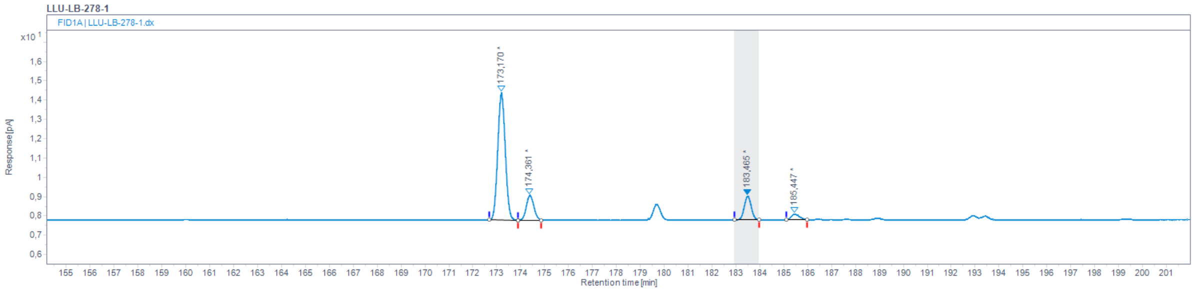

#	Name	Signal description	RT (min)	Area	Area%	Height	Height%	Amount	Concentration	Start time (min)	End time (min)
1		FID1A	173,170	143,629	70,587	6,601	70,05			172,691	173,904
2		FID1A	174,361	28,037	13,779	1,292	13,71			173,904	174,872
3		FID1A	183,465	24,741	12,159	1,230	13,06			182,943	183,955
4		FID1A	185,447	7,073	3,476	0,299	3,18			185,106	185,986

5. X-Ray Analysis

X-Ray diffraction: Data sets for compound **2v** were collected with a Bruker D8 Venture PHOTON III diffractometer. Programs used: data collection: APEX3 V2019.1-0[7] (Bruker AXS Inc., **2019**); cell refinement: SAINT V8.40A (Bruker AXS Inc., **2019**); data reduction: SAINT V8.40A (Bruker AXS Inc., **2019**); absorption correction, SADABS V2016/2 (Bruker AXS Inc., **2019**); structure solution *SHELXT-2015*[8] (Sheldrick, G. M. *Acta Cryst.,* **2015**, *A71*, 3-8); structure refinement *SHELXL-2015*[9] (Sheldrick, G. M. *Acta Cryst.,* **2015**, *C71* (1), 3-8) and graphics, *XP*[10] (Version 5.1, Bruker AXS Inc., Madison, Wisconsin, USA, **1998**). *R*-values are given for observed reflections, and wR^2 values are given for all reflections.

X-Ray crystal structure analysis of 2v: A colorless plate-like specimen of $C_{13}H_{15}NO$, approximate dimensions 0.032 mm x 0.104 mm x 0.156 mm, was used for the X-ray crystallographic analysis. The X-ray intensity data were measured on a single crystal Bruker D8 Venture Photon III Diffractometer system equipped with a micro focus tube CuKα (CuKα, λ = 1.54178 Å) and a MX mirror monochromator. A total of 1491 frames were collected. The total exposure time was 20.89 hours. The frames were integrated with the Bruker SAINT software package using a wide-frame algorithm. The integration of the data using a monoclinic unit cell yielded a total of 8868 reflections to a maximum θ angle of 66.56° (0.84 Å resolution), of which 1795 were independent (average redundancy 4.940, completeness = 99.2%, R_{int} = 4.31%, R_{sig} = 3.18%) and 1678 (93.48%) were greater than $2\sigma(F^2)$. The final cell constants of a = 8.5060(3) Å, b = 5.2936(2) Å, c = 12.0994(4) Å, β = 107.629(2)°, volume = 519.22(3) Å^3, are based upon the refinement of the XYZ-centroids of 5884 reflections above 20 σ(I) with 7.666° < 2θ < 133.1°. Data were corrected for absorption effects using the Multi-Scan method (SADABS). The ratio of minimum to maximum apparent transmission was 0.884. The calculated minimum and maximum transmission coefficients (based on crystal size) are 0.9070 and 0.9800. The structure was solved and refined using the Bruker SHELXTL Software Package, using the space

group $P2_1$, with Z = 2 for the formula unit, $C_{13}H_{15}NO$. The final anisotropic full-matrix least-squares refinement on F^2 with 137 variables converged at R1 = 3.84%, for the observed data and wR2 = 9.17% for all data. The goodness-of-fit was 1.071. The largest peak in the final difference electron density synthesis was 0.265 $e^-/Å^3$ and the largest hole was -0.174 $e^-/Å^3$ with an RMS deviation of 0.036 $e^-/Å^3$. On the basis of the final model, the calculated density was 1.287 g/cm^3 and F(000), 216 e^-. Flack parameter was refined to 0.04(13). CCDC Nr.: 2089176.

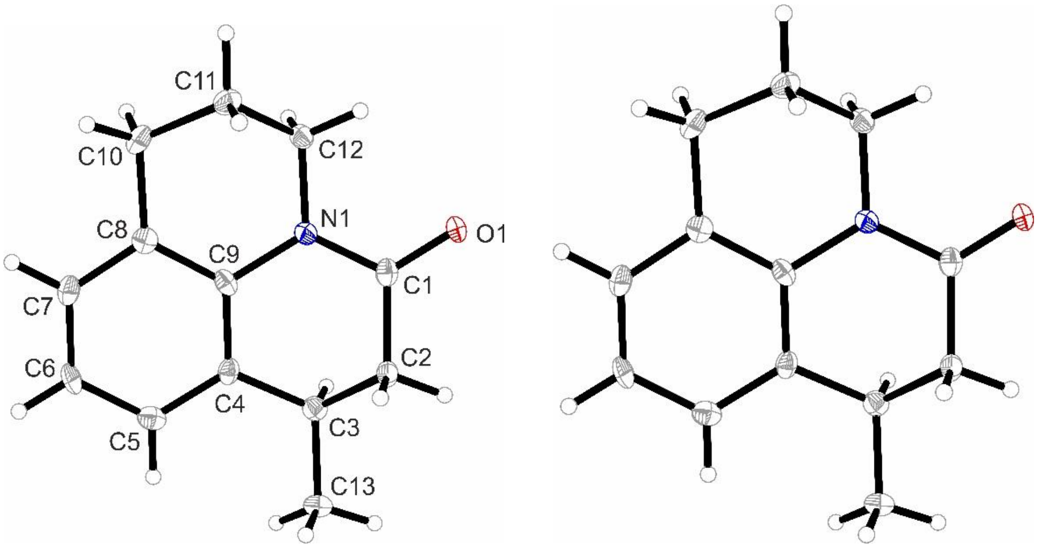

Figure S1: Crystal structure of compound **2v**. Thermal ellipsoids are shown at 30% probability.

6. Proposed enantiodiscrimination model

On the basis of our previous mechanism study[11] and experiment results, we propose a simple enantiodiscrimination model here. As shown in the following figure, precatalyst **C** could be transformed to complex **D** under hydrogen, which was considered as the active catalyst. The stereochemical outcome of this reaction depends on the attack direction from the metal centre of the catalyst. The attack via *Si* face was favored and the major (*R*)-product was generated in this case.

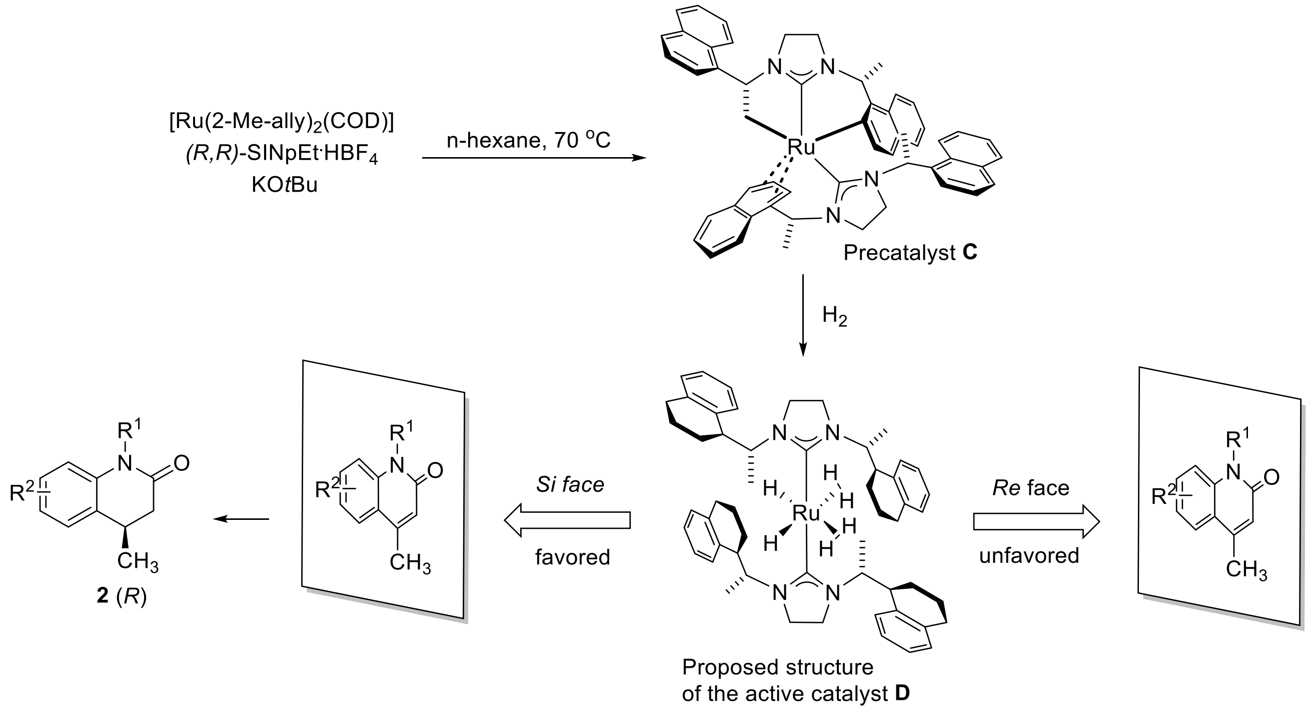

Figure S2: Proposed enantiodiscrimination model for the Ru-NHC catalyzed asymmetric hydrogenation of 2-quinolones

7. References

[1] N. Priya, A. Gupta, K. Chand, P. Singh, A. Kathuria, H. G. Raj, V. S. Parmar, S. K. Sharma, *Bioorg. Med. Chem*. **2010**, *18*, 4085-4094.

[2] M. Kuzuya, E. Mano, M. Adachi, A. Noguch, T. Okudj, *Chemistry Letters* **1982**, 475-478.

[3] K. K. S. Sai, T. M. Gilbert, D. A. Klumpp, *J. Org. Chem*. **2007**, *72*, 9761-9764.

[4] P. J. Manley, M. T. Bilodeau, *Org. Lett*. **2004**, *6*, 2433-2435.

[5] Y. Kim, E. Shin, P. Beak, Y. S. Park, *Synthesis* **2006**, *22*, 3805-3808.

[6] D. Moock, T. Wagener, T. Hu, T. Gallagher, F. Glorius, *Angew. Chem. Int. Ed.* **2021**, *60*, 13677-13681; *Angew. Chem.* **2021**, *133*, 13791-13796.

[7] Bruker AXS (**2019**) *APEX3 Version 2019.1-0*, *SAINT Version 8.40A* and *SADABS Bruker AXS area detector scaling and absorption correction Version 2016/2*, Bruker AXS Inc., Madison, Wisconsin, USA.

[8] Sheldrick, G. M., *SHELXT – Integrated space-group and crystal-structure determination*, *Acta Cryst.,* **2015**, *A71*, 3-8.

[9] Sheldrick, G.M., *Crystal structure refinement with SHELXL*, *Acta Cryst.*, **2015**, *C71 (1)*, 3-8.

[10] Bruker AXS (**1998**) *XP – Interactive molecular graphics, Version 5.1,* Bruker AXS Inc., Madison, Wisconsin, USA.

[11] D. Paul, B. Beiring, M. Plois, N. Ortega, S. Kock, D. Schluns, J. Neugebauer, R. Wolf, F. Glorius, *Organometallics* **2016**, *35*, 3641-3646.

8. Copies of NMR spectra

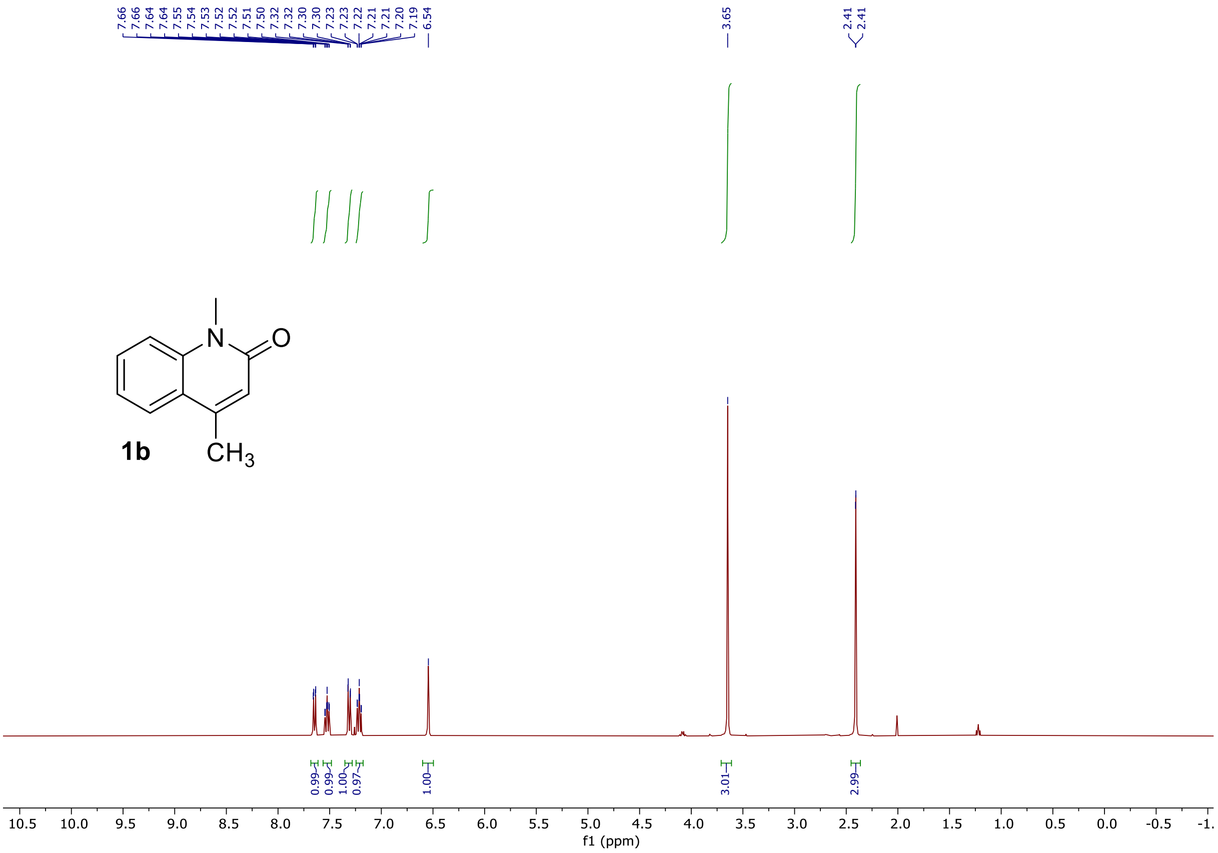

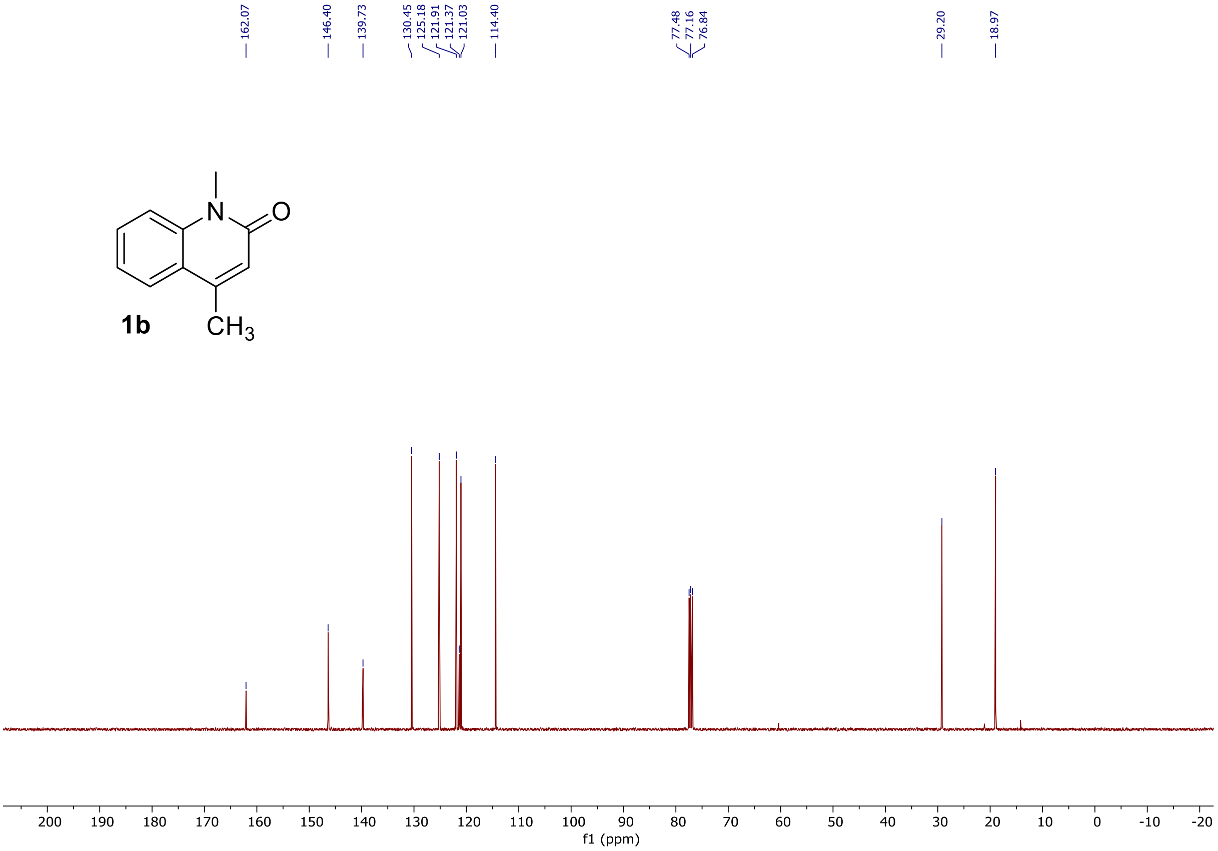

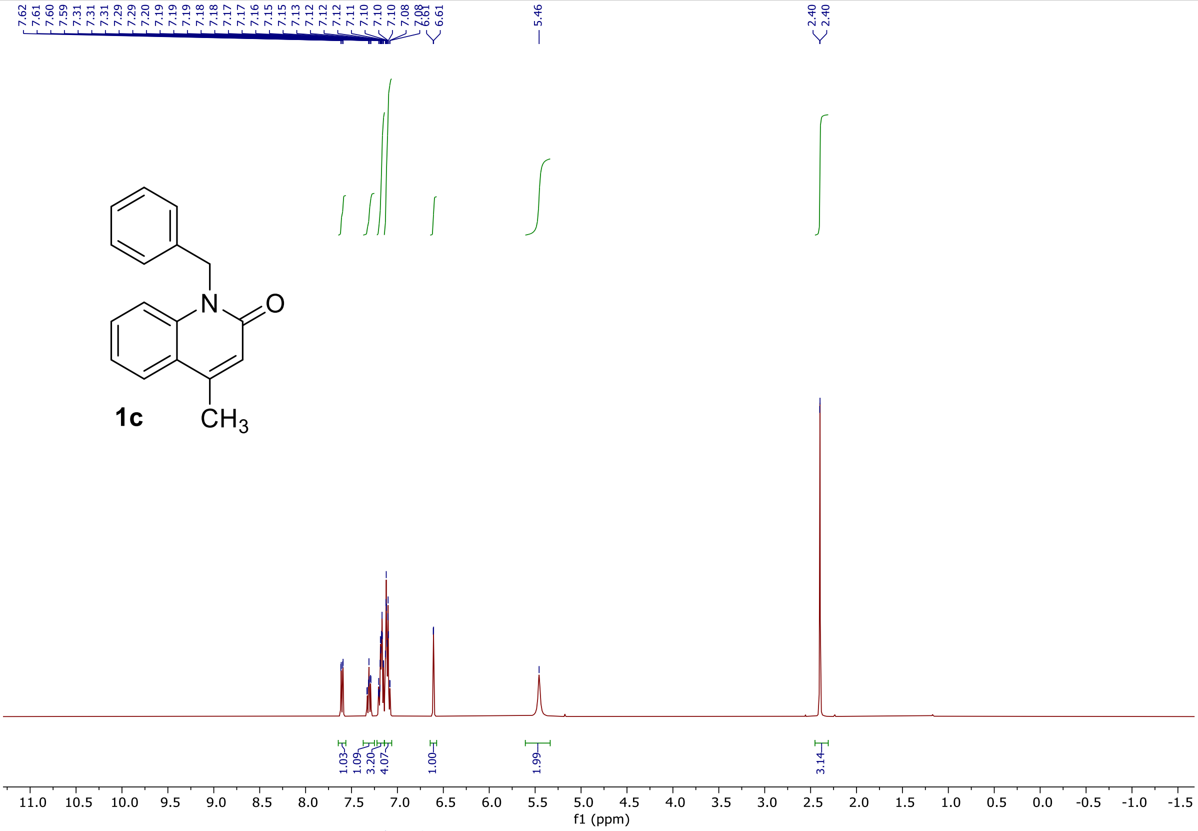

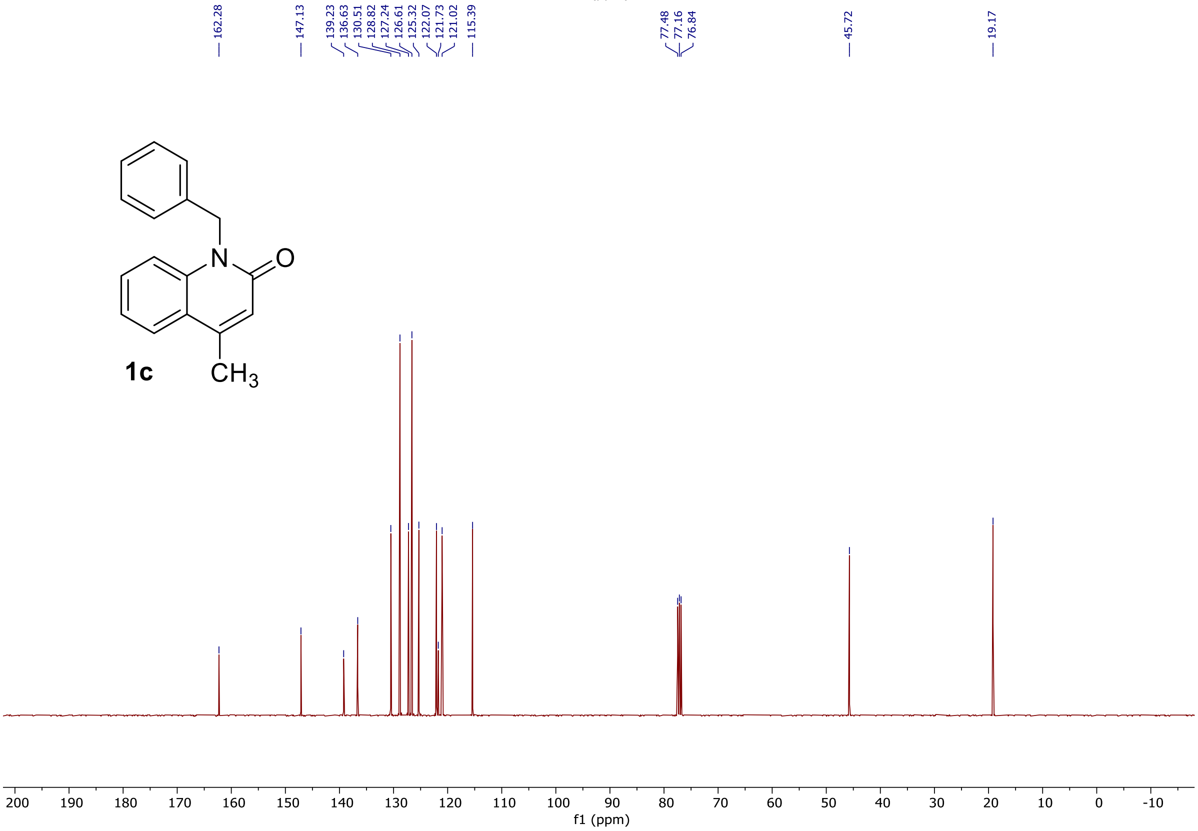

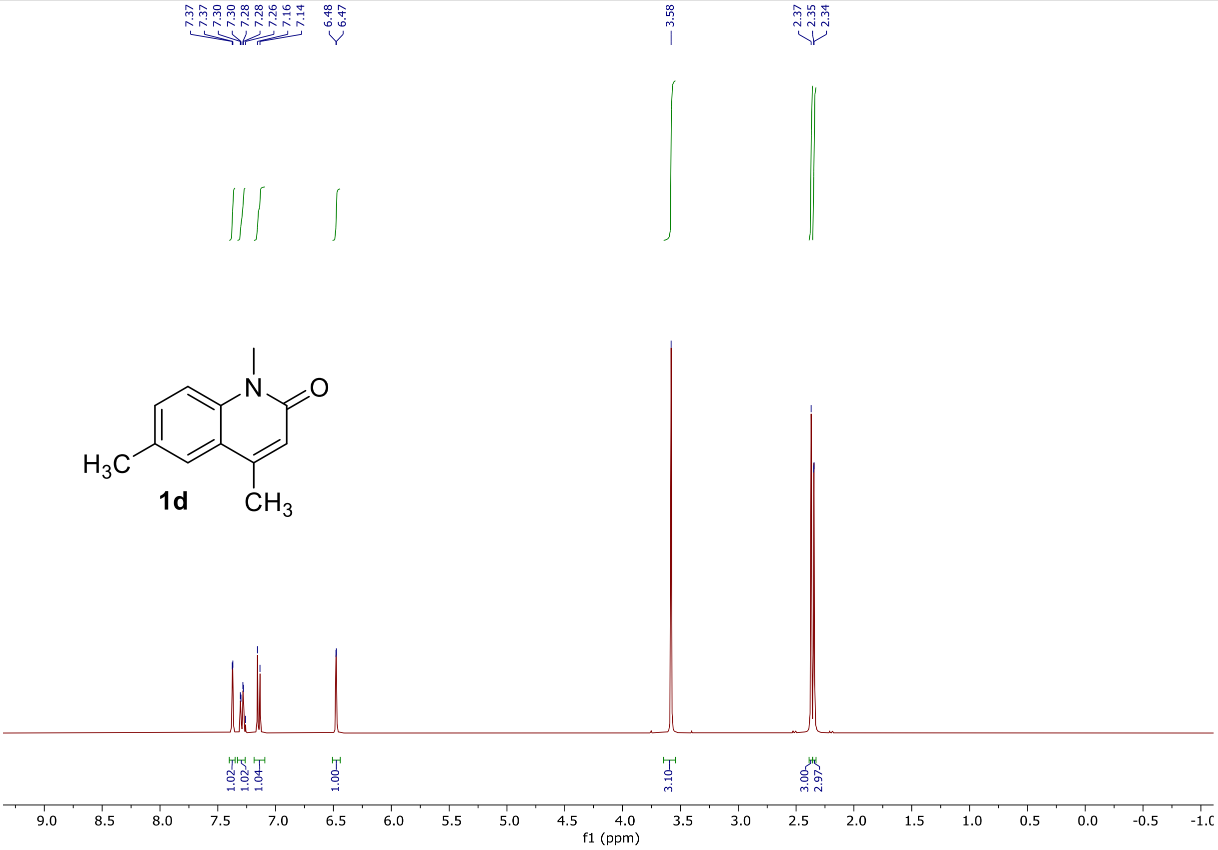
7.37
7.37
7.30
7.28
7.28
7.26
7.16
7.14
6.48
6.47
3.58
2.37
2.35
2.34
H3C
N
O
CH3
1d
1.02
1.02
1.04
1.00
3.10
3.00
2.97
9.0 8.5 8.0 7.5 7.0 6.5 6.0 5.5 5.0 4.5 4.0 3.5 3.0 2.5 2.0 1.5 1.0 0.5 0.0 -0.5
f1 (ppm)

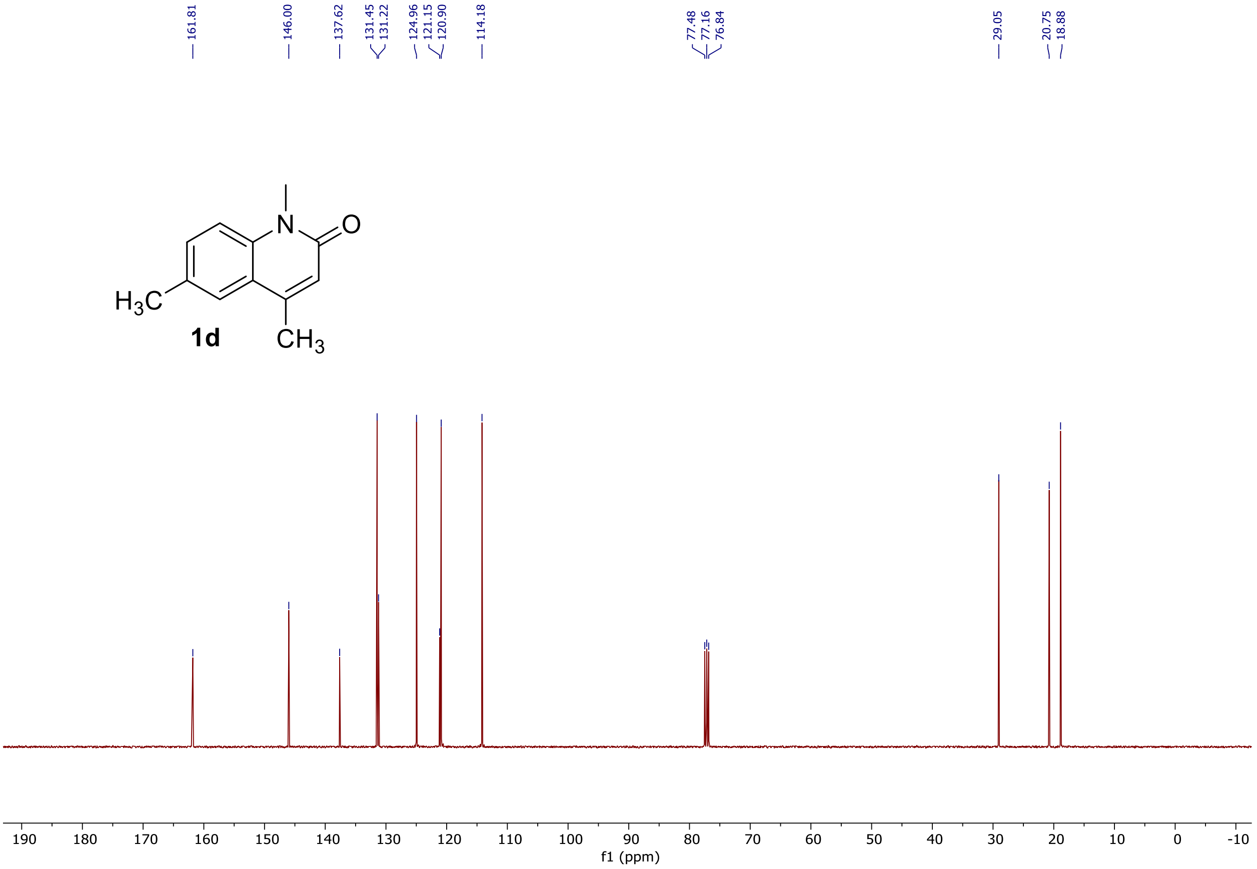
161.81
146.00
137.62
131.45
131.22
124.96
121.15
120.90
114.18
77.48
77.16
76.84
29.05
20.75
18.88
H3C
N
O
CH3
1d
190 180 170 160 150 140 130 120 110 100 90 80 70 60 50 40 30 20 10 0 -10
f1 (ppm)

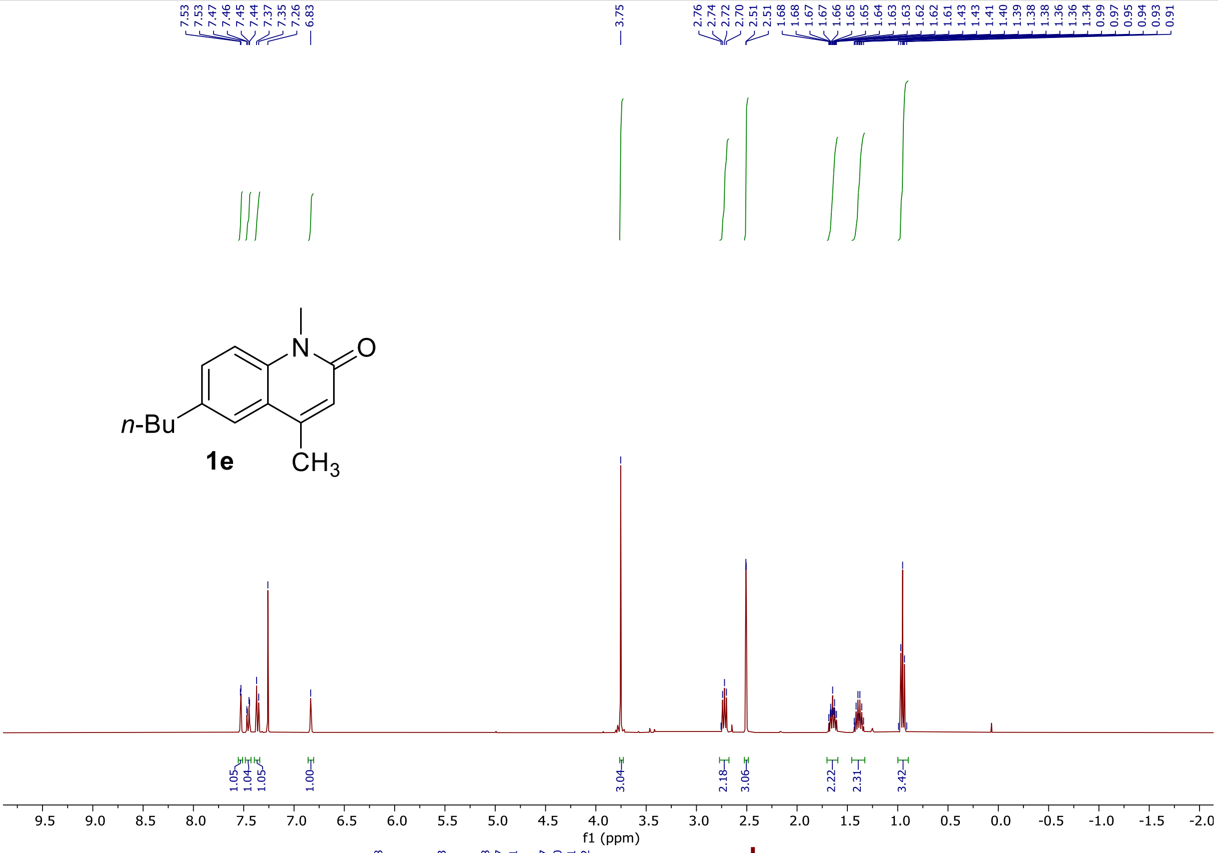
7.53
7.53
7.47
7.46
7.45
7.44
7.37
7.35
7.26
6.83
3.75
2.76
2.74
2.72
2.70
2.51
2.51
1.68
1.68
1.67
1.67
1.66
1.65
1.65
1.64
1.63
1.63
1.62
1.62
1.61
1.43
1.43
1.41
1.40
1.39
1.38
1.38
1.36
1.36
1.34
0.99
0.97
0.95
0.94
0.93
0.91
N
O
n-Bu
1e
CH3
1.05
1.04
1.05
1.00
3.04
2.18
3.06
2.22
2.31
3.42
f1 (ppm)

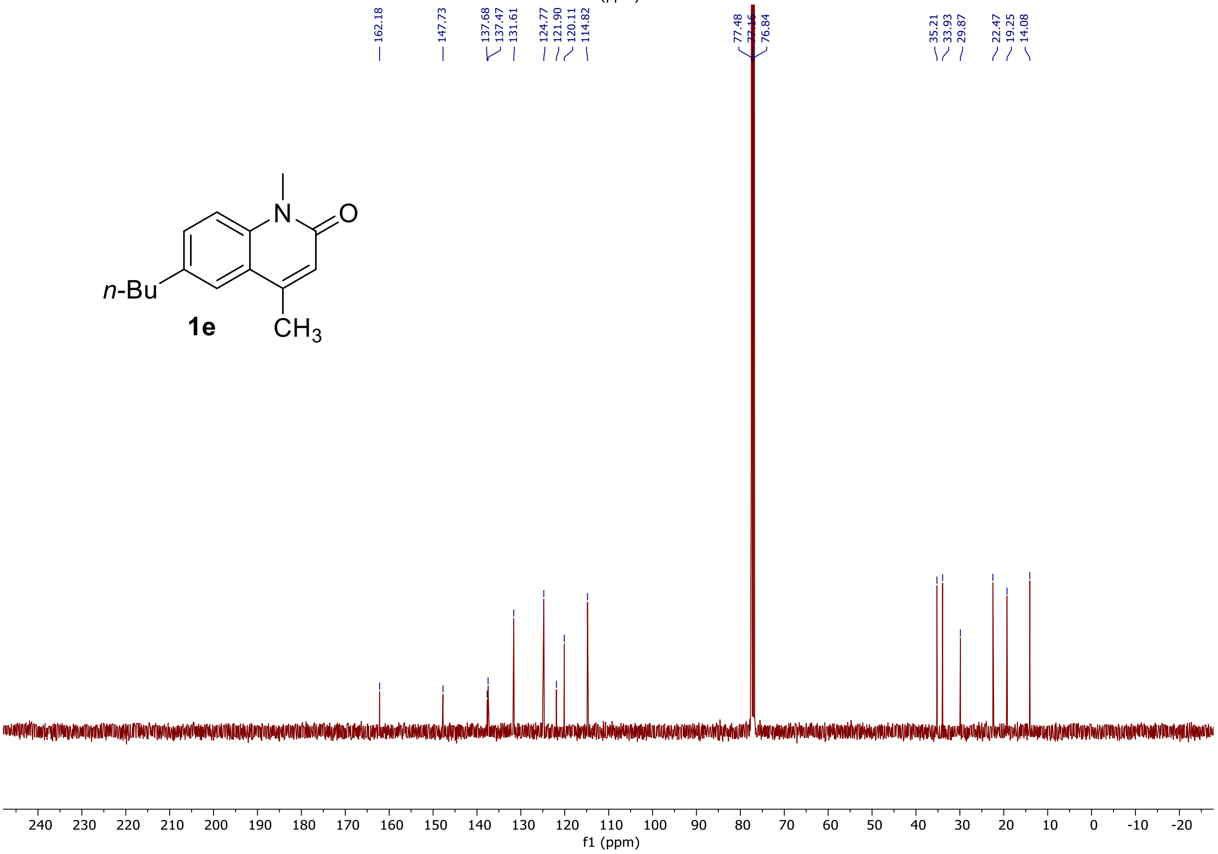
162.18
147.73
137.68
137.47
131.61
124.77
121.90
120.11
114.82
77.48
76.84
35.21
33.93
29.87
22.47
19.25
14.08
N
O
n-Bu
1e
CH3
f1 (ppm)

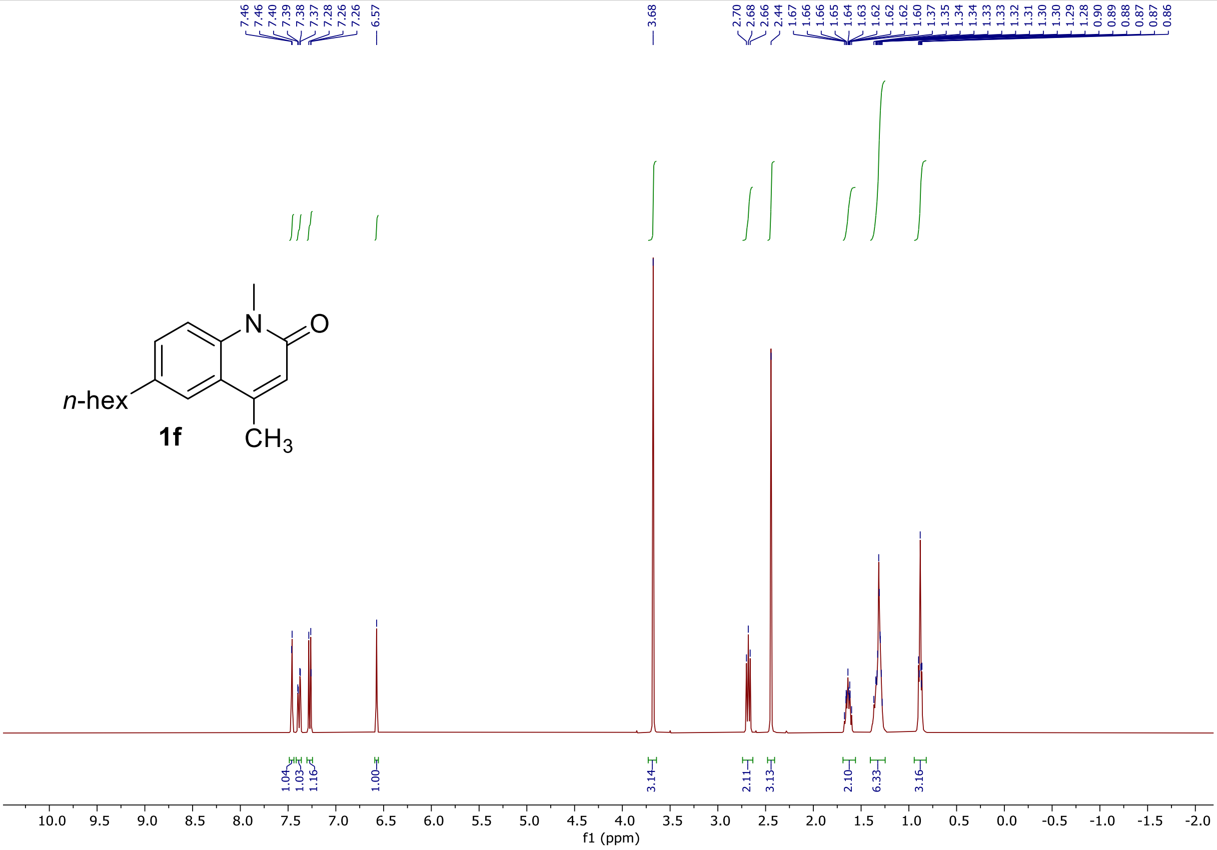
7.46
7.46
7.40
7.39
7.38
7.37
7.28
7.26
7.26
6.57
3.68
2.70
2.68
2.66
2.44
1.67
1.66
1.65
1.64
1.63
1.62
1.62
1.60
1.37
1.35
1.34
1.34
1.33
1.33
1.32
1.31
1.30
1.30
1.29
1.28
0.90
0.89
0.88
0.87
0.87
0.86
n-hex
1f
CH3
N
O
1.04
1.03
1.16
1.00
3.14
2.11
3.13
2.10
6.33
3.16
f1 (ppm)

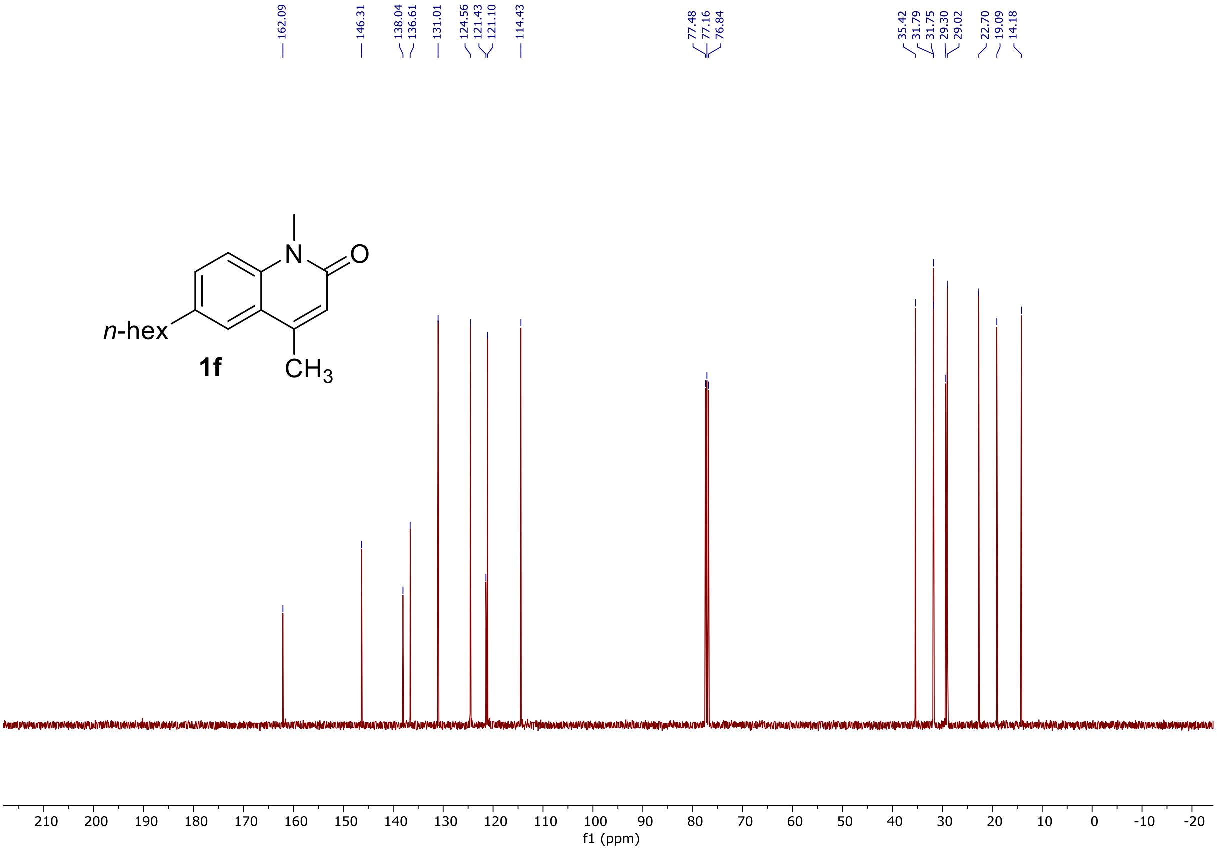
162.09
146.31
138.04
136.61
131.01
124.56
121.43
121.10
114.43
77.48
77.16
76.84
35.42
31.79
31.75
29.30
29.02
22.70
19.09
14.18
n-hex
1f
CH3
N
O
f1 (ppm)

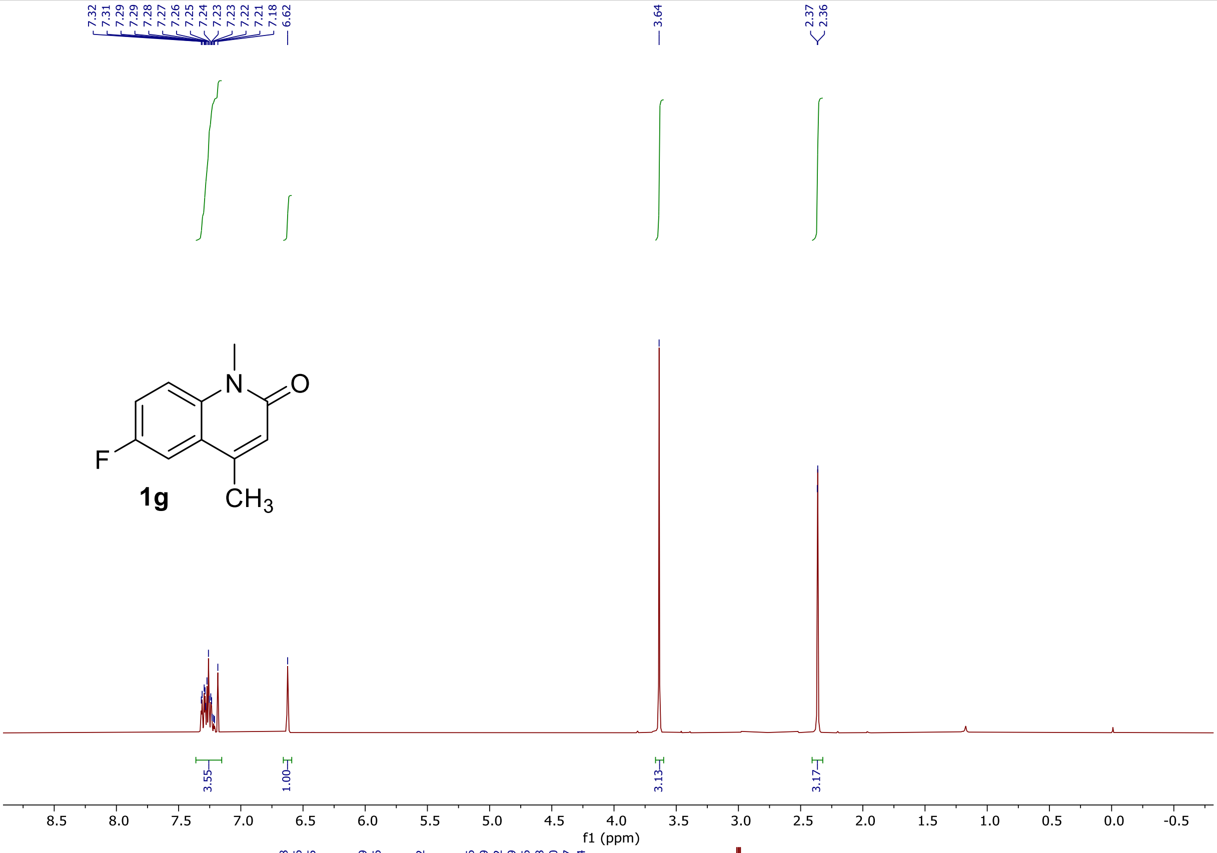
7.32
7.31
7.29
7.29
7.28
7.27
7.26
7.25
7.24
7.23
7.23
7.22
7.21
7.18
6.62
3.64
2.37
2.36
F
N
O
1g
CH3
3.55
1.00
3.13
3.17
8.5 8.0 7.5 7.0 6.5 6.0 5.5 5.0 4.5 4.0 3.5 3.0 2.5 2.0 1.5 1.0 0.5 0.0 -0.5
f1 (ppm)

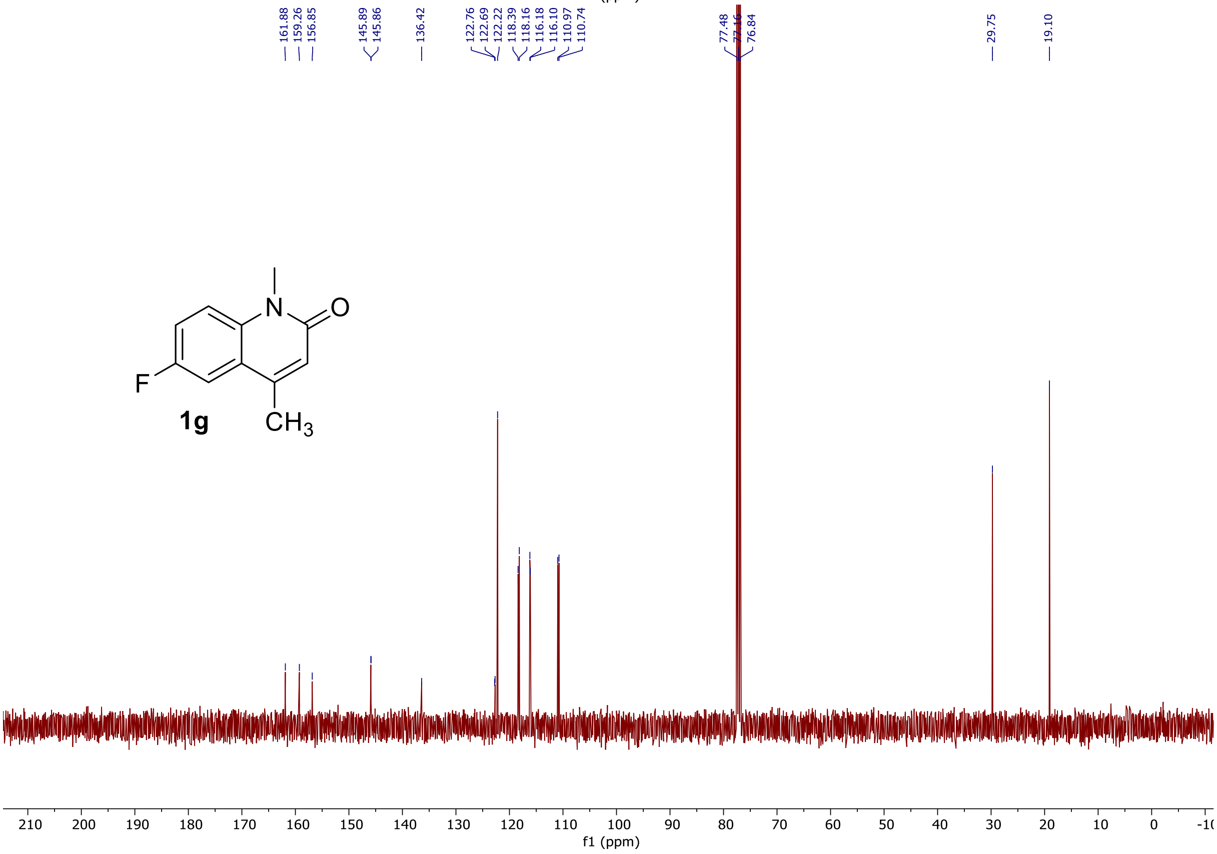
161.88
159.26
156.85
145.89
145.86
136.42
122.76
122.69
122.22
118.39
118.16
116.18
116.10
110.97
110.74
77.48
76.84
29.75
19.10
F
N
O
1g
CH3
210 200 190 180 170 160 150 140 130 120 110 100 90 80 70 60 50 40 30 20 10 0
f1 (ppm)

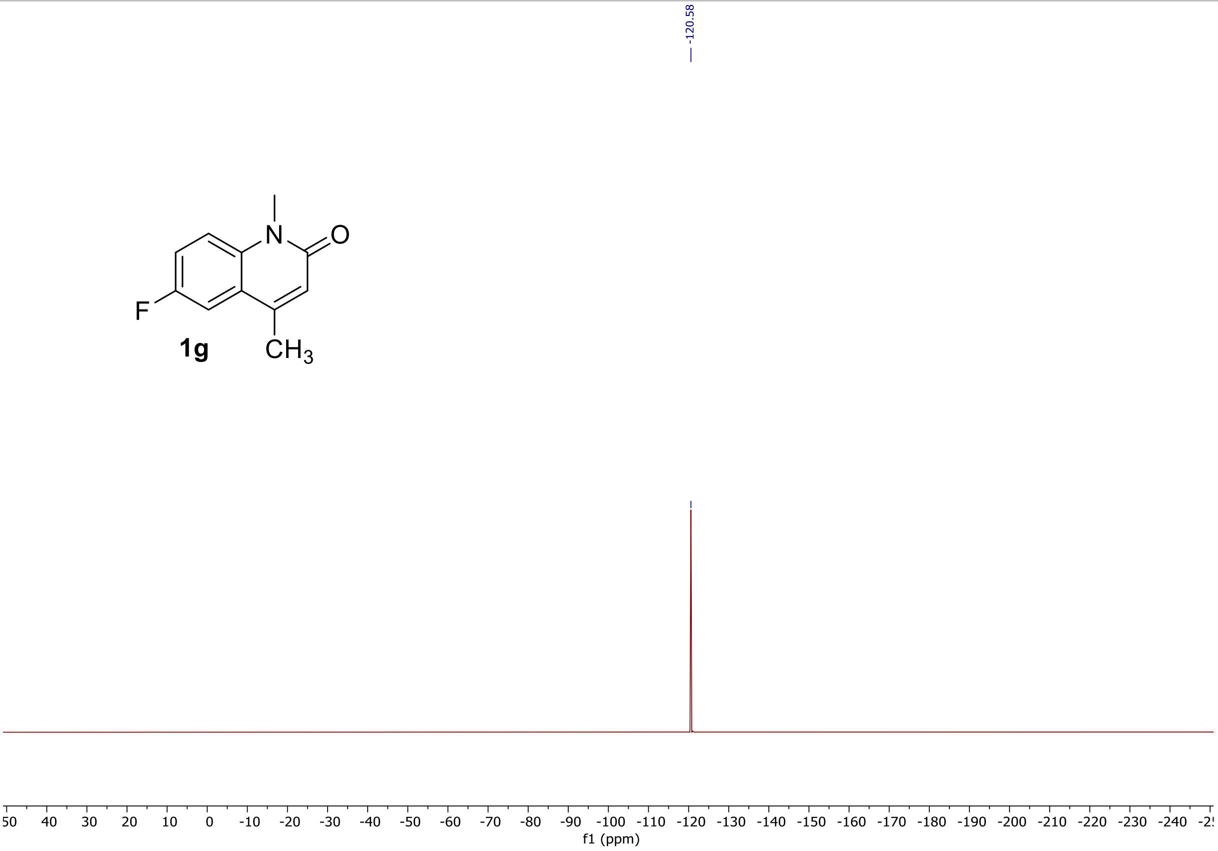
-120.58
1g
F
N
O
CH3
f1 (ppm)

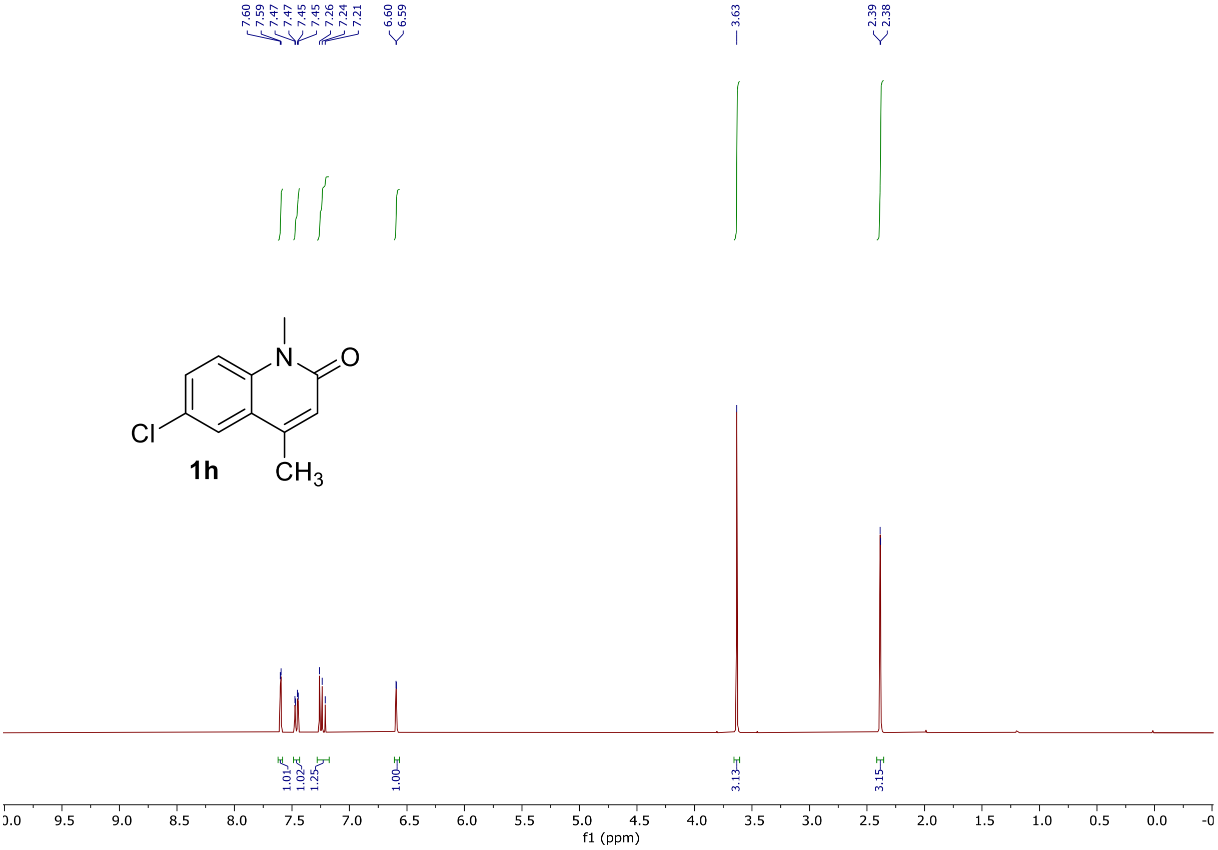
7.60 7.59 7.47 7.45 7.45 7.26 7.24 7.21
6.60 6.59
3.63
2.39 2.38
1h
Cl
N
O
CH3
1.01 1.02 1.25 1.00 3.13 3.15
f1 (ppm)

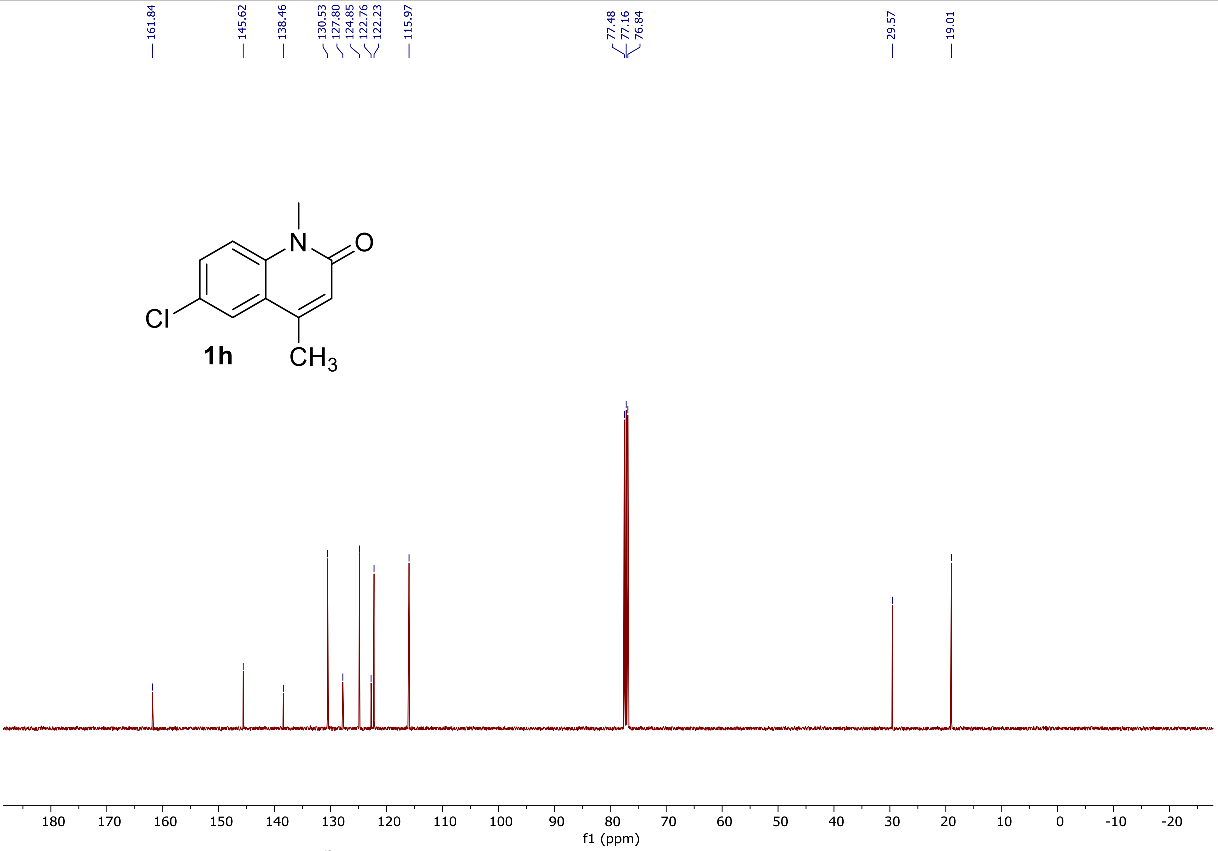
161.84
145.62
138.46
130.53
127.80
124.85
122.76
122.23
115.97
77.48
77.16
76.84
29.57
19.01
Cl
N
O
CH3
1h
180 170 160 150 140 130 120 110 100 90 80 70 60 50 40 30 20 10 0 -10 -20
f1 (ppm)

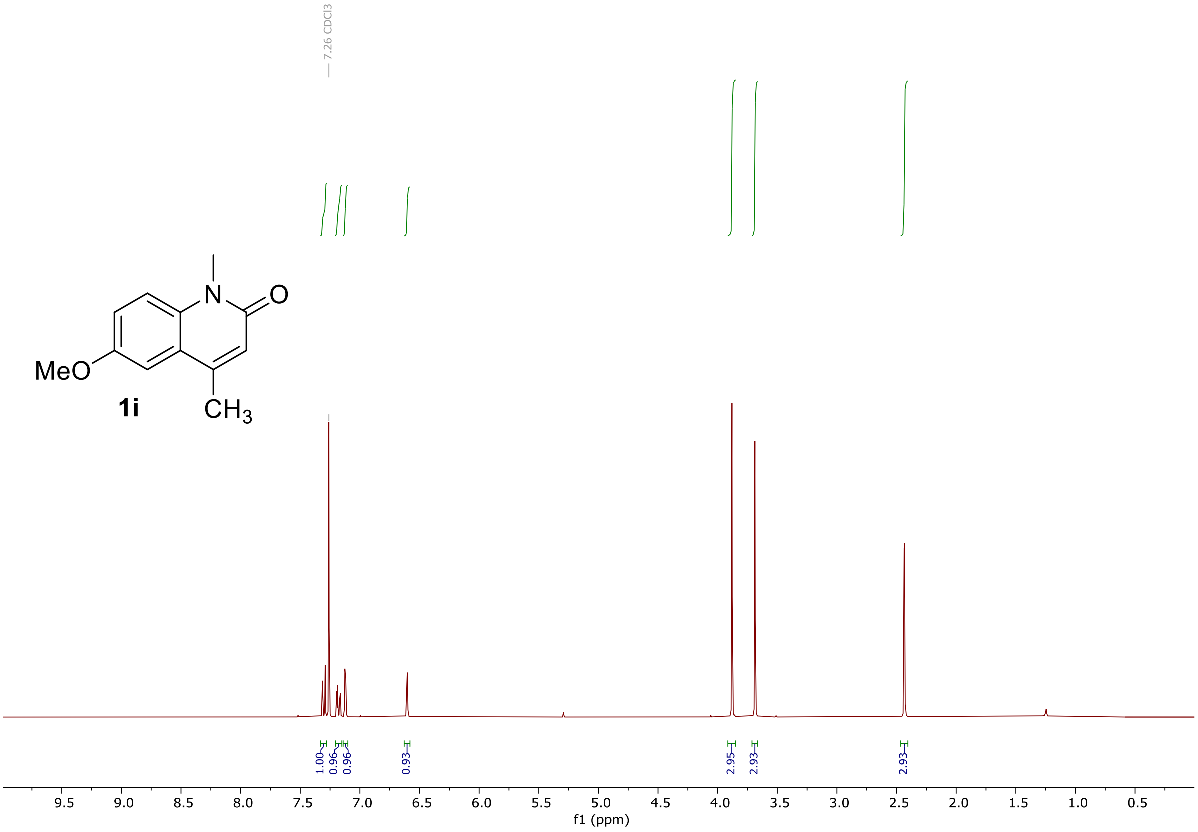
7.26 CDCl3
MeO
N
O
CH3
1i
1.00
0.96
0.96
0.93
2.95
2.93
2.93
9.5 9.0 8.5 8.0 7.5 7.0 6.5 6.0 5.5 5.0 4.5 4.0 3.5 3.0 2.5 2.0 1.5 1.0 0.5
f1 (ppm)

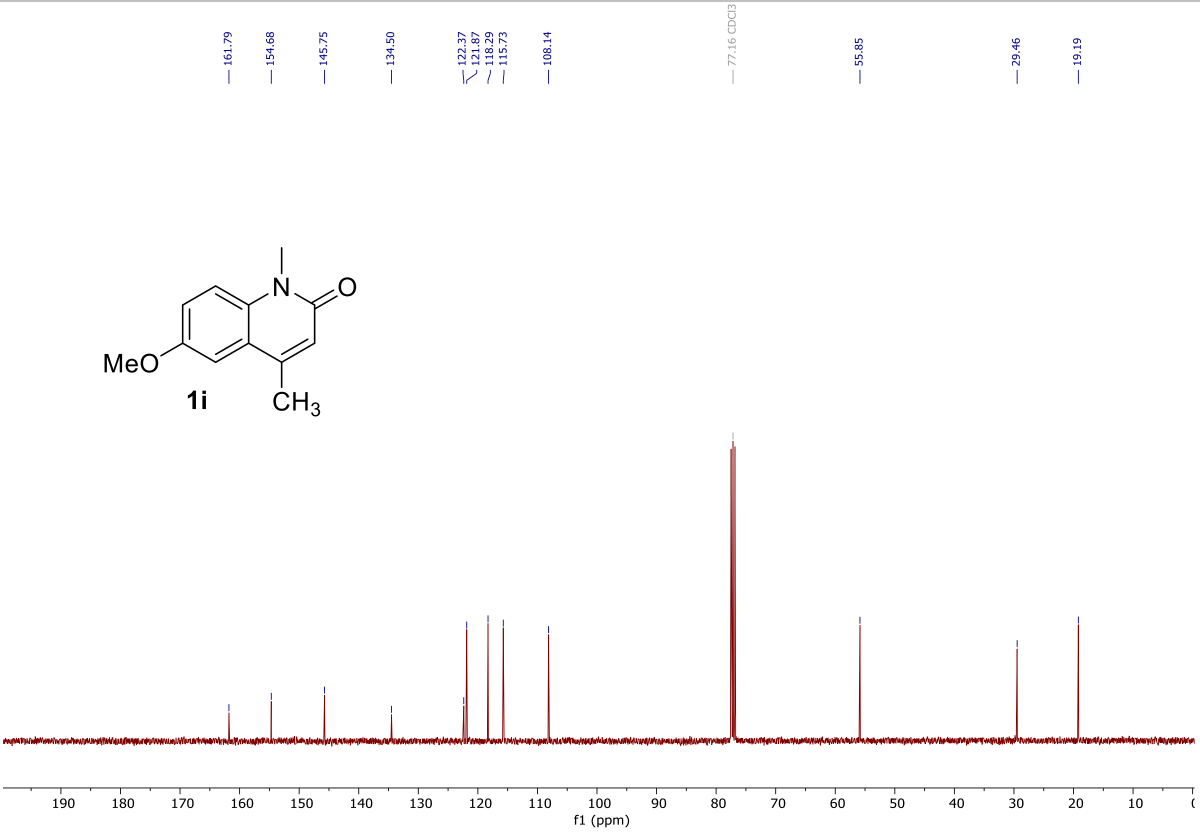
161.79
154.68
145.75
134.50
122.37
121.87
118.29
115.73
108.14
77.16 CDCl3
55.85
29.46
19.19
MeO
N
O
1i
CH3
f1 (ppm)

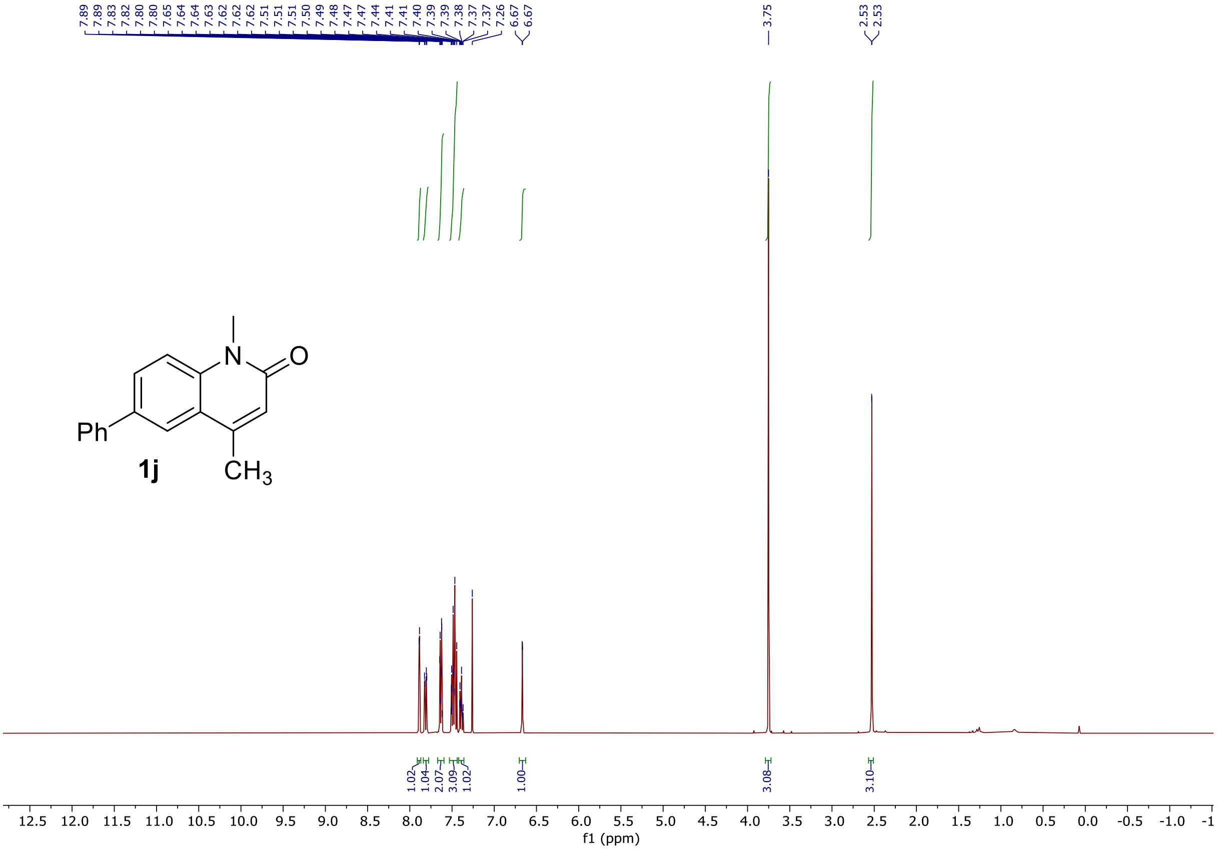
3.75
2.53
2.53
N
O
Ph
1j
CH3
1.02
1.04
2.07
3.09
1.02
1.00
3.08
3.10
f1 (ppm)

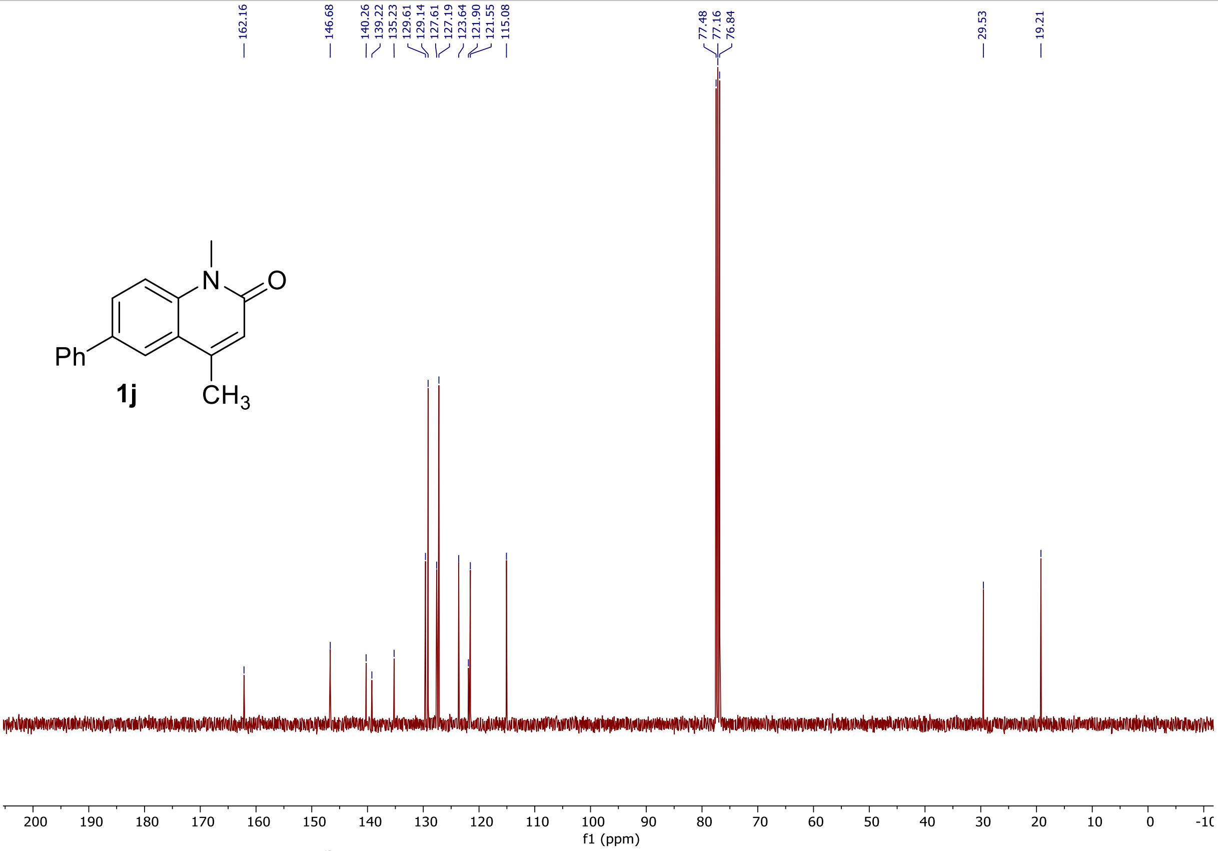
162.16
146.68
140.26
139.22
135.23
129.61
129.14
127.61
127.19
123.64
121.90
121.55
115.08
77.48
77.16
76.84
29.53
19.21
Ph
1j
CH_3
N
O
f1 (ppm)

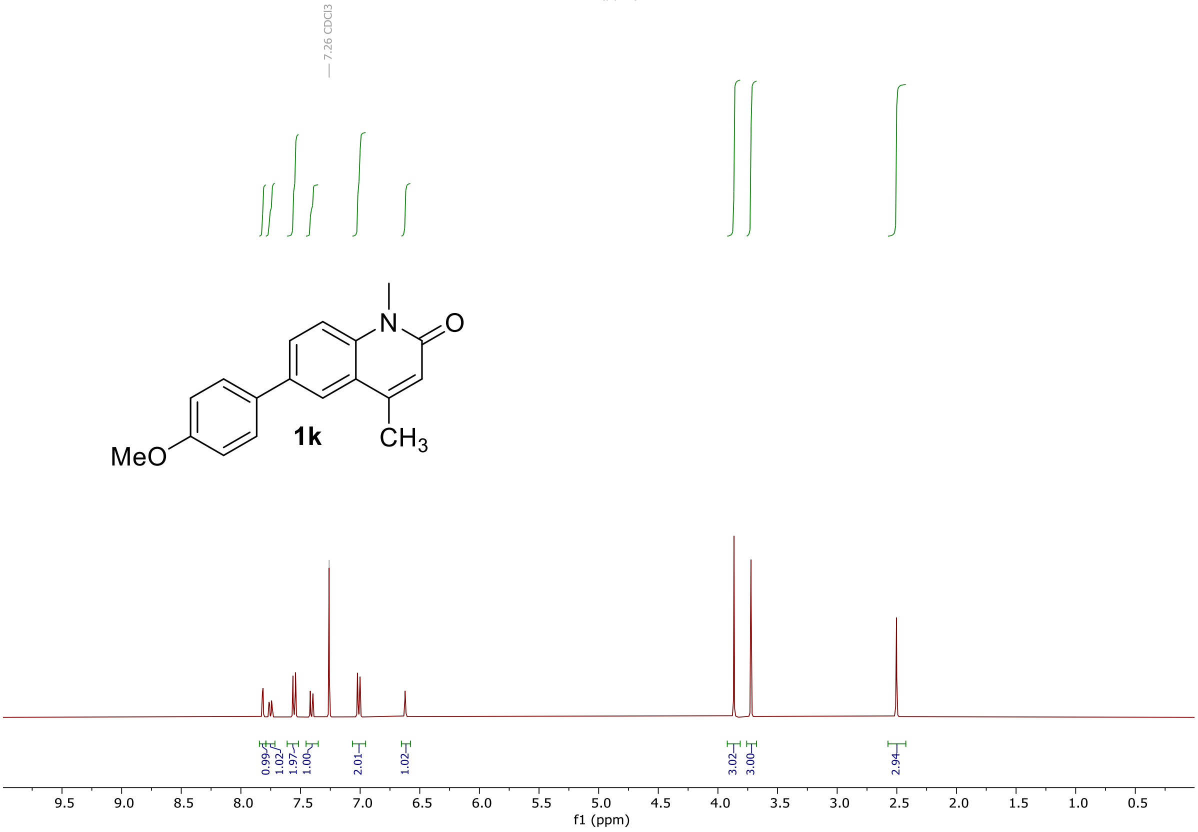
7.26 CDCl3
MeO
1k
CH_3
N
O
0.99
1.02
1.97
1.00
2.01
1.02
3.02
3.00
2.94
f1 (ppm)

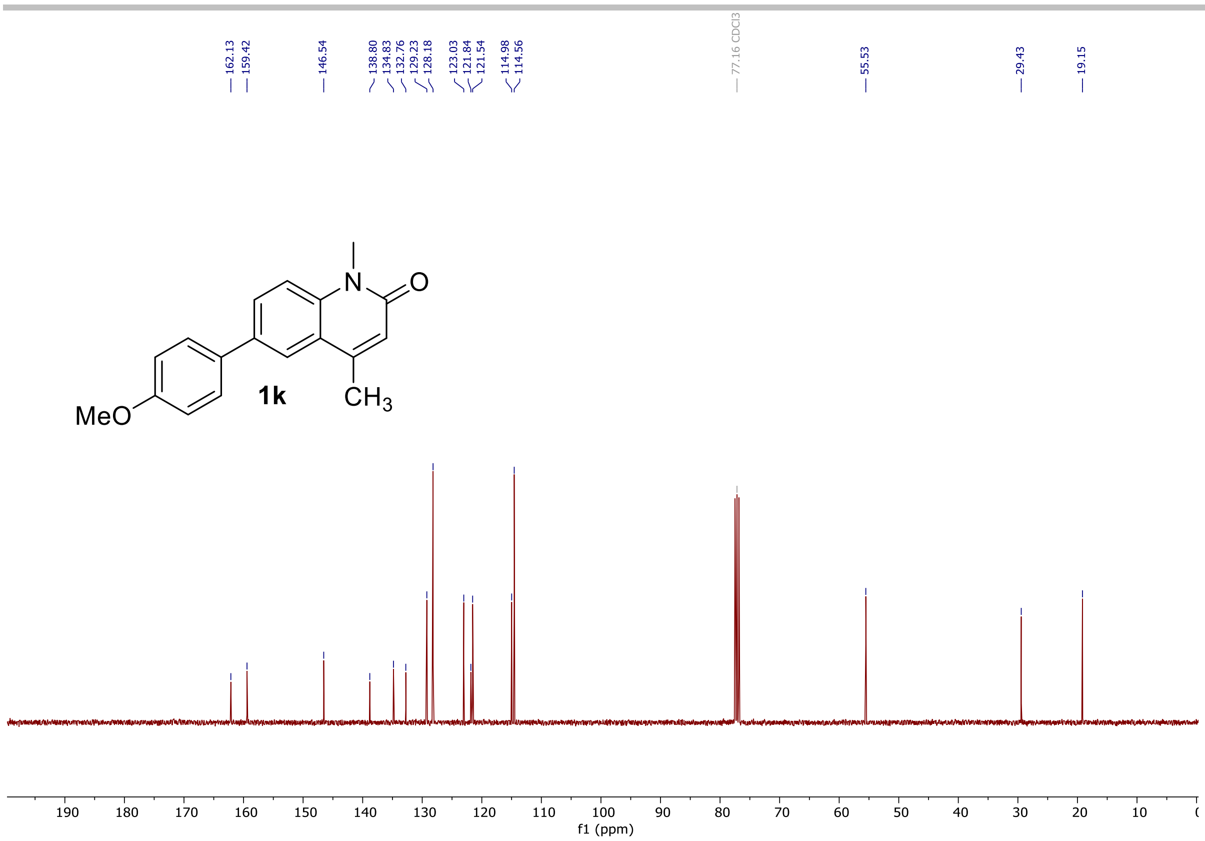
162.13
159.42
146.54
138.80
134.83
132.76
129.23
128.18
123.03
121.84
121.54
114.98
114.56
77.16 CDCl3
55.53
29.43
19.15
MeO
N
O
1k
CH3
f1 (ppm)

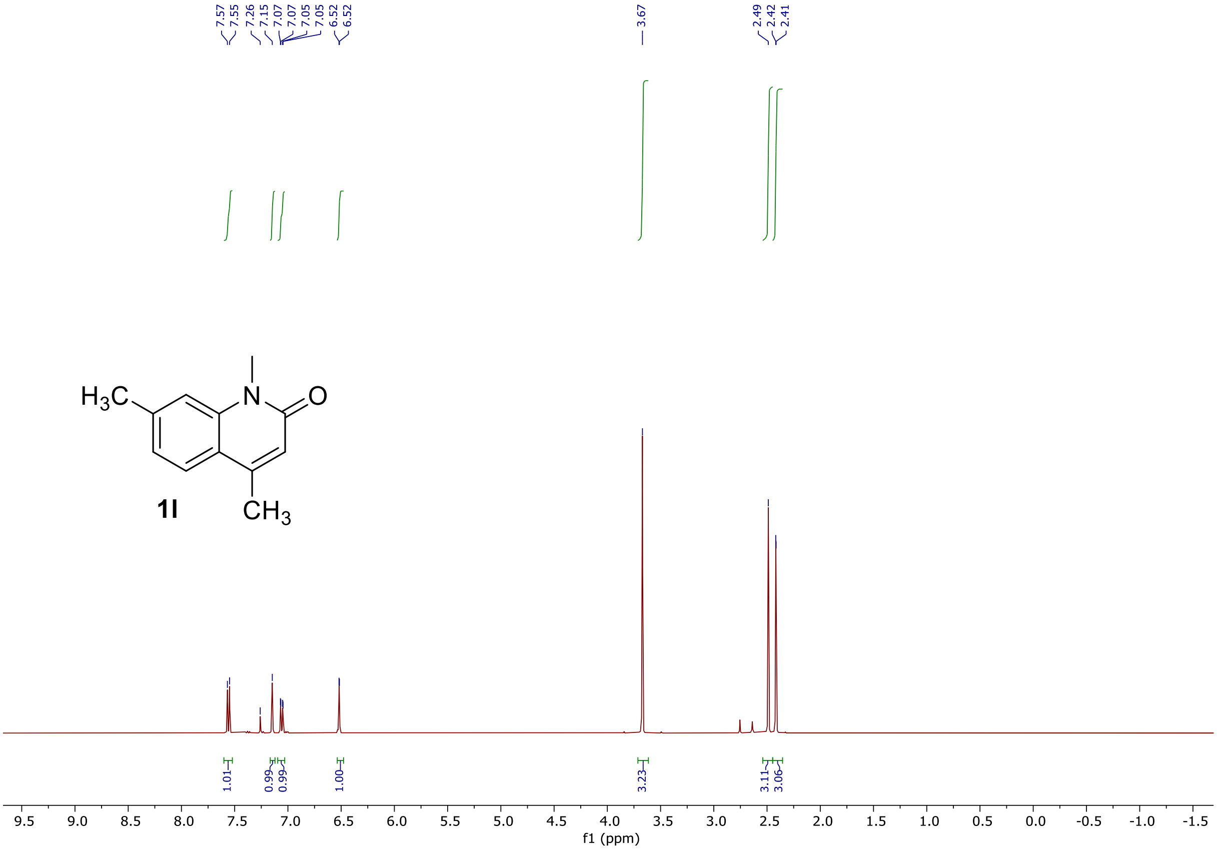
7.57
7.55
7.26
7.15
7.07
7.07
7.05
7.05
6.52
6.52
3.67
2.49
2.42
2.41
H3C
N
O
1l
CH3
1.01
0.99
0.99
1.00
3.23
3.11
3.06
f1 (ppm)

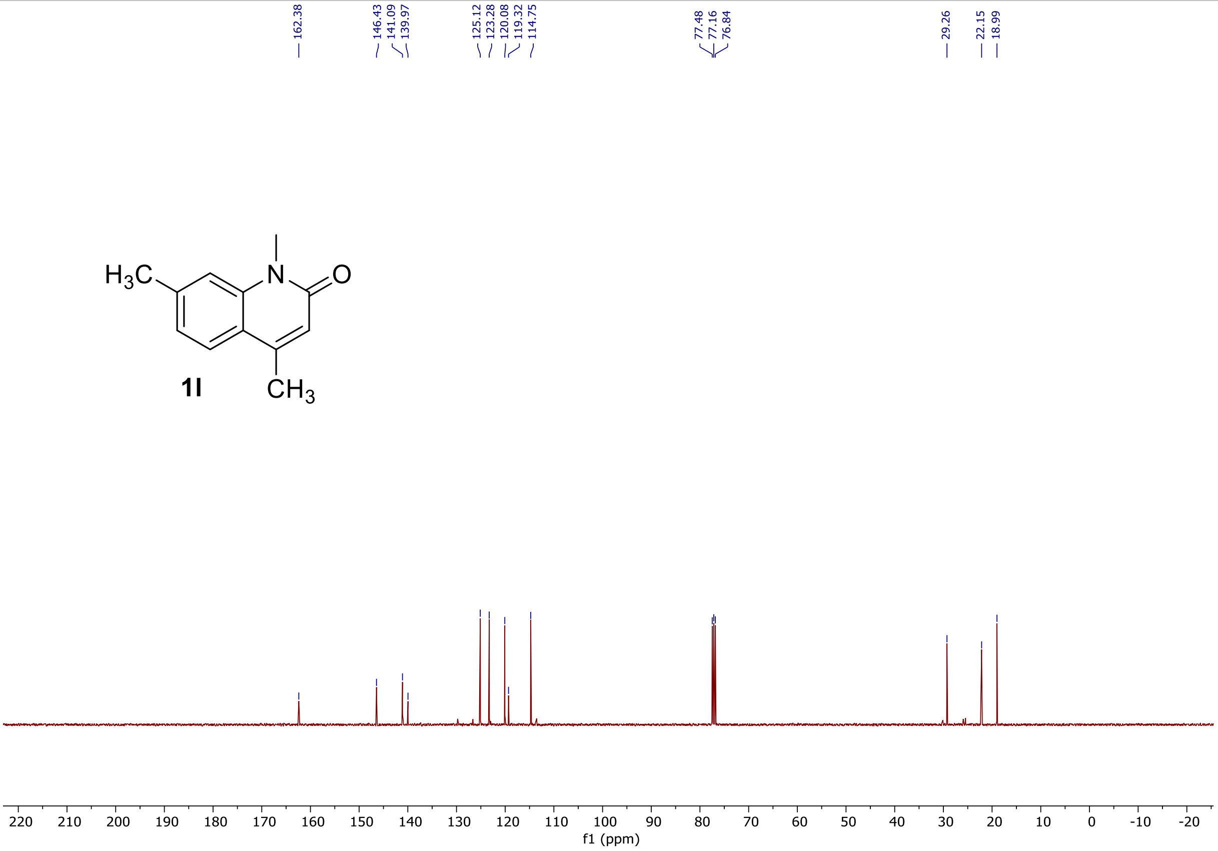
162.38
146.43
141.09
139.97
125.12
123.28
120.08
119.32
114.75
77.48
77.16
76.84
29.26
22.15
18.99
H_3C
N
O
1l
CH_3
220 210 200 190 180 170 160 150 140 130 120 110 100 90 80 70 60 50 40 30 20 10 0 -10 -20
f1 (ppm)

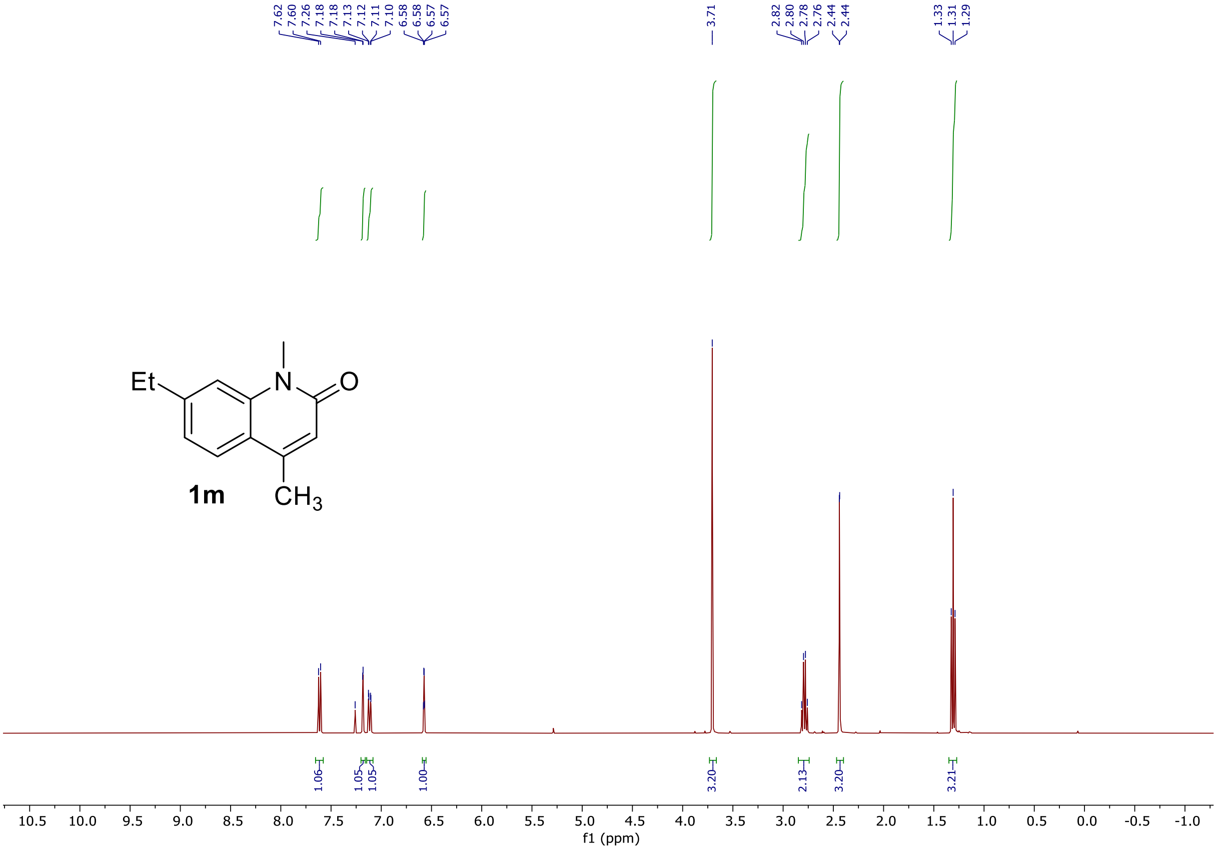
7.62
7.60
7.26
7.18
7.18
7.13
7.12
7.11
7.10
6.58
6.58
6.57
6.57
3.71
2.82
2.80
2.78
2.76
2.44
2.44
1.33
1.31
1.29
Et
N
O
1m
CH_3
1.06
1.05
1.05
1.00
3.20
2.13
3.20
3.21
10.5 10.0 9.5 9.0 8.5 8.0 7.5 7.0 6.5 6.0 5.5 5.0 4.5 4.0 3.5 3.0 2.5 2.0 1.5 1.0 0.5 0.0 -0.5 -1.0
f1 (ppm)

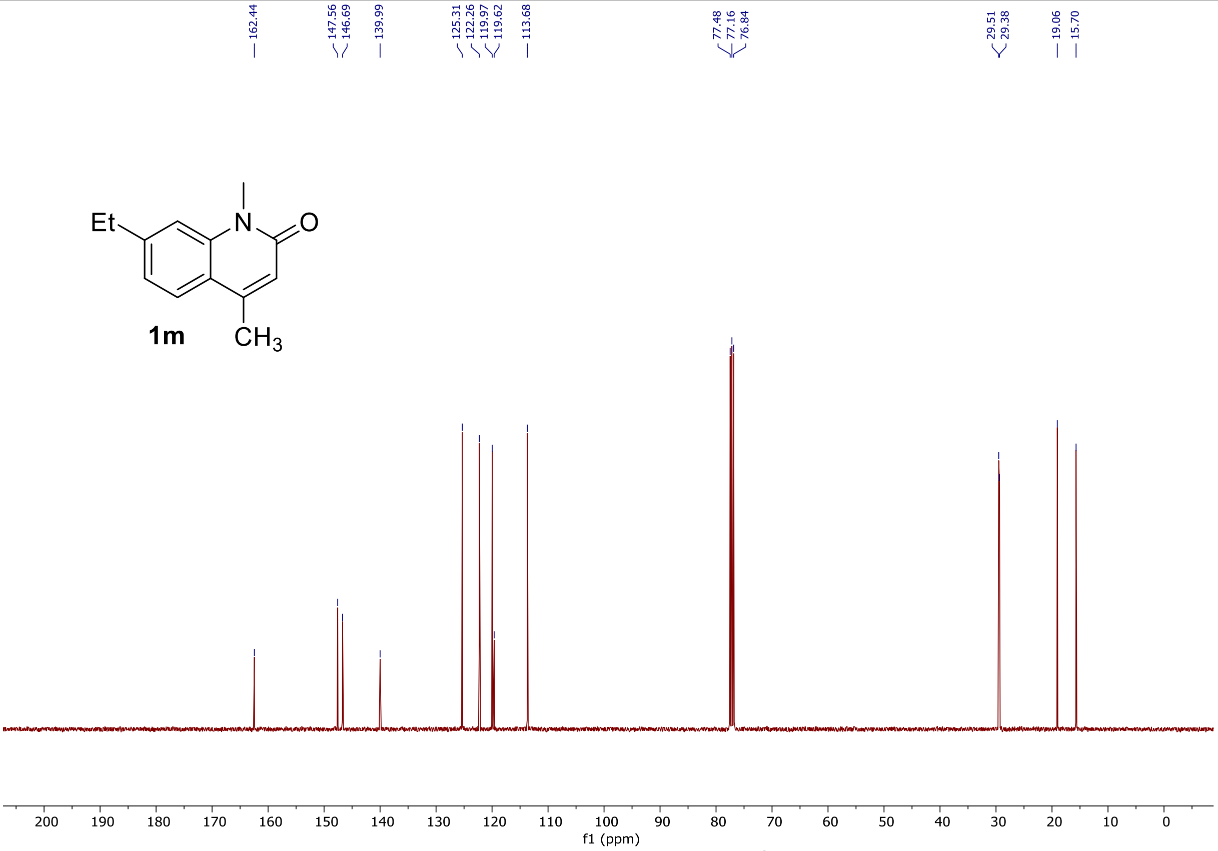
162.44
147.56
146.69
139.99
125.31
122.26
119.97
119.62
113.68
77.48
77.16
76.84
29.51
29.38
19.06
15.70
Et
N
O
1m
CH3
f1 (ppm)

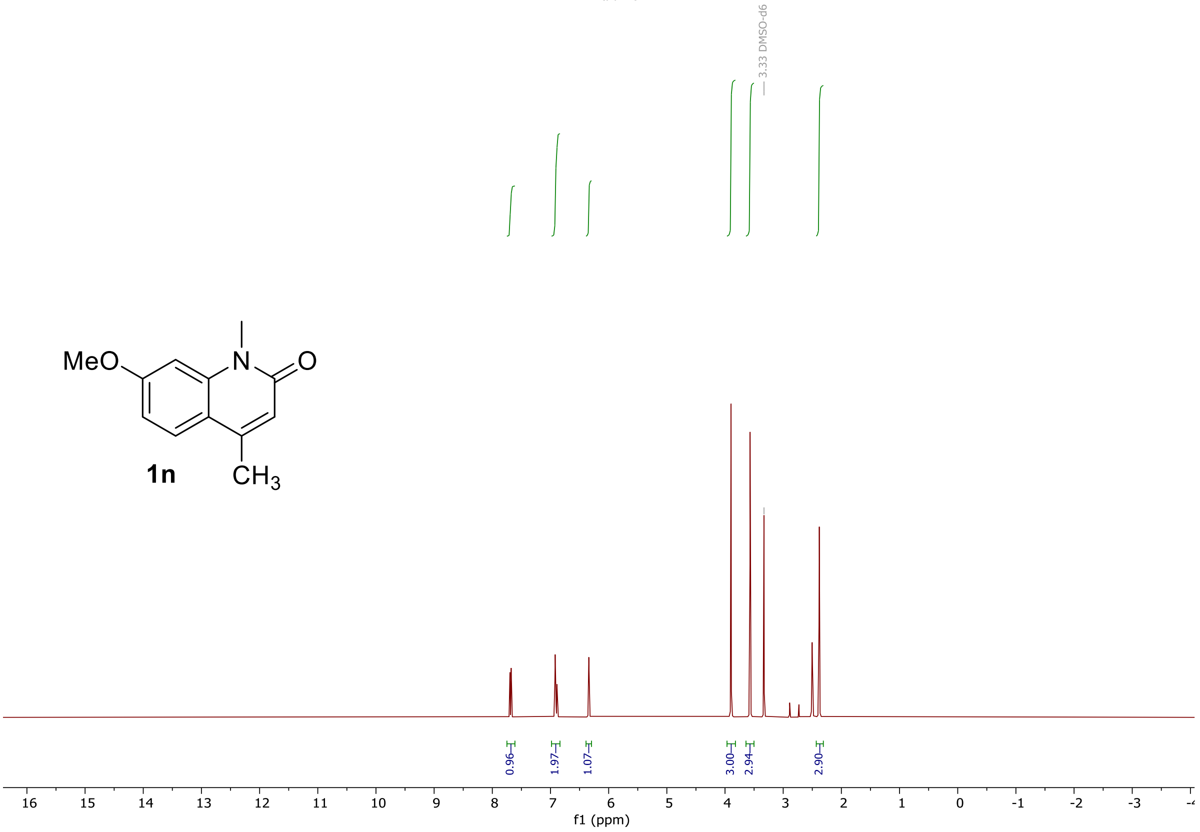
3.33 DMSO-d6
MeO
N
O
1n
CH3
0.96
1.97
1.07
3.00
2.94
2.90
f1 (ppm)

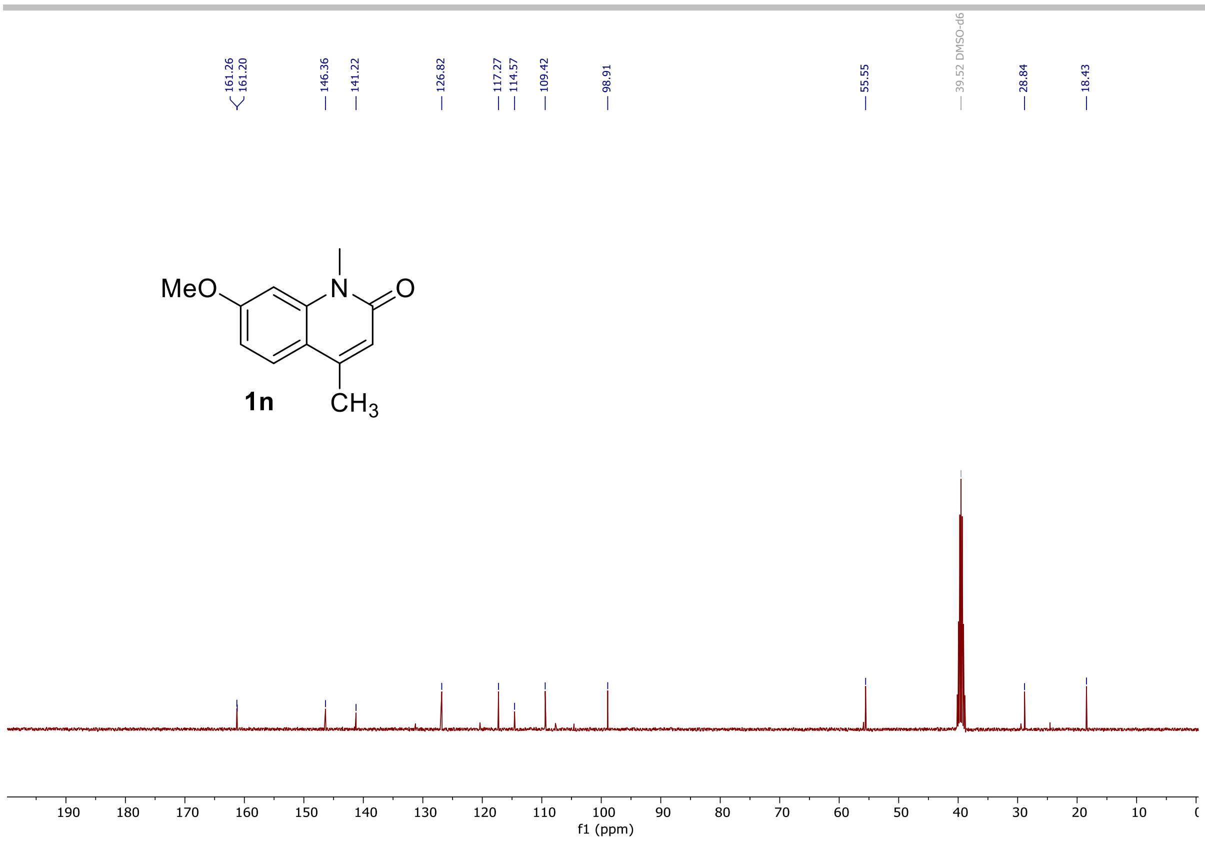
161.26
161.20
146.36
141.22
126.82
117.27
114.57
109.42
98.91
55.55
39.52 DMSO-d6
28.84
18.43
MeO
N
O
1n
CH3
f1 (ppm)

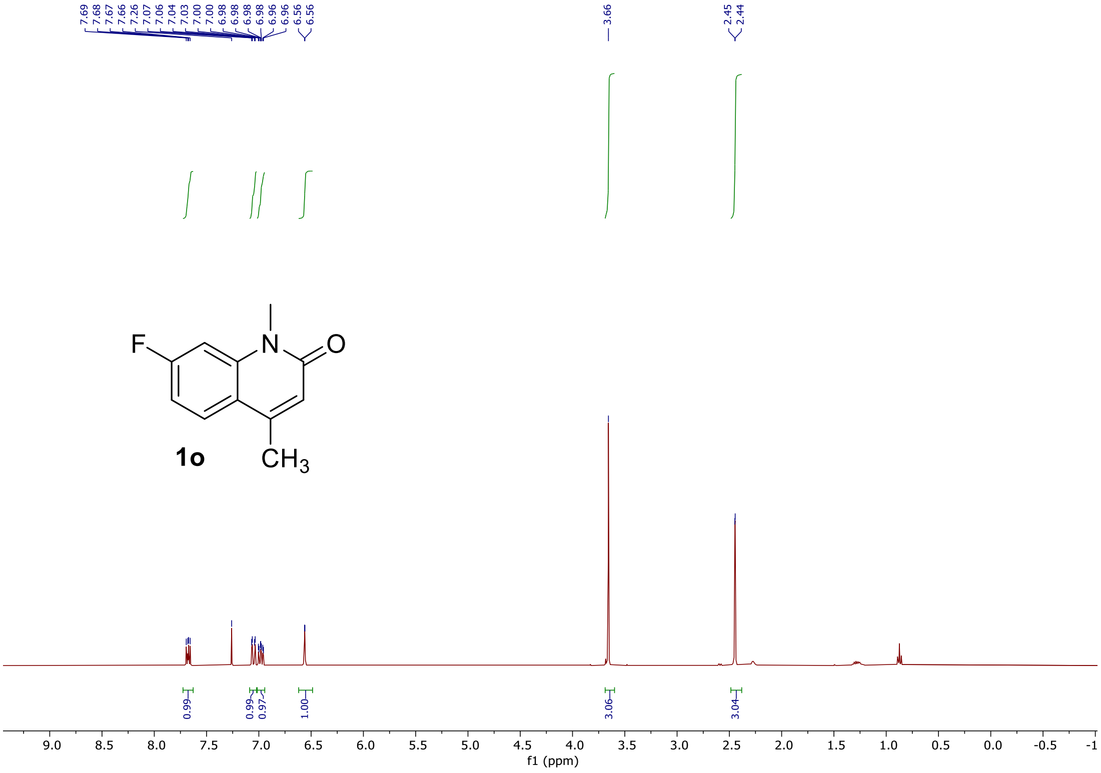
7.69
7.68
7.67
7.66
7.26
7.07
7.06
7.04
7.03
7.00
7.00
6.98
6.98
6.98
6.98
6.96
6.96
6.56
6.56
3.66
2.45
2.44
F
N
O
1o
CH3
0.99
0.99
0.97
1.00
3.06
3.04
f1 (ppm)

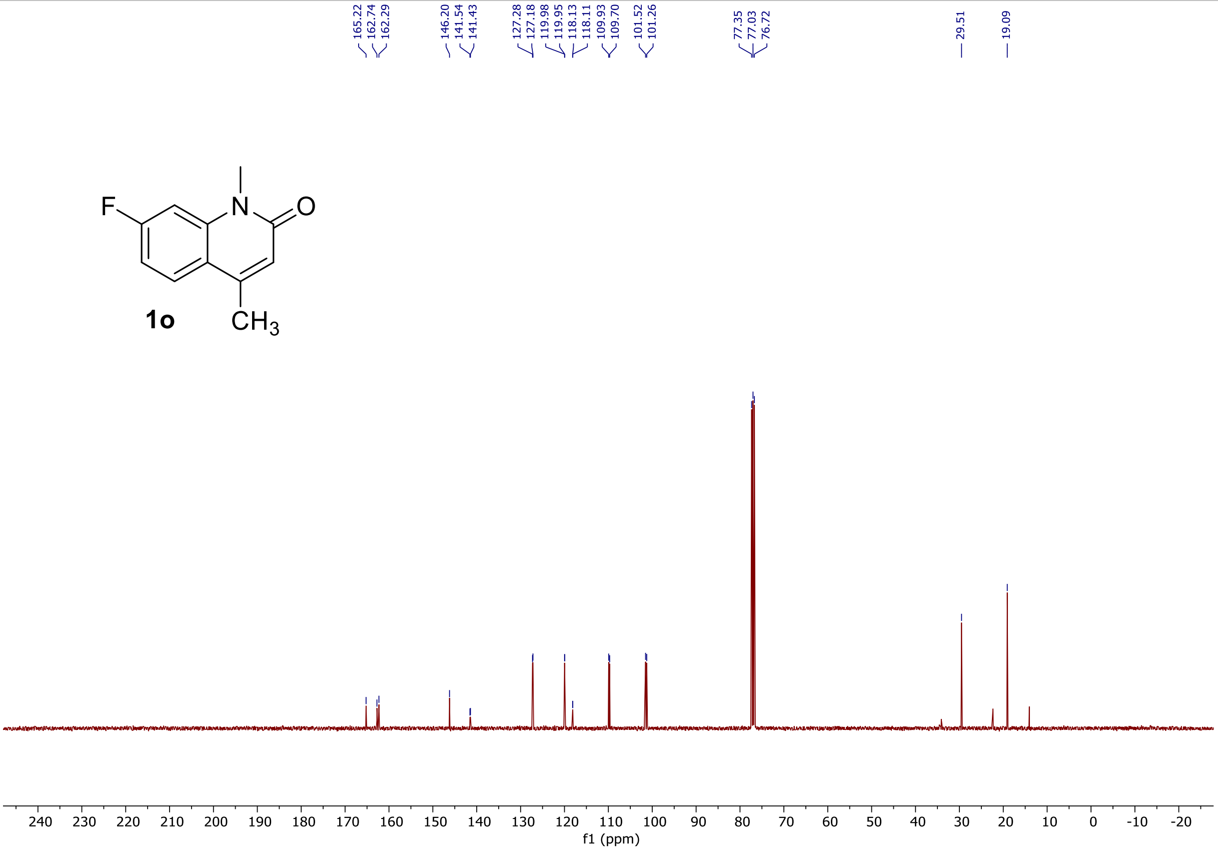
165.22
162.74
162.29
146.20
141.54
141.43
127.28
127.18
119.98
119.95
118.13
118.11
109.93
109.70
101.52
101.26
77.35
77.03
76.72
29.51
19.09
F
N
O
1o
CH3
240 230 220 210 200 190 180 170 160 150 140 130 120 110 100 90 80 70 60 50 40 30 20 10 0 -10 -20
f1 (ppm)

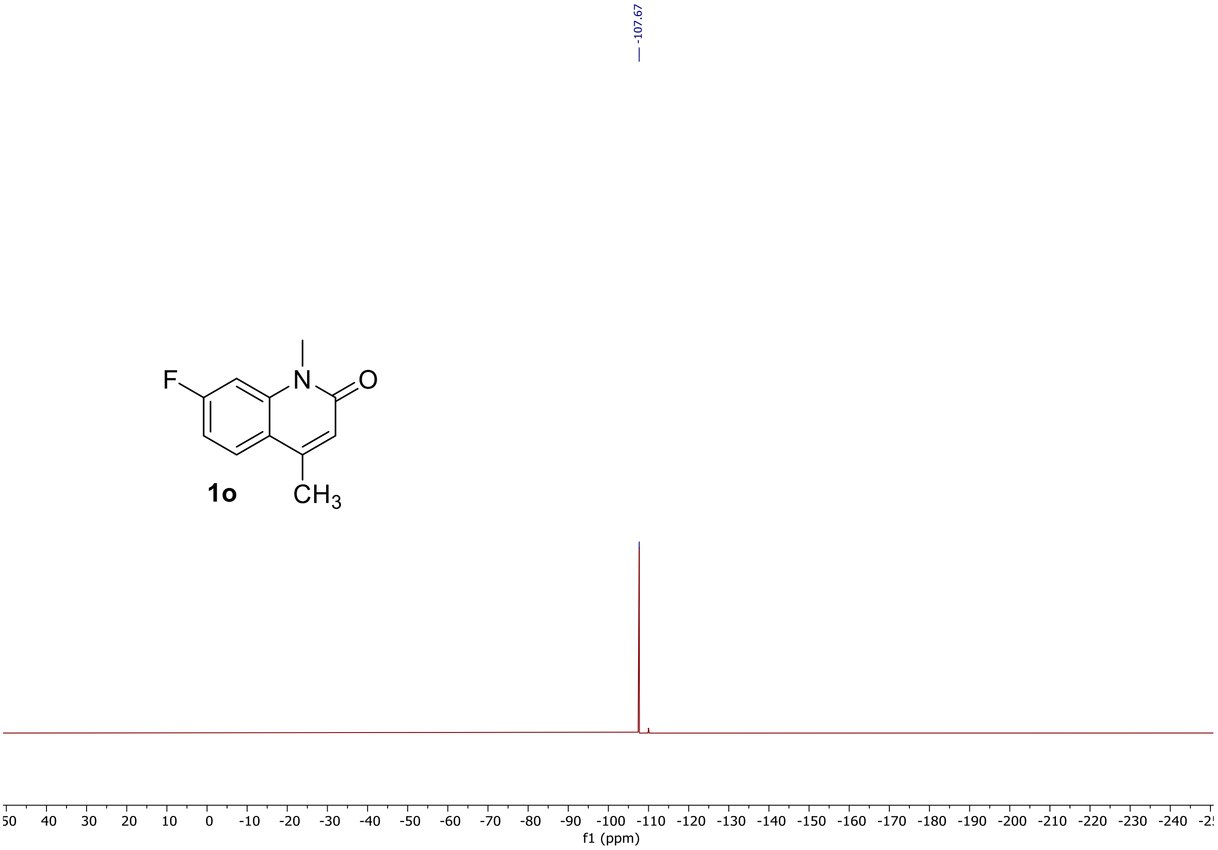
-107.67
F
N
O
1o
CH3
50 40 30 20 10 0 -10 -20 -30 -40 -50 -60 -70 -80 -90 -100 -110 -120 -130 -140 -150 -160 -170 -180 -190 -200 -210 -220 -230 -240
f1 (ppm)

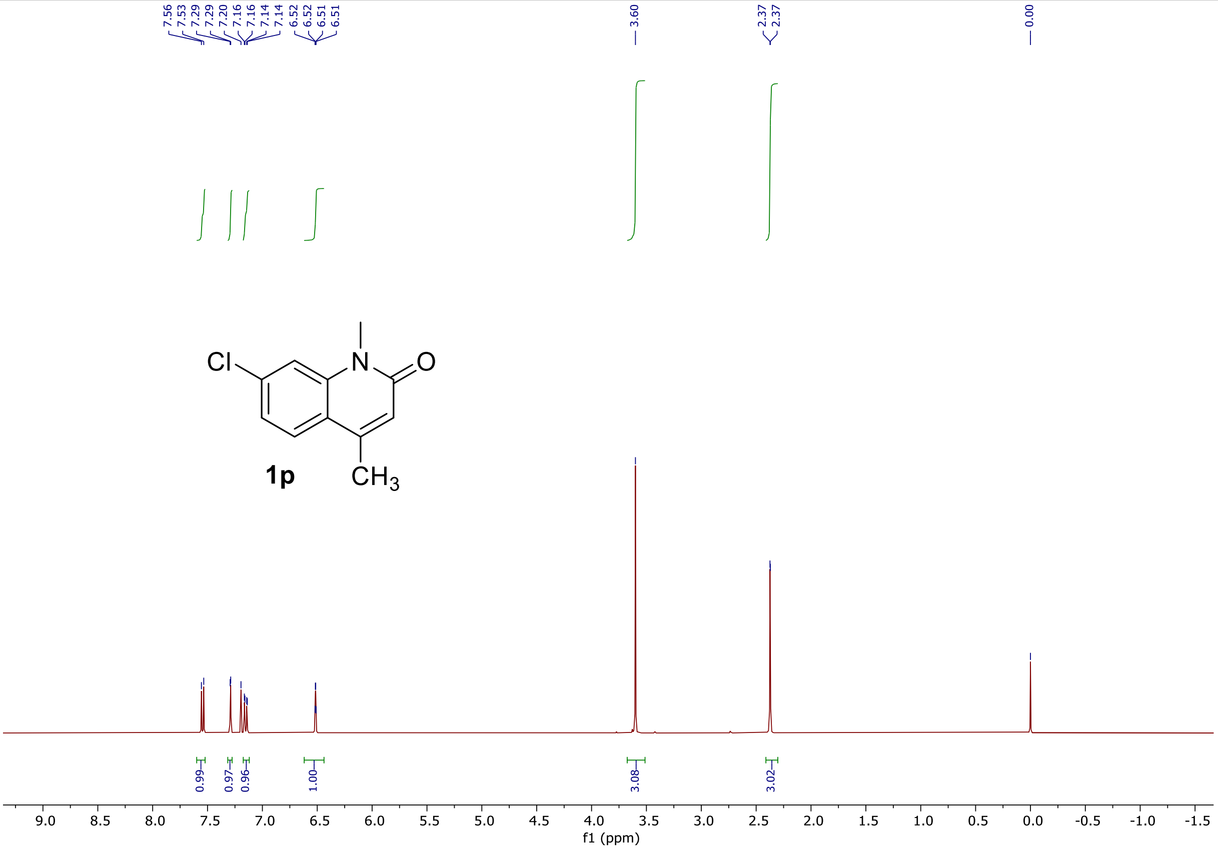
7.56
7.53
7.29
7.29
7.20
7.16
7.16
7.14
7.14
6.52
6.52
6.51
6.51
3.60
2.37
2.37
0.00
Cl
N
O
1p
CH3
0.99
0.97
0.96
1.00
3.08
3.02
f1 (ppm)

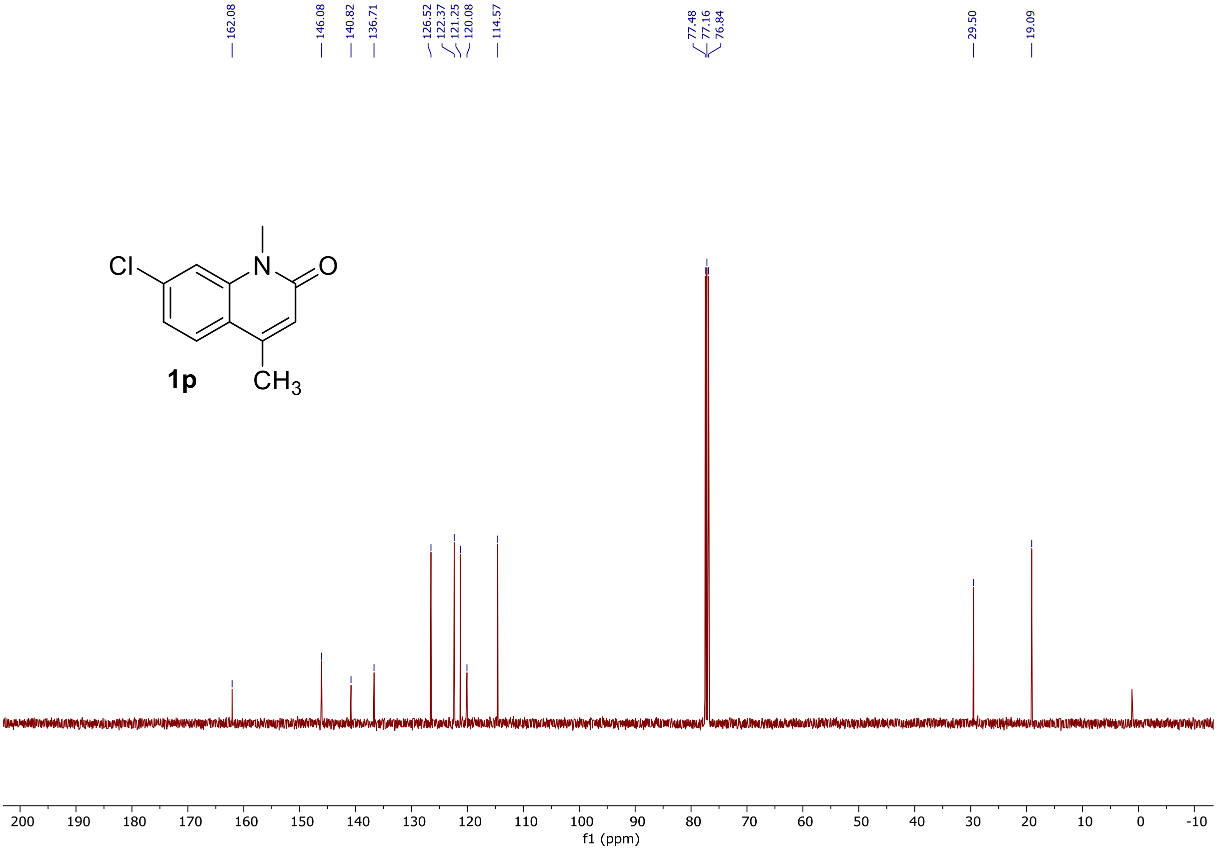
162.08
146.08
140.82
136.71
126.52
122.37
121.25
120.08
114.57
77.48
77.16
76.84
29.50
19.09
Cl
N
O
1p
CH3
f1 (ppm)

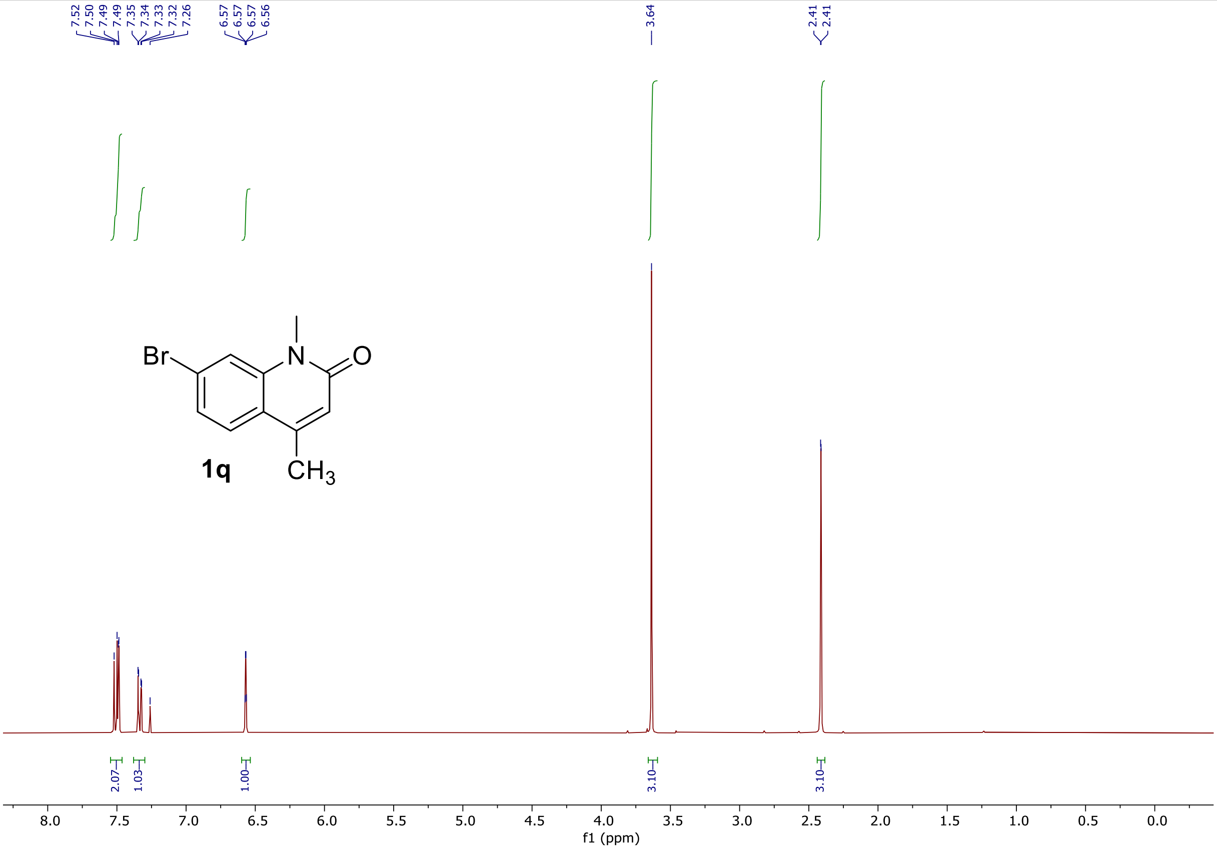
7.52 7.50 7.49 7.49 7.35 7.34 7.33 7.32 7.26 6.57 6.57 6.57 6.56
3.64
2.41 2.41
Br
N
O
1q
CH3
2.07 1.03 1.00 3.10 3.10
f1 (ppm)

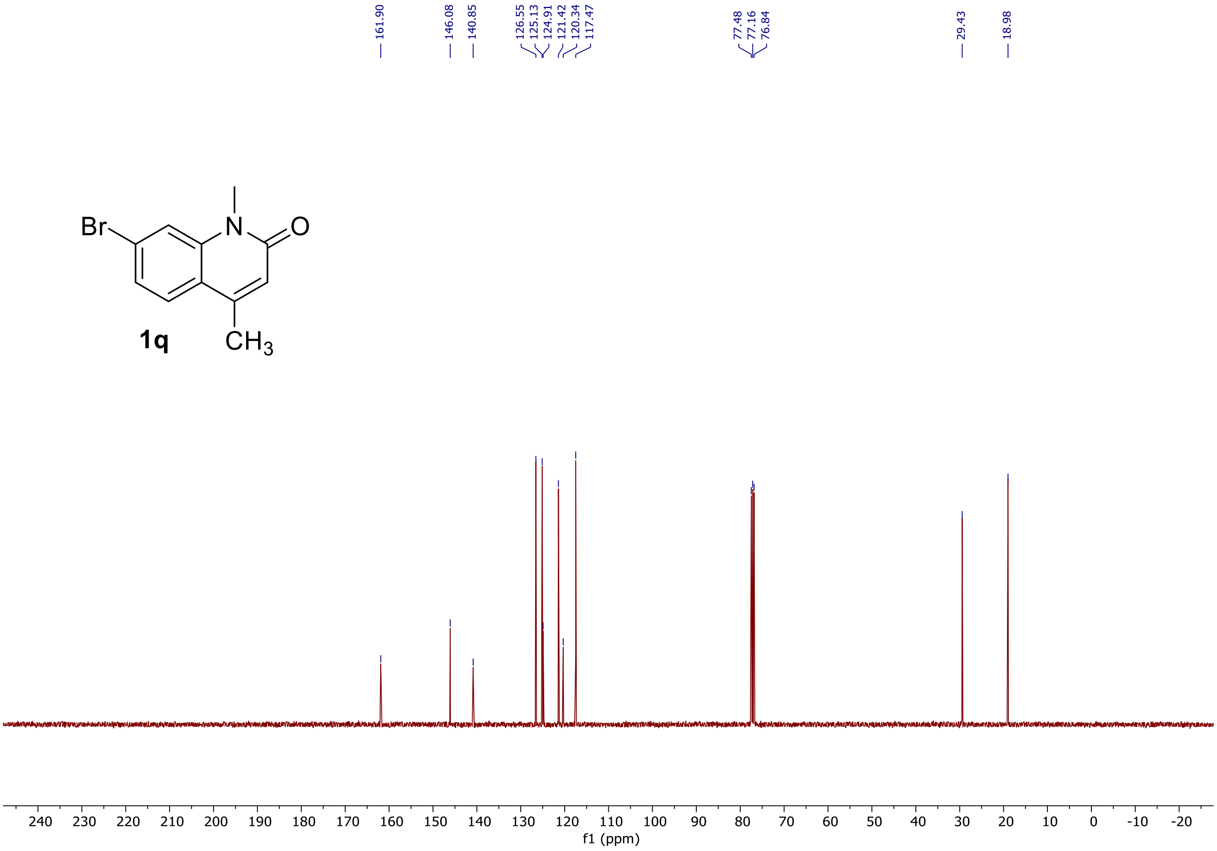
161.90
146.08 140.85
126.55 125.13 124.91 121.42 120.34 117.47
77.48 77.16 76.84
29.43
18.98
Br
N
O
1q
CH3
f1 (ppm)

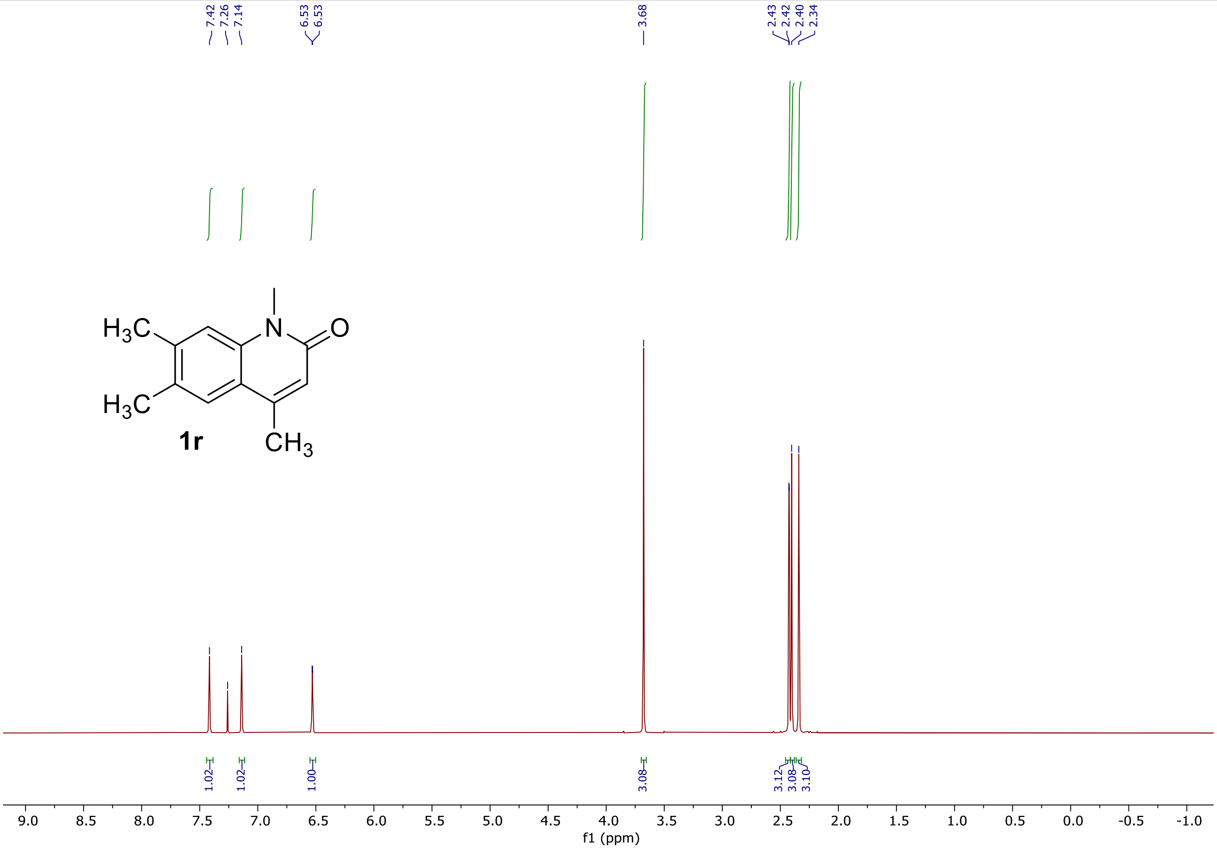
7.42
7.26
7.14
6.53
6.53
3.68
2.43
2.42
2.40
2.34
H3C
H3C
N
O
CH3
1r
1.02
1.02
1.00
3.08
3.12
3.08
3.10
f1 (ppm)

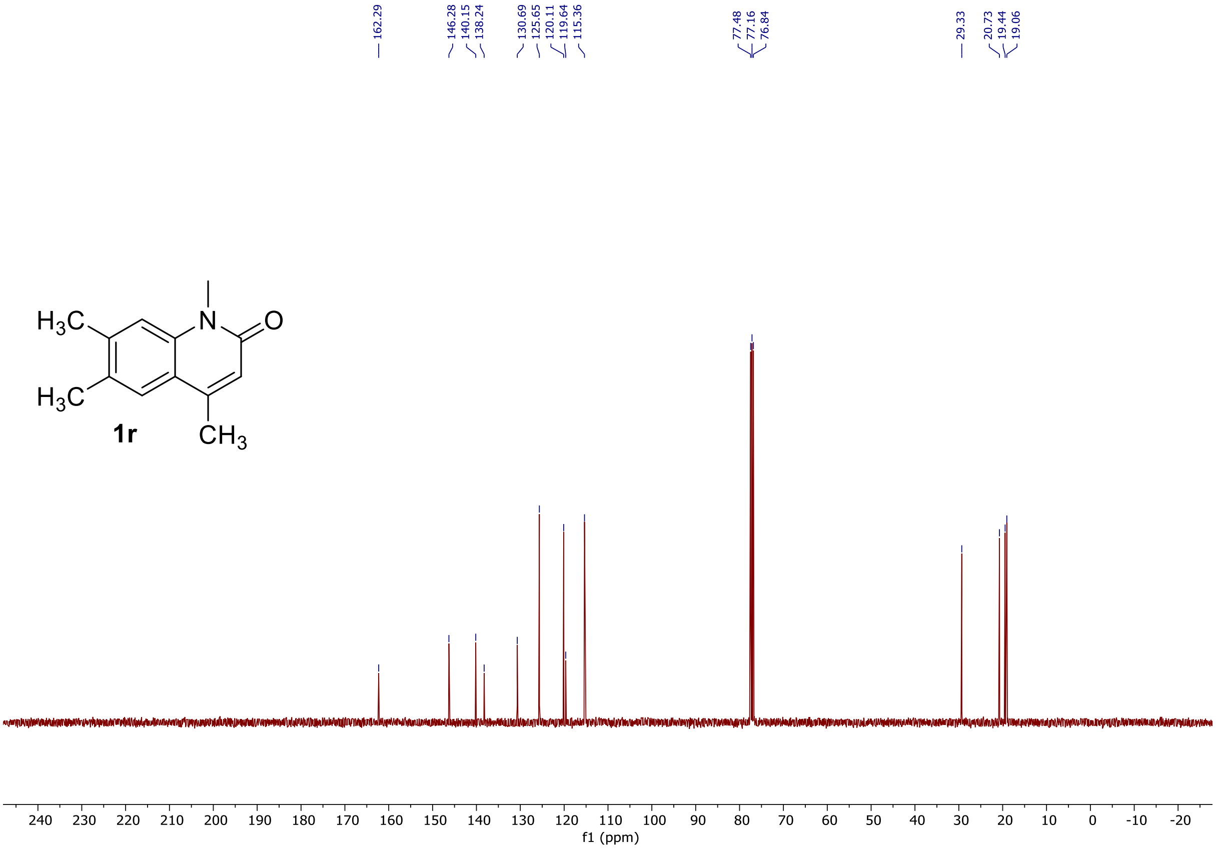
162.29
146.28
140.15
138.24
130.69
125.65
120.11
119.64
115.36
77.48
77.16
76.84
29.33
20.73
19.44
19.06
H3C
H3C
N
O
CH3
1r
f1 (ppm)

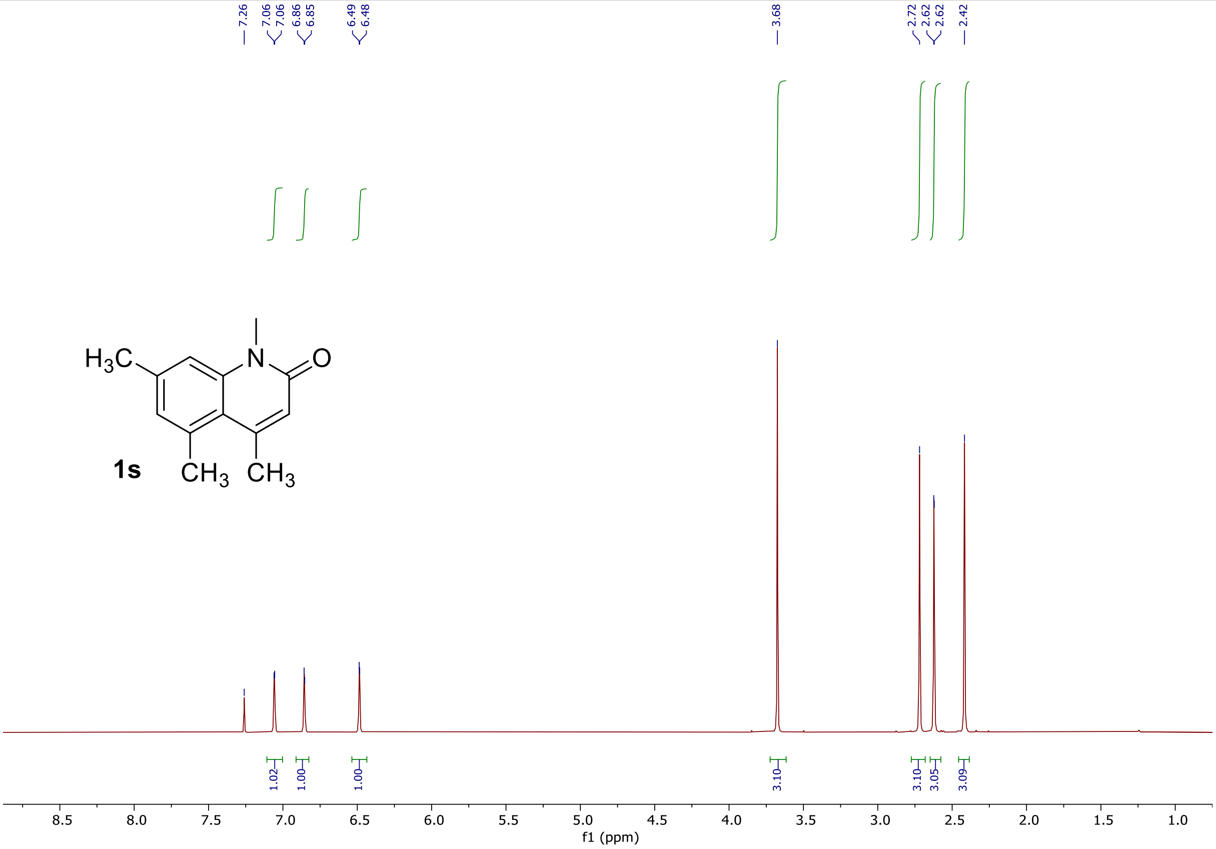
7.26
7.06
7.06
6.86
6.85
6.49
6.48
3.68
2.72
2.62
2.62
2.42
H3C
N
O
1s
CH3 CH3
1.02
1.00
1.00
3.10
3.10
3.05
3.09
8.5 8.0 7.5 7.0 6.5 6.0 5.5 5.0 4.5 4.0 3.5 3.0 2.5 2.0 1.5 1.0
f1 (ppm)

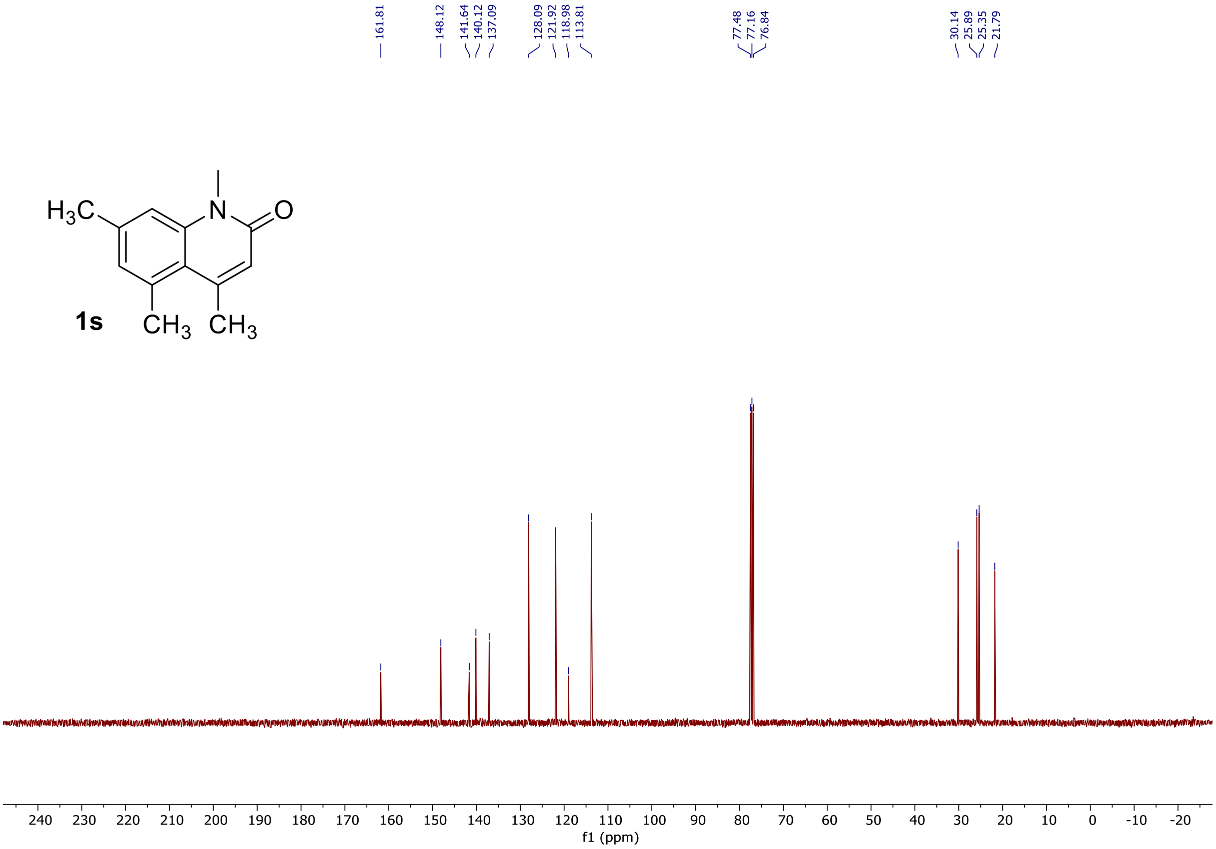
161.81
148.12
141.64
140.12
137.09
128.09
121.92
118.98
113.81
77.48
77.16
76.84
30.14
25.89
25.35
21.79
H3C
N
O
1s
CH3 CH3
240 230 220 210 200 190 180 170 160 150 140 130 120 110 100 90 80 70 60 50 40 30 20 10 0 -10 -20
f1 (ppm)

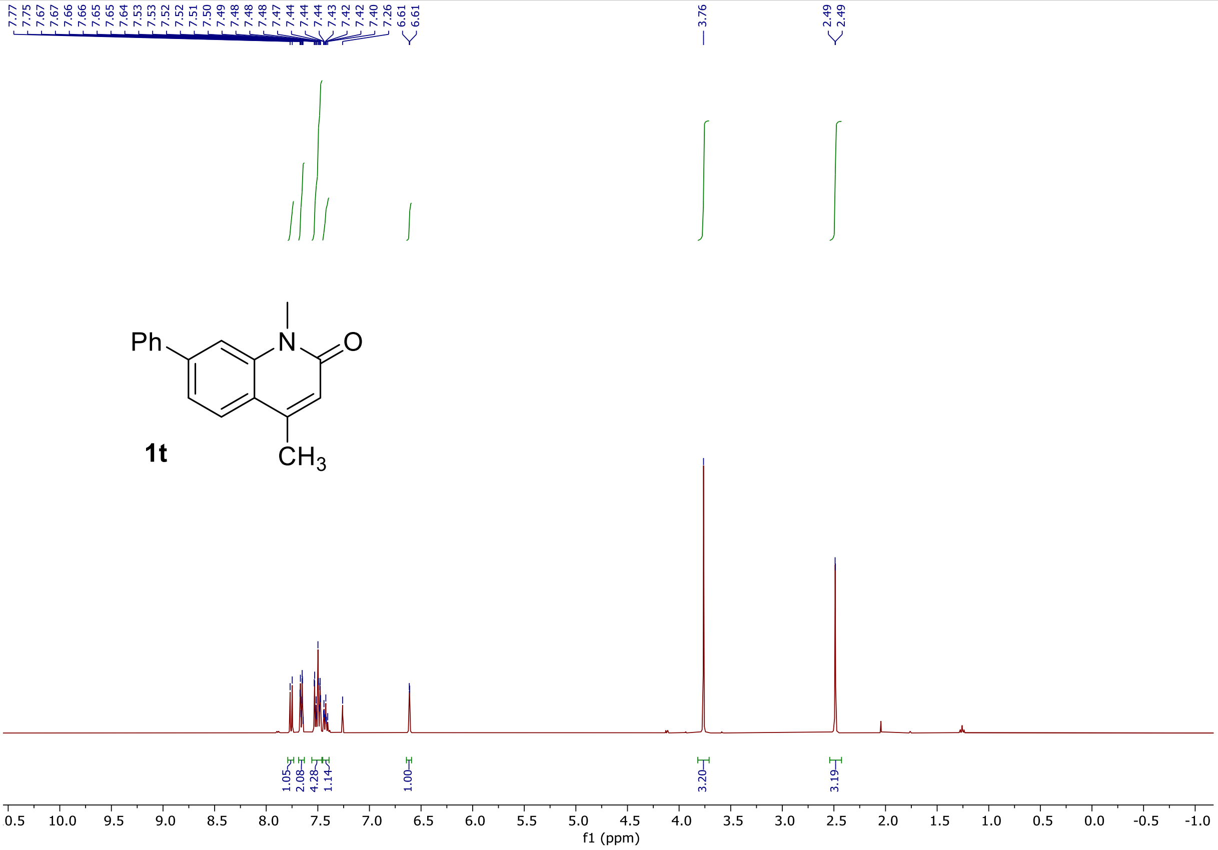
7.77 7.75 7.67 7.67 7.66 7.66 7.65 7.65 7.64 7.53 7.53 7.52 7.52 7.51 7.50 7.49 7.48 7.48 7.48 7.47 7.44 7.44 7.44 7.43 7.42 7.42 7.40 7.26 6.61 6.61
3.76
2.49 2.49
Ph
N
O
1t
CH3
1.05 2.08 4.28 1.14
1.00
3.20
3.19
f1 (ppm)

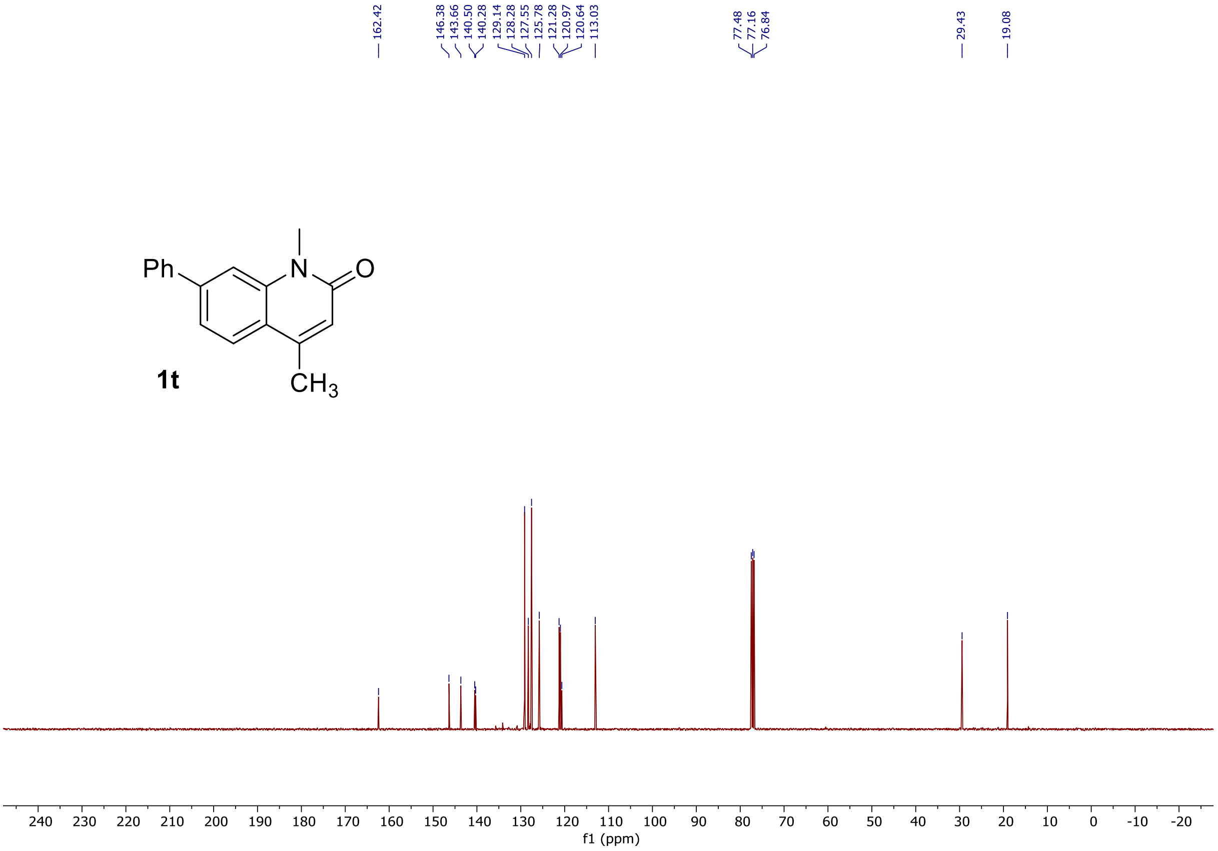
162.42
146.38 143.66 140.50 140.28 129.14 128.28 127.55 125.78 121.28 120.97 120.64 113.03
77.48 77.16 76.84
29.43
19.08
Ph
N
O
1t
CH3
f1 (ppm)

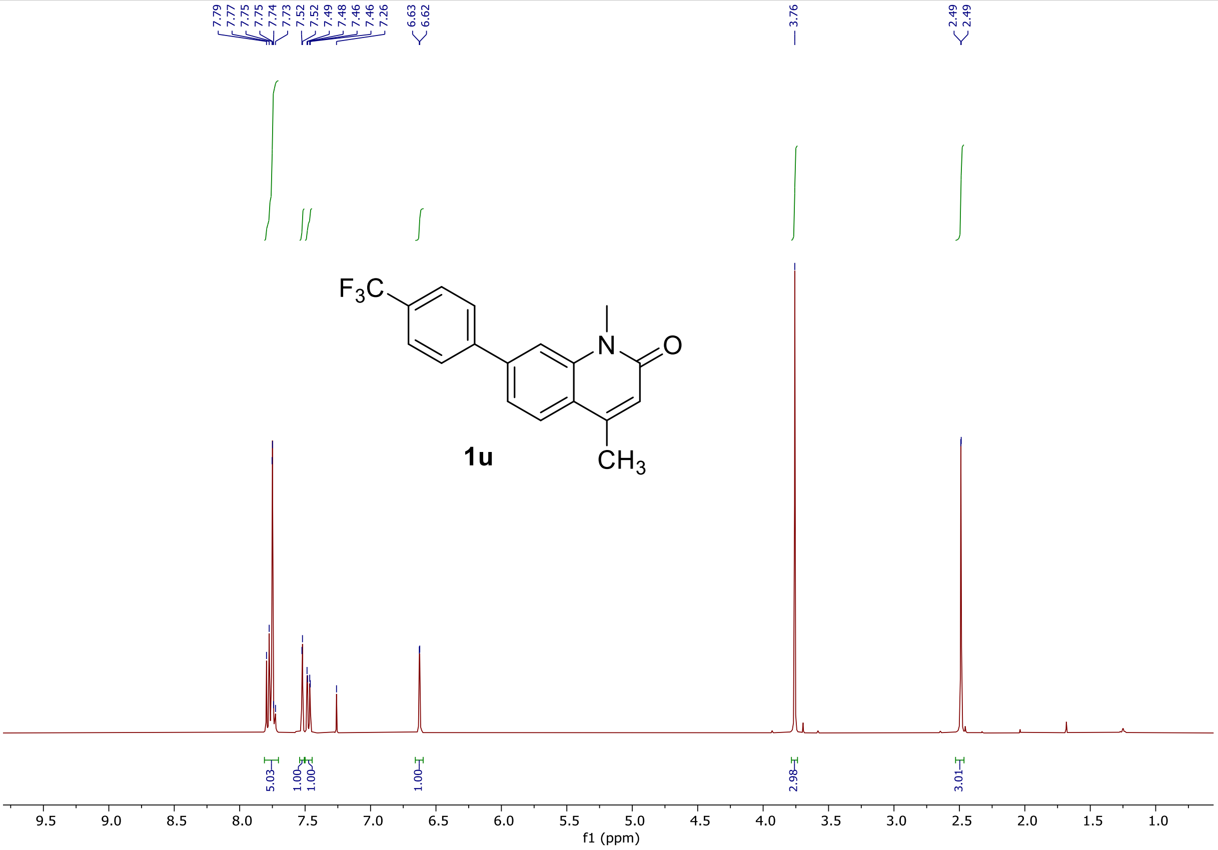
7.79
7.77
7.75
7.75
7.74
7.73
7.52
7.52
7.49
7.48
7.46
7.46
7.26
6.63
6.62
3.76
2.49
2.49
F3C
N
O
1u
CH3
5.03
1.00
1.00
1.00
2.98
3.01
9.5 9.0 8.5 8.0 7.5 7.0 6.5 6.0 5.5 5.0 4.5 4.0 3.5 3.0 2.5 2.0 1.5 1.0
f1 (ppm)

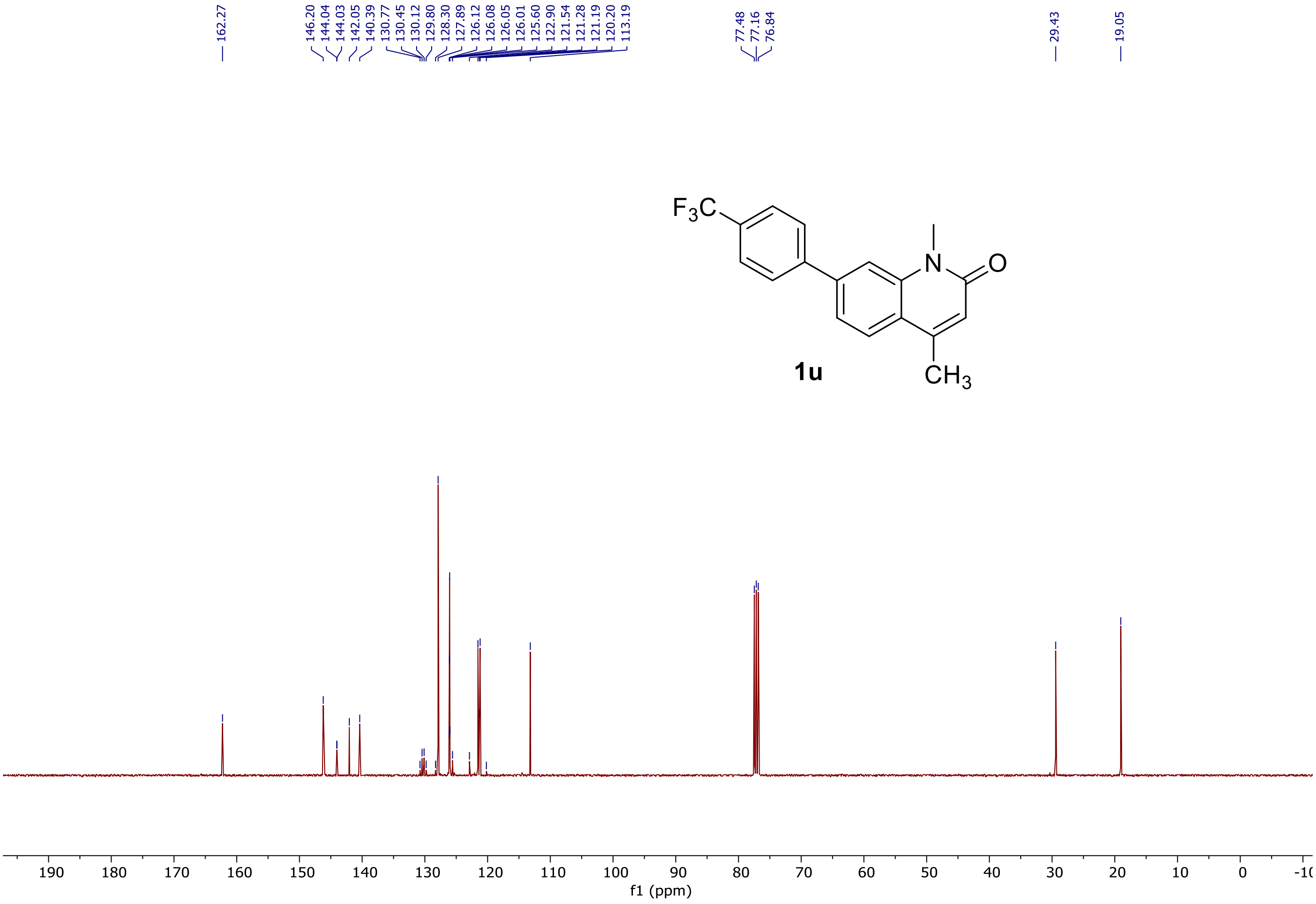
162.27
146.20
144.04
144.03
142.05
140.39
130.77
130.45
130.12
129.80
128.30
127.89
126.12
126.08
126.05
126.01
125.60
122.90
121.54
121.28
121.19
120.20
113.19
77.48
77.16
76.84
29.43
19.05
F3C
N
O
1u
CH3
190 180 170 160 150 140 130 120 110 100 90 80 70 60 50 40 30 20 10 0
f1 (ppm)

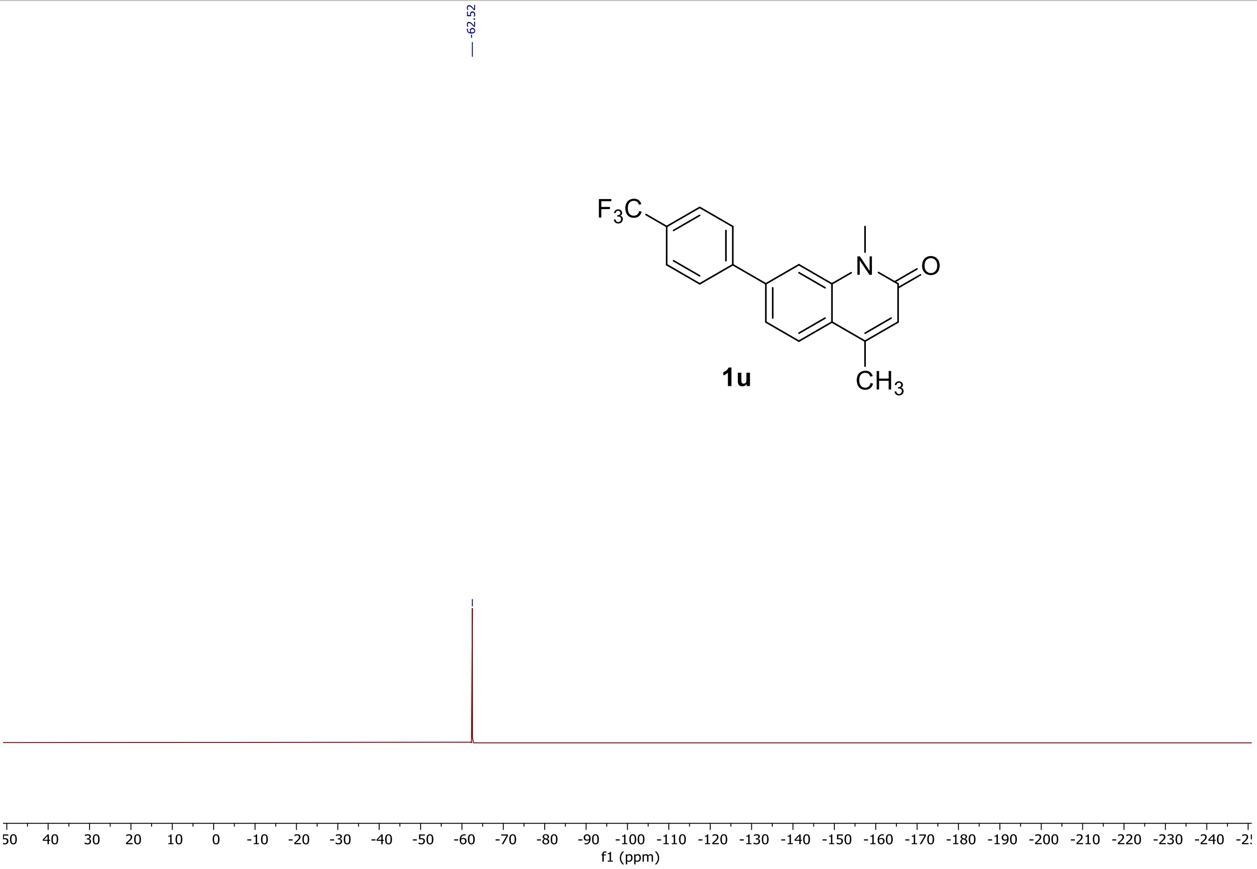
-62.52
F3C
N
O
1u
CH3
f1 (ppm)

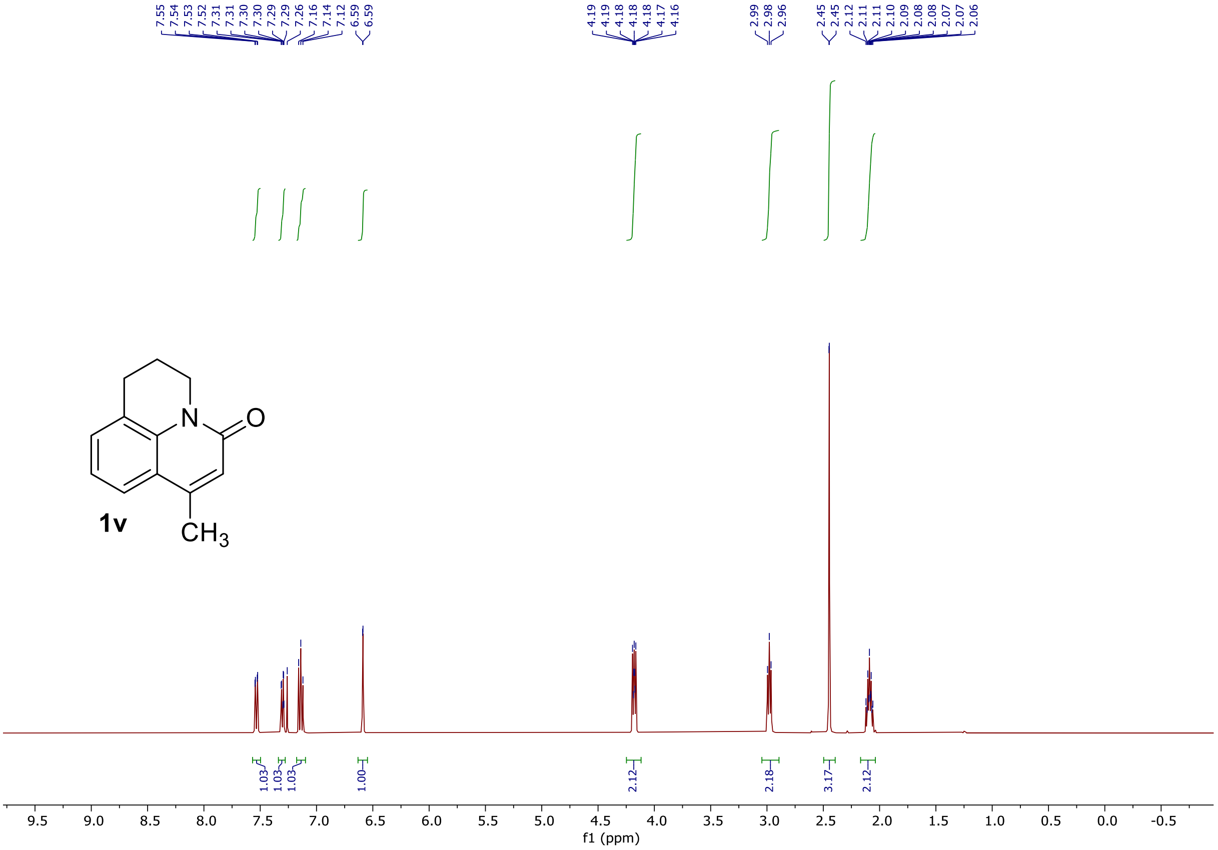
N
O
1v
CH3
1.03
1.03
1.03
1.00
2.12
2.18
3.17
2.12
f1 (ppm)

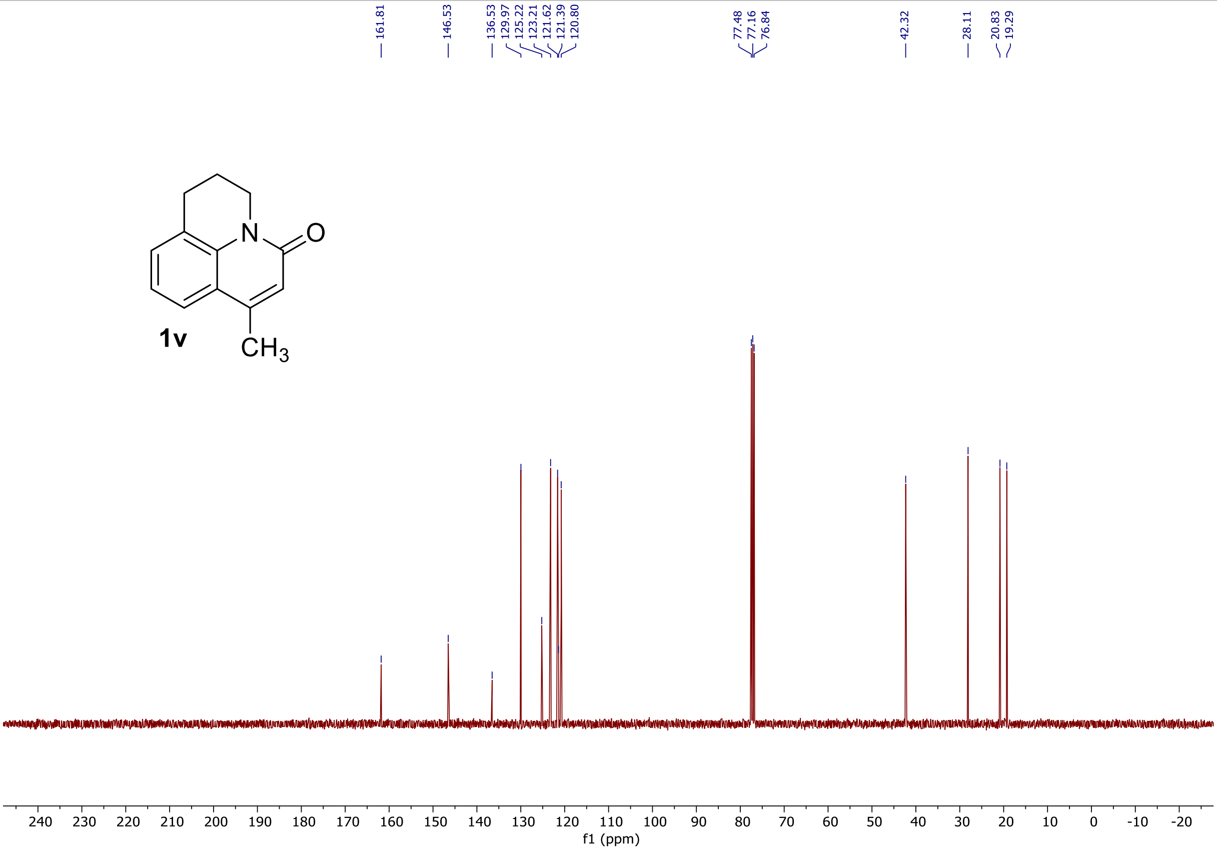
161.81
146.53
136.53
129.97
125.22
123.21
121.62
121.39
120.80
77.48
77.16
76.84
42.32
28.11
20.83
19.29
1v
f1 (ppm)

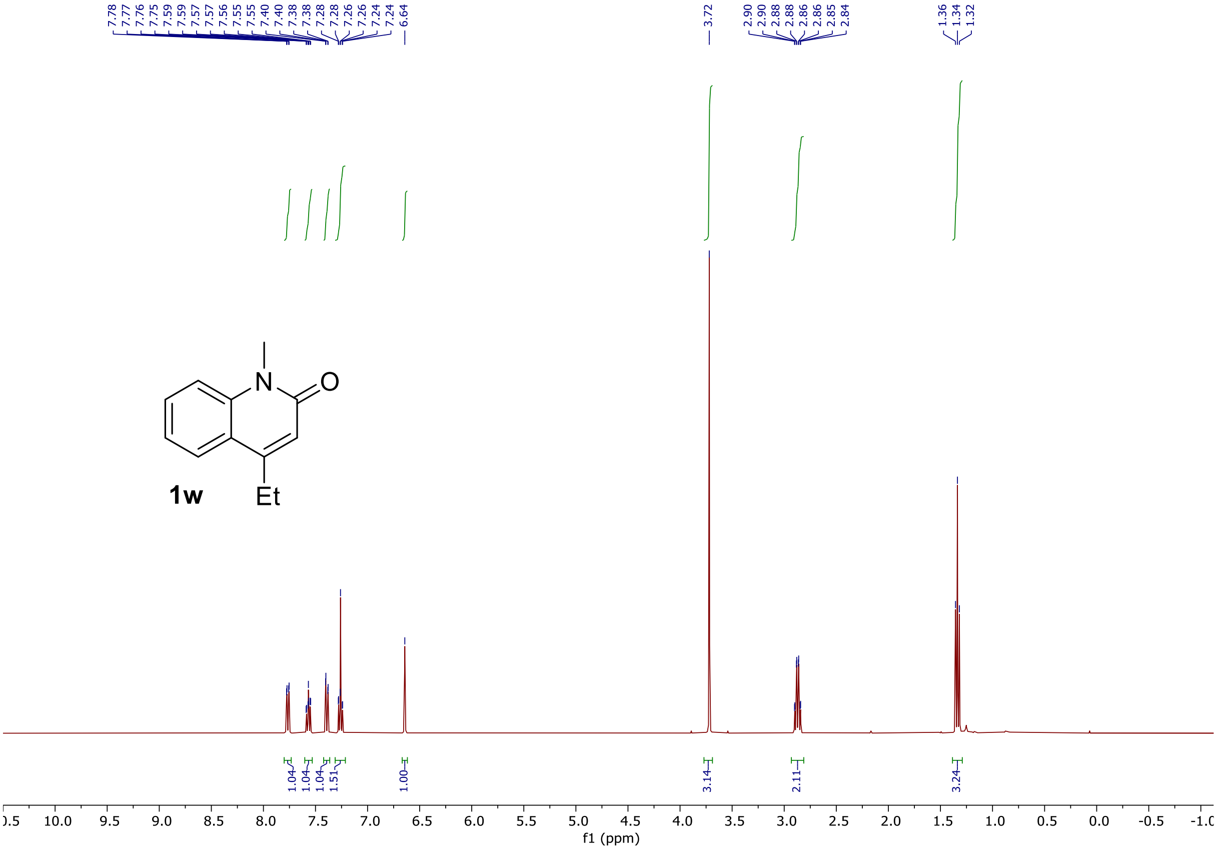
7.78 7.77 7.76 7.75 7.59 7.59 7.57 7.57 7.56 7.55 7.55 7.40 7.40 7.38 7.38 7.28 7.28 7.26 7.26 7.24 7.24
6.64
3.72
2.90 2.90 2.88 2.88 2.86 2.86 2.85 2.84
1.36 1.34 1.32
1.04 1.04 1.04 1.51 1.00 3.14 2.11 3.24
1w
Et
f1 (ppm)

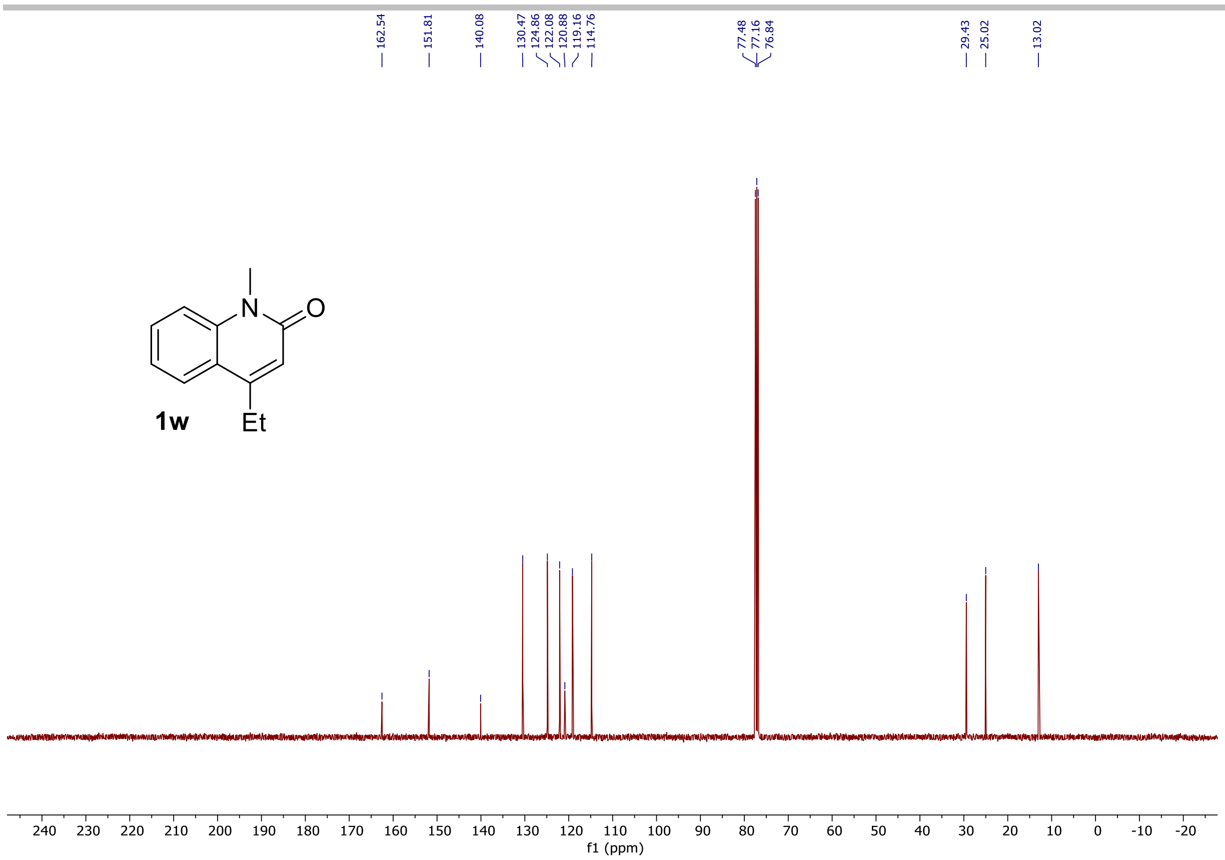
162.54
151.81
140.08
130.47
124.86
122.08
120.88
119.16
114.76
77.48
77.16
76.84
29.43
25.02
13.02
N
O
1w
Et
f1 (ppm)

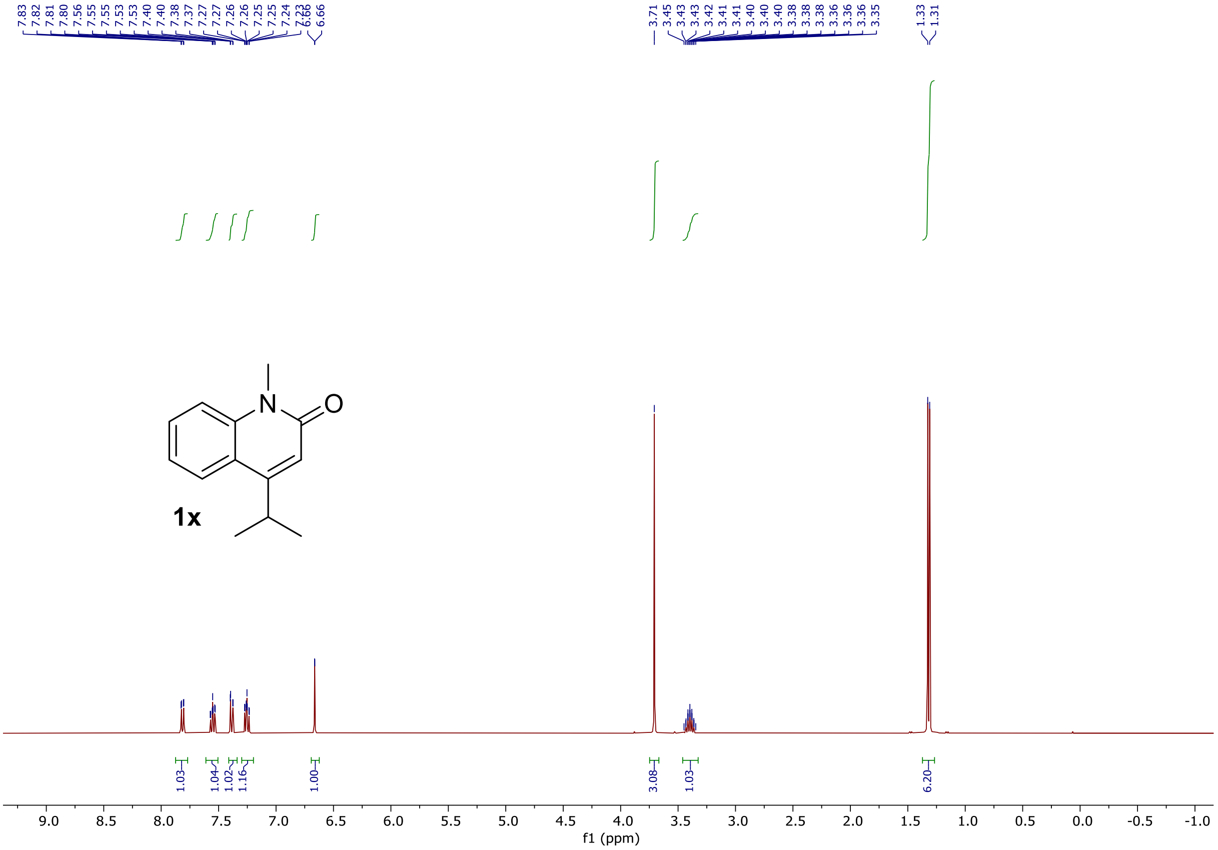
7.83
7.82
7.81
7.80
7.56
7.55
7.55
7.53
7.53
7.40
7.40
7.38
7.37
7.27
7.27
7.26
7.25
7.25
7.24
7.23
6.66
6.66
3.71
3.45
3.43
3.43
3.42
3.41
3.41
3.40
3.40
3.38
3.38
3.38
3.36
3.36
3.35
1.33
1.31
N
O
1x
1.03
1.04
1.02
1.16
1.00
3.08
1.03
6.20
f1 (ppm)

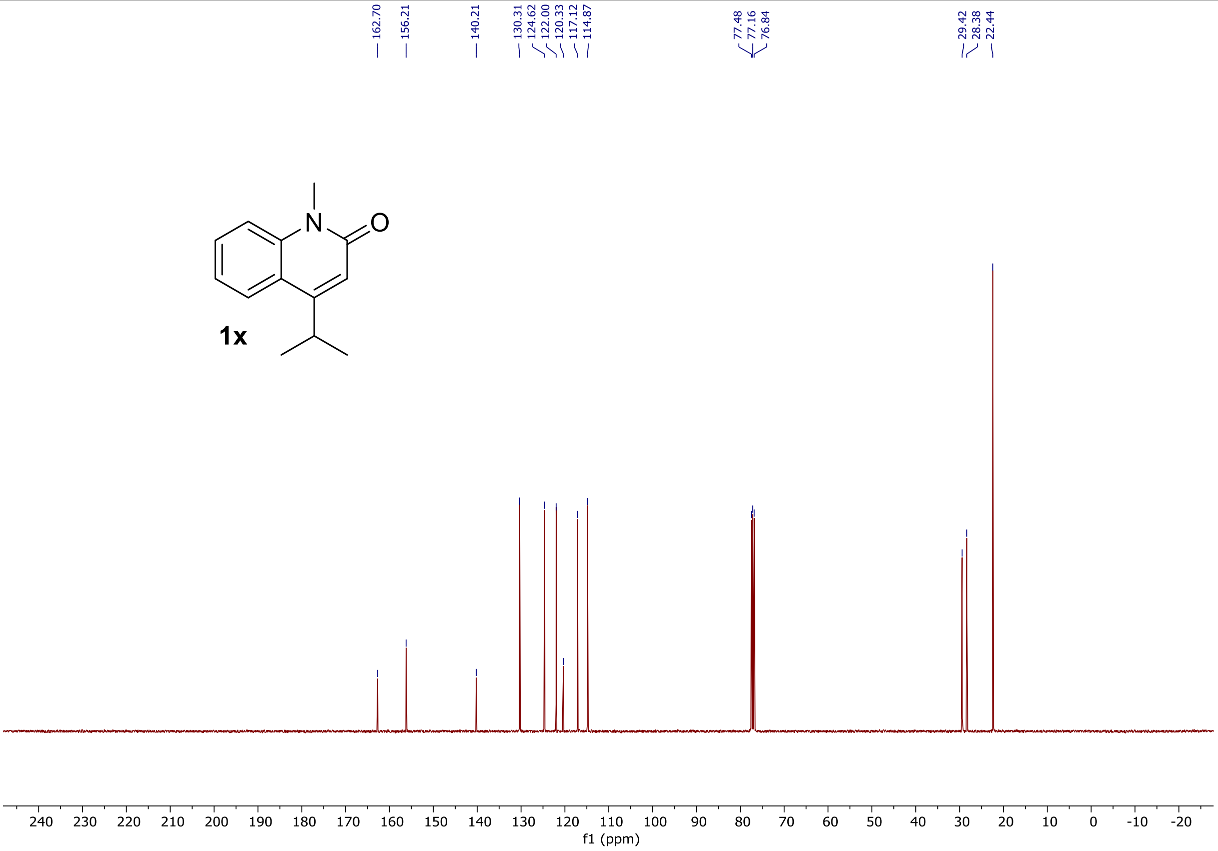

162.70
156.21
140.21
130.31
124.62
122.00
120.33
117.12
114.87
77.48
77.16
76.84
29.42
28.38
22.44
1x
240 230 220 210 200 190 180 170 160 150 140 130 120 110 100 90 80 70 60 50 40 30 20 10 0 -10 -20
f1 (ppm)

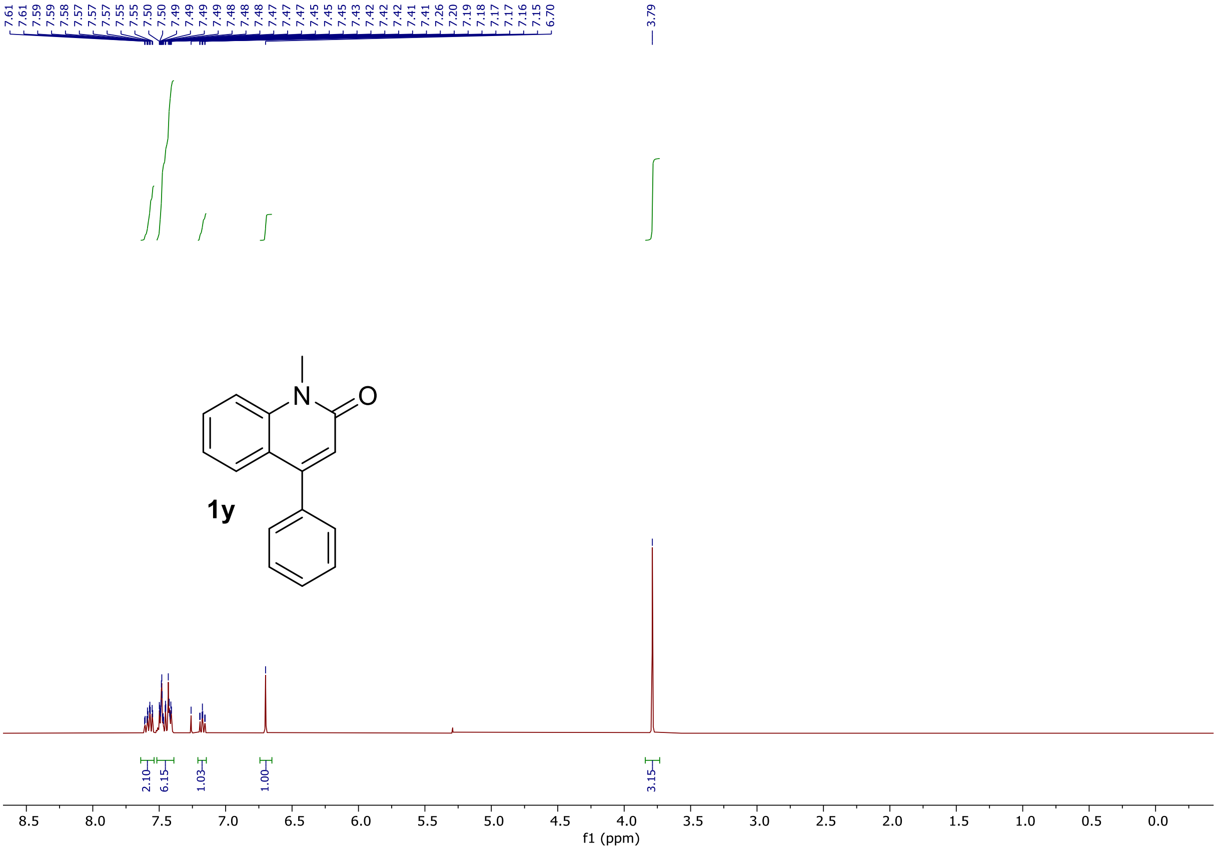

7.61 7.61 7.59 7.59 7.58 7.57 7.57 7.57 7.55 7.55 7.50 7.50 7.49 7.49 7.49 7.49 7.48 7.48 7.48 7.47 7.47 7.47 7.45 7.45 7.45 7.43 7.42 7.42 7.42 7.41 7.41 7.26 7.20 7.19 7.18 7.17 7.17 7.16 7.15 6.70
3.79
1y
2.10
6.15
1.03
1.00
3.15
8.5 8.0 7.5 7.0 6.5 6.0 5.5 5.0 4.5 4.0 3.5 3.0 2.5 2.0 1.5 1.0 0.5 0.0
f1 (ppm)

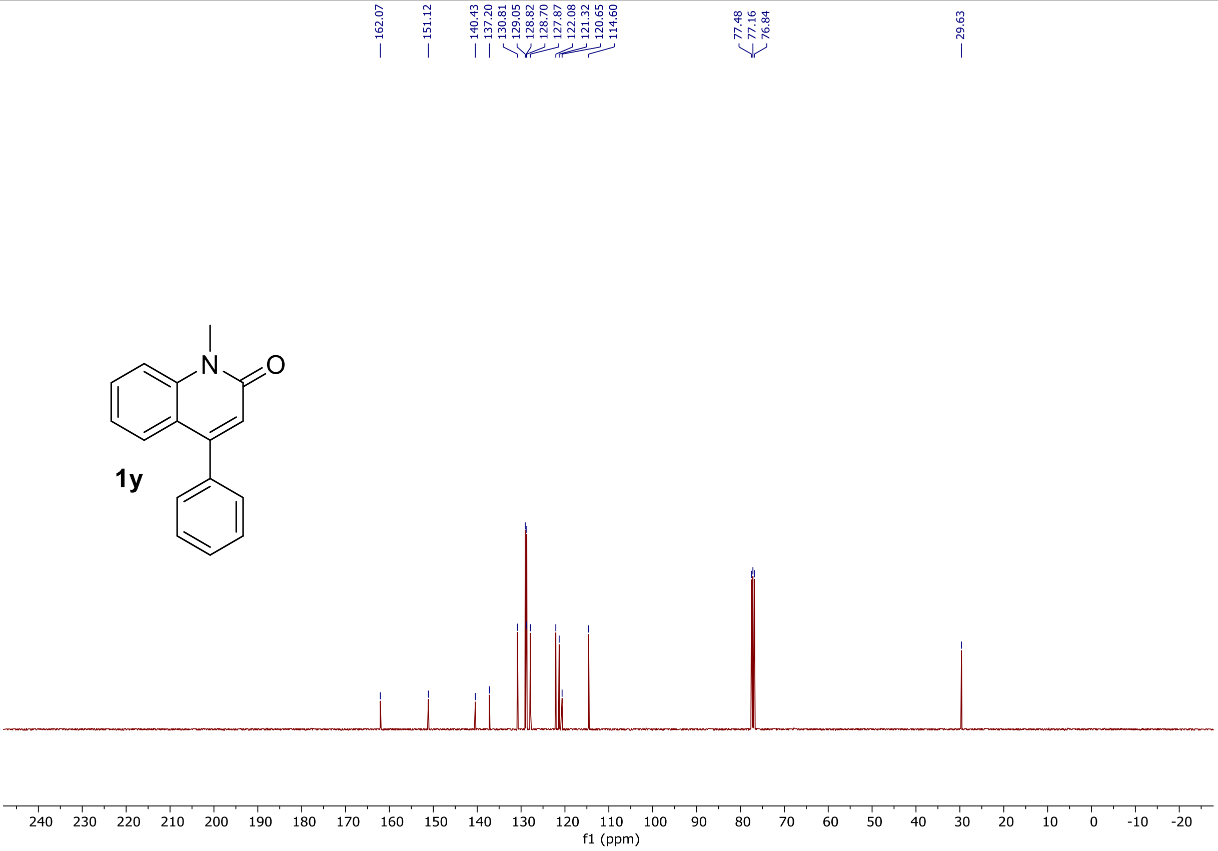
162.07
151.12
140.43
137.20
130.81
129.05
128.82
128.70
127.87
122.08
121.32
120.65
114.60
77.48
77.16
76.84
29.63
1y
f1 (ppm)

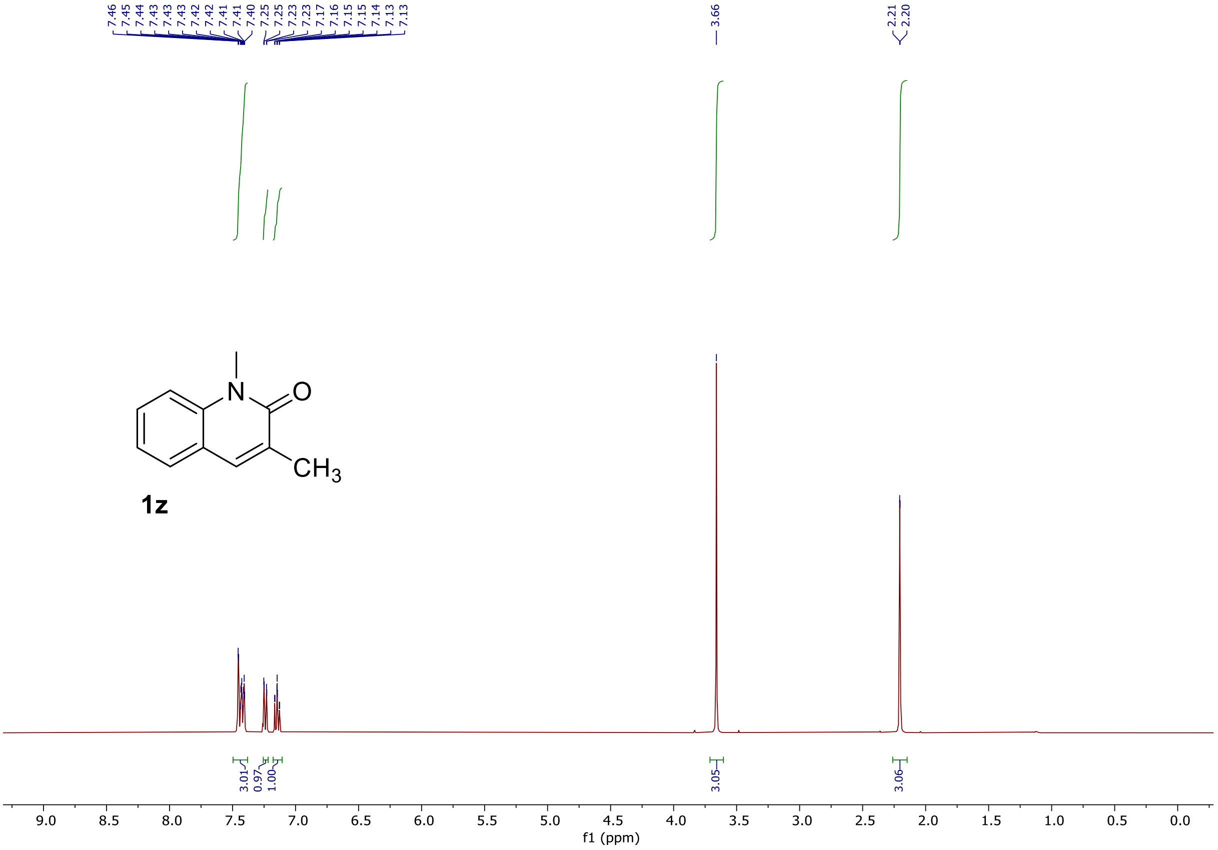
7.46
7.45
7.44
7.43
7.43
7.43
7.42
7.41
7.40
7.25
7.25
7.23
7.23
7.17
7.16
7.15
7.14
7.13
7.13
3.66
2.21
2.20
1z
CH3
3.01
0.97
1.00
3.05
3.06
f1 (ppm)

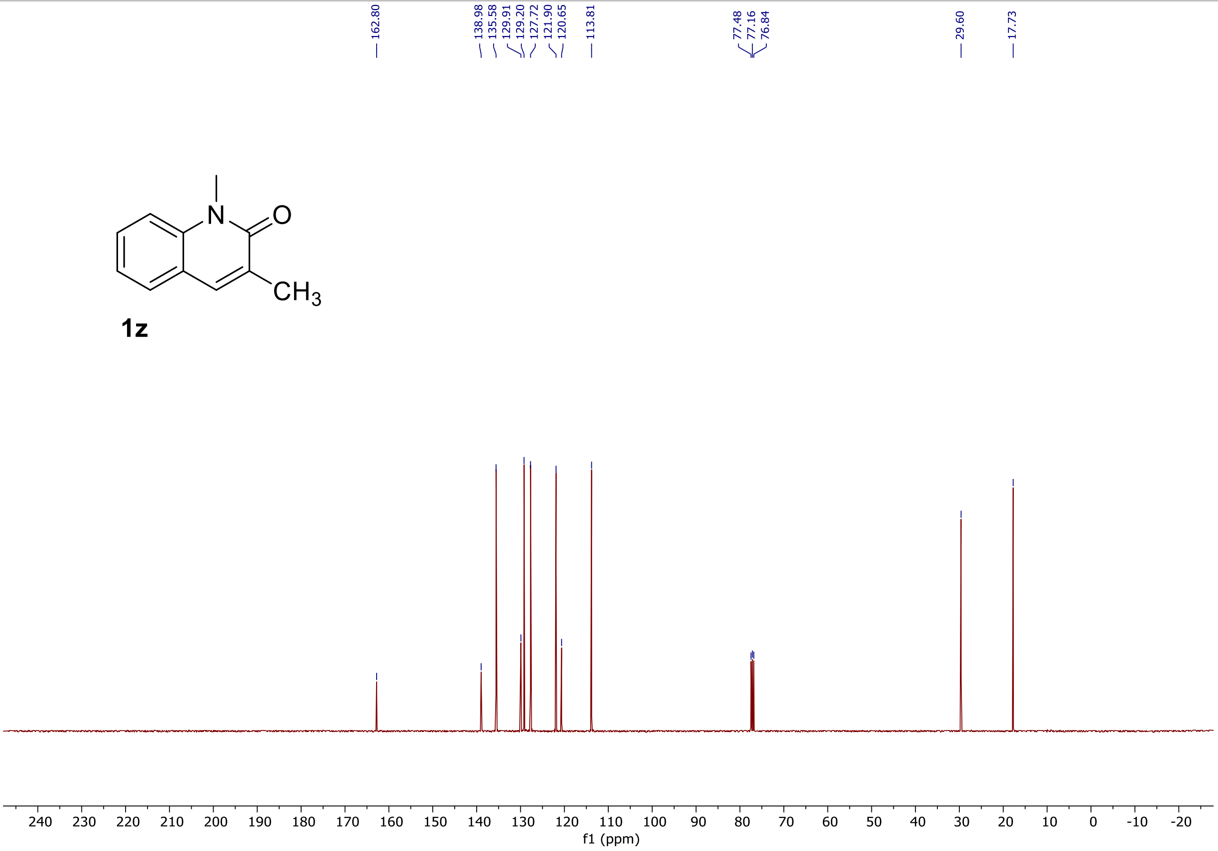
162.80
138.98
135.58
129.91
129.20
127.72
121.90
120.65
113.81
77.48
77.16
76.84
29.60
17.73
1z
f1 (ppm)

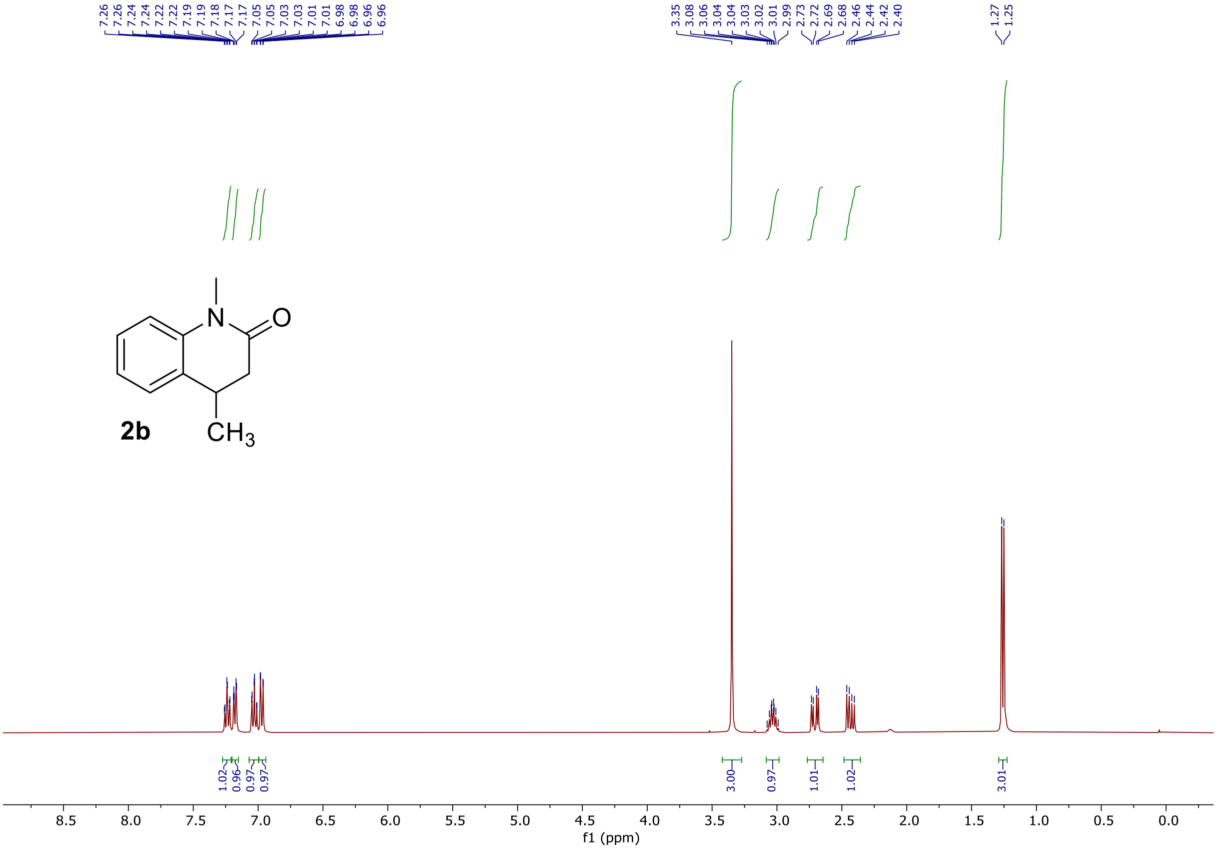
7.26
7.26
7.24
7.24
7.22
7.22
7.19
7.19
7.18
7.17
7.17
7.05
7.05
7.03
7.03
7.01
7.01
6.98
6.98
6.96
6.96
3.35
3.08
3.06
3.04
3.04
3.03
3.02
3.01
2.99
2.73
2.72
2.69
2.68
2.46
2.44
2.42
2.40
1.27
1.25
2b
1.02
0.96
0.97
0.97
3.00
0.97
1.01
1.02
3.01
f1 (ppm)

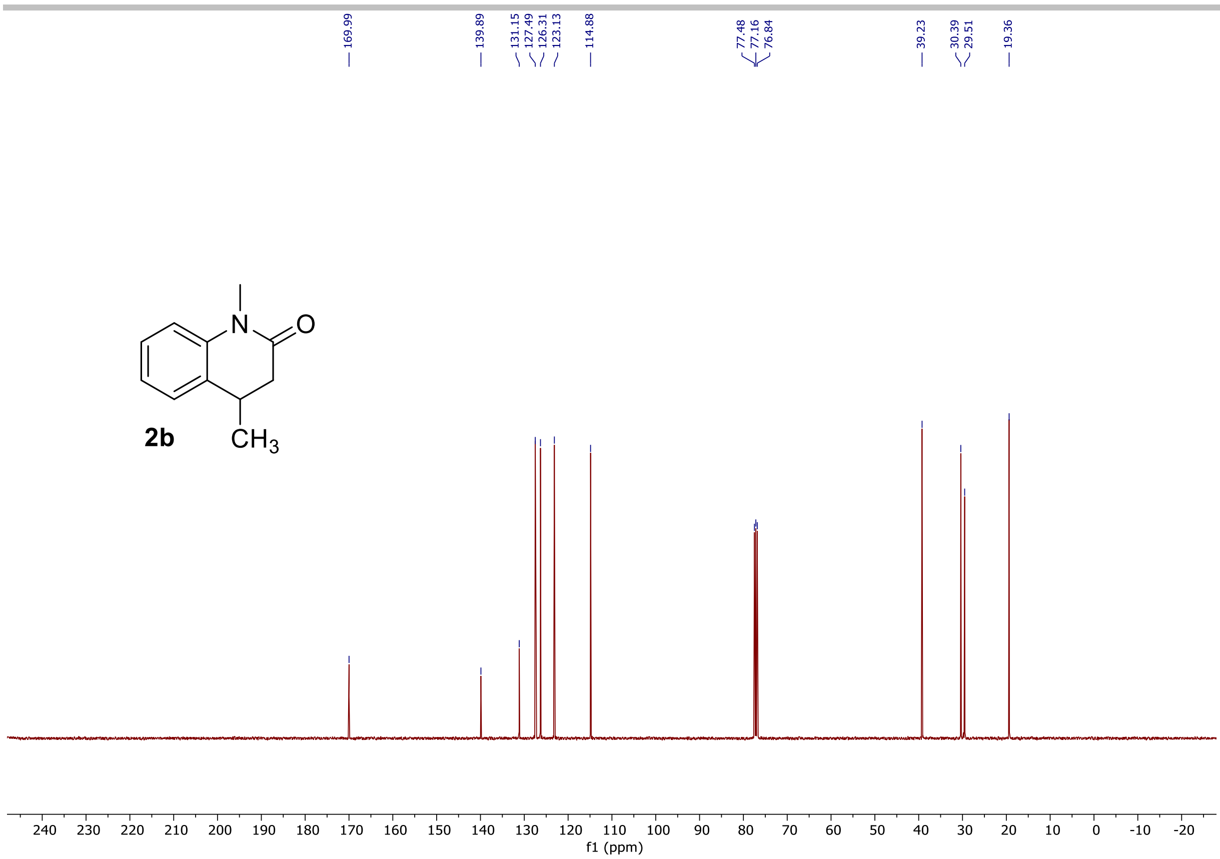
169.99
139.89
131.15
127.49
126.31
123.13
114.88
77.48
77.16
76.84
39.23
30.39
29.51
19.36
2b
CH3
N
O
f1 (ppm)

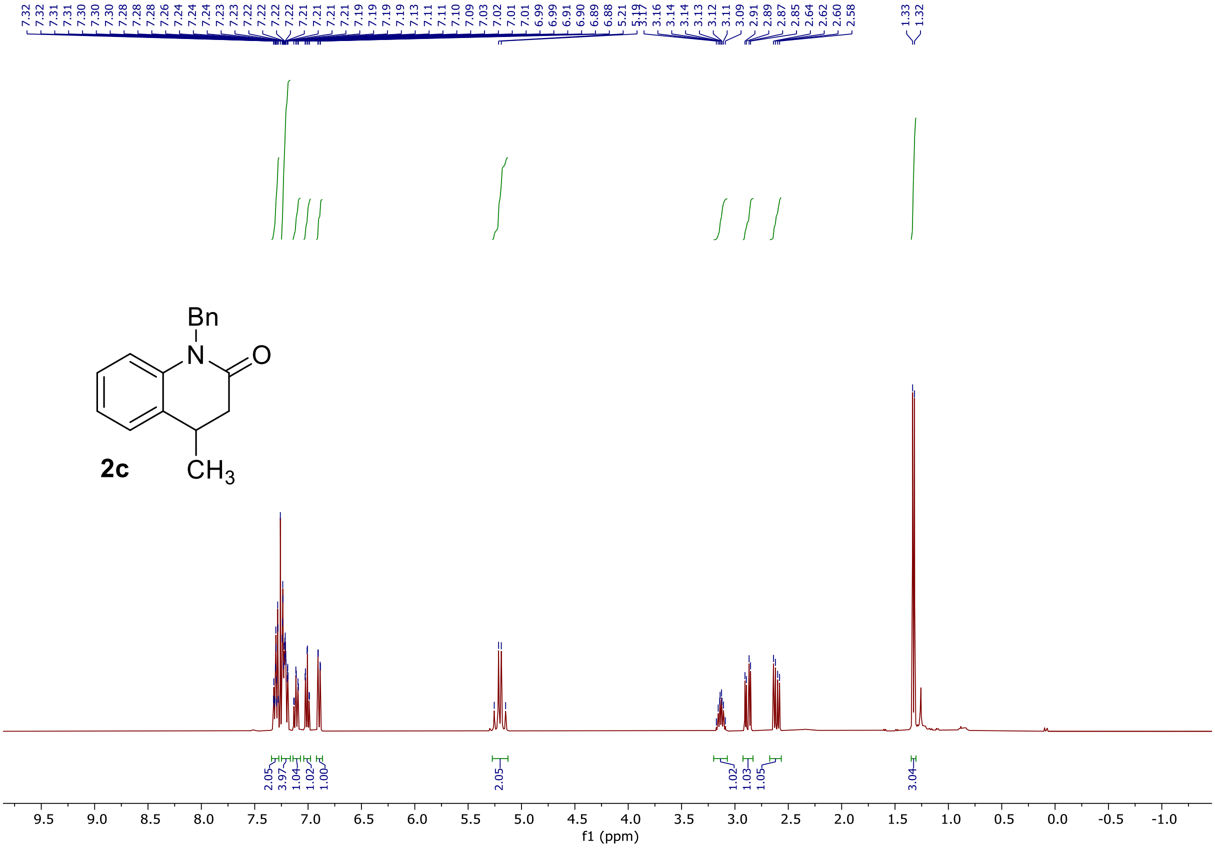
7.32 7.32 7.31 7.31 7.30 7.30 7.30 7.28 7.28 7.28 7.26 7.24 7.24 7.24 7.23 7.23 7.22 7.22 7.22 7.22 7.21 7.21 7.21 7.21 7.19 7.19 7.19 7.19 7.13 7.11 7.11 7.10 7.09 7.03 7.02 7.01 7.01 6.99 6.99 6.91 6.90 6.89 6.88 5.21 5.19 5.17
3.16 3.14 3.14 3.13 3.12 3.11 3.09 2.91 2.89 2.87 2.85 2.64 2.62 2.60 2.58
1.33 1.32
Bn
N
O
2c
CH3
2.05 3.97 1.04 1.02 1.00
2.05
1.02 1.03 1.05
3.04
f1 (ppm)

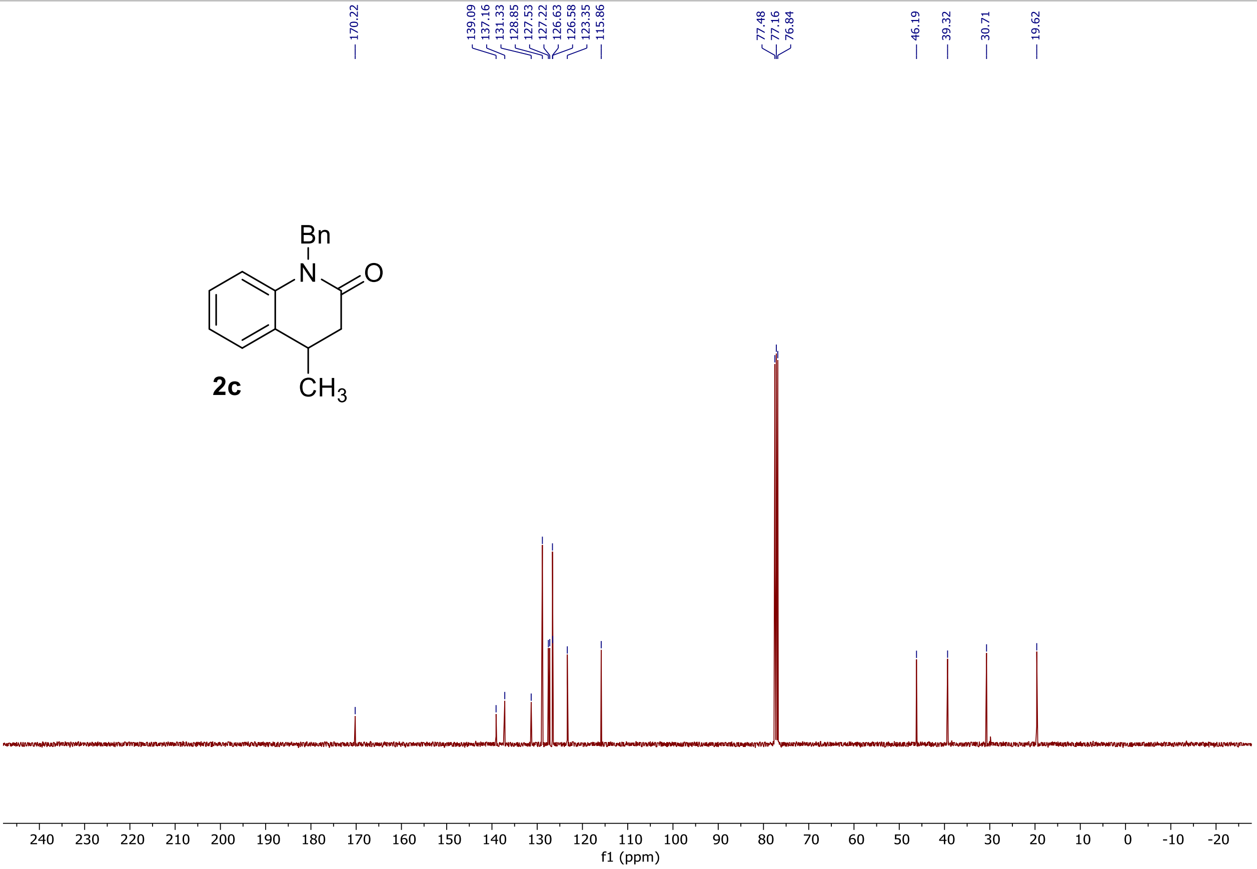
170.22
139.09
137.16
131.33
128.85
127.53
127.22
126.63
126.58
123.35
115.86
77.48
77.16
76.84
46.19
39.32
30.71
19.62
Bn
N
O
2c
CH_3
f1 (ppm)

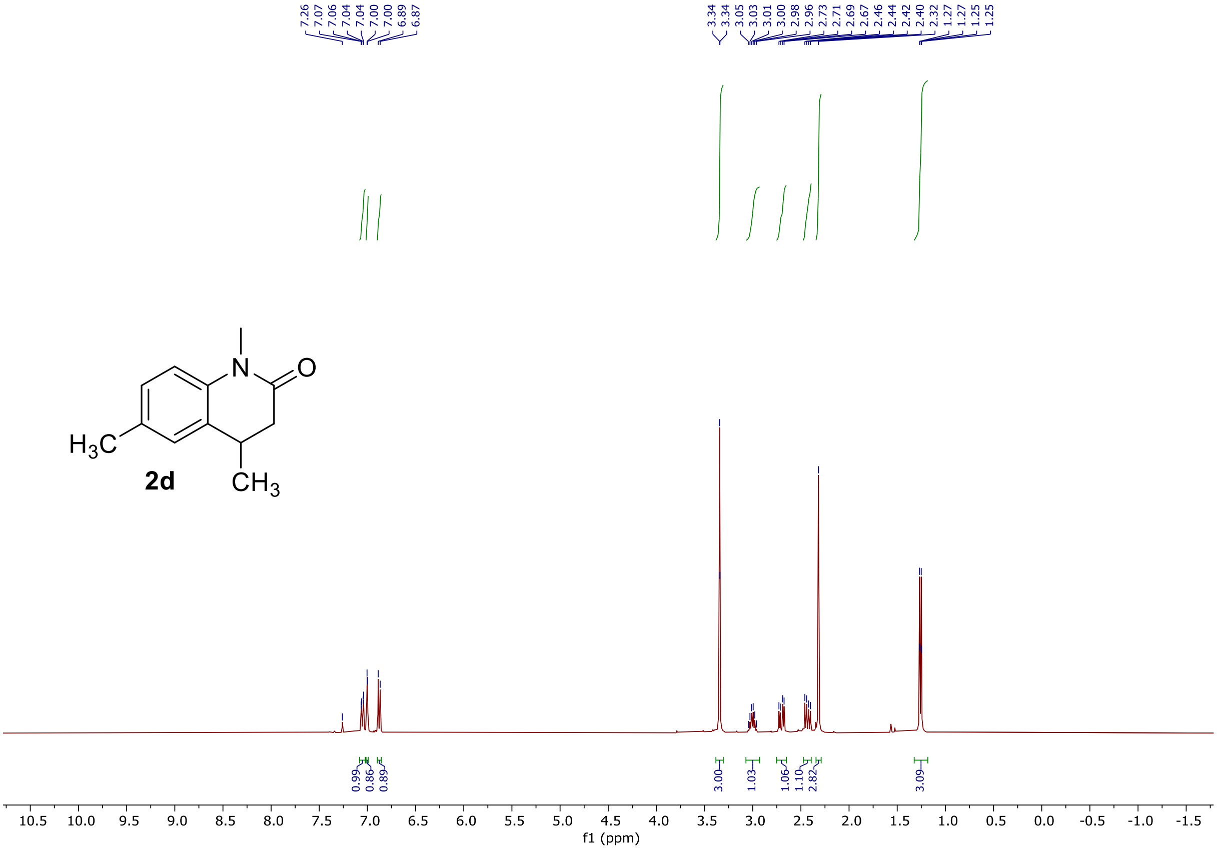
7.26
7.07
7.06
7.04
7.04
7.00
7.00
6.89
6.87
3.34
3.34
3.05
3.03
3.01
3.00
2.98
2.96
2.73
2.71
2.69
2.67
2.46
2.44
2.42
2.40
2.32
1.27
1.27
1.25
1.25
N
O
H_3C
2d
CH_3
0.99
0.86
0.89
3.00
1.03
1.06
1.10
2.82
3.09
f1 (ppm)

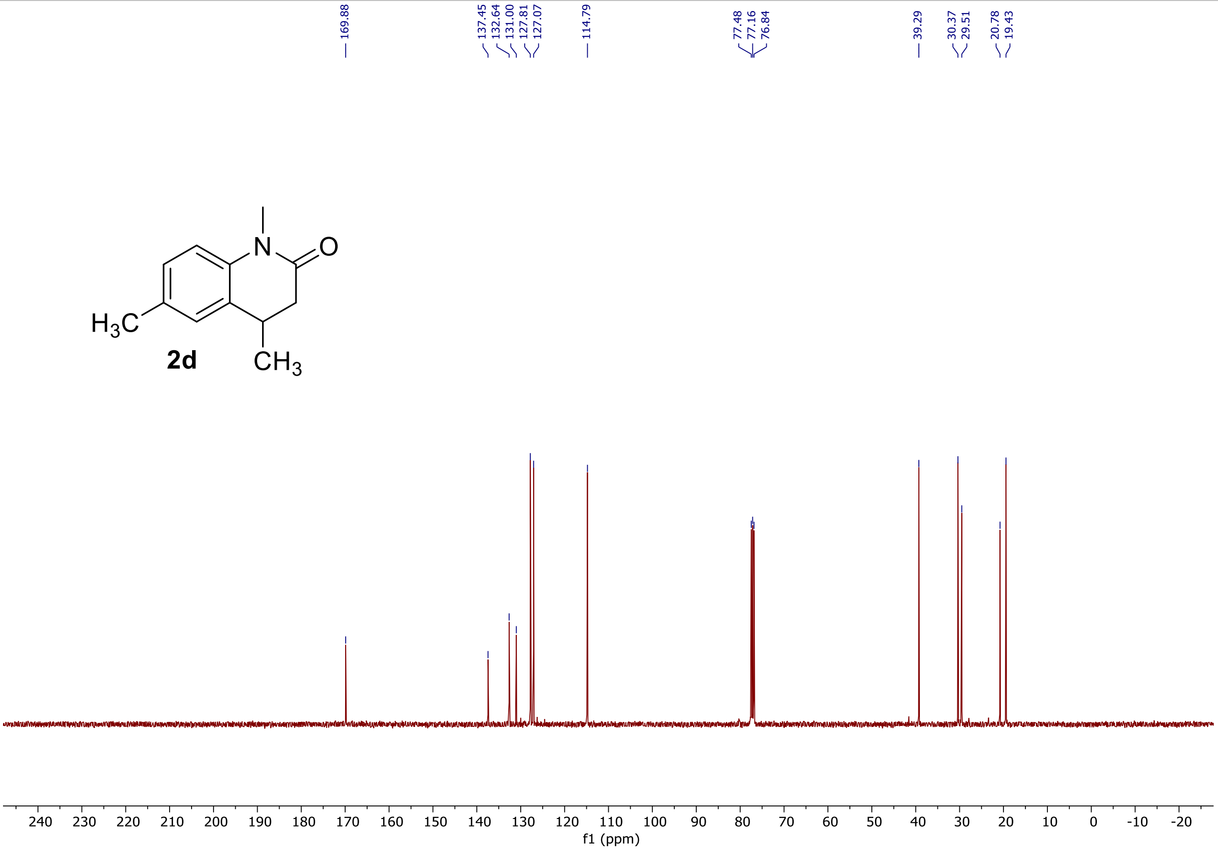
169.88
137.45
132.64
131.00
127.81
127.07
114.79
77.48
77.16
76.84
39.29
30.37
29.51
20.78
19.43
N
O
H_3C
2d
CH_3
f1 (ppm)

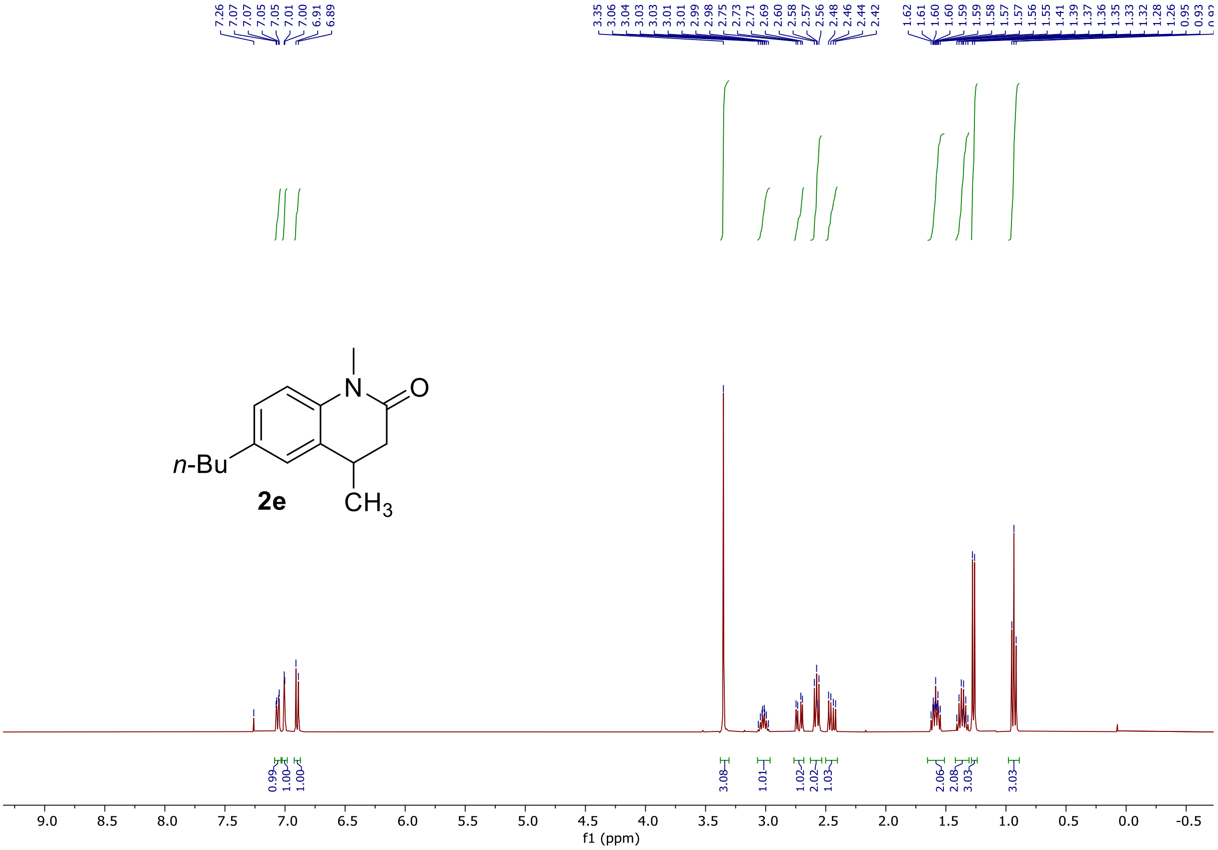
7.26 7.07 7.07 7.05 7.05 7.01 7.00 6.91 6.89
3.35 3.06 3.04 3.03 3.03 3.01 2.99 2.98 2.75 2.73 2.71 2.69 2.60 2.58 2.57 2.56 2.48 2.46 2.44 2.42
1.62 1.61 1.60 1.59 1.59 1.58 1.57 1.56 1.55 1.41 1.39 1.37 1.36 1.35 1.33 1.32 1.28 1.26 0.95 0.93
N
O
n-Bu
2e
CH_3
0.99 1.00 1.00
3.08 1.01 1.02 2.02 1.03
2.06 2.08 3.03 3.03
f1 (ppm)

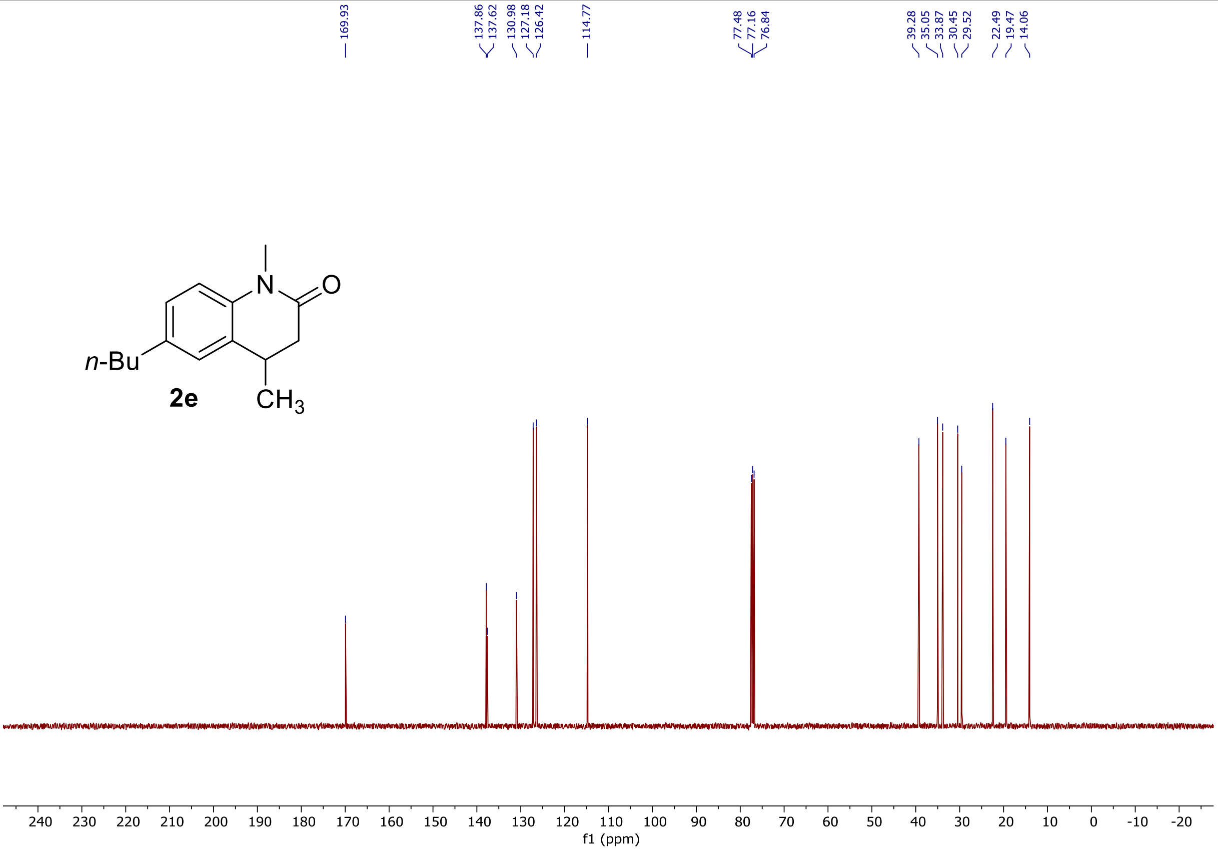
169.93
137.86
137.62
130.98
127.18
126.42
114.77
77.48
77.16
76.84
39.28
35.05
33.87
30.45
29.52
22.49
19.47
14.06
n-Bu
2e
CH3
f1 (ppm)

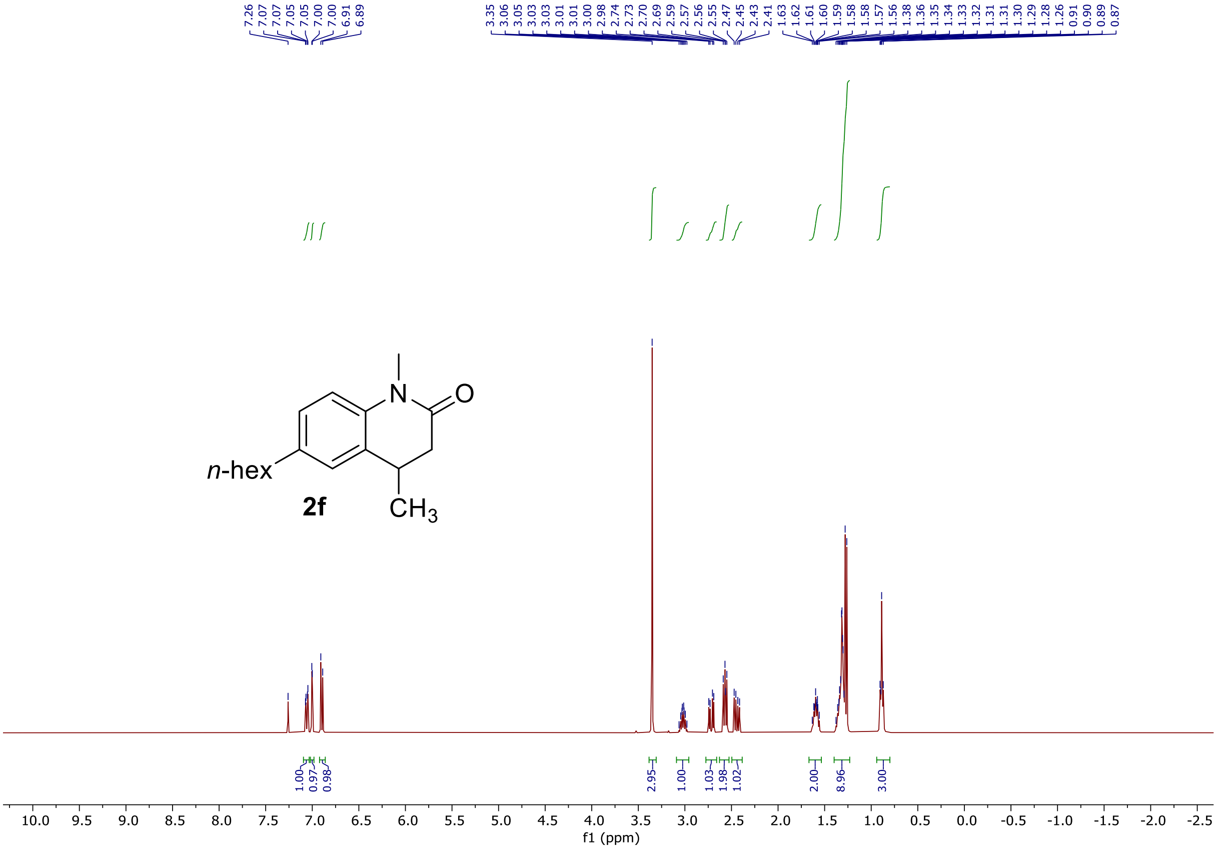
n-hex
2f
CH3
1.00
0.97
0.98
2.95
1.00
1.03
1.98
1.02
2.00
8.96
3.00
f1 (ppm)

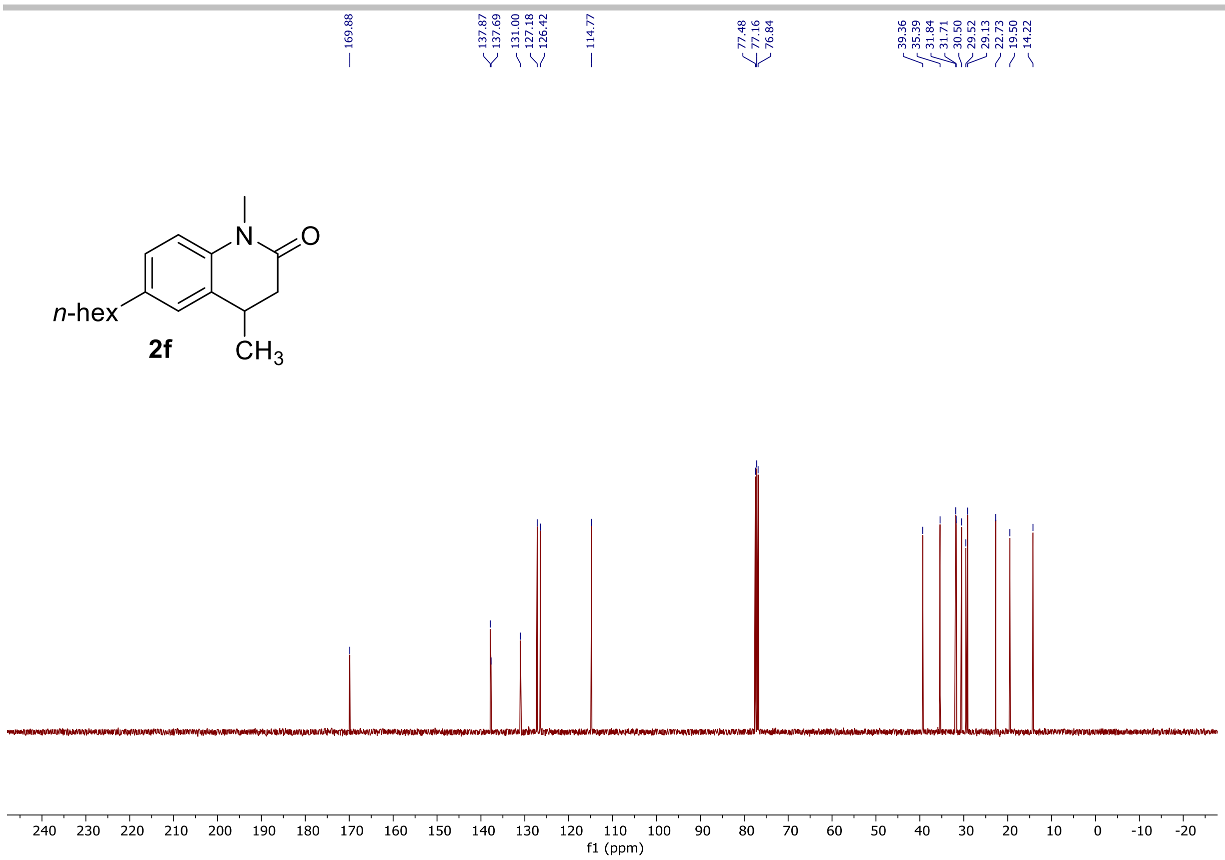
169.88
137.87
137.69
131.00
127.18
126.42
114.77
77.48
77.16
76.84
39.36
35.39
31.84
31.71
30.50
29.52
29.13
22.73
19.50
14.22
N
O
n-hex
2f
CH3
f1 (ppm)

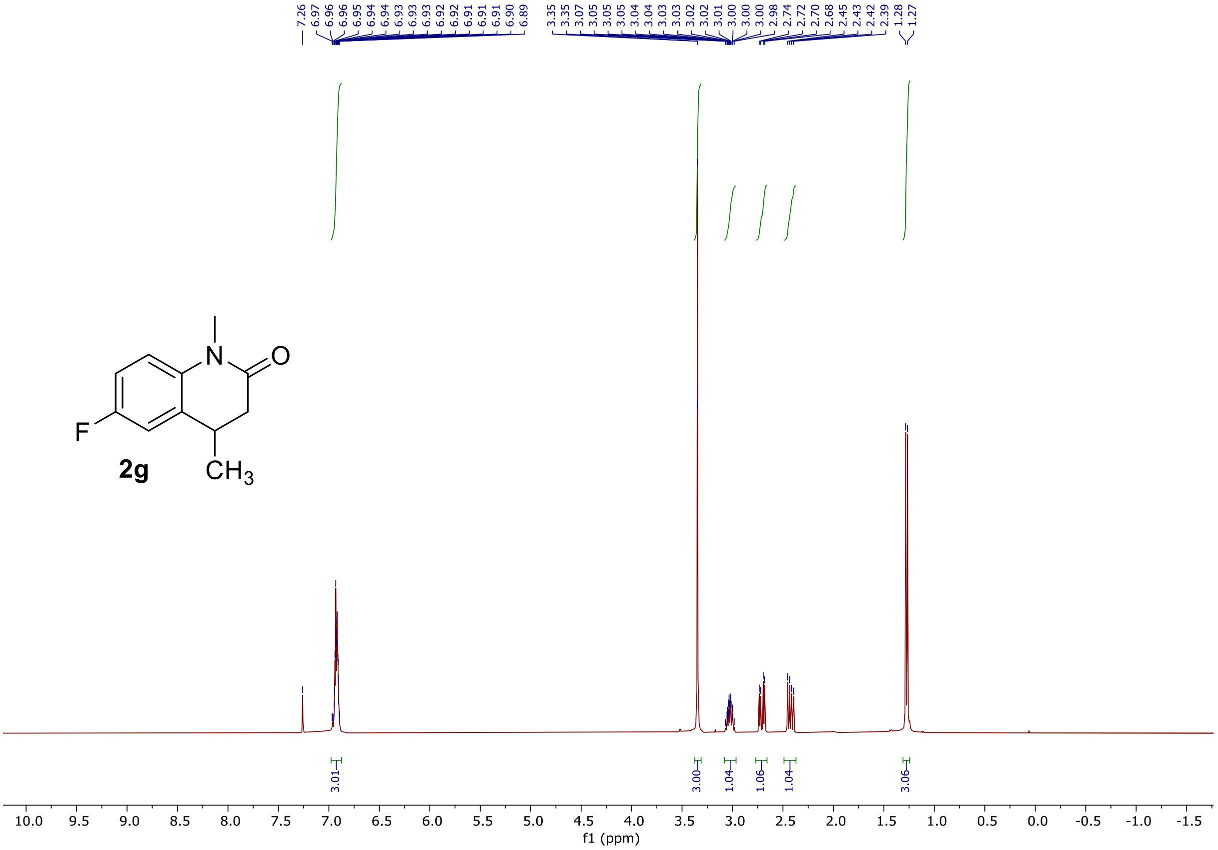
7.26
6.97
6.96
6.96
6.95
6.94
6.94
6.93
6.93
6.93
6.92
6.92
6.91
6.91
6.91
6.90
6.89
3.35
3.35
3.07
3.05
3.05
3.05
3.04
3.04
3.03
3.03
3.02
3.02
3.01
3.00
3.00
3.00
2.98
2.74
2.72
2.70
2.68
2.45
2.43
2.42
2.39
1.28
1.27
N
O
F
2g
CH3
3.01
3.00
1.04
1.06
1.04
3.06
f1 (ppm)

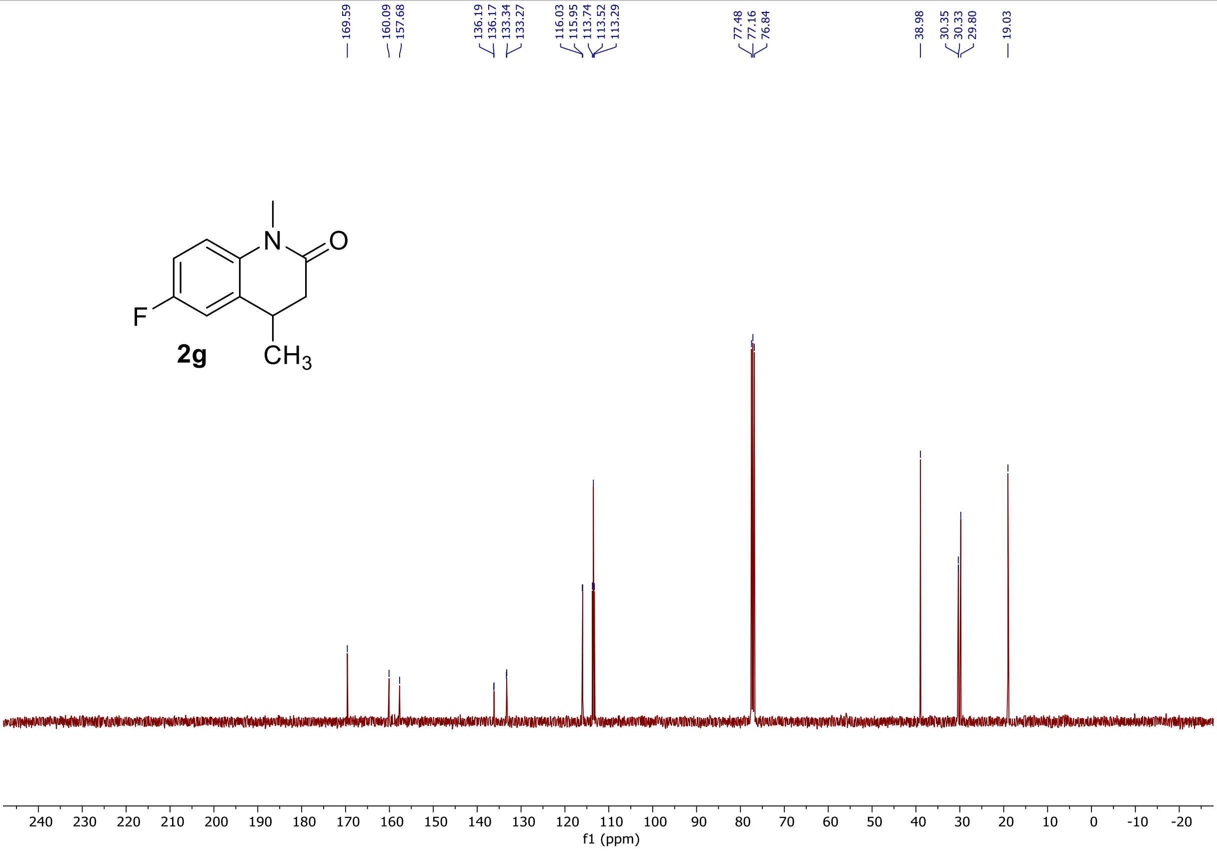
169.59
160.09
157.68
136.19
136.17
133.34
133.27
116.03
115.95
113.74
113.52
113.29
77.48
77.16
76.84
38.98
30.35
30.33
29.80
19.03
F
N
O
CH3
2g
f1 (ppm)

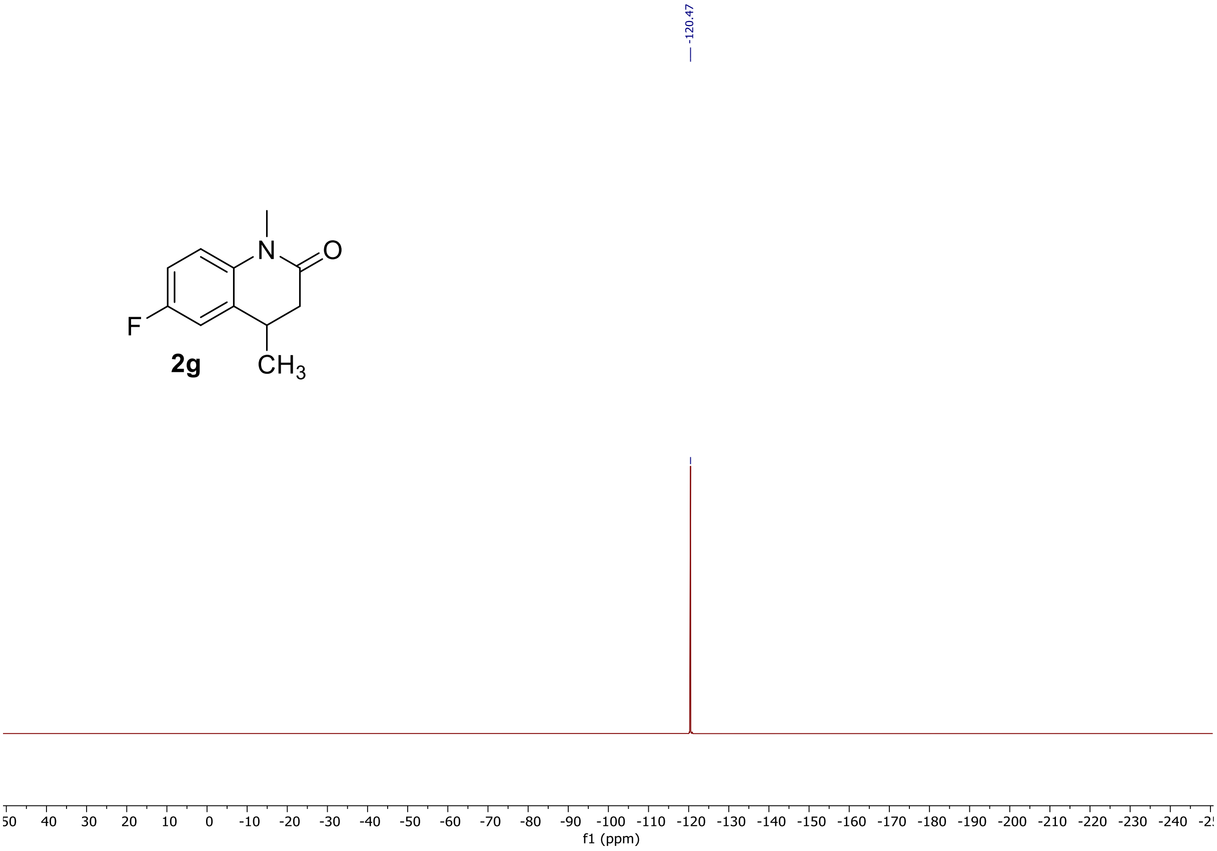
-120.47
F
N
O
CH3
2g
f1 (ppm)

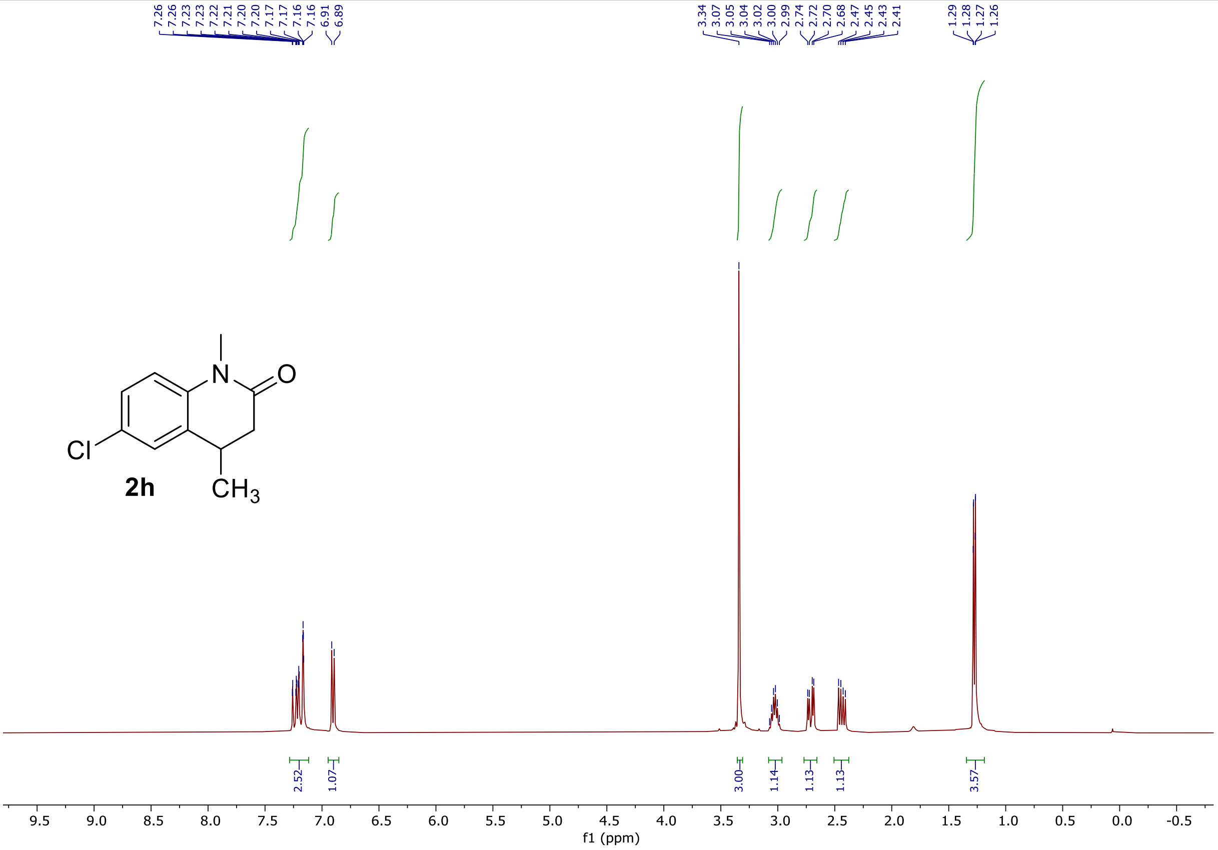
7.26 7.26 7.23 7.23 7.22 7.21 7.20 7.20 7.17 7.17 7.16 7.16 6.91 6.89
3.34 3.07 3.05 3.04 3.02 3.00 2.99 2.74 2.72 2.70 2.68 2.47 2.45 2.43 2.41
1.29 1.28 1.27 1.26
N
O
Cl
2h
CH3
2.52 1.07 3.00 1.14 1.13 1.13 3.57
9.5 9.0 8.5 8.0 7.5 7.0 6.5 6.0 5.5 5.0 4.5 4.0 3.5 3.0 2.5 2.0 1.5 1.0 0.5 0.0 -0.5
f1 (ppm)

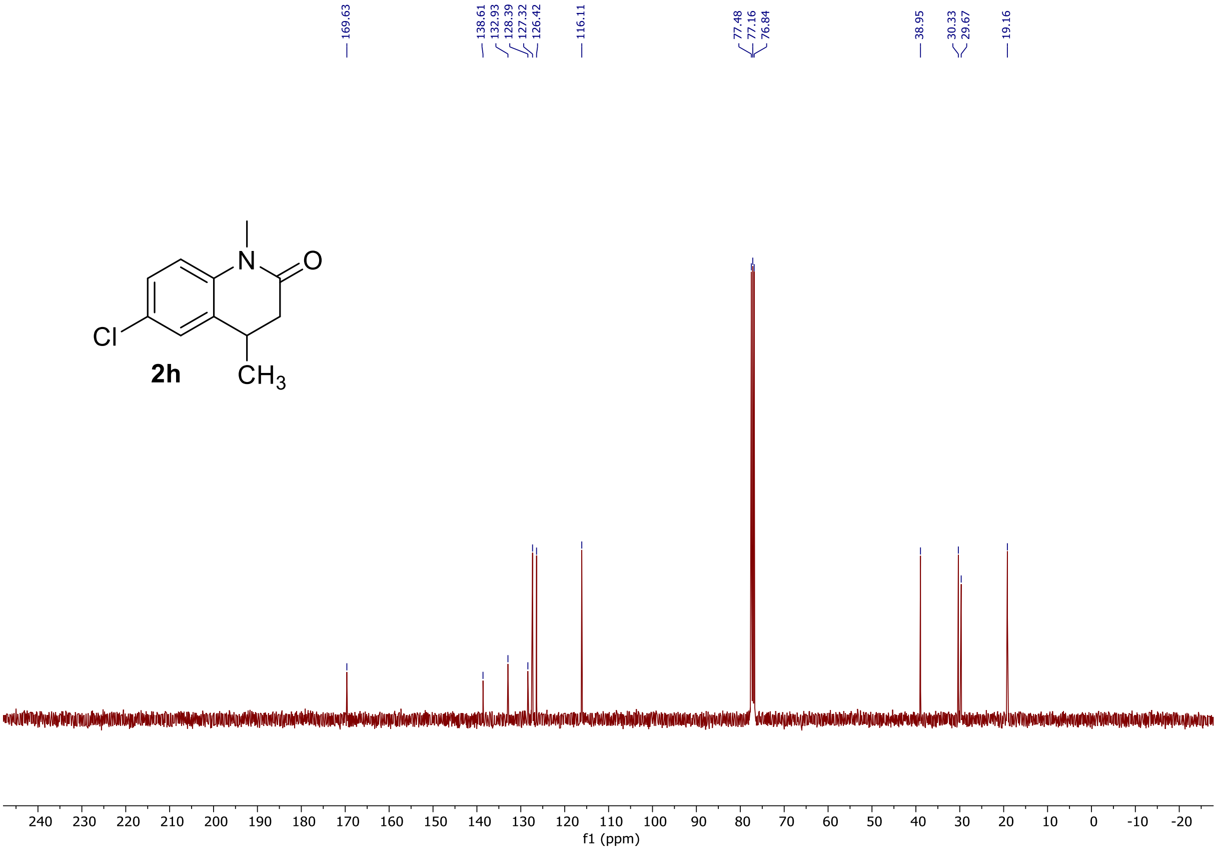
169.63
138.61 132.93 128.39 127.32 126.42
116.11
77.48 77.16 76.84
38.95
30.33 29.67
19.16
N
O
Cl
2h
CH3
240 230 220 210 200 190 180 170 160 150 140 130 120 110 100 90 80 70 60 50 40 30 20 10 0 -10 -20
f1 (ppm)

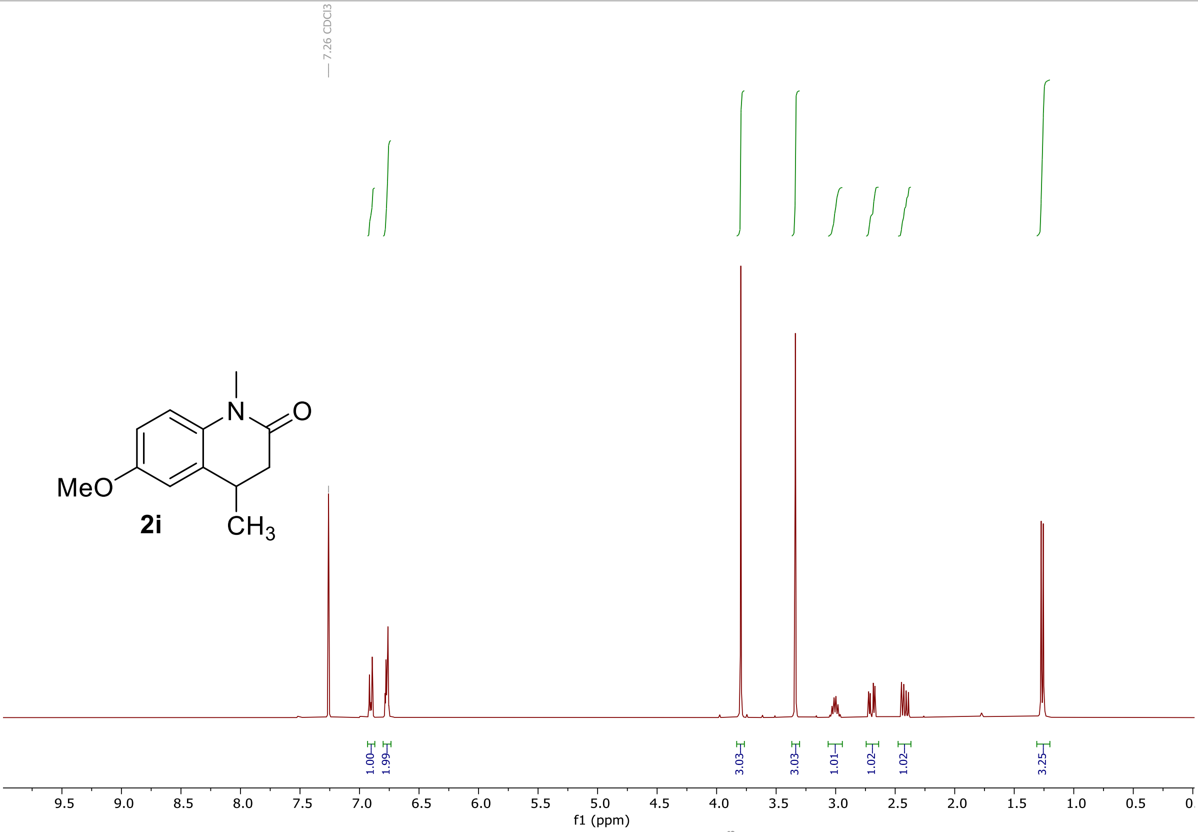
7.26 CDCl3
MeO
N
O
2i
CH3
1.00
1.99
3.03
3.03
1.01
1.02
1.02
3.25
9.5
9.0
8.5
8.0
7.5
7.0
6.5
6.0
5.5
5.0
4.5
4.0
3.5
3.0
2.5
2.0
1.5
1.0
0.5
f1 (ppm)

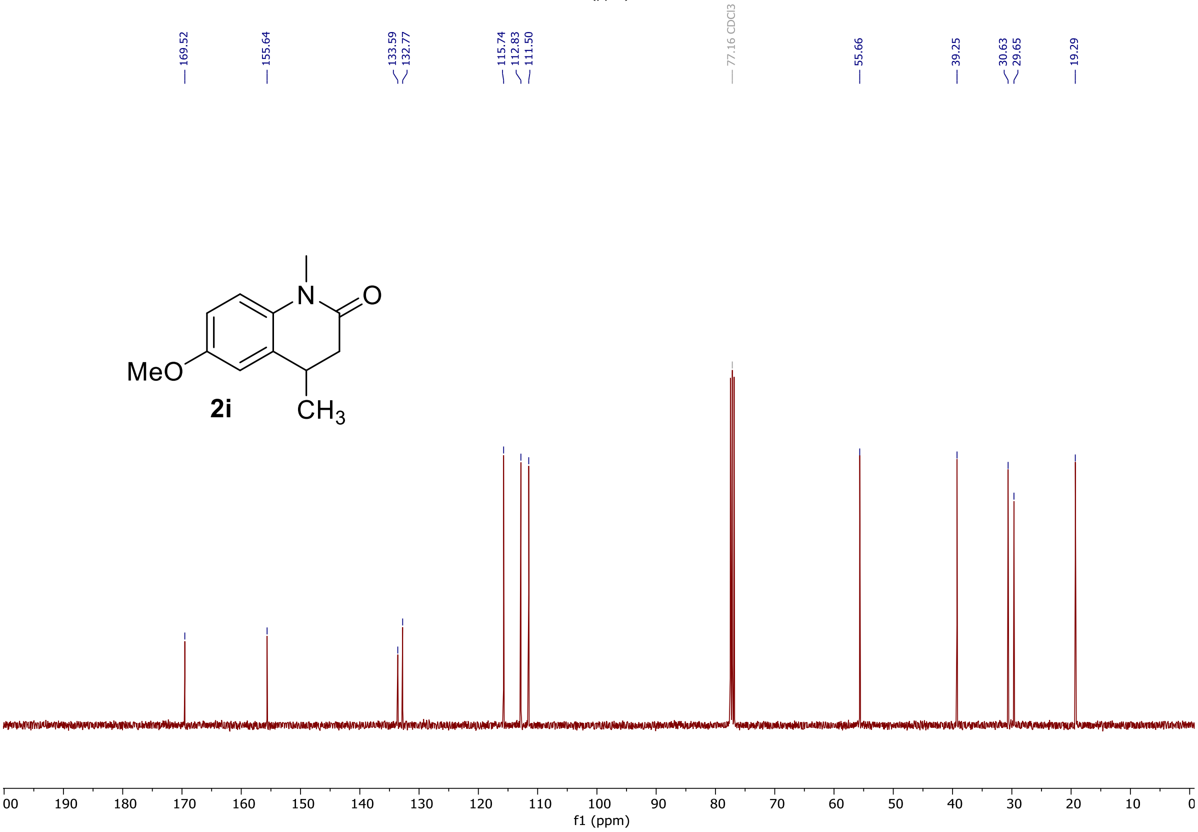
169.52
155.64
133.59
132.77
115.74
112.83
111.50
77.16 CDCl3
55.66
39.25
30.63
29.65
19.29
MeO
N
O
2i
CH3
190
180
170
160
150
140
130
120
110
100
90
80
70
60
50
40
30
20
10
0
f1 (ppm)

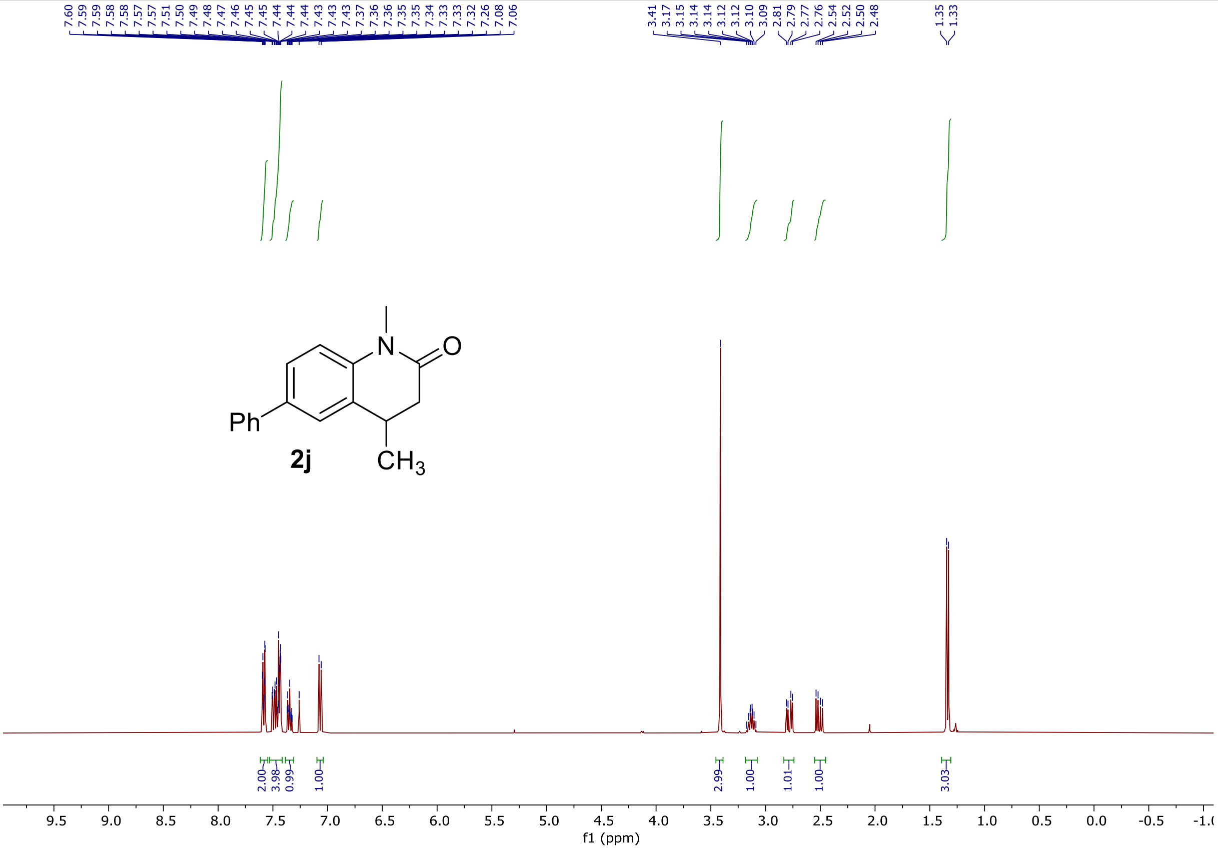
7.60
7.59
7.59
7.58
7.58
7.57
7.57
7.51
7.50
7.49
7.48
7.47
7.46
7.45
7.45
7.44
7.44
7.44
7.43
7.43
7.43
7.37
7.36
7.36
7.35
7.35
7.34
7.33
7.33
7.32
7.26
7.08
7.06
3.41
3.17
3.15
3.14
3.14
3.12
3.12
3.10
3.09
2.81
2.79
2.77
2.76
2.54
2.52
2.50
2.48
1.35
1.33
Ph
N
O
2j
CH3
2.00
3.98
0.99
1.00
2.99
1.00
1.01
1.00
3.03
f1 (ppm)

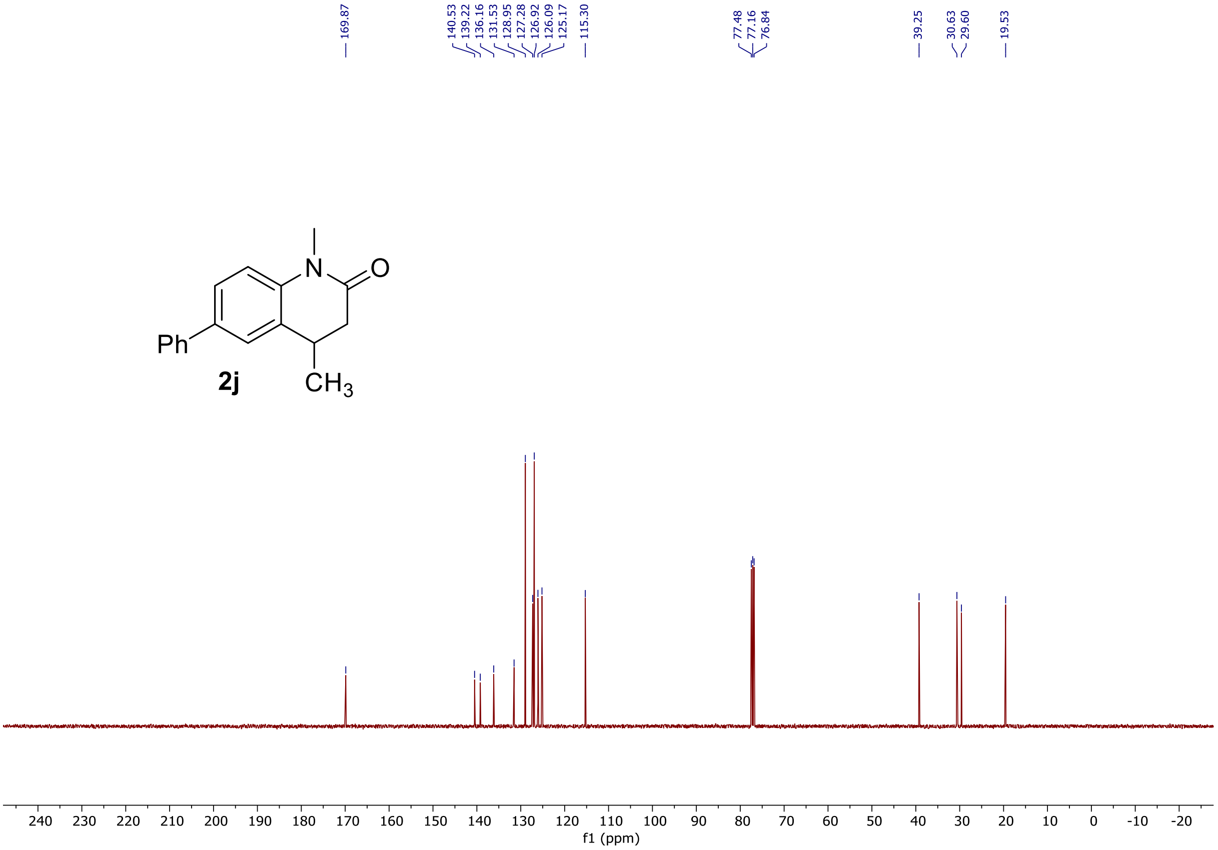
169.87
140.53
139.22
136.16
131.53
128.95
127.28
126.92
126.09
125.17
115.30
77.48
77.16
76.84
39.25
30.63
29.60
19.53
Ph
N
O
2j
CH3
f1 (ppm)

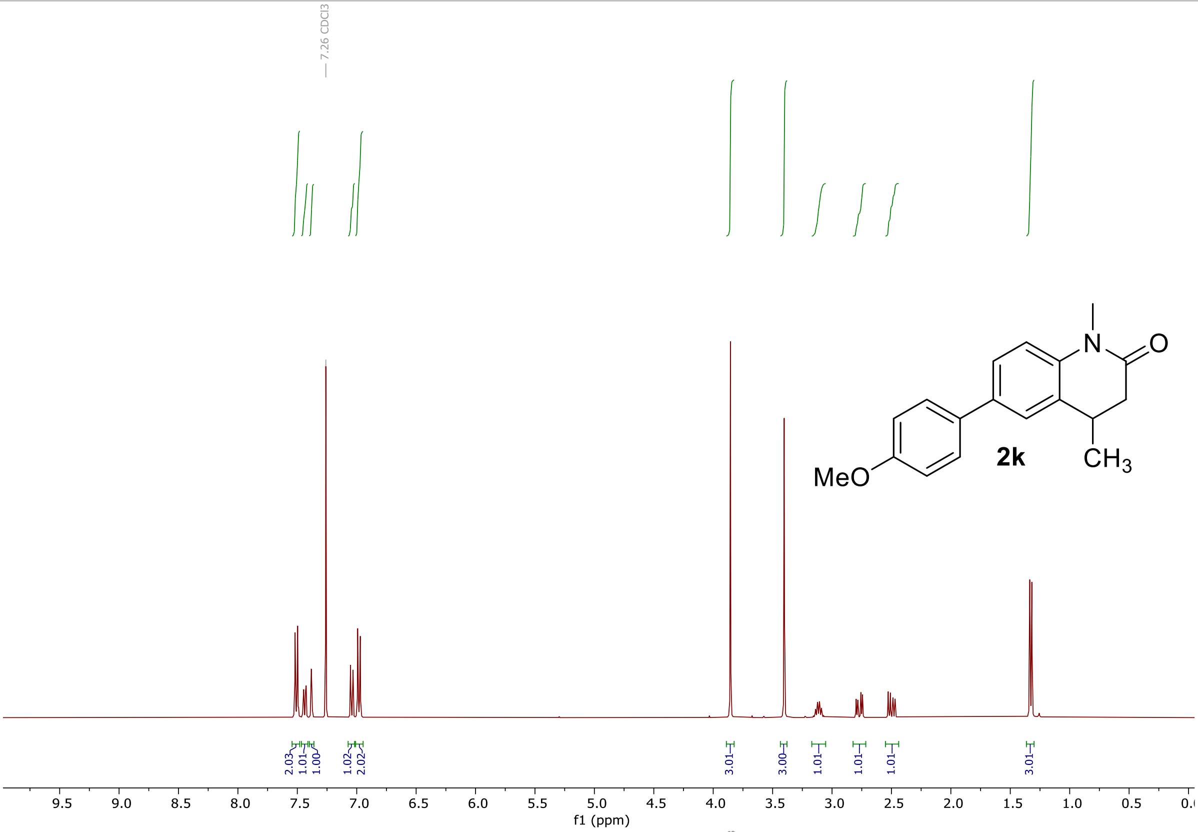
7.26 CDCl3
2k
MeO
CH3
N
O
2.03
1.01
1.00
1.02
2.02
3.01
3.00
1.01
1.01
1.01
3.01
f1 (ppm)

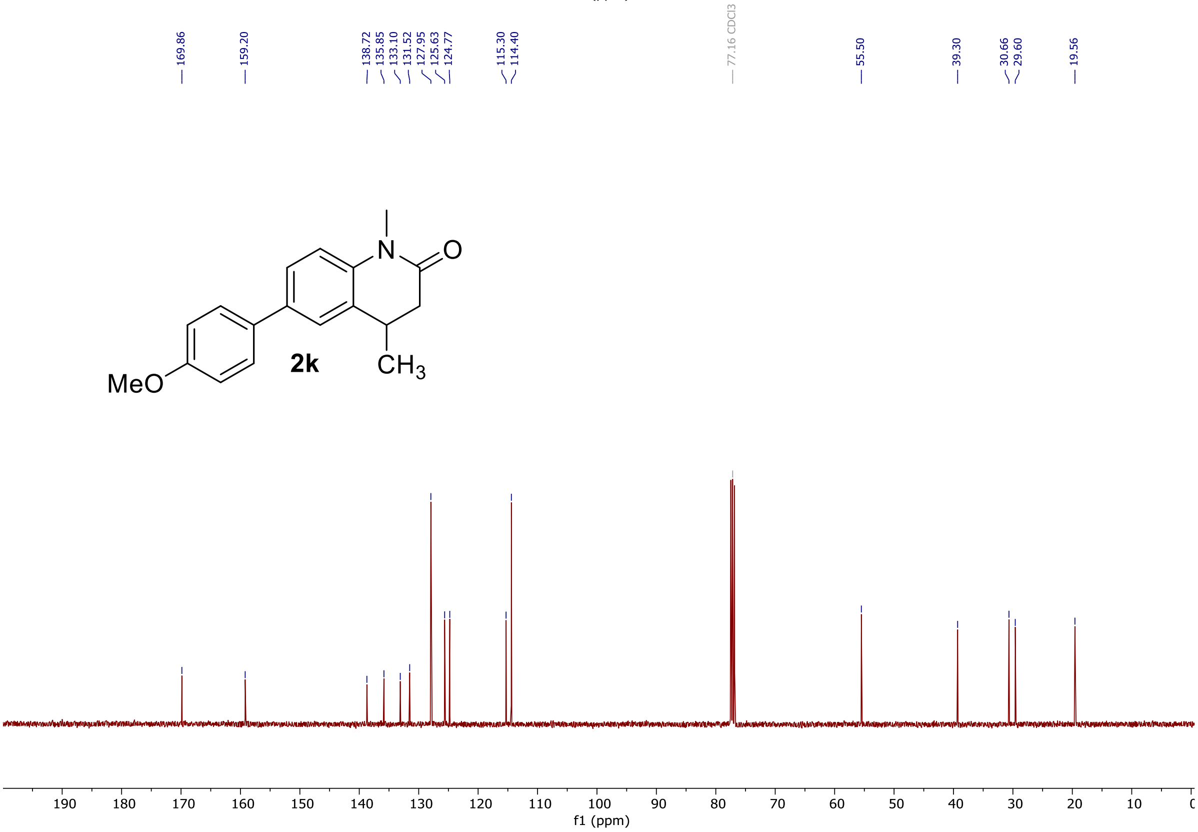
169.86
159.20
138.72
135.85
133.10
131.52
127.95
125.63
124.77
115.30
114.40
77.16 CDCl3
55.50
39.30
30.66
29.60
19.56
2k
MeO
CH3
N
O
f1 (ppm)

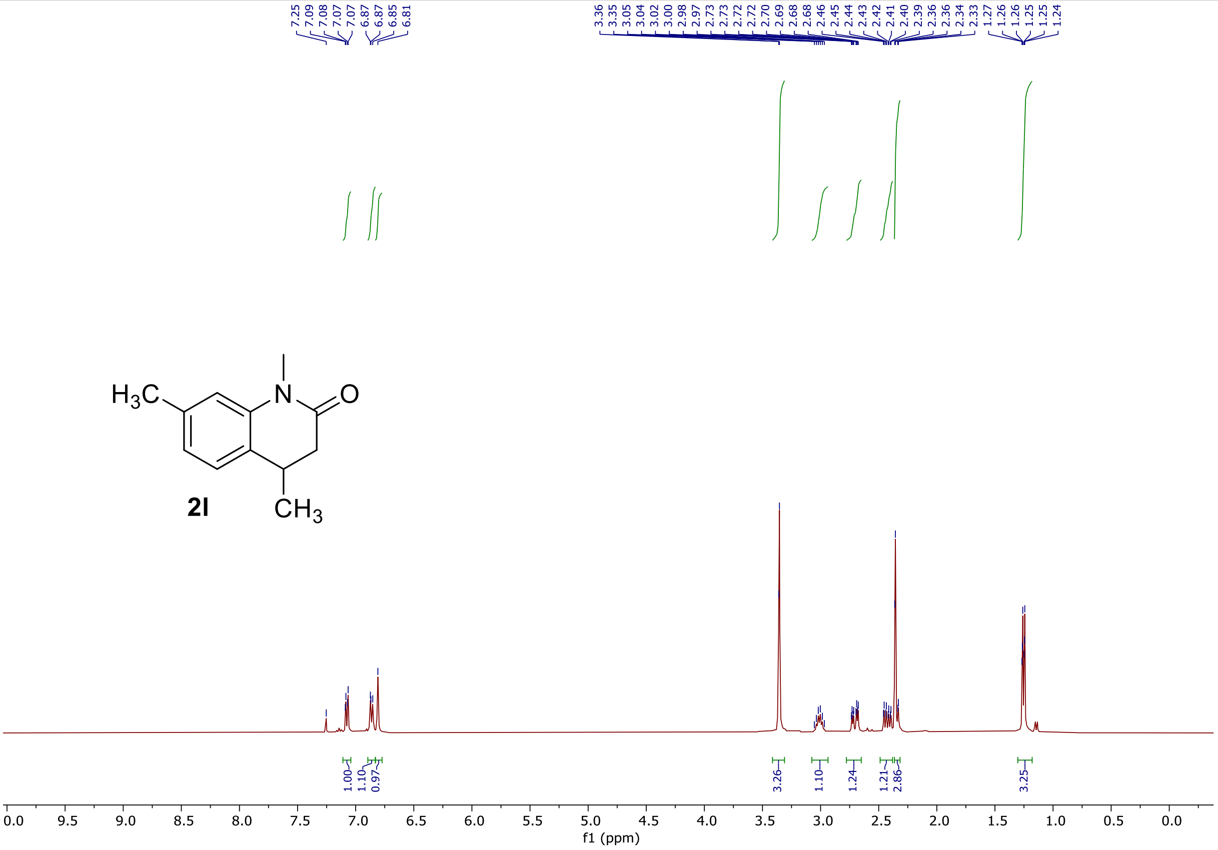
7.25
7.09
7.08
7.07
7.07
6.87
6.87
6.85
6.81
3.36
3.35
3.05
3.04
3.02
3.00
2.98
2.97
2.73
2.73
2.72
2.72
2.70
2.69
2.68
2.68
2.46
2.45
2.44
2.43
2.42
2.41
2.40
2.39
2.36
2.36
2.34
2.33
1.27
1.26
1.26
1.25
1.25
1.24
H3C
N
O
2l
CH3
1.00
1.10
0.97
3.26
1.10
1.24
1.21
2.86
3.25
f1 (ppm)

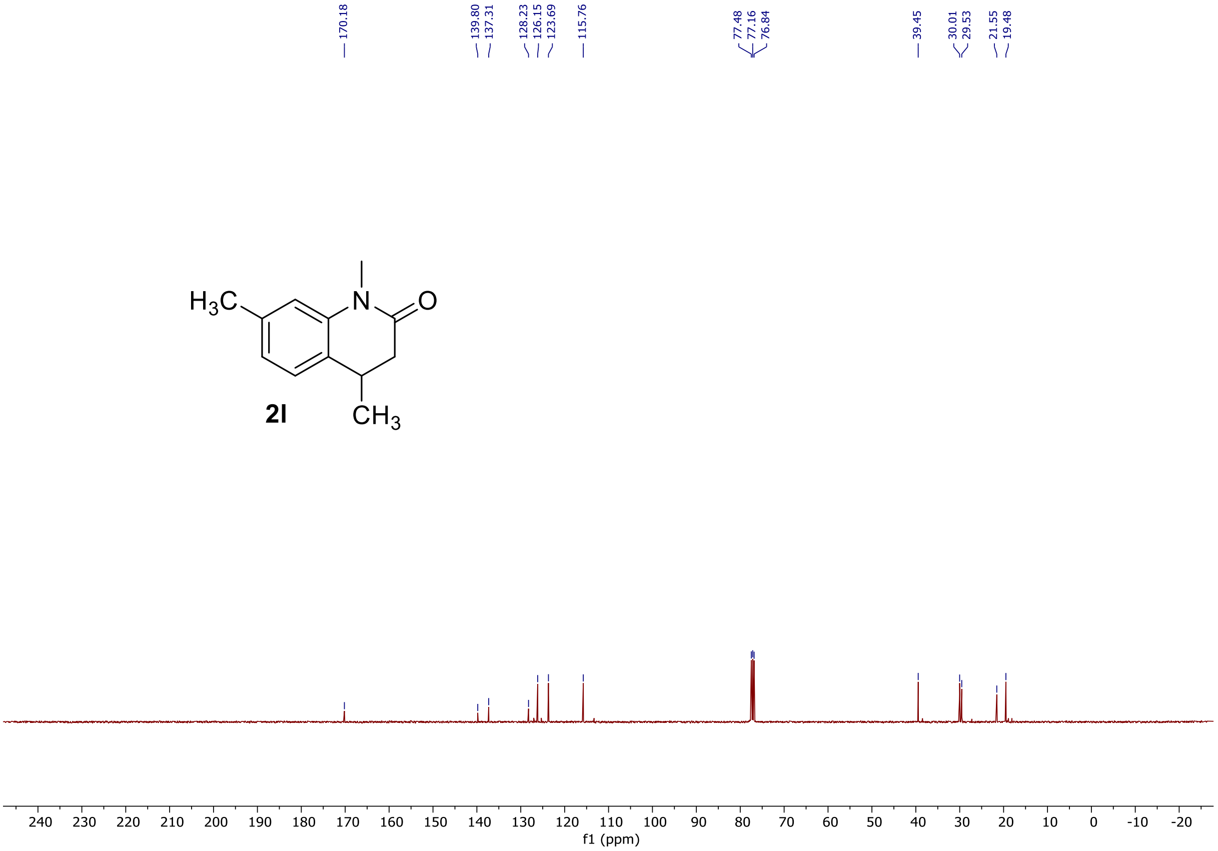
170.18
139.80
137.31
128.23
126.15
123.69
115.76
77.48
77.16
76.84
39.45
30.01
29.53
21.55
19.48
H3C
N
O
2l
CH3
f1 (ppm)

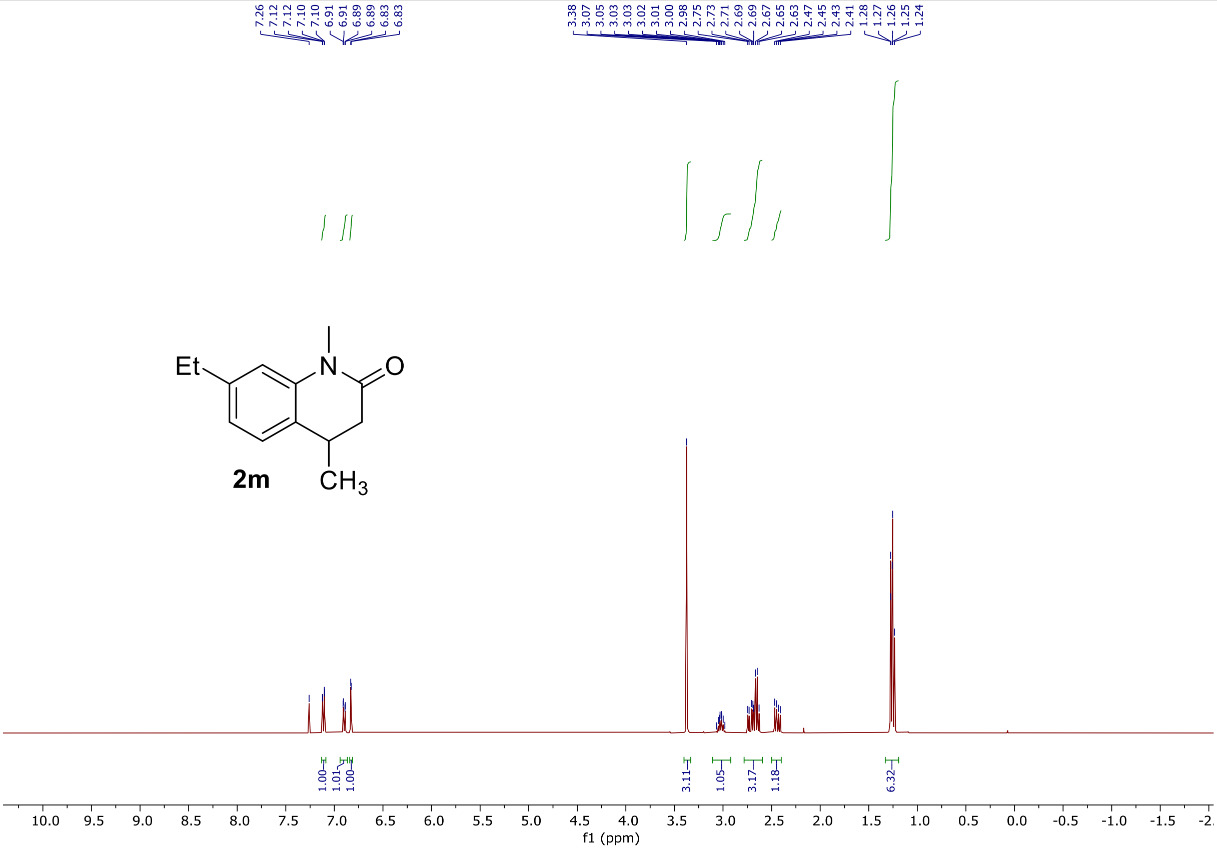
7.26
7.12
7.12
7.10
7.10
6.91
6.91
6.89
6.89
6.83
6.83
3.38
3.07
3.05
3.03
3.03
3.02
3.01
3.00
2.98
2.75
2.73
2.71
2.69
2.69
2.67
2.65
2.63
2.47
2.45
2.43
2.41
1.28
1.27
1.26
1.25
1.24
Et
N
O
2m
CH3
1.00
1.01
1.00
3.11
1.05
3.17
1.18
6.32
f1 (ppm)

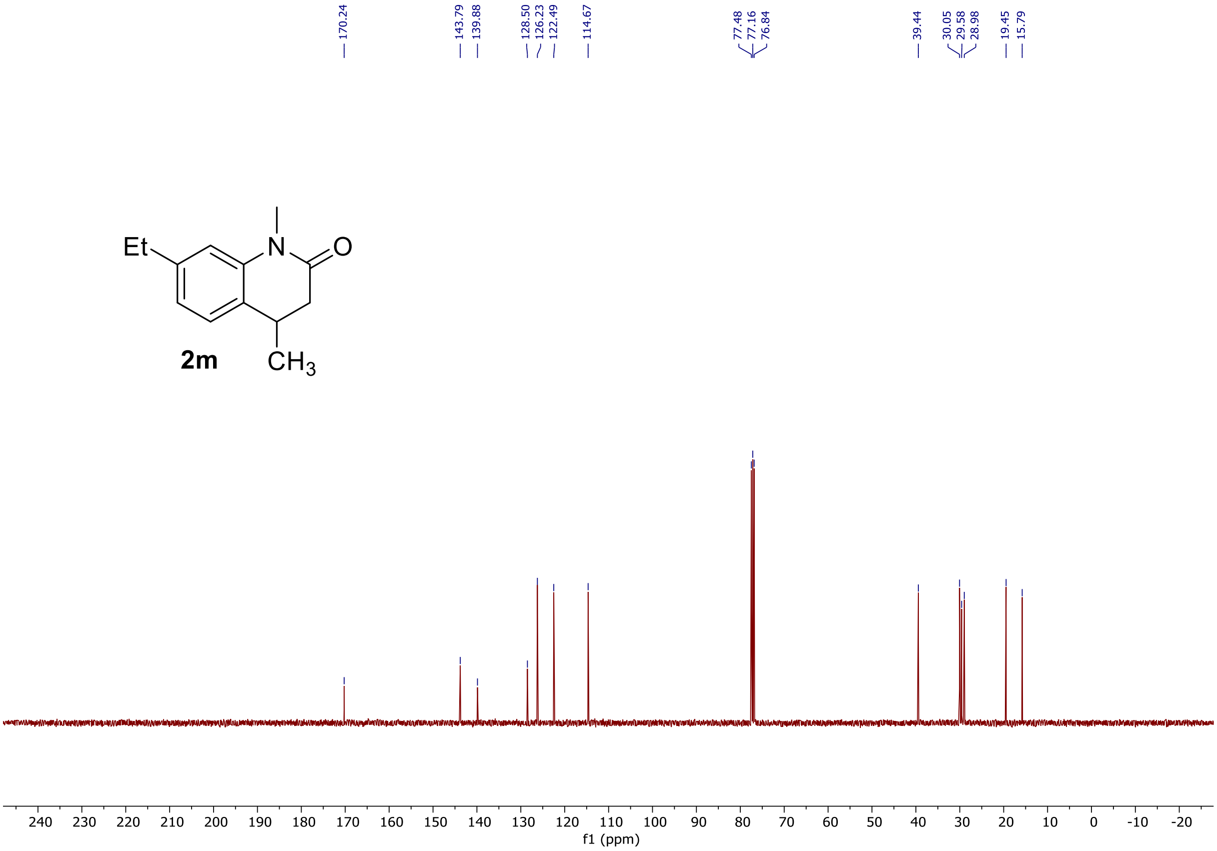
170.24
143.79
139.88
128.50
126.23
122.49
114.67
77.48
77.16
76.84
39.44
30.05
29.58
28.98
19.45
15.79
Et
N
O
2m
CH3
f1 (ppm)

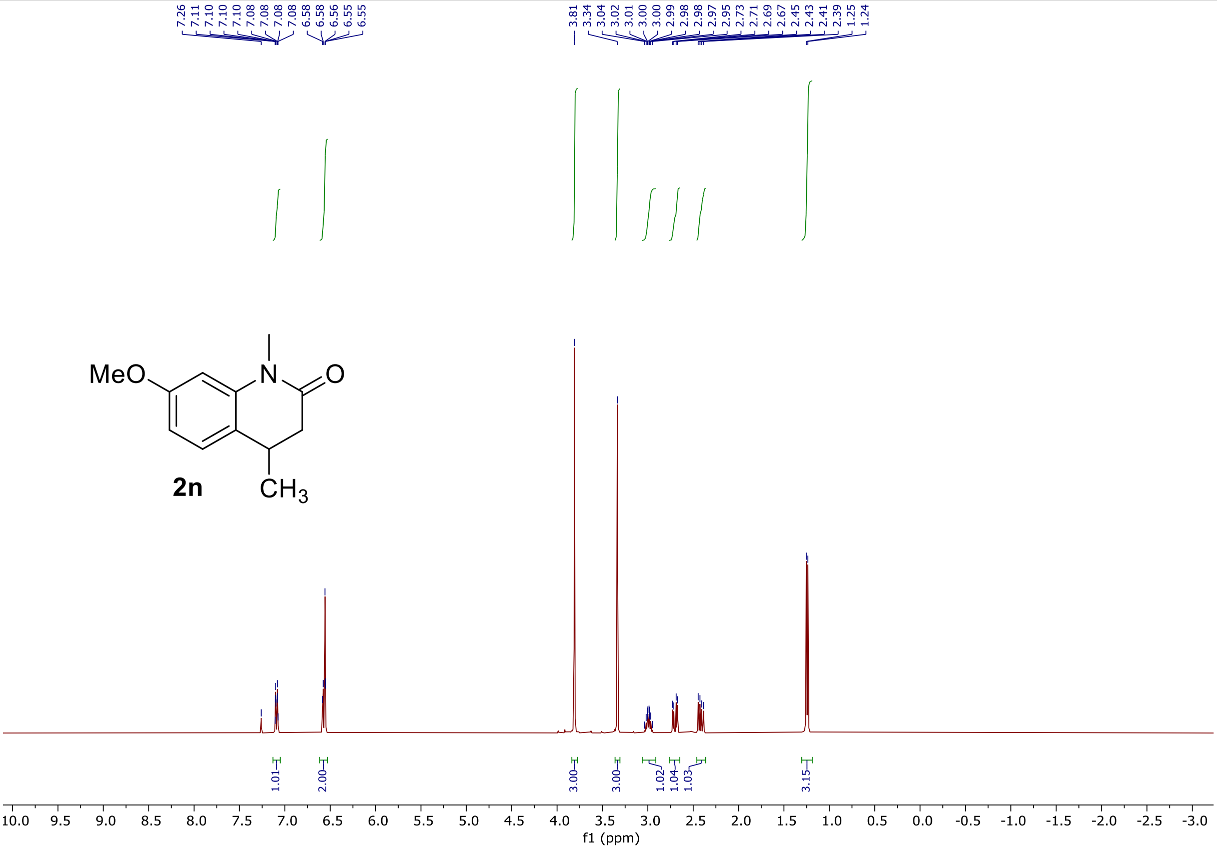
7.26
7.11
7.10
7.10
7.10
7.08
7.08
7.08
6.58
6.58
6.56
6.55
6.55
3.81
3.34
3.04
3.02
3.01
3.00
3.00
2.99
2.98
2.98
2.97
2.95
2.73
2.71
2.69
2.67
2.45
2.43
2.41
2.39
1.25
1.24
MeO
N
O
2n
CH3
1.01
2.00
3.00
3.00
1.02
1.04
1.03
3.15
f1 (ppm)

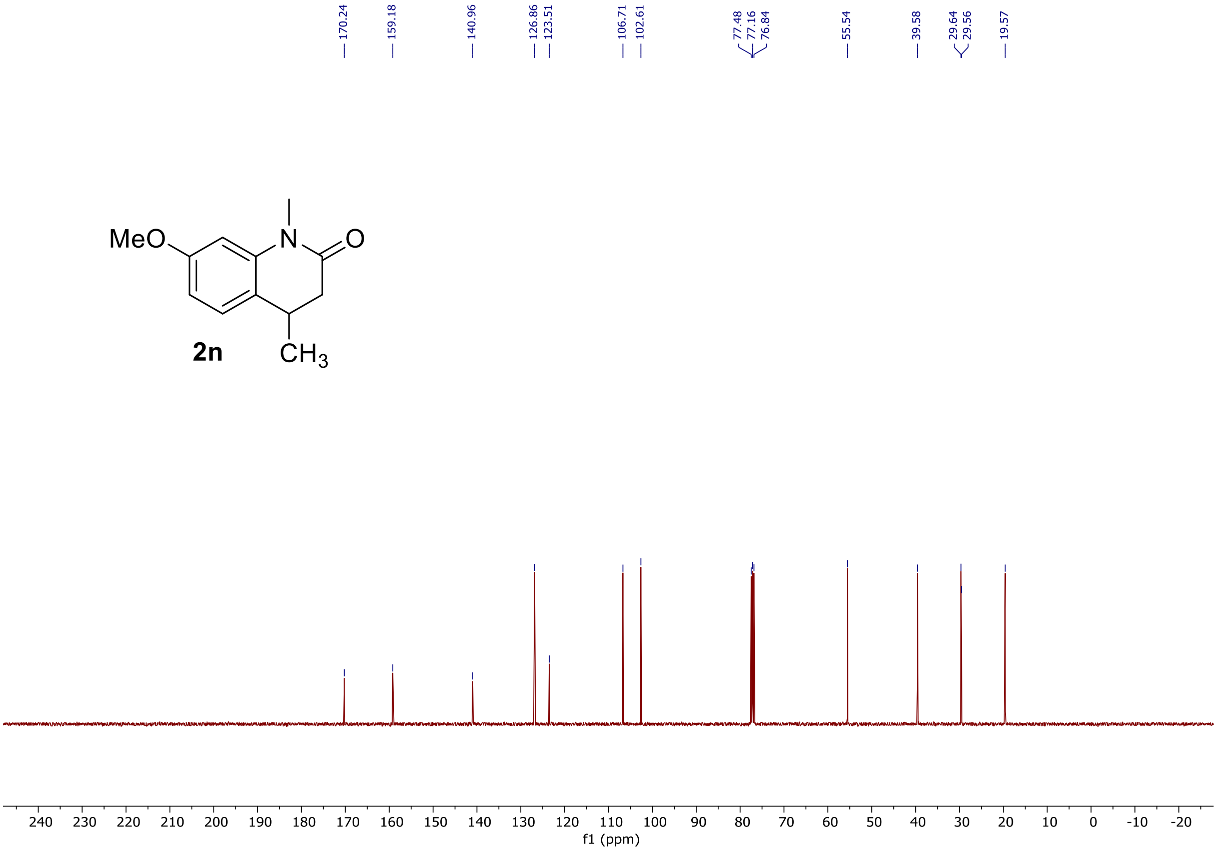
170.24
159.18
140.96
126.86
123.51
106.71
102.61
77.48
77.16
76.84
55.54
39.58
29.64
29.56
19.57
MeO
N
O
2n
CH3
f1 (ppm)

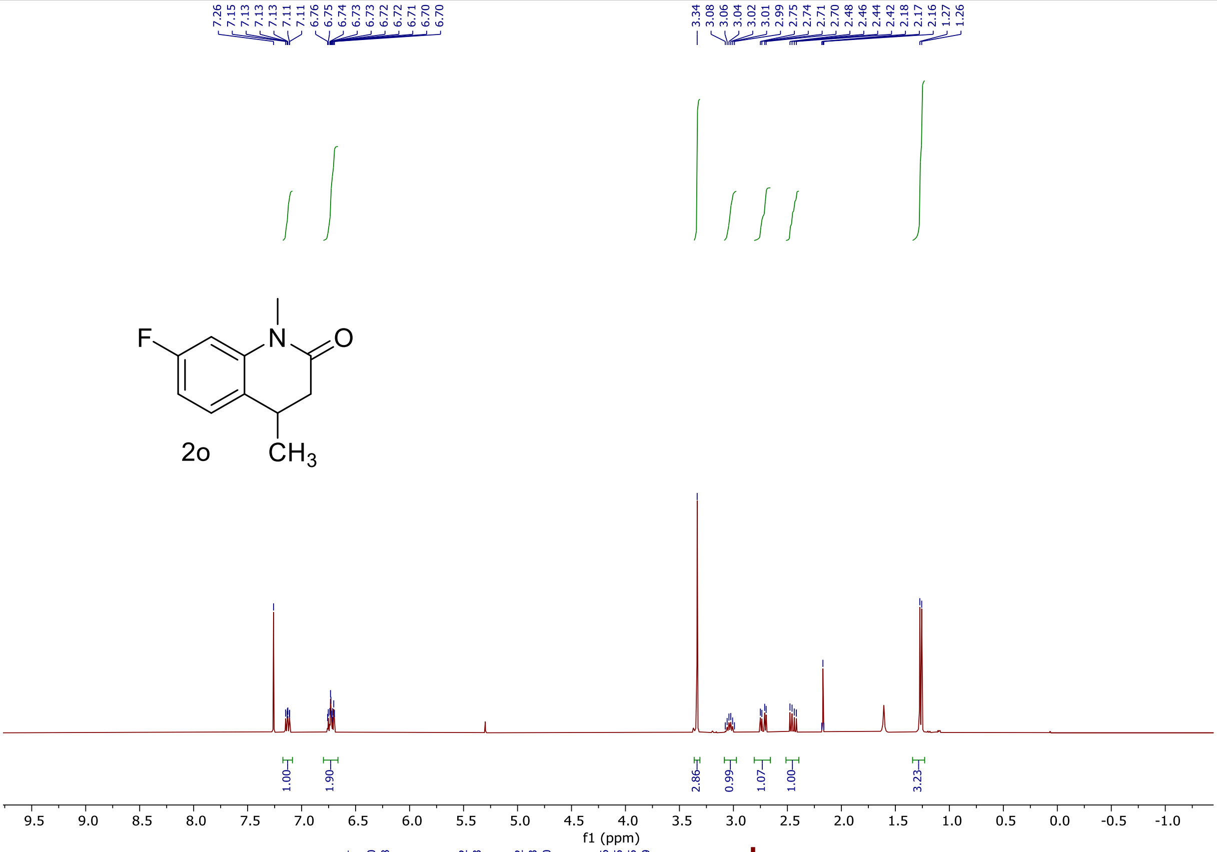
7.26
7.15
7.13
7.13
7.13
7.11
7.11
6.76
6.75
6.74
6.73
6.73
6.72
6.72
6.71
6.70
6.70
3.34
3.08
3.06
3.04
3.02
3.01
2.99
2.75
2.74
2.71
2.70
2.48
2.46
2.44
2.42
2.18
2.17
2.16
1.27
1.26
F
N
O
2o
CH3
1.00
1.90
2.86
0.99
1.07
1.00
3.23
f1 (ppm)

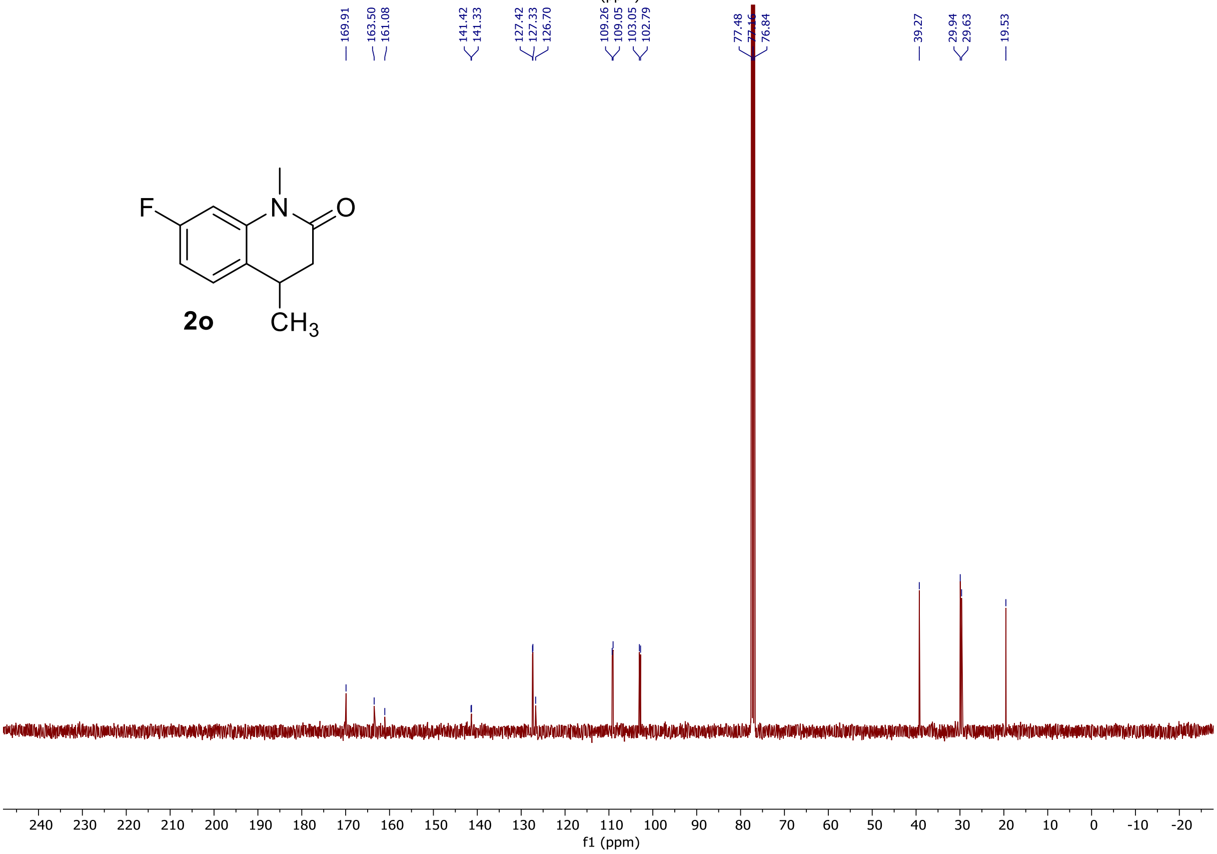
169.91
163.50
161.08
141.42
141.33
127.42
127.33
126.70
109.26
109.05
103.05
102.79
77.48
76.84
39.27
29.94
29.63
19.53
F
N
O
2o
CH3
f1 (ppm)

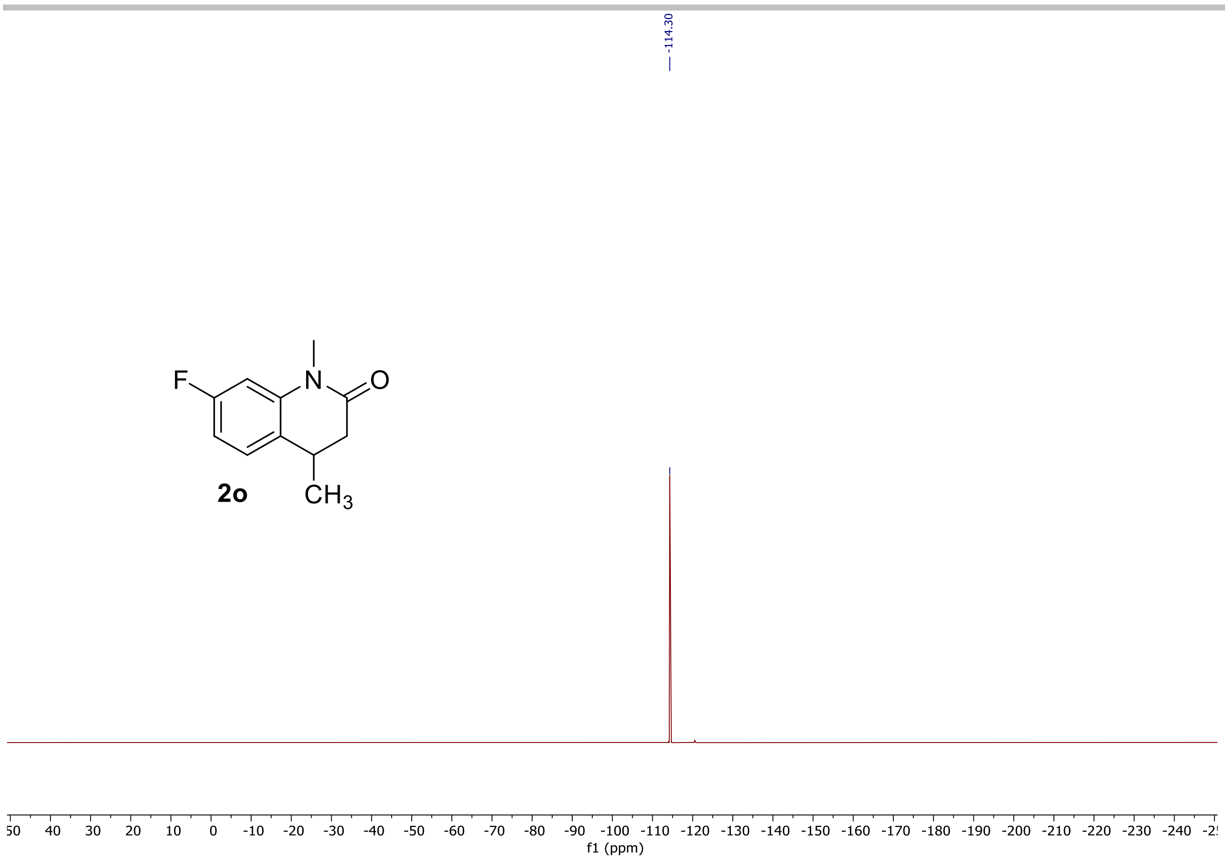
-114.30
F
N
O
2o
CH3
50 40 30 20 10 0 -10 -20 -30 -40 -50 -60 -70 -80 -90 -100 -110 -120 -130 -140 -150 -160 -170 -180 -190 -200 -210 -220 -230 -240
f1 (ppm)

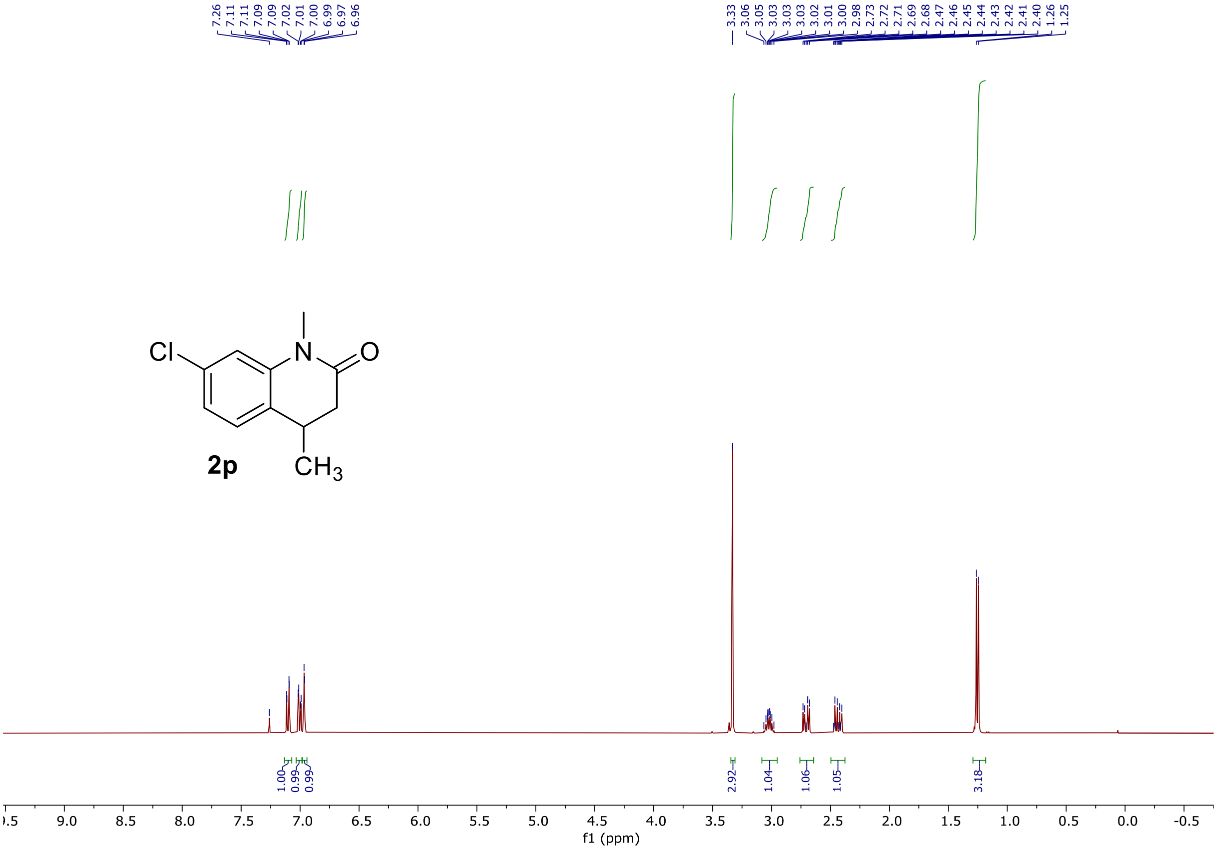
7.26 7.11 7.11 7.09 7.09 7.02 7.01 7.00 6.99 6.97 6.96
3.33 3.06 3.05 3.03 3.03 3.02 3.01 3.00 2.98 2.73 2.72 2.71 2.69 2.68 2.47 2.46 2.45 2.44 2.43 2.42 2.41 2.40 1.26 1.25
Cl
N
O
2p
CH3
1.00 0.99 0.99
2.92 1.04 1.06 1.05 3.18
9.0 8.5 8.0 7.5 7.0 6.5 6.0 5.5 5.0 4.5 4.0 3.5 3.0 2.5 2.0 1.5 1.0 0.5 0.0 -0.5
f1 (ppm)

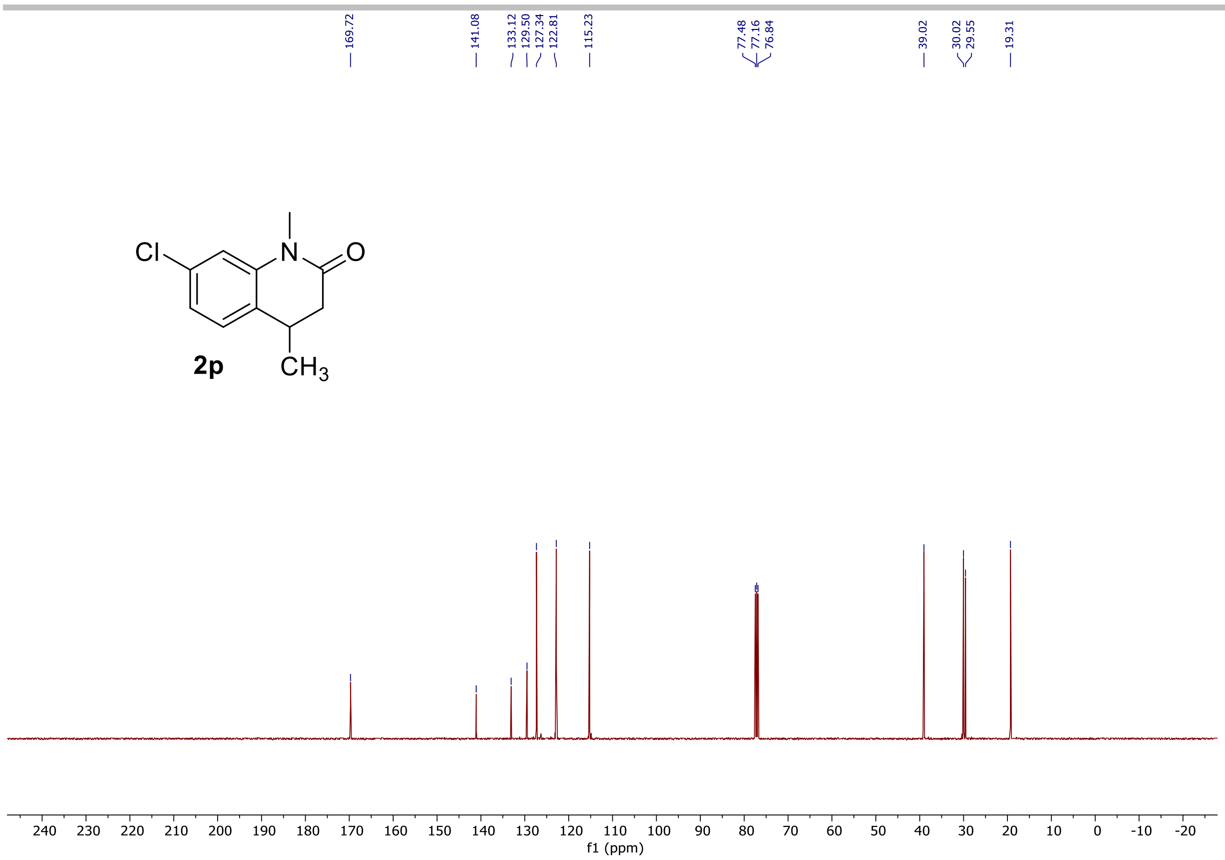
169.72
141.08
133.12
129.50
127.34
122.81
115.23
77.48
77.16
76.84
39.02
30.02
29.55
19.31
Cl
N
O
2p
CH3
f1 (ppm)

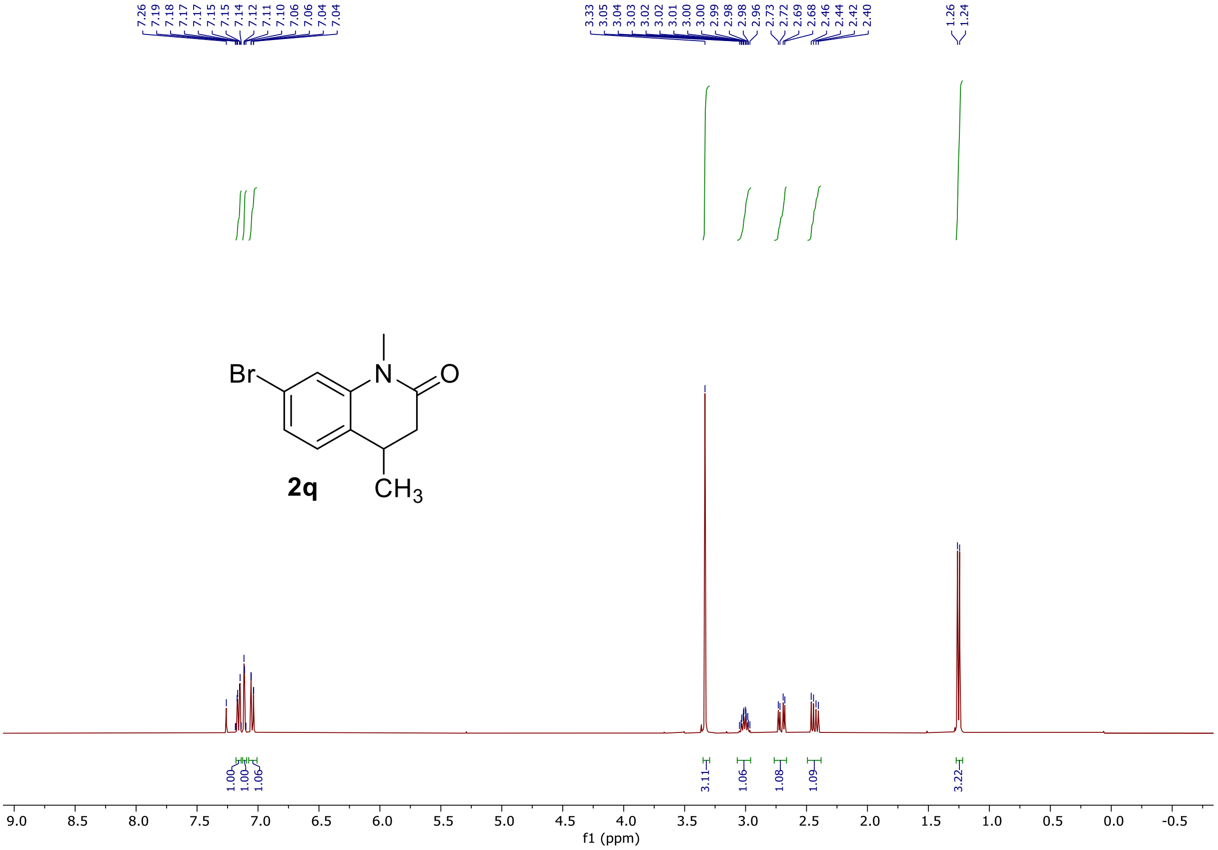
7.26
7.19
7.18
7.17
7.17
7.15
7.15
7.14
7.12
7.11
7.10
7.06
7.06
7.04
7.04
3.33
3.05
3.04
3.03
3.02
3.02
3.01
3.00
3.00
2.99
2.98
2.98
2.96
2.73
2.72
2.69
2.68
2.46
2.44
2.42
2.40
1.26
1.24
Br
N
O
2q
CH3
1.00
1.00
1.06
3.11
1.06
1.08
1.09
3.22
f1 (ppm)

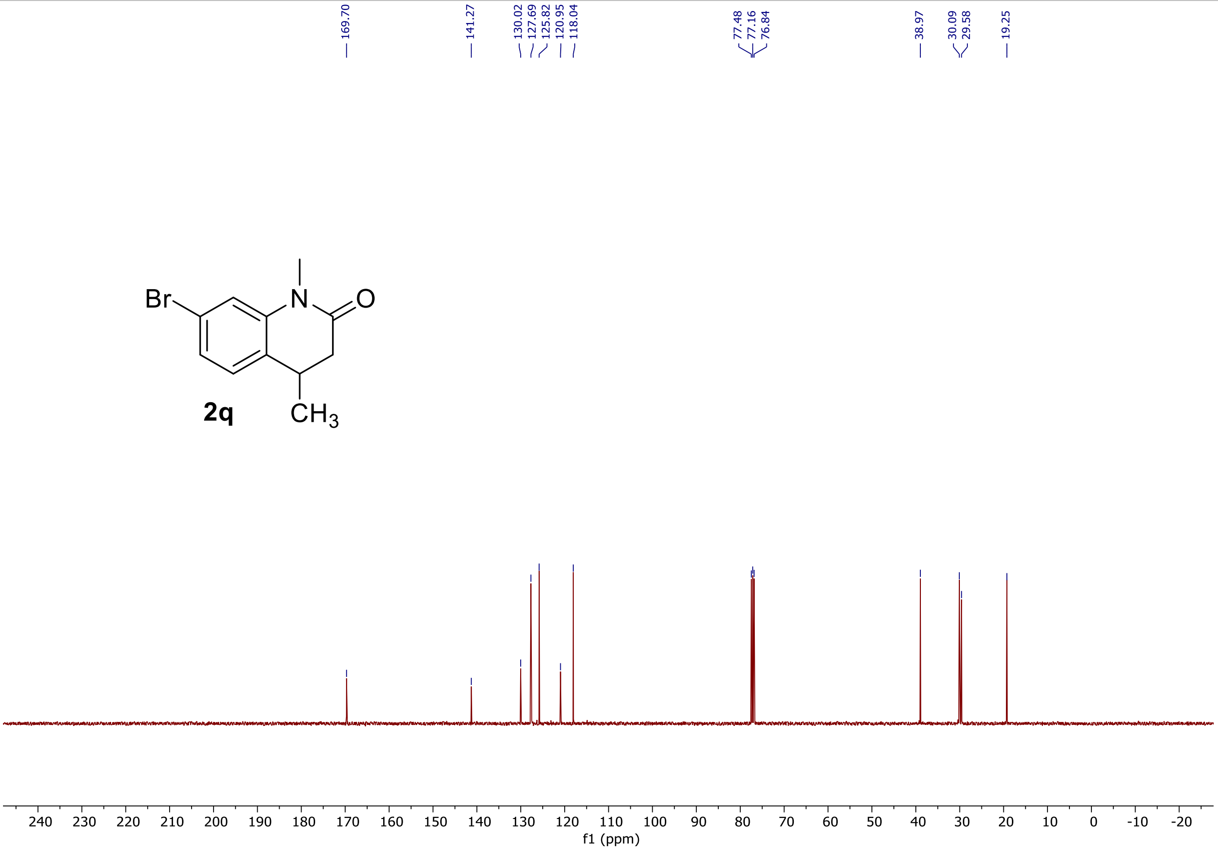
169.70
141.27
130.02
127.69
125.82
120.95
118.04
77.48
77.16
76.84
38.97
30.09
29.58
19.25
Br
N
O
2q
CH3
240 230 220 210 200 190 180 170 160 150 140 130 120 110 100 90 80 70 60 50 40 30 20 10 0 -10 -20
f1 (ppm)

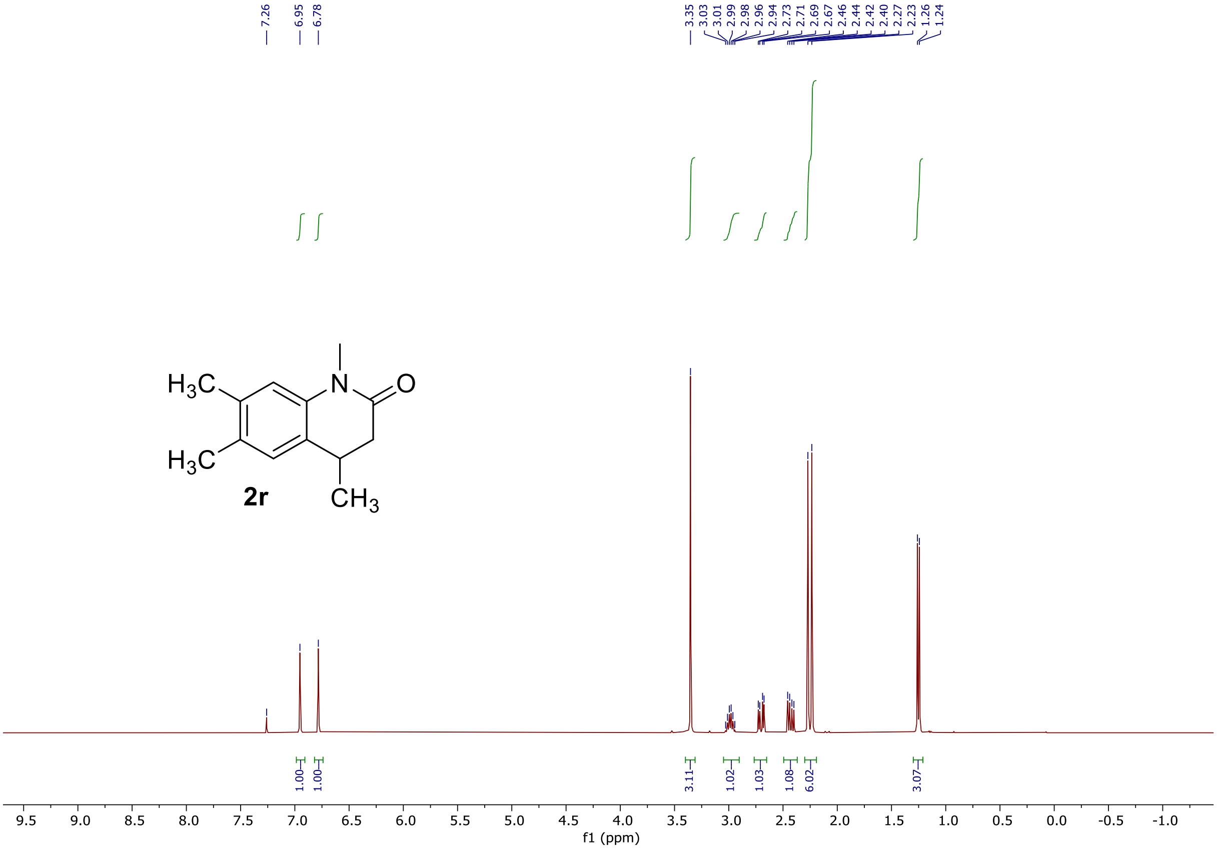
7.26
6.95
6.78
3.35
3.03
3.01
2.99
2.98
2.96
2.94
2.73
2.71
2.69
2.67
2.46
2.44
2.42
2.40
2.27
2.23
1.26
1.24
H3C
H3C
N
O
2r
CH3
1.00
1.00
3.11
1.02
1.03
1.08
6.02
3.07
9.5 9.0 8.5 8.0 7.5 7.0 6.5 6.0 5.5 5.0 4.5 4.0 3.5 3.0 2.5 2.0 1.5 1.0 0.5 0.0 -0.5 -1.0
f1 (ppm)

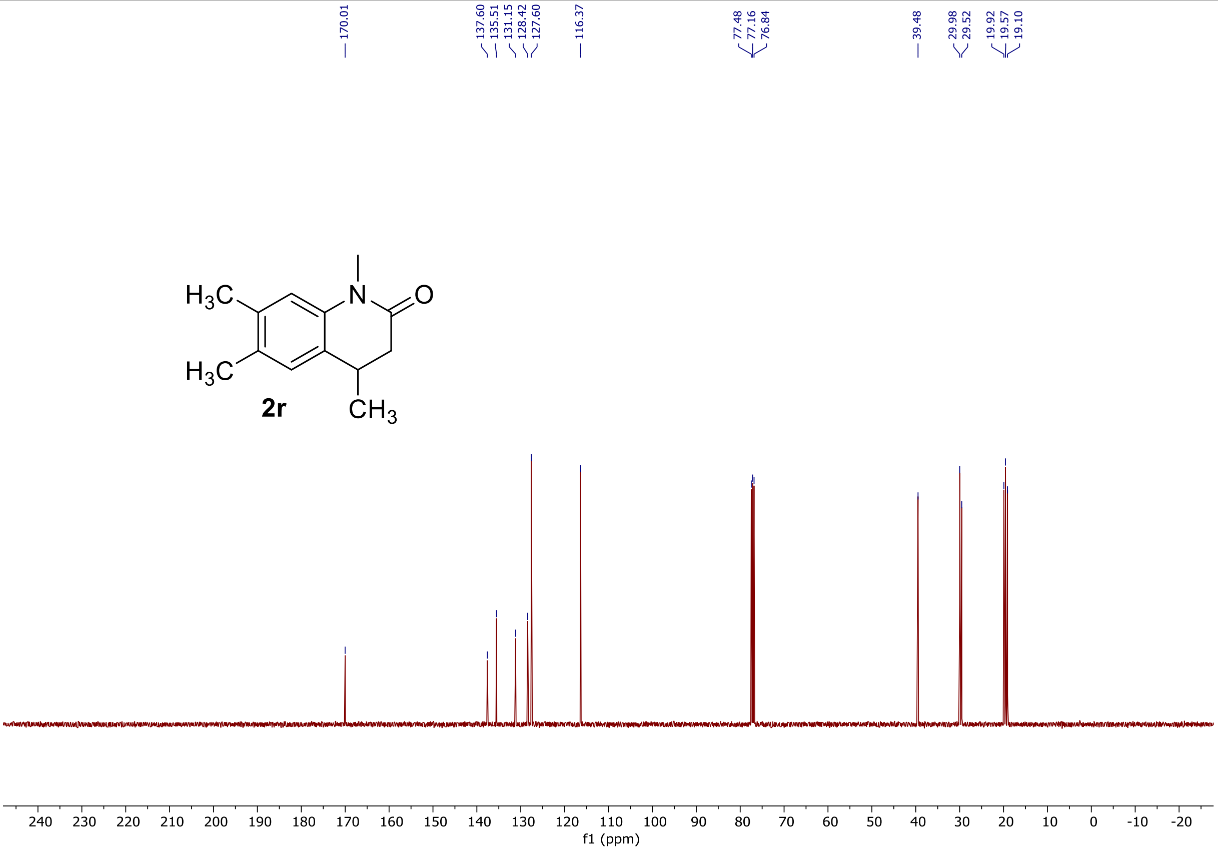

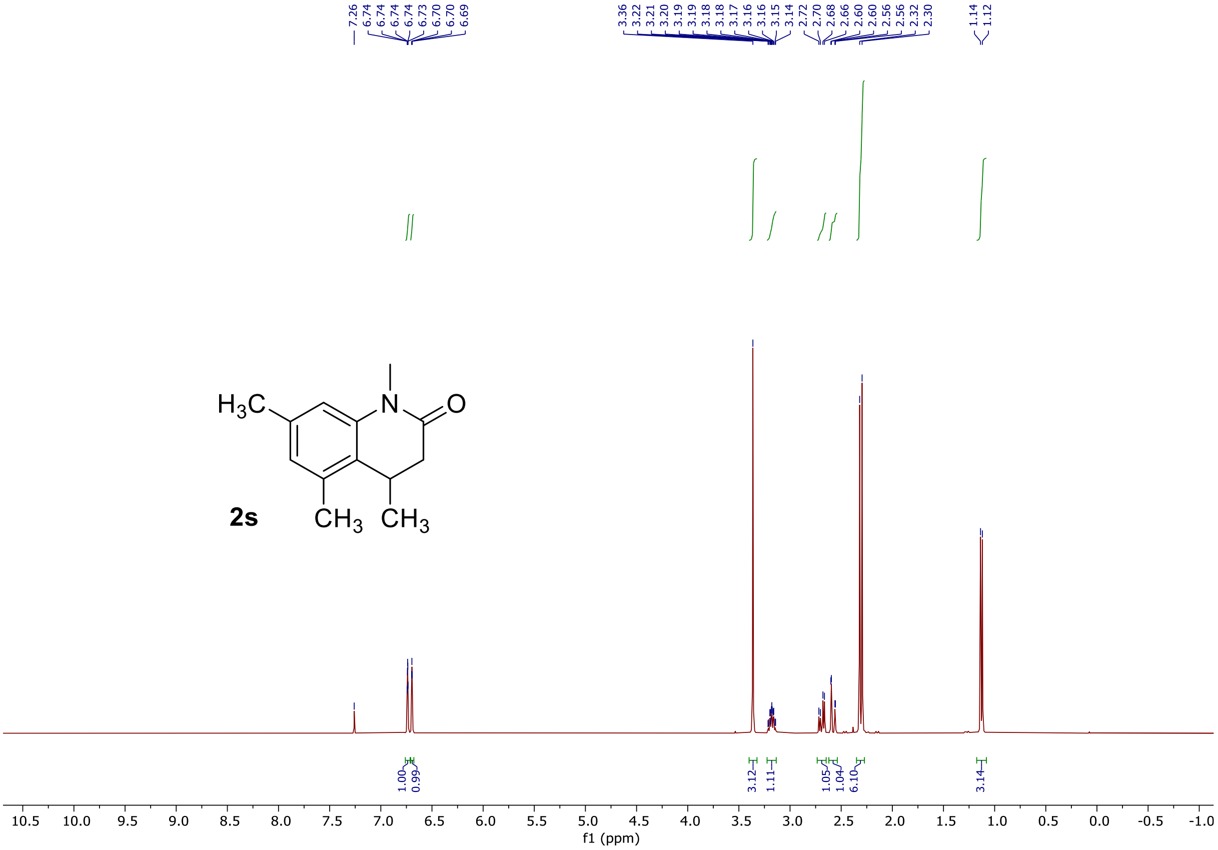

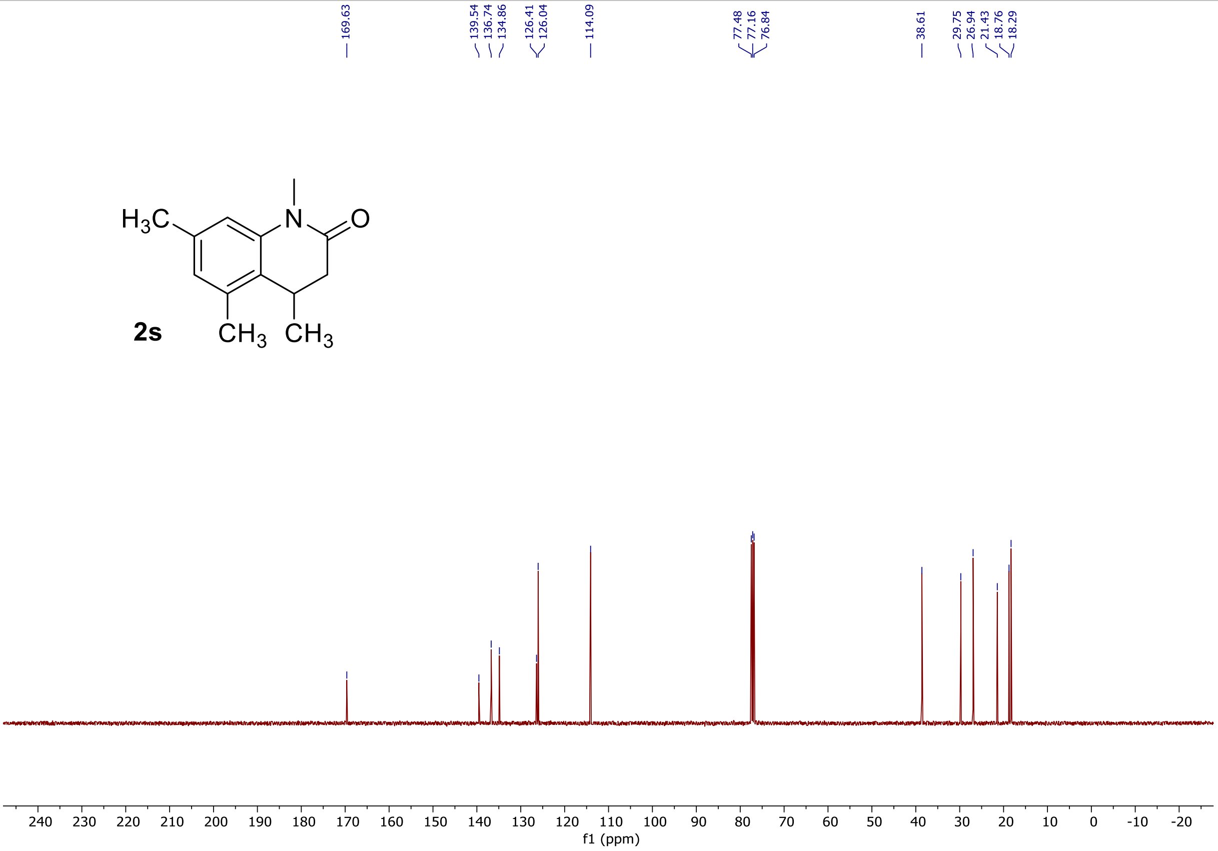
169.63
139.54
136.74
134.86
126.41
126.04
114.09
77.48
77.16
76.84
38.61
29.75
26.94
21.43
18.76
18.29
H3C
N
O
CH3 CH3
f1 (ppm)

2s

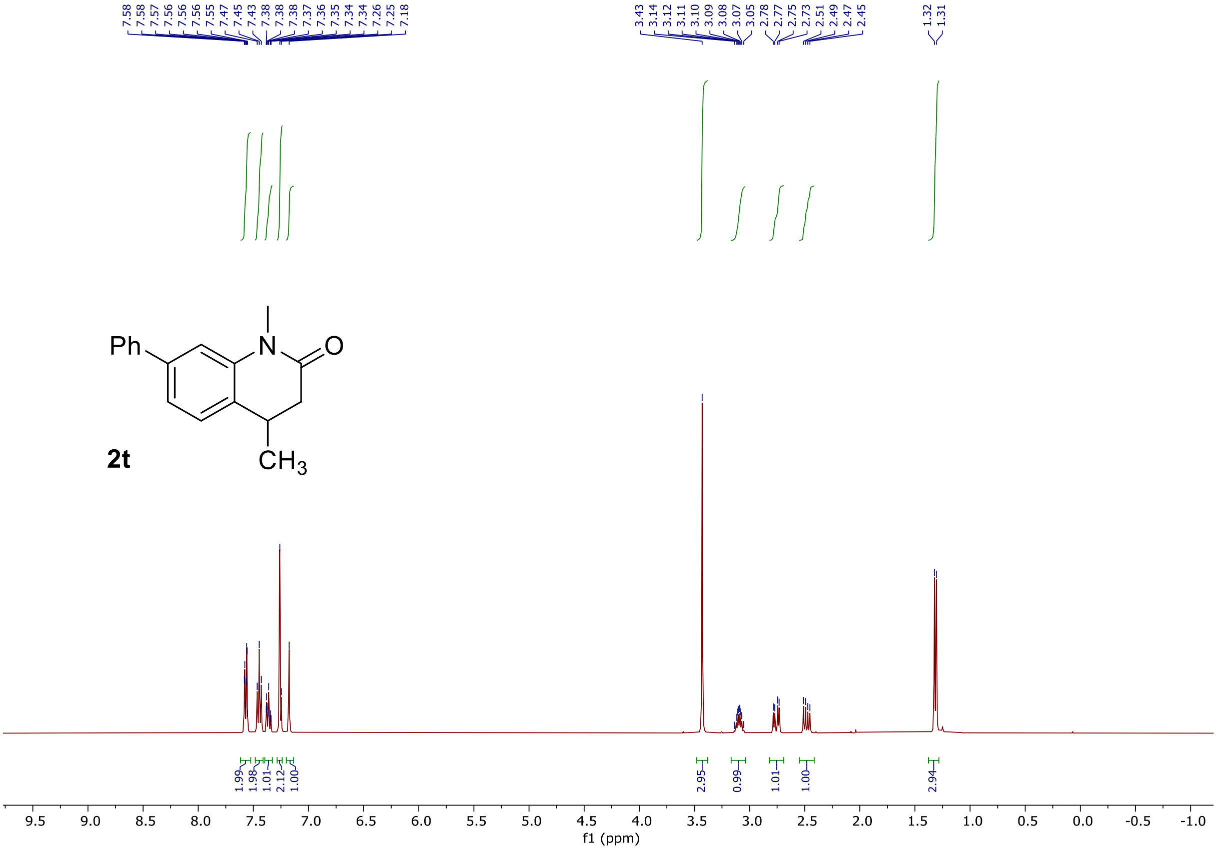
7.58 7.58 7.57 7.56 7.56 7.56 7.55 7.47 7.45 7.43 7.38 7.38 7.38 7.37 7.36 7.35 7.34 7.34 7.26 7.25 7.18
3.43 3.14 3.12 3.11 3.10 3.09 3.08 3.07 3.05 2.78 2.77 2.75 2.73 2.51 2.49 2.47 2.45
1.32 1.31
Ph
N
O
CH3
1.99 1.98 1.01 2.12 1.00
2.95 0.99 1.01 1.00
2.94
f1 (ppm)

2t

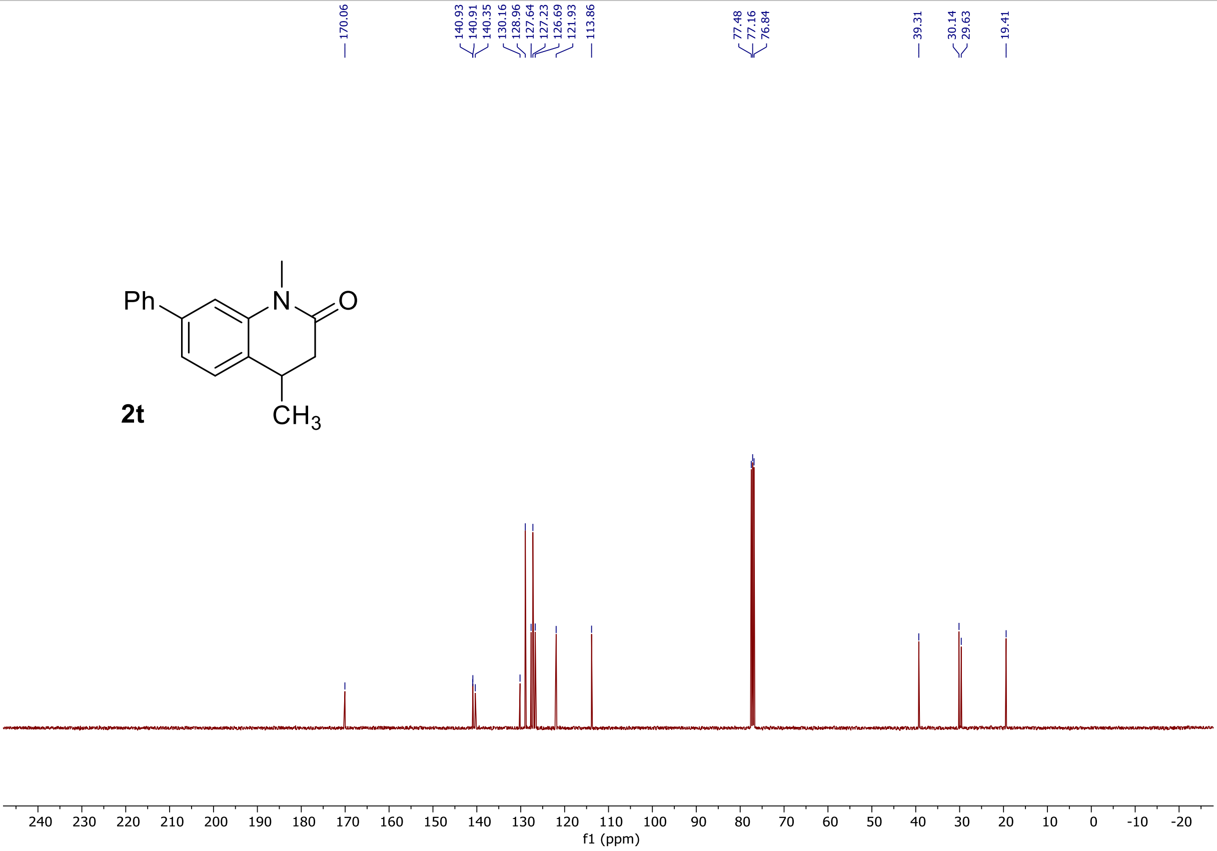
170.06
140.93
140.91
140.35
130.16
128.96
127.64
127.23
126.69
121.93
113.86
77.48
77.16
76.84
39.31
30.14
29.63
19.41
Ph
N
O
2t
CH3
240 230 220 210 200 190 180 170 160 150 140 130 120 110 100 90 80 70 60 50 40 30 20 10 0 -10 -20
f1 (ppm)

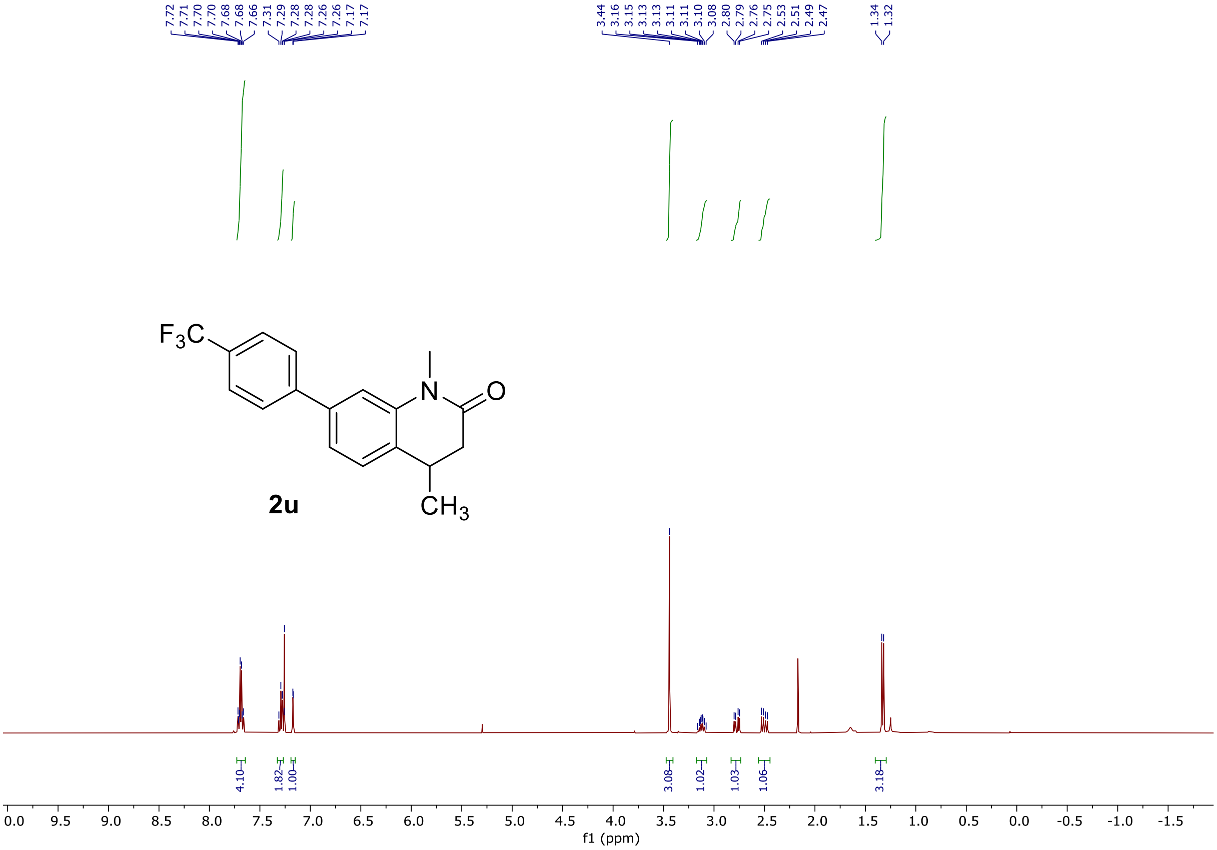
7.72
7.71
7.70
7.68
7.68
7.66
7.31
7.29
7.28
7.28
7.26
7.26
7.17
7.17
3.44
3.16
3.15
3.13
3.13
3.11
3.11
3.10
3.08
2.80
2.79
2.76
2.75
2.53
2.51
2.49
2.47
1.34
1.32
F3C
N
O
2u
CH3
4.10
1.82
1.00
3.08
1.02
1.03
1.06
3.18
0.0 9.5 9.0 8.5 8.0 7.5 7.0 6.5 6.0 5.5 5.0 4.5 4.0 3.5 3.0 2.5 2.0 1.5 1.0 0.5 0.0 -0.5 -1.0 -1.5
f1 (ppm)

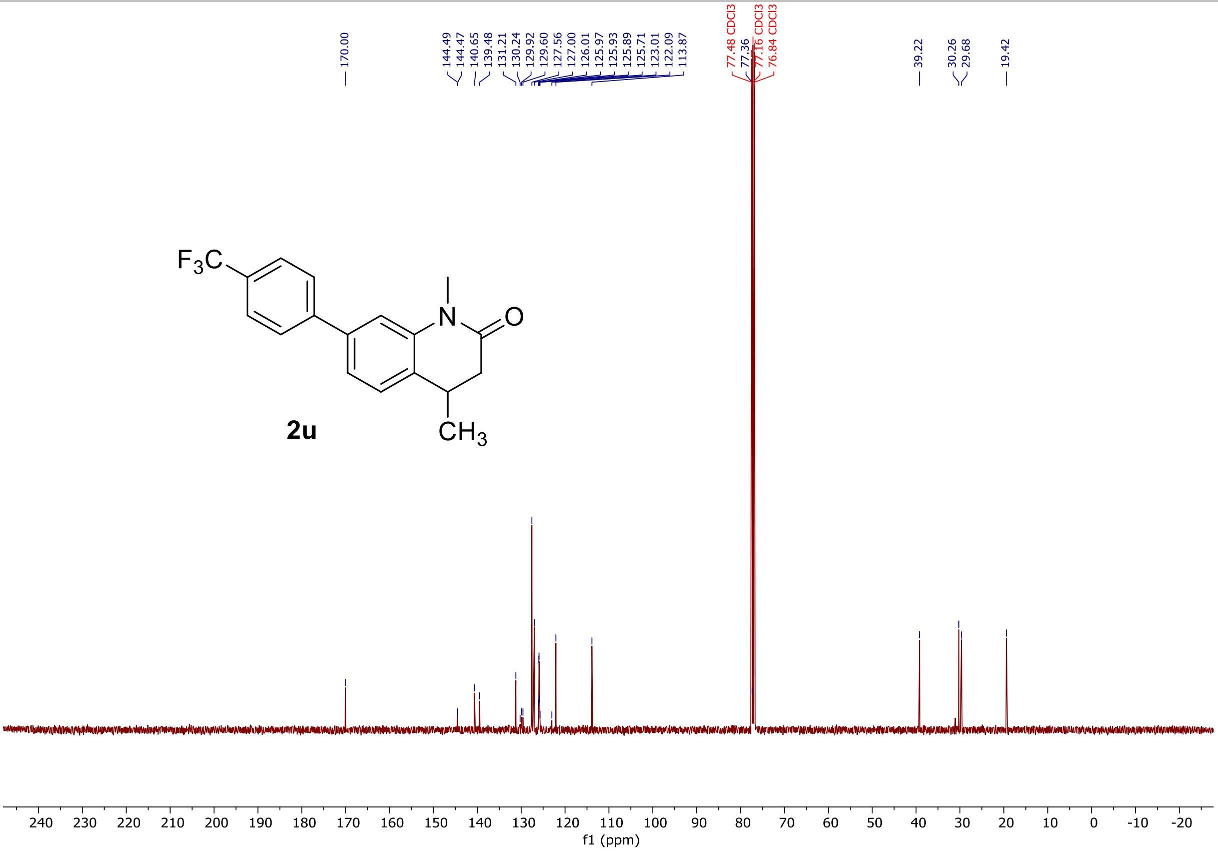
170.00
144.49
144.47
140.65
139.48
131.21
130.24
129.92
129.60
127.56
127.00
126.01
125.97
125.93
125.89
125.71
123.01
122.09
113.87
77.48 CDCl3
77.36
77.16 CDCl3
76.84 CDCl3
39.22
30.26
29.68
19.42
F3C
N
O
CH3
2u
f1 (ppm)

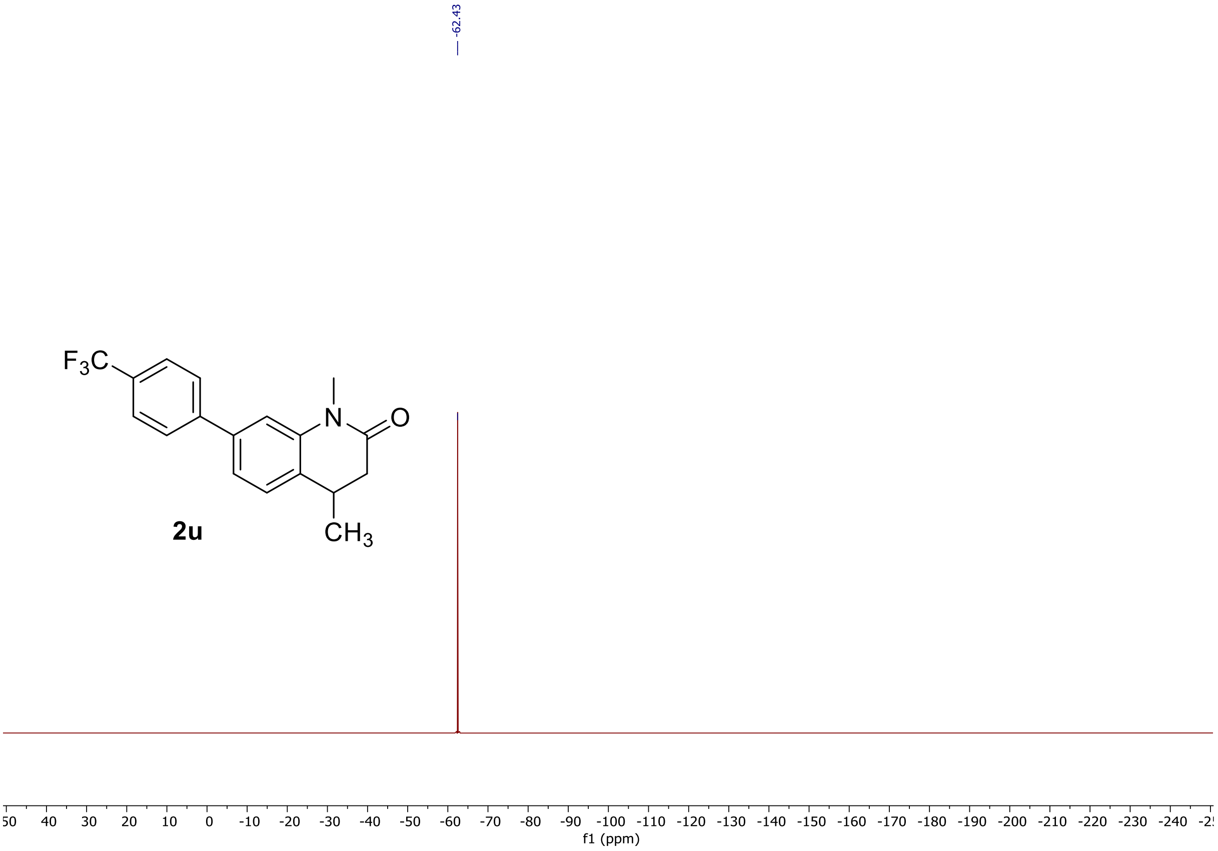
-62.43
F3C
N
O
CH3
2u
f1 (ppm)

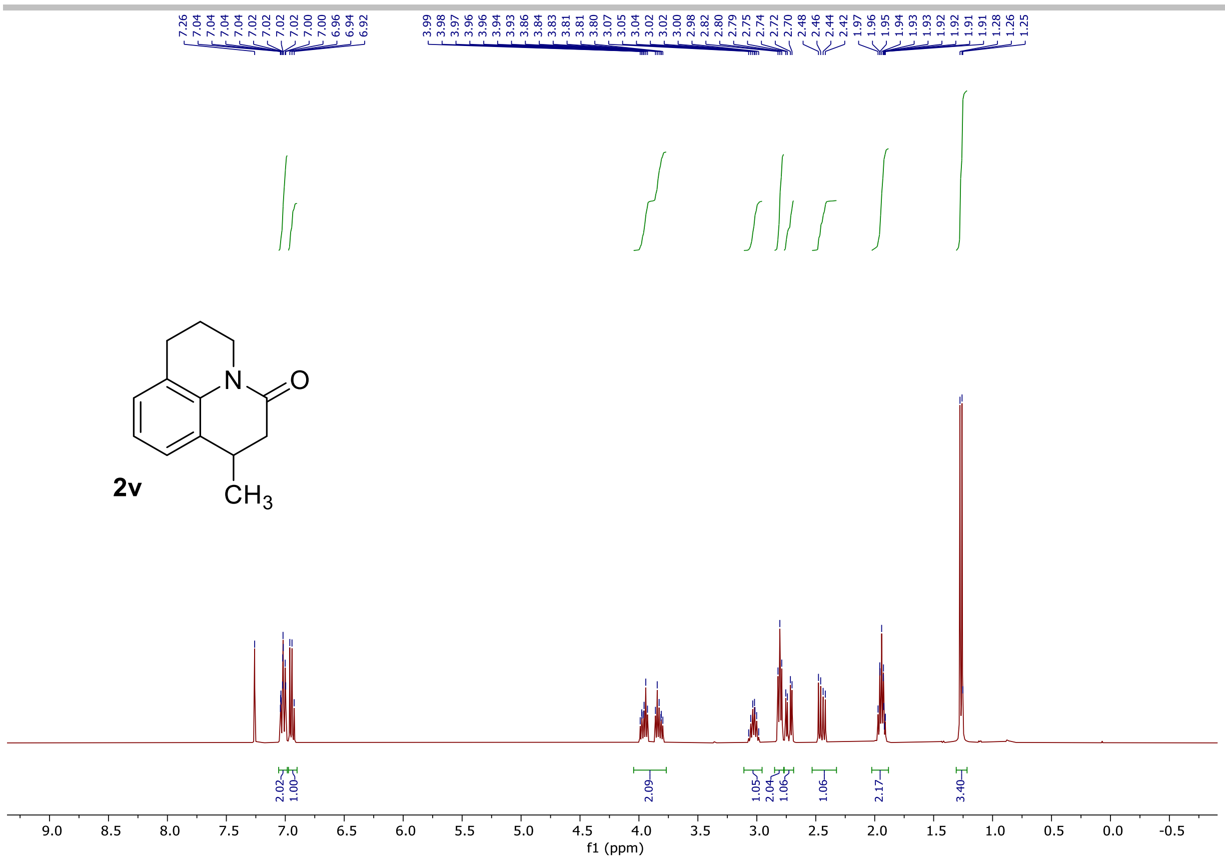
7.26 7.04 7.04 7.04 7.04 7.02 7.02 7.02 7.02 7.00 7.00 6.96 6.94 6.92
3.99 3.98 3.97 3.96 3.96 3.94 3.93 3.93 3.86 3.84 3.83 3.81 3.81 3.80 3.07 3.05 3.04 3.02 3.02 3.02 3.00 2.98 2.82 2.80 2.79 2.75 2.74 2.72 2.70 2.48 2.46 2.44 2.42 1.97 1.96 1.95 1.94 1.93 1.93 1.92 1.92 1.91 1.91 1.28 1.26 1.25
N
O
2v
CH3
2.02
1.00
2.09
1.05
2.04
1.06
1.06
2.17
3.40
f1 (ppm)

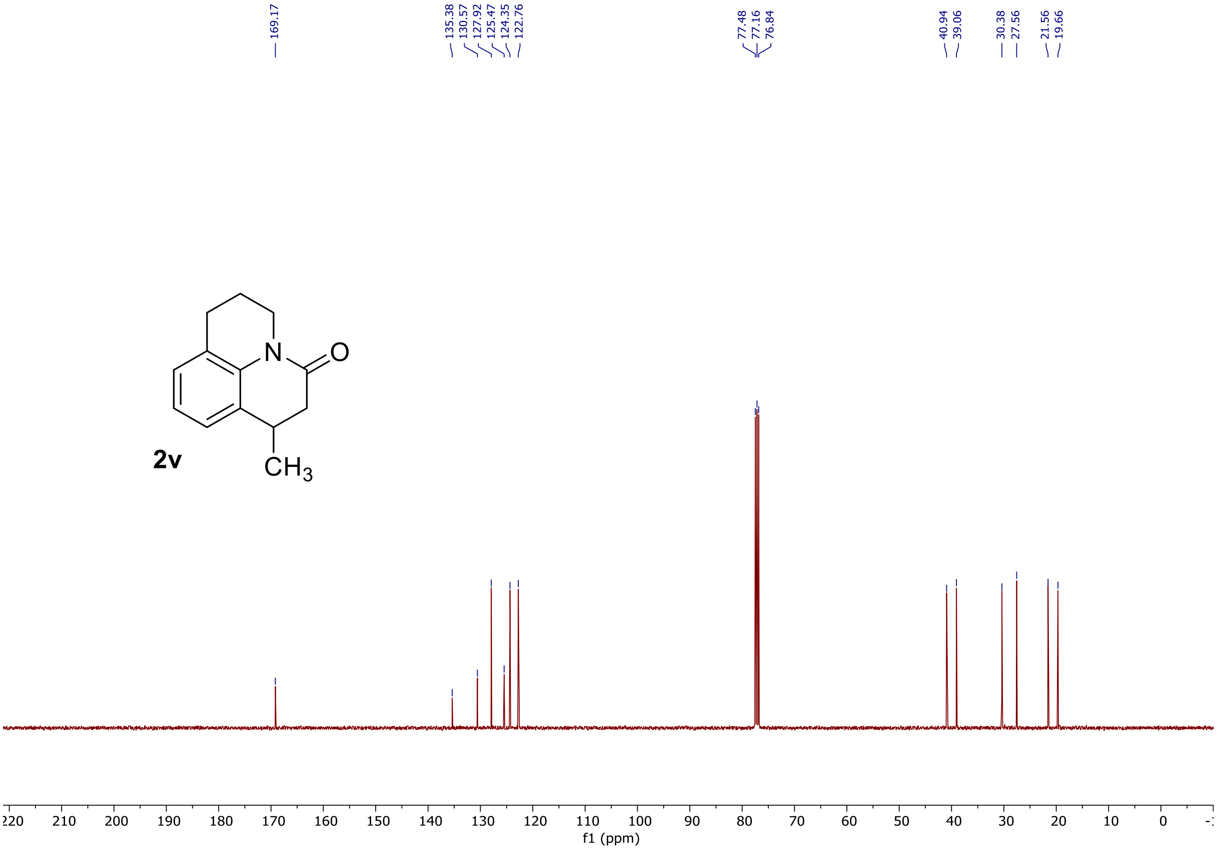
169.17
135.38 130.57 127.92 125.47 124.35 122.76
77.48 77.16 76.84
40.94 39.06
30.38 27.56
21.56 19.66
N
O
2v
CH3
f1 (ppm)

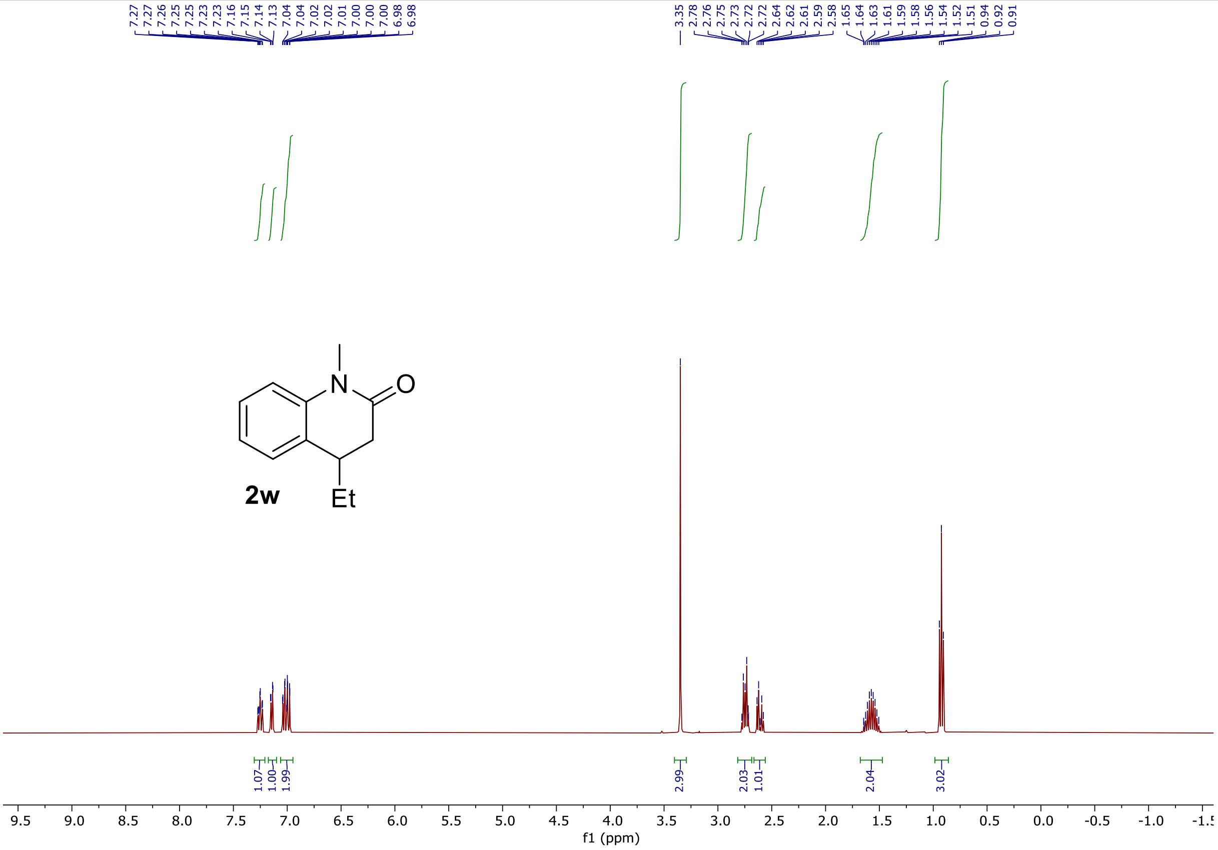
7.27
7.27
7.26
7.25
7.25
7.23
7.23
7.16
7.15
7.14
7.13
7.04
7.04
7.02
7.02
7.01
7.00
7.00
7.00
6.98
6.98
3.35
2.78
2.76
2.75
2.73
2.72
2.72
2.64
2.62
2.61
2.59
2.58
1.65
1.64
1.63
1.61
1.59
1.58
1.56
1.54
1.52
1.51
0.94
0.92
0.91
2w
Et
N
O
1.07
1.00
1.99
2.99
2.03
1.01
2.04
3.02
f1 (ppm)

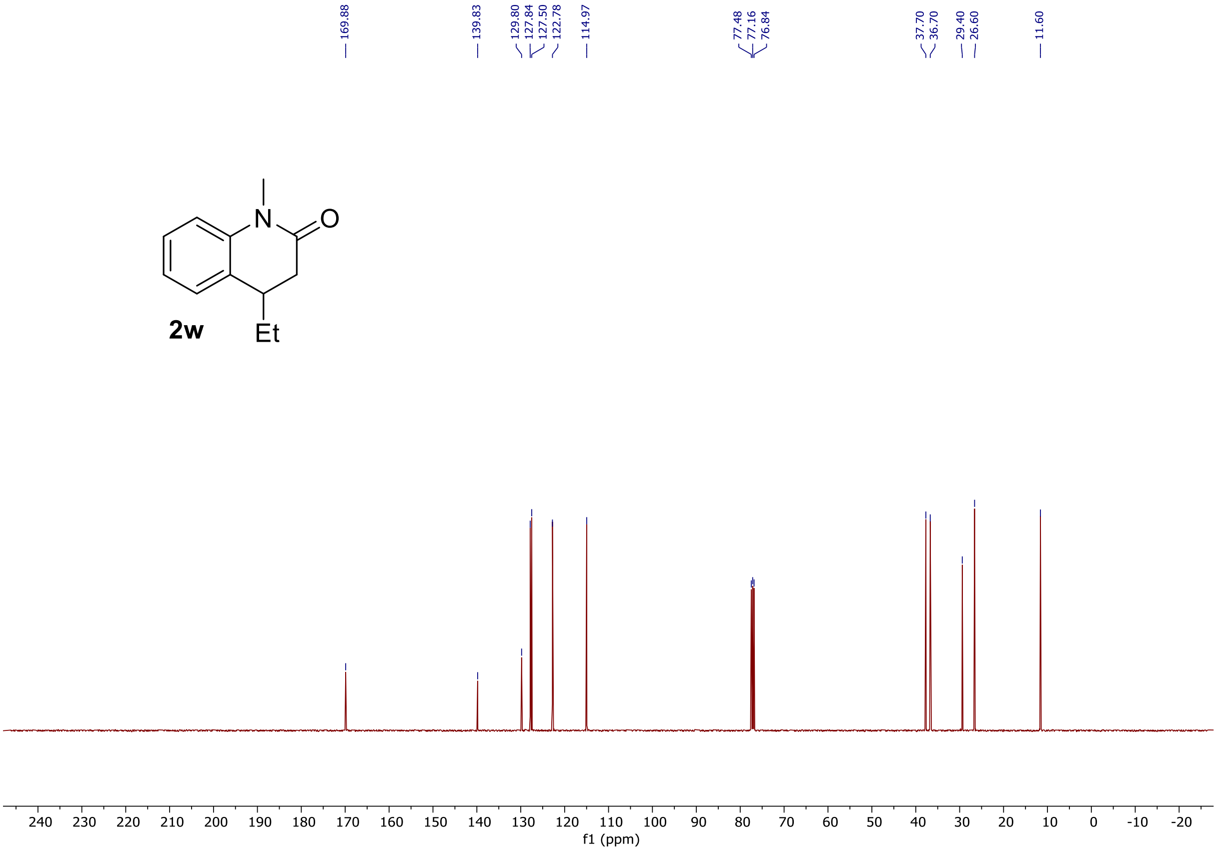
169.88
139.83
129.80
127.84
127.50
122.78
114.97
77.48
77.16
76.84
37.70
36.70
29.40
26.60
11.60
2w
Et
N
O
f1 (ppm)

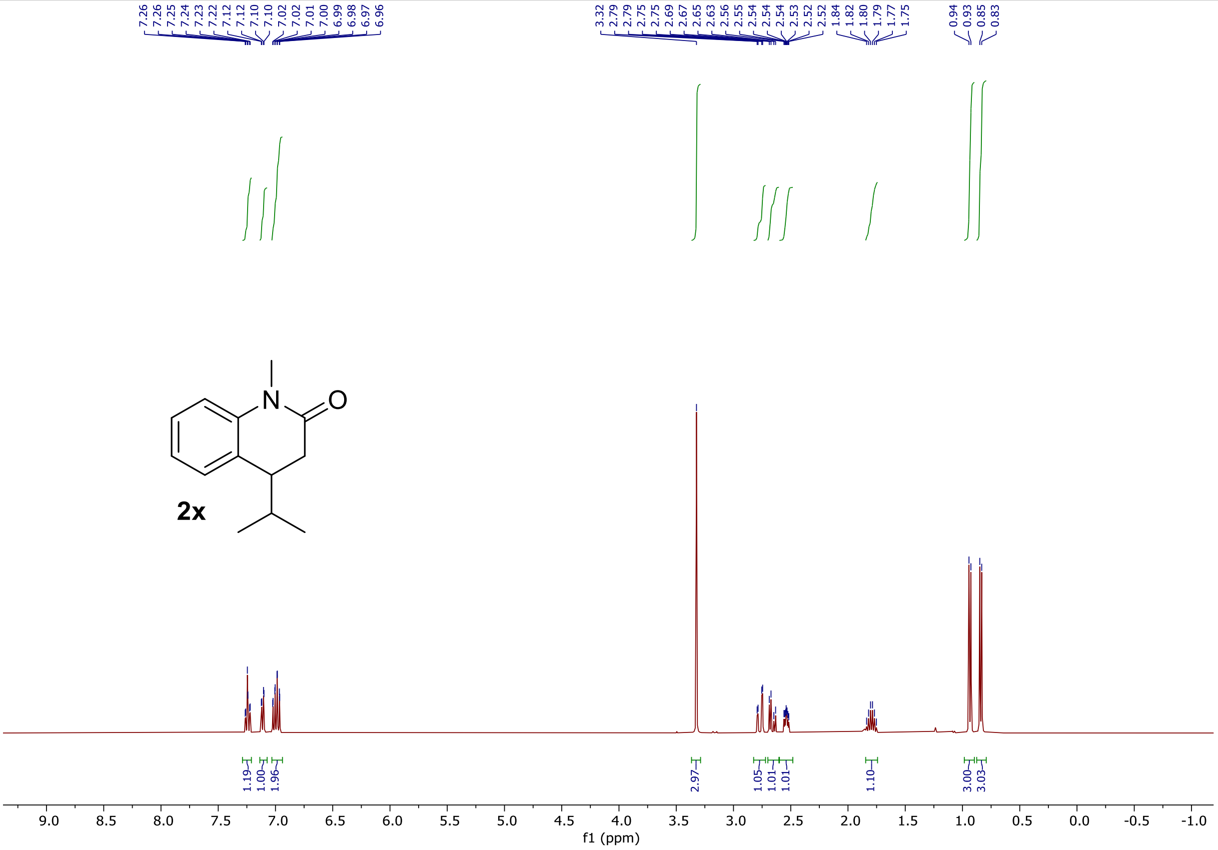
7.26
7.26
7.25
7.24
7.23
7.22
7.12
7.12
7.10
7.10
7.02
7.02
7.01
7.00
6.99
6.98
6.97
6.96
3.32
2.79
2.79
2.75
2.75
2.69
2.67
2.65
2.63
2.56
2.55
2.54
2.54
2.54
2.53
2.52
2.52
1.84
1.82
1.80
1.79
1.77
1.75
0.94
0.93
0.85
0.83
2x
1.19
1.00
1.96
2.97
1.05
1.01
1.01
1.10
3.00
3.03
9.0 8.5 8.0 7.5 7.0 6.5 6.0 5.5 5.0 4.5 4.0 3.5 3.0 2.5 2.0 1.5 1.0 0.5 0.0 -0.5 -1.0
f1 (ppm)

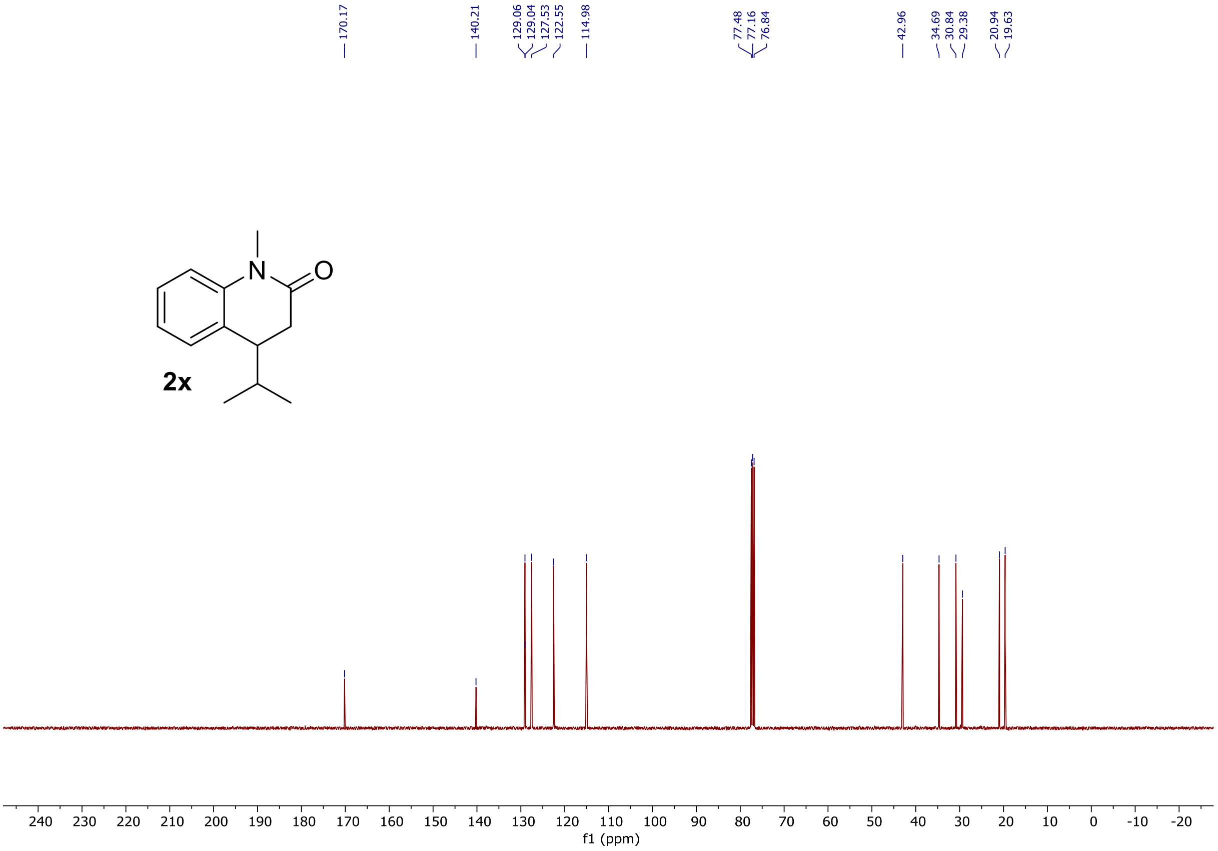
170.17
140.21
129.06
129.04
127.53
122.55
114.98
77.48
77.16
76.84
42.96
34.69
30.84
29.38
20.94
19.63
2x
240 230 220 210 200 190 180 170 160 150 140 130 120 110 100 90 80 70 60 50 40 30 20 10 0 -10 -20
f1 (ppm)

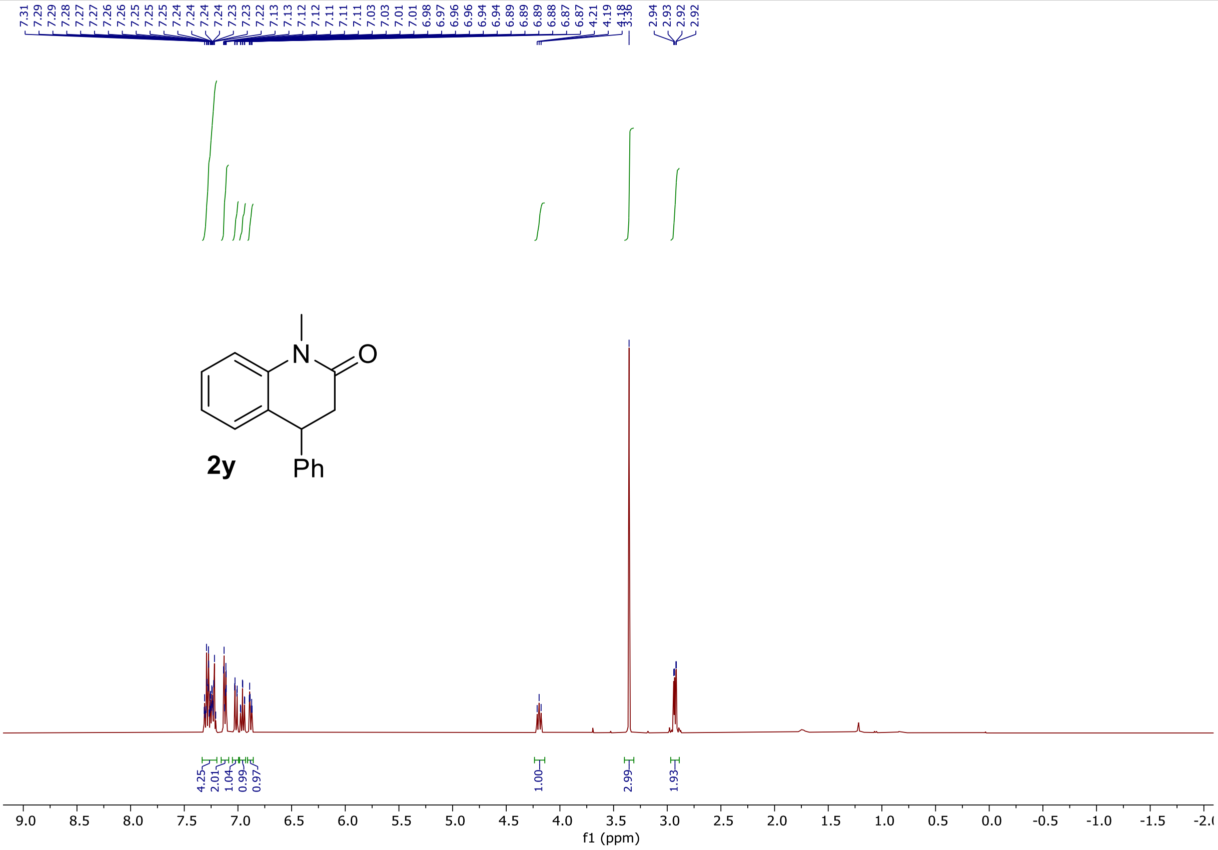
7.31 7.29 7.29 7.28 7.27 7.27 7.26 7.26 7.25 7.25 7.25 7.24 7.24 7.24 7.24 7.23 7.23 7.22 7.13 7.13 7.12 7.12 7.11 7.11 7.11 7.03 7.03 7.01 7.01 6.98 6.97 6.96 6.96 6.94 6.94 6.94 6.89 6.89 6.89 6.88 6.87 6.87 4.21 4.19 4.18 3.38 2.94 2.93 2.92 2.92
2y
Ph
4.25 2.01 1.04 0.99 0.97 1.00 2.99 1.93
f1 (ppm)

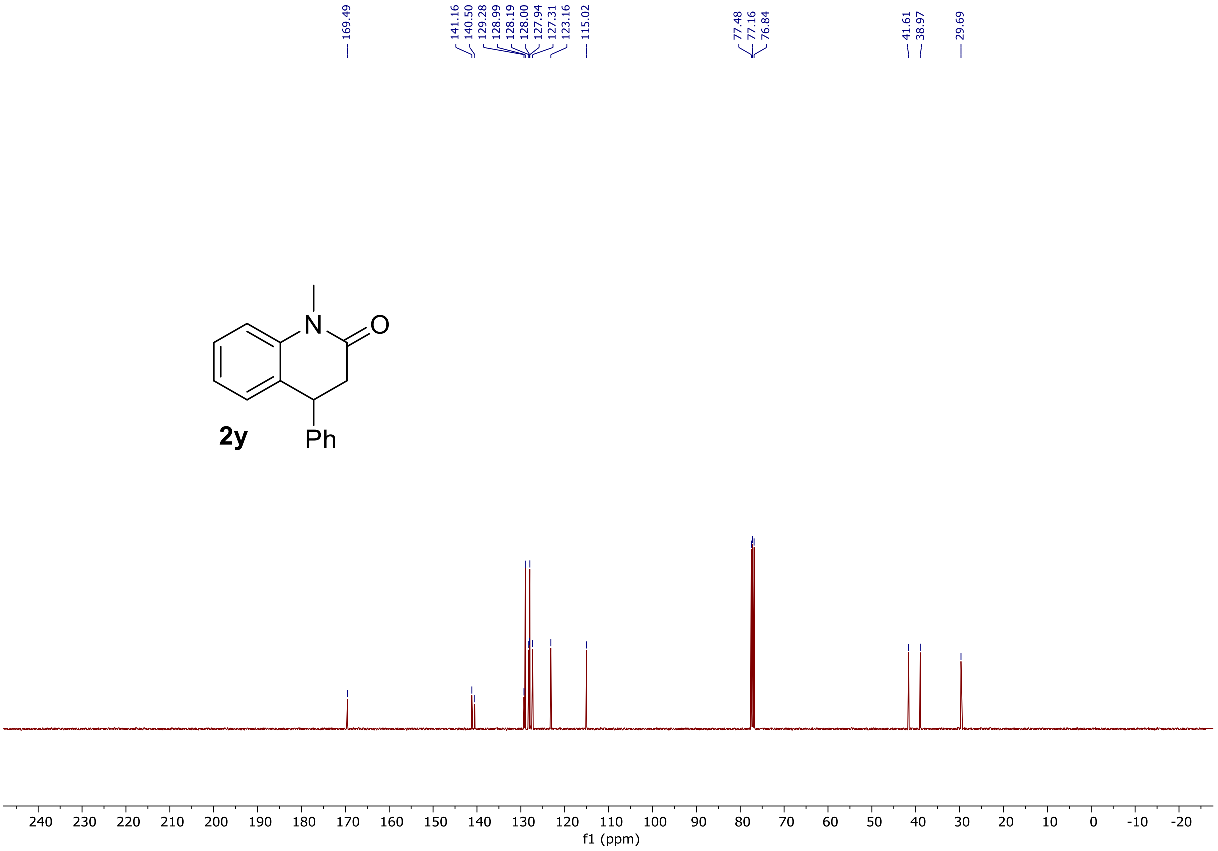
169.49
141.16 140.50 129.28 128.99 128.19 128.00 127.94 127.31 123.16 115.02
77.48 77.16 76.84
41.61 38.97 29.69
2y
Ph
f1 (ppm)

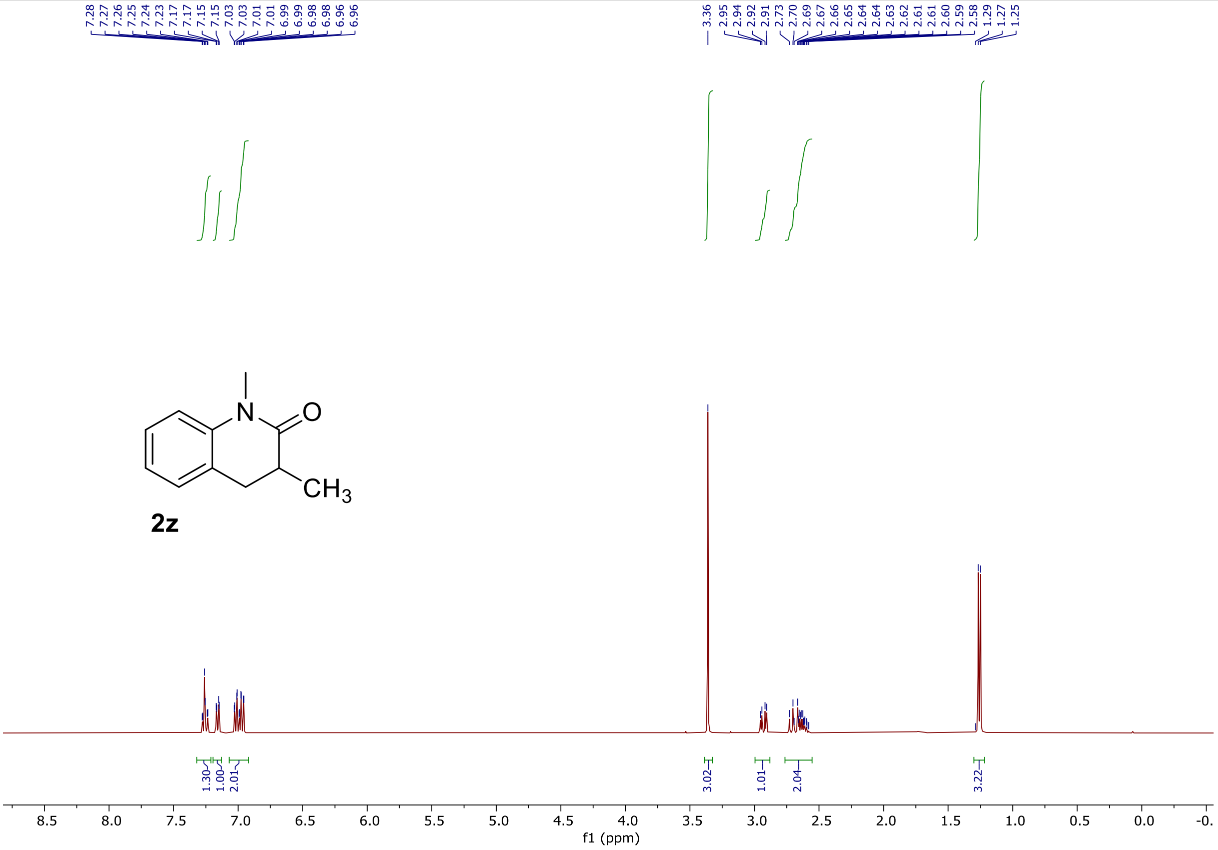
7.28 7.27 7.26 7.25 7.24 7.23 7.17 7.17 7.15 7.15 7.03 7.03 7.01 7.01 6.99 6.99 6.98 6.98 6.96 6.96
3.36 2.95 2.94 2.92 2.91 2.73 2.70 2.69 2.67 2.66 2.65 2.64 2.64 2.63 2.62 2.61 2.61 2.60 2.59 2.58 1.29 1.27 1.25
N
O
CH3
2z
1.30 1.00 2.01 3.02 1.01 2.04 3.22
8.5 8.0 7.5 7.0 6.5 6.0 5.5 5.0 4.5 4.0 3.5 3.0 2.5 2.0 1.5 1.0 0.5 0.0 -0.
f1 (ppm)

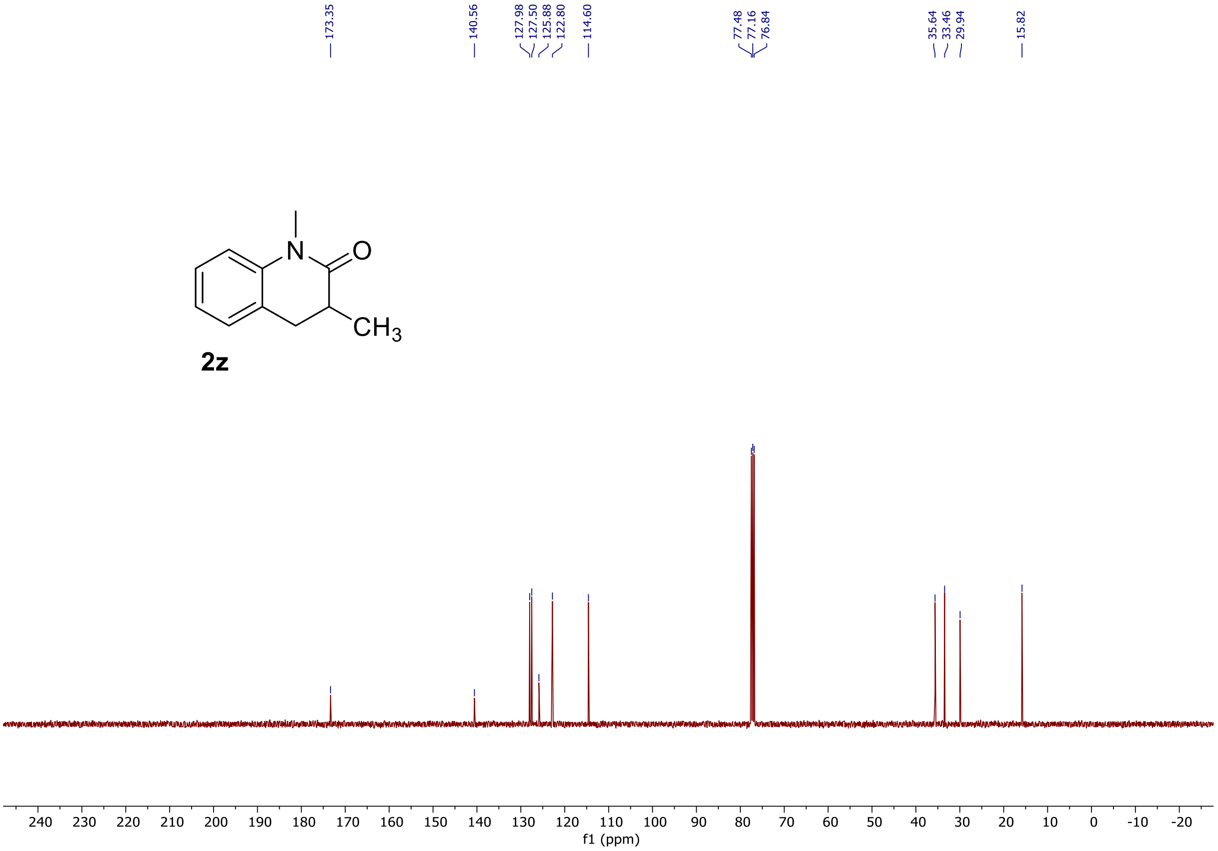
173.35 140.56 127.98 127.50 125.88 122.80 114.60 77.48 77.16 76.84 35.64 33.46 29.94 15.82
N
O
CH3
2z
240 230 220 210 200 190 180 170 160 150 140 130 120 110 100 90 80 70 60 50 40 30 20 10 0 -10 -20
f1 (ppm)

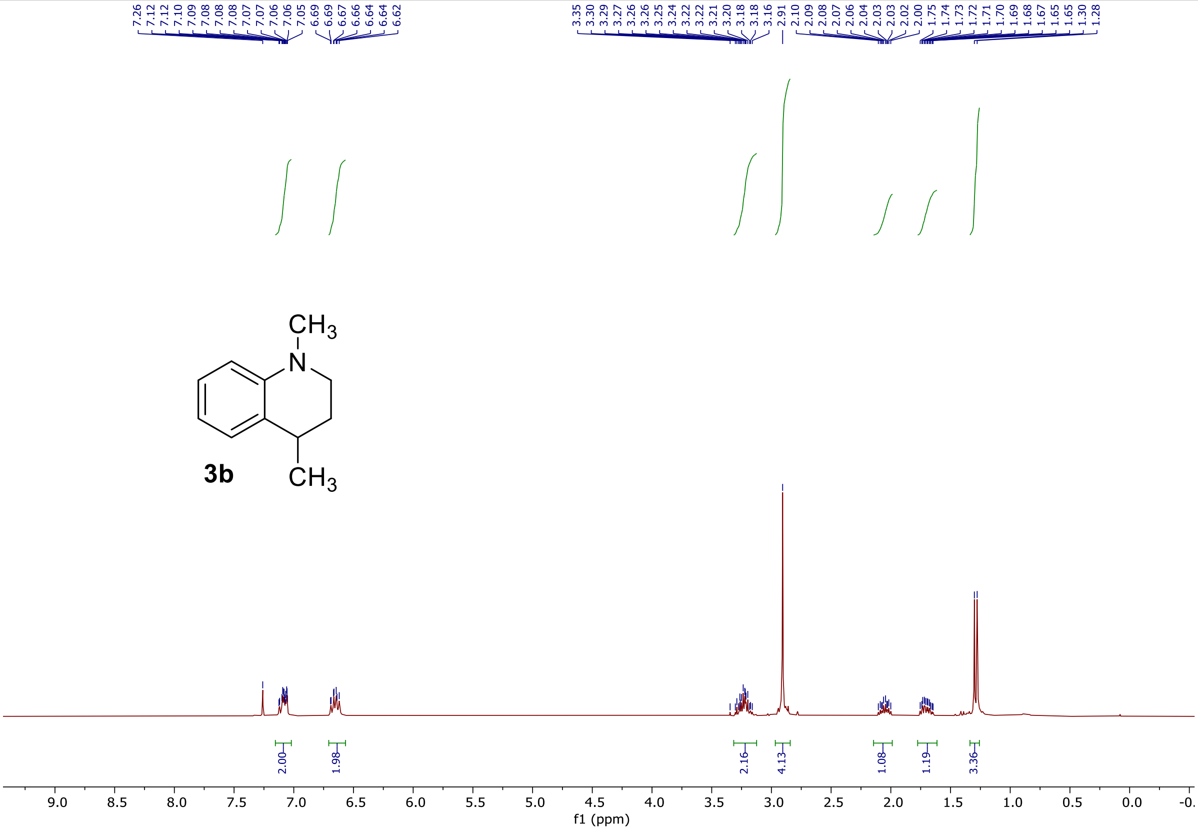
7.26
7.12
7.12
7.10
7.09
7.08
7.08
7.08
7.07
7.06
7.06
7.05
6.69
6.69
6.67
6.66
6.64
6.64
6.62
3.35
3.30
3.29
3.27
3.26
3.26
3.25
3.24
3.22
3.22
3.21
3.20
3.18
3.18
3.16
2.91
2.10
2.09
2.08
2.07
2.06
2.04
2.03
2.03
2.02
2.02
2.00
1.75
1.74
1.73
1.72
1.71
1.70
1.69
1.68
1.67
1.65
1.65
1.30
1.28
CH3
N
3b
CH3
2.00
1.98
2.16
4.13
1.08
1.19
3.36
f1 (ppm)

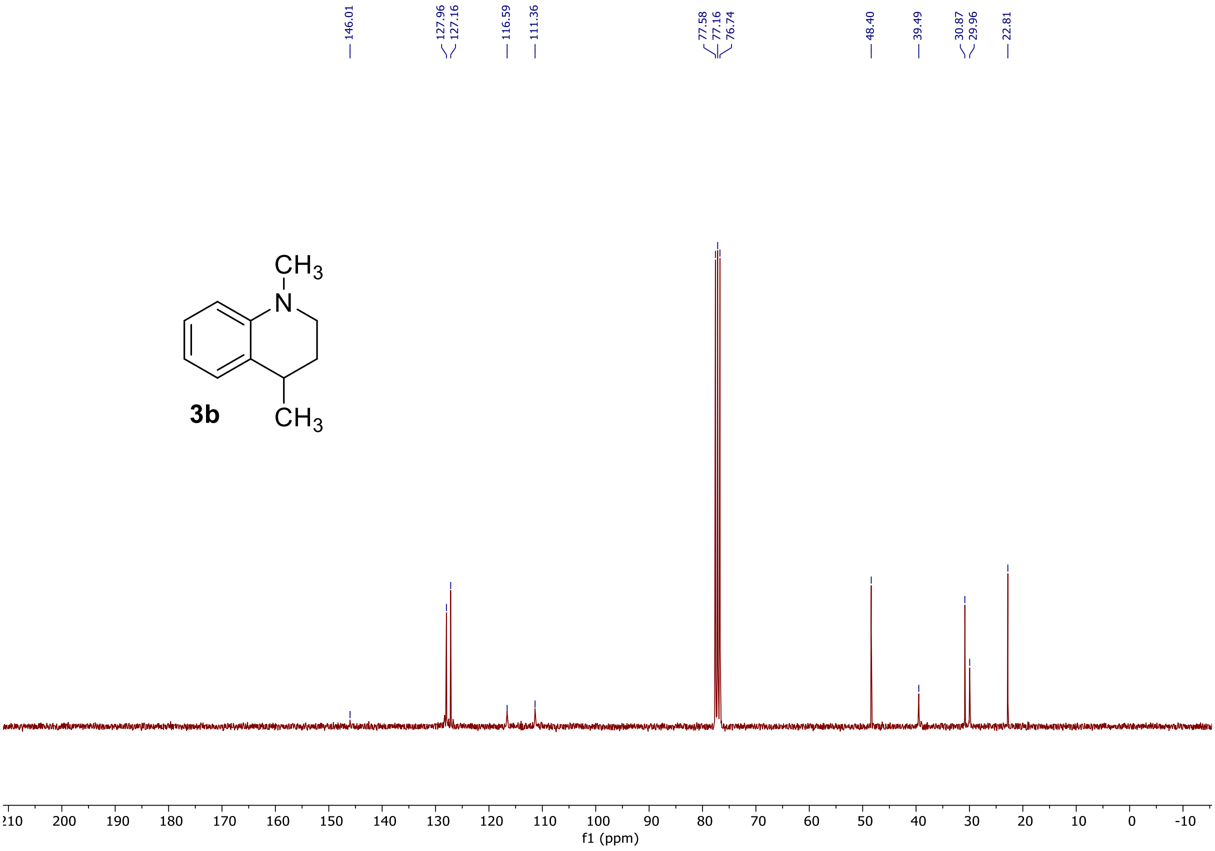
146.01
127.96
127.16
116.59
111.36
77.58
77.16
76.74
48.40
39.49
30.87
29.96
22.81
CH3
N
3b
CH3
f1 (ppm)

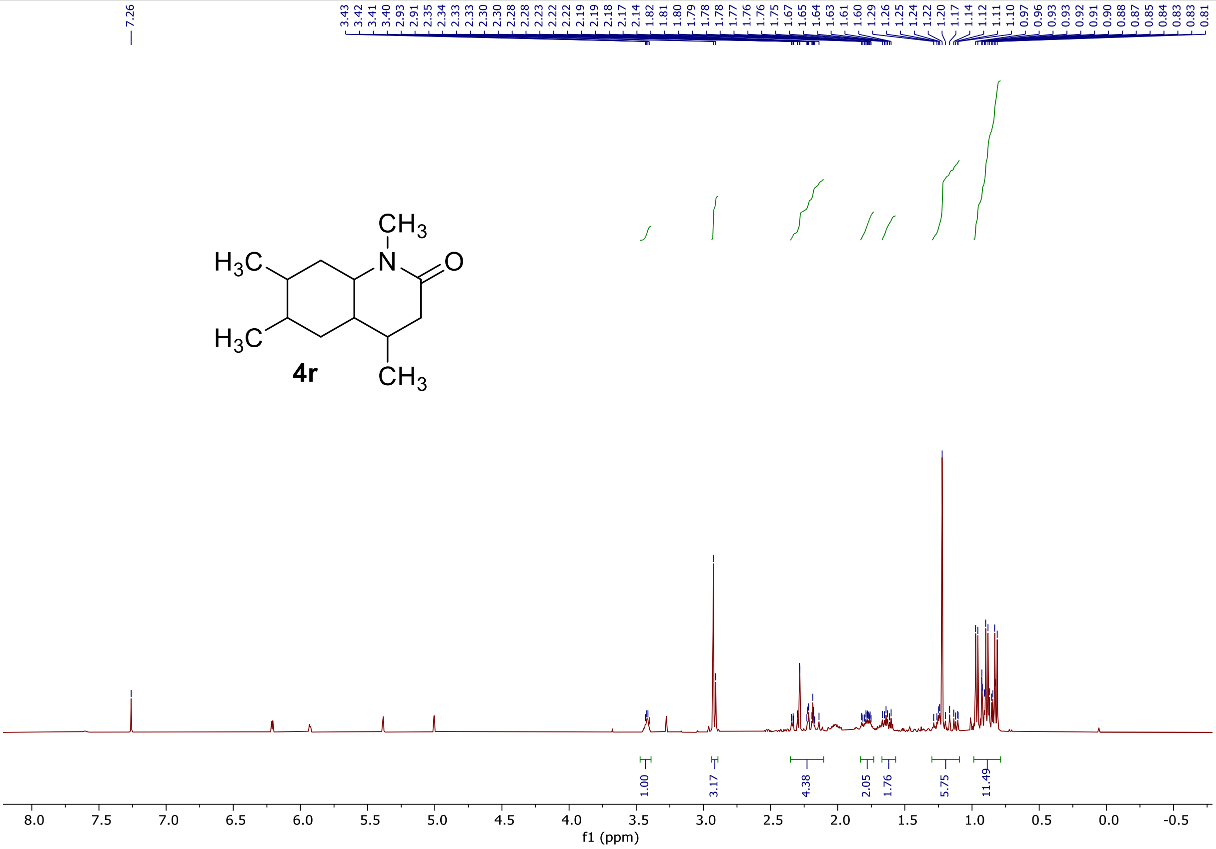
7.26
3.43 3.42 3.41 3.40 2.93 2.91 2.35 2.34 2.33 2.33 2.30 2.30 2.28 2.28 2.23 2.22 2.22 2.19 2.19 2.18 2.17 2.14 1.82 1.81 1.80 1.79 1.78 1.78 1.77 1.76 1.76 1.75 1.67 1.65 1.64 1.63 1.61 1.60 1.29 1.26 1.25 1.24 1.22 1.20 1.17 1.14 1.12 1.11 1.10 0.97 0.96 0.93 0.93 0.92 0.91 0.90 0.88 0.87 0.85 0.84 0.83 0.83 0.81
CH3
H3C
N
O
H3C
4r
CH3
1.00
3.17
4.38
2.05
1.76
5.75
11.49
8.0 7.5 7.0 6.5 6.0 5.5 5.0 4.5 4.0 3.5 3.0 2.5 2.0 1.5 1.0 0.5 0.0 -0.5
f1 (ppm)

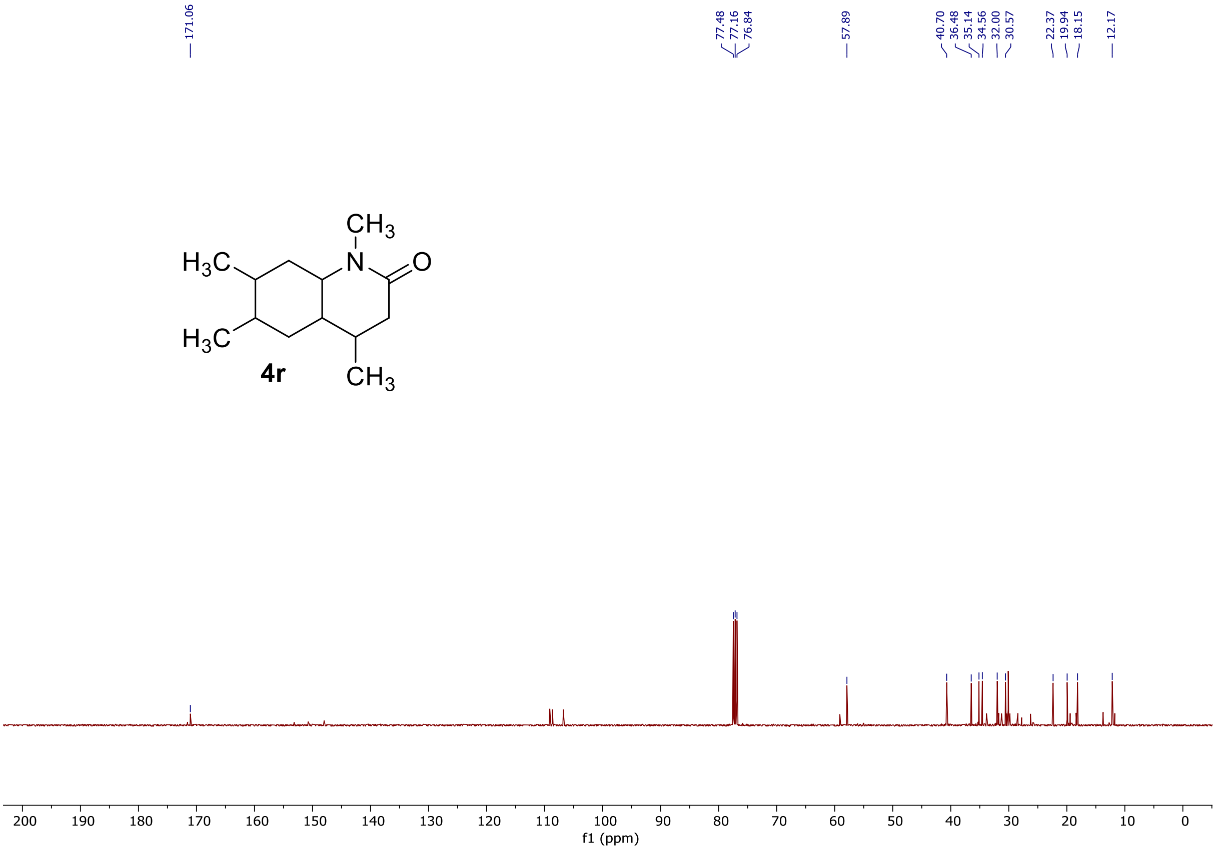
171.06
77.48 77.16 76.84
57.89
40.70 36.48 35.14 34.56 32.00 30.57
22.37 19.94 18.15
12.17
CH3
H3C
N
O
H3C
4r
CH3
200 190 180 170 160 150 140 130 120 110 100 90 80 70 60 50 40 30 20 10 0
f1 (ppm)
